# Synthesis of Fe^III^ and Fe^IV^ Cyanide Complexes Using Hypervalent Iodine Reagents as Cyano‐Transfer One‐Electron Oxidants

**DOI:** 10.1002/anie.202201699

**Published:** 2022-03-29

**Authors:** Charafa Souilah, Sergio A. V. Jannuzzi, Derya Demirbas, Sergei Ivlev, Marcel Swart, Serena DeBeer, Alicia Casitas

**Affiliations:** ^1^ Fachbereich Chemie Philipps-Universität Marburg Hans-Meerwein-Straße 4 35043 Marburg Germany; ^2^ Max Planck Institute for Chemical Energy Conversion (MPI CEC) Stiftstraße 34–36 45470 Mülheim an der Ruhr Germany; ^3^ ICREA Pg. Lluís Companys 23 08010 Barcelona Spain; ^4^ Institut de Química Computacional i Catàlisi, Facultat de Ciències Universitat de Girona c/ M.A. Capmany 69 17003 Girona Spain

**Keywords:** Group-Transfer Reactions, Hypervalent Compounds, Iron, Iron(IV) Cyanide, Single-Electron Transfer

## Abstract

We disclose a new reactivity mode for electrophilic cyano λ^3^‐iodanes as group transfer one‐electron oxidants to synthesize Fe^III^ and Fe^IV^ cyanide complexes. The inherent thermal instability of high‐valent Fe^IV^ compounds without π‐donor ligands (such as oxido (O^2−^), imido (RN^2−^) or nitrido (N^3−^)) makes their isolation and structural characterization a very challenging task. We report the synthesis of an Fe^IV^ cyanide complex [(N_3_N′)FeCN] (**4**) by two consecutive single electron transfer (SET) processes from Fe^II^ precursor [(N_3_N′)FeLi(THF)] (**1**) with cyanobenziodoxolone (CBX). The Fe^IV^ complex can also be prepared by reaction of [(N_3_N′)Fe^III^] (**3**) with CBX. In contrast, the oxidation of Fe^II^ with 1‐cyano‐3,3‐dimethyl‐3‐(1H)‐1,2‐benziodoxole (CDBX) enables the preparation of Fe^III^ cyanide complex [(N_3_N′)Fe^III^(CN)(Li)(THF)_3_] (**2‐Li^THF^**). Complexes **4** and **2‐Li^THF^** have been structurally characterized by single crystal X‐ray diffraction and their electronic structure has been examined by Mössbauer, EPR spectroscopy, and computational analyses.

## Graphical abstract

Cyano λ^3^‐iodanes react with iron(II) and iron(III) complexes as cyano‐transfer one‐electron oxidants. This approach enables the straightforward synthesis of iron(III) and iron(IV) cyanide complexes, which have been thoroughly characterized by spectroscopic methods, including X‐ray diffraction, EPR, Mössbauer and NMR spectroscopies and computational methods.

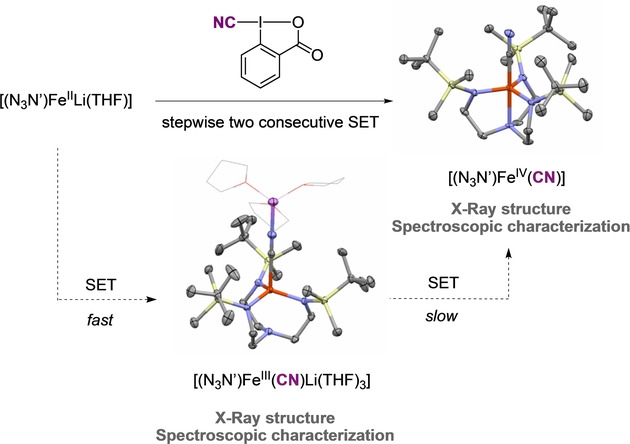

## Introduction

Iodine(III) compounds are powerful oxidants and versatile group transfer reagents that have many practical uses in organic synthesis.^[1]^ Owing to the high abundance, low price and low toxicity of iron, the activation of such iodine(III) reagents with this metal offers the opportunity to develop environmentally friendly bond‐forming catalytic methodologies.^[2]^ Despite the synthetic advances in this field, the mechanistic understanding of such reactions is limited, which hampers the rational design of novel and more efficient iron‐catalyzed group transfer reactions.

**Figure 1:**
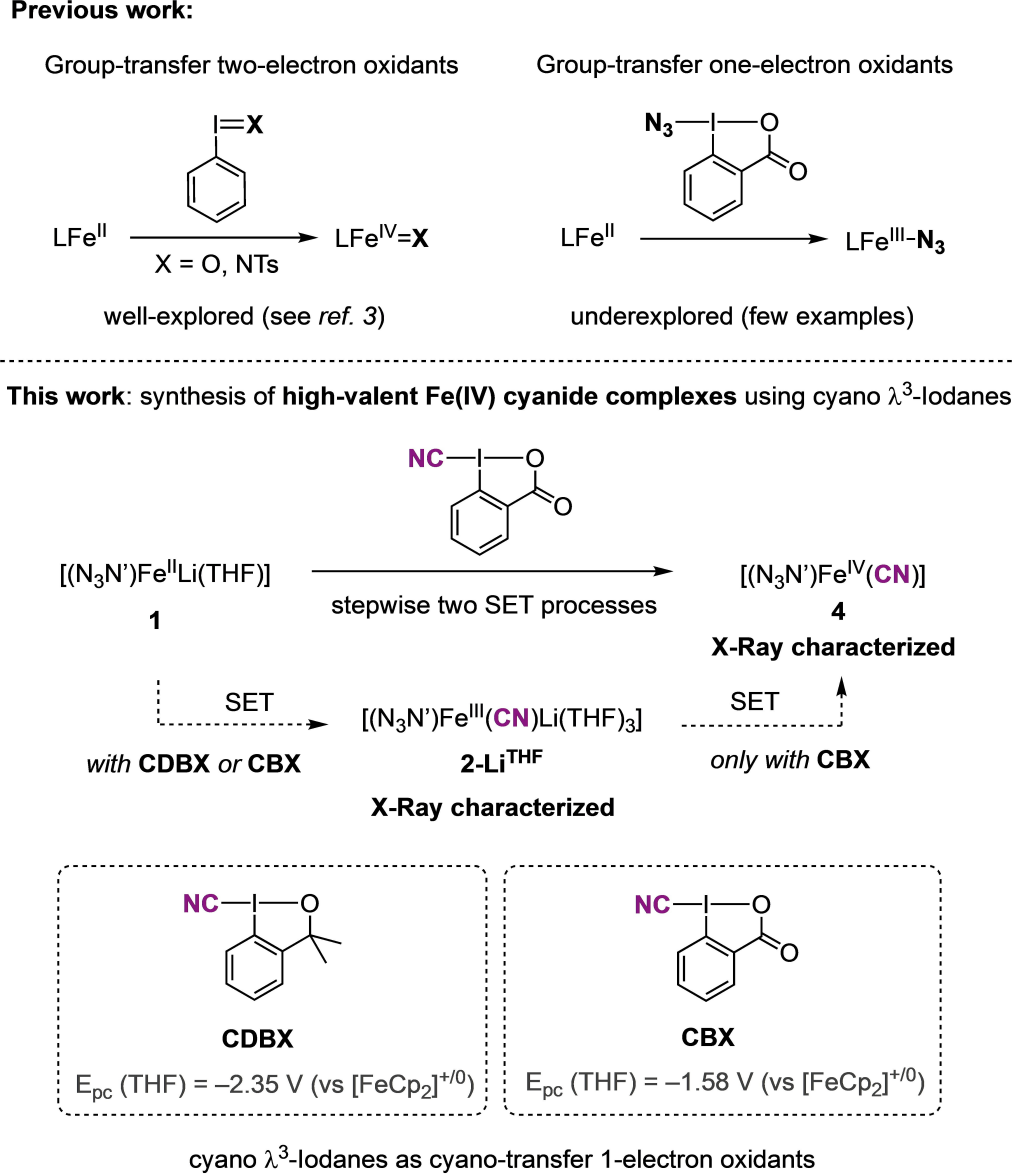
Reactivity of iron complexes with hypervalent iodine reagents.

In depth mechanistic understanding of the activation of iodine(III) reagents with iron complexes has primarily focused on the traditional group‐transfer 2‐electron oxidants, such as iodosylbenzene (PhIO) and imidoiodanes (i.e. PhINTs) (Figure 1).^[3]^ Indeed, the reaction of iron coordination complexes with these λ^3^‐iodanes has enabled the characterization and/or isolation of high‐valent Fe^*n*^‐oxo and ‐imido complexes (*n*=4, 5 or 6), which are relevant intermediates in a myriad of biological and synthetic group‐transfer processes.^[4]^ However, investigations focused on the reactivity of iron complexes with other iodine(III) compounds, such as azide, trifluoromethyl or fluoro λ^3^‐iodanes remain restricted to a few examples despite their potential use in catalysis.^[5]^

Herein, we aim at obtaining mechanistic understanding of iron‐catalyzed group‐transfer reactions with λ^3^‐iodanes, while developing straightforward synthetic strategies towards high‐valent Fe^IV^ complexes. As a starting point, we have focused on the synthesis of Fe^IV^ cyanide complexes that are an unconventional class of iron compounds.^[6]^ One must emphasize that the ability to access Fe^IV^ species without π‐donor ligands such as oxido (O^2−^), imido (RN^2−^) or nitrido (N^3−^), is limited due to their inherent thermal instability.^[7]^ Thus, isolation and structural characterization of highly electrophilic Fe^IV^ cyanide complexes is a very challenging task and information on their structure and preparation is scarce. Gaining insight on how to generate these highly reactive compounds may pave the way towards the development of iron‐catalyzed cyanation reactions proceeding via high‐valent iron intermediates.

We report the unprecedented reactivity of iron(II) and iron(III) complexes with cyano λ^3^‐iodanes, particularly with cyano‐3,3‐dimethyl‐1,2‐benziodoxole (CDBX) and cyanobenziodoxolone (CBX).^[8]^ We show that both CDBX and CBX react as cyano‐transfer one‐electron oxidants. By the appropriate selection of the ligand and the cyano λ^3^‐iodane we have accomplished the straightforward synthesis of Fe^III^ and Fe^IV^ cyanide complexes. In this regard, we selected the triamidoamine ligand tris(N‐*tert*‐butyldimethylsilyl‐2‐amidoethyl)amine [N(CH_2_CH_2_NSiBu^*t*^Me_2_)_3_] (hereafter abbreviated as **N_3_N′**) as a well‐known redox‐innocent ligand that stabilizes metals in high‐oxidation states.^[9]^

## Results and Discussion

**Figure 2:**
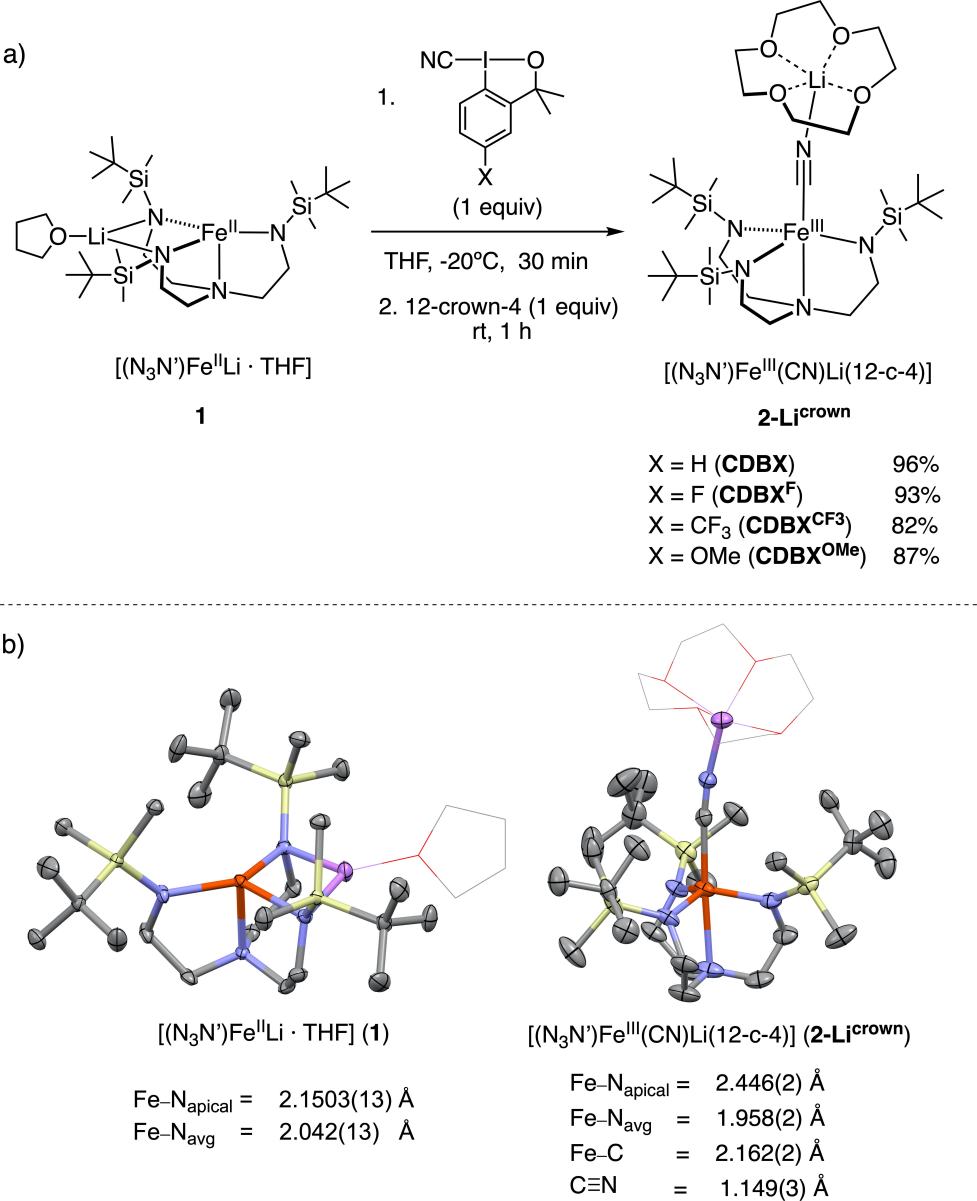
a) Synthesis of **2‐Li^crown^** upon reaction of **1** with CDBX^R^. Isolated yields are given as an average of two runs. b) XRD structures of single crystals of **1** and **2‐Li^crown^**. Thermal ellipsoids drawn at 50 % of probability. Hydrogens are omitted for clarity. THF and 12‐crown‐4 ether molecules are represented as sticks for clarity.

**Figure 3:**
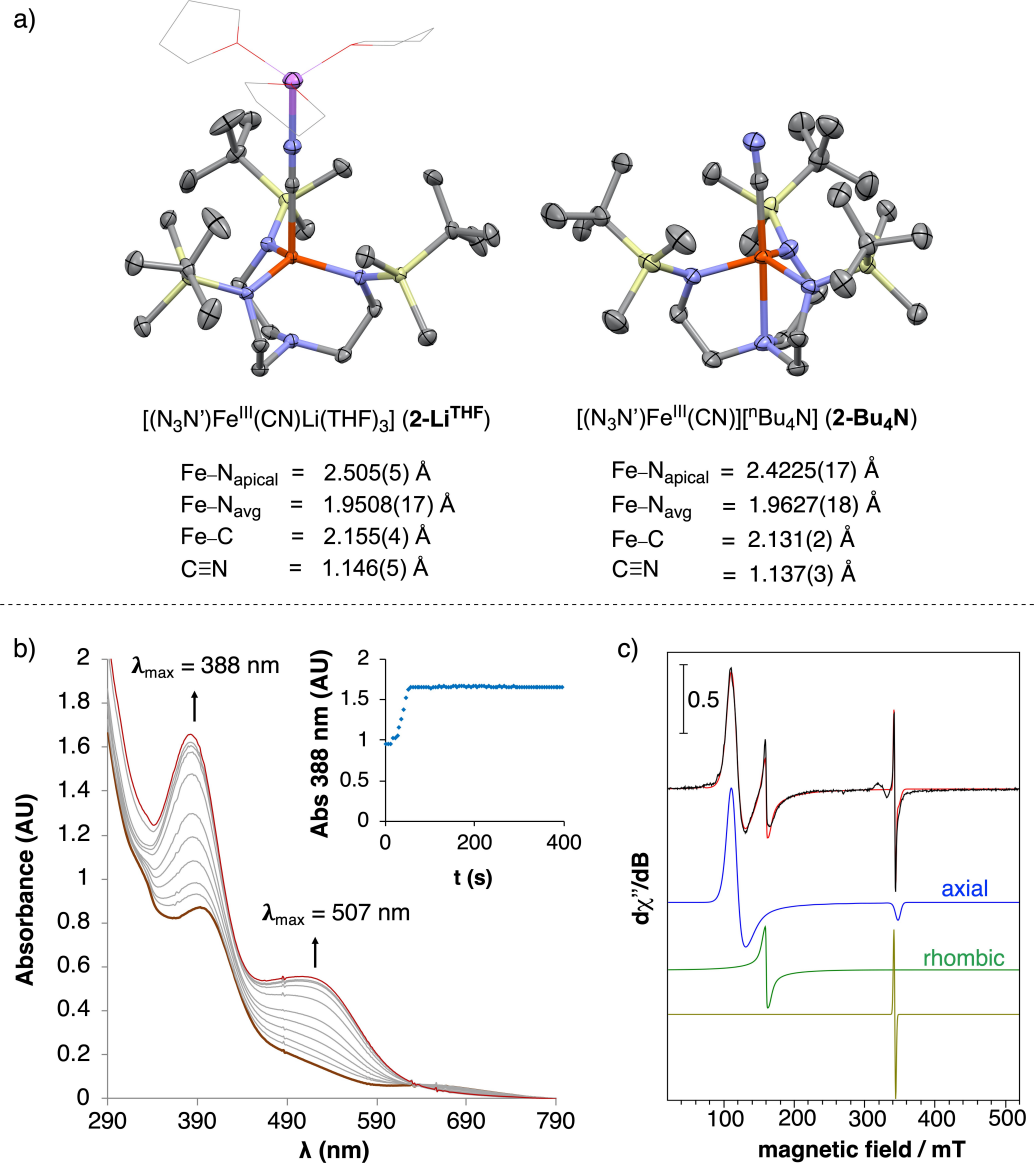
a) XRD of complexes **2‐Li^THF^** and **2‐Bu_4_N**. Thermal ellipsoids drawn at 50 % of probability. Hydrogens and Bu_4_N^+^ cation are omitted for clarity. THF molecules are represented as sticks for clarity. b) UV/Vis spectral changes upon oxidation of **1** (1.6 mM) with CDBX (1 equiv) in THF at −20 °C. Inset plot: kinetic trace at λ=388 nm. c) Perpendicular‐mode X‐band EPR spectra at 11 K of a freeze‐quenched reaction of **1** (20 mM) and 1 equiv of CDBX^F^ in THF after 15 min (black line, 9.63 GHz, power 40 μW, modulation frequency 100 kHz, modulation amplitude 0.75 mT); red line, three‐component simulation for an axial (*g*=[5.77, 6.05, 1.98], 81 %), rhombic (*g*_iso_=4.29, 18 %) and radical species (*g*_iso_=2.007, 1 %).

Our studies started with the reaction of the Fe^II^ complex [(N_3_N′)Fe^II^Li(THF)] (**1**)^[10]^ with 1 equivalent of CDBX in THF at −20 °C, which affords the corresponding Fe^III^ cyanide complex [(N_3_N′)Fe^III^(CN)(Li)(THF)_3_] (**2‐Li^THF^**, Figure 2). Attempts to obtain isolated yields of **2‐Li^THF^** were not successful since removal of solvent under vacuum triggers its decomposition. However, sequestration of the Li^+^ by treatment of **2‐Li^THF^** with 1 equivalent of 12‐crown‐4 ether (12‐c‐4) enables the isolation of [(N_3_N′)Fe^III^(CN)(Li)(12‐c‐4)] (**2‐Li^crown^**) in 96 % yield. **2‐Li^crown^** can also be obtained from **1** in 82–93 % yield following the same procedure using several substituted CDBX^R^ (R=F, OMe, CF_3_) derivatives. Suitable crystals for X‐ray diffraction (XRD) analysis of **2‐Li^THF^** were obtained from a saturated THF solution of the reaction crude at −78 °C (Figure 3a).^[11]^ The solid‐state structure of **2‐Li^THF^** showed the coordination of the Li^+^ to the axial cyanide ligand as in complex **2‐Li^crown^**.

The formation of **2‐Li^THF^** was monitored by UV/Vis spectroscopy. Reaction of **1** with CDBX gives rise to the rapid formation of an absorption band at λ=507 nm, together with an isosbestic point at 633 nm (Figure 3b). The EPR analysis of a freeze‐quenched reaction of **1** with CDBX^F^ revealed the formation of an *S*=5/2 axial species along with an *S*=5/2 rhombic species, both consistent with high‐spin Fe^III^ (Figure 3c). The axial:rhombic ratio was 81 : 18 and 74 : 25 from two independent experiments. For comparative purposes, we prepared a sample of Fe^III^ cyanide complex [(N_3_N′)Fe^III^(CN)](Bu_4_N) (**2‐Bu_4_N**), which contains *n*‐Bu_4_N^+^ cation instead of Li^+^ (see Supporting Information). In this case, the EPR spectrum of **2‐Bu_4_N** reveals the presence of a similar axial high‐spin Fe^III^, which corresponds to >99 % of the EPR signal (Figure SI‐4). We hypothesize that both *S*=5/2 axial and rhombic species detected in the crude reaction correspond to Fe^III^ cyanide species and that the rhombic signal may originate from anisotropic interactions to the Fe^III^ center that disturb the three‐fold symmetry. This has been observed previously for other iron complexes featuring N‐based tripodal ligands.^[12]^ However, an in‐depth spectroscopic investigation of the nature of the rhombic species observed in the EPR spectrum shall be addressed in future studies. Structural comparison between **2‐Li^crown^**, **2‐Li^THF^** and **2‐Bu_4_N** indicates that the Li^+^ coordinates strongly to the N atom of the cyanide ligand with concomitant elongation of the C−N_apical_ and Fe−C bonds (Figure 2). Whereas a trigonal bipyramidal (TBP) geometry is found for **2‐Li^crown^** and **2‐Bu_4_N**, the strong lithium interaction enforces tetrahedral coordination geometry in **2‐Li^THF^** (Figure 2). Despite these structural changes, the DFT calculations at S12g/TZ2P level on **2‐Li^THF^** and **2‐Bu_4_N** predict an *S*=5/2 ground state for both complexes in agreement with the EPR spectra.

These findings indicate that CDBX reacts with **1** as a cyano‐transfer one‐electron oxidant to form an Fe^III^−CN complex, despite the strong σ‐donating properties of the **N_3_N′** ligand. This contrasts with the more commonly explored hypervalent iodine compounds such as 2‐iodoxybenzoic acid (IBX), iodosylbenzene (PhIO) and iminoiodanes (i.e. PhINTs), which react with Fe^II^ as oxygen‐ and tosylimido‐transfer 2‐electron oxidants to form Fe^IV^‐oxo and Fe^IV^‐imido complexes, respectively.^[3]^ Only recently has been reported the reaction of an Fe^II^ complex with azidobenziodoxolone (ABX) via SET and homolytic cleavage of the I−N_3_ bond in ABX.^[5a]^ For CDBX, as opposed to ABX, the I−CN bond is expected to cleave heterolytically upon SET. It is remarkable that despite the higher I−CN bond dissociation energy compared to I−N_3_ bond in ABX, these reactions have a similar outcome.^[13]^ The 1‐electron process reported for iron herein differs also with the 2‐electron oxidative processes reported for the reaction of a variety of λ^3^‐iodanes with 4d and 5d transition metal complexes (Pd, Rh, Ir, Au),^[14]^ as well as with late 3d transition metals, such as Cu and Ni.^[15]^

**Figure 4:**
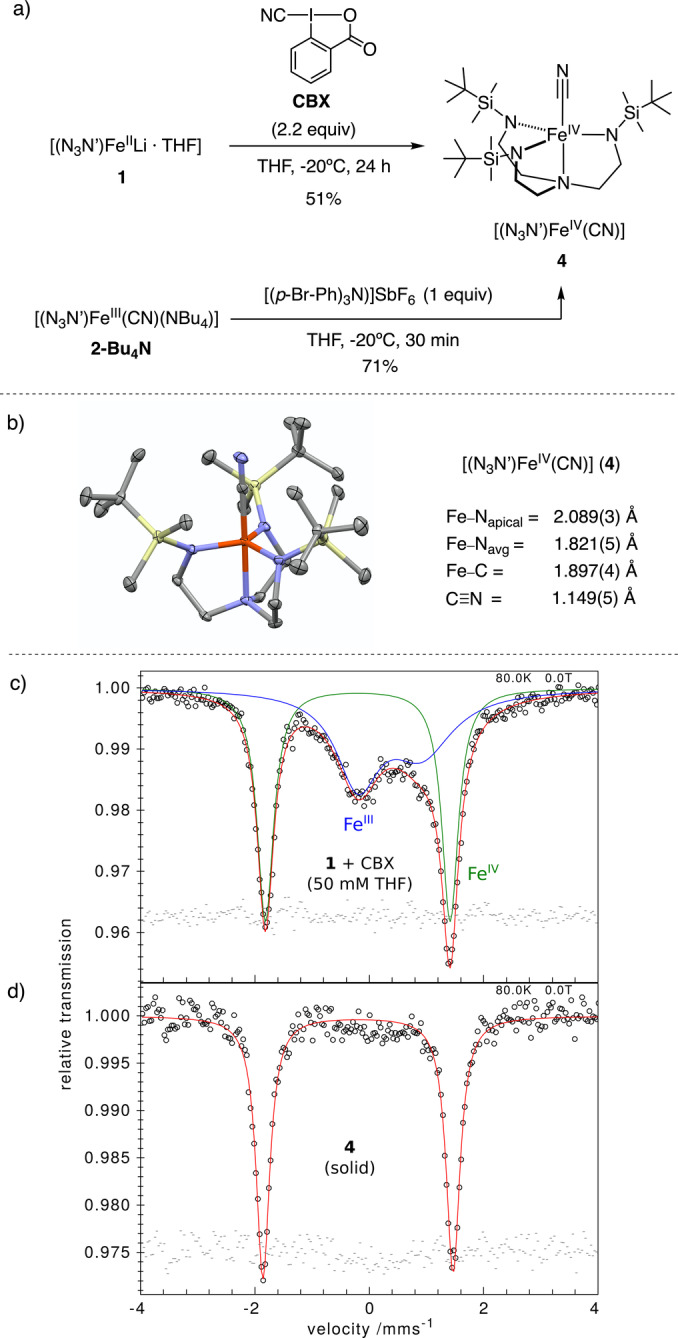
a) Synthetic routes towards the Fe^IV^ cyanide complex **4**. b) Solid‐state structure of **4** obtained by XRD analysis. Thermal ellipsoids drawn at 50 % of probability. Hydrogens are omitted for clarity. c) Zero‐field Mössbauer spectra at 80 K of frozen solution of reaction of **1** and 2.2 equiv of CBX (*δ*_1_=−0.20 mm s^−1^, |Δ*E*_Q_|_1_=3.23 mm s^−1^, 51 %; *δ*_2_=0.36 mm s^−1^, |Δ*E*_Q_|_2_=1.12 mm s^−1^, 49 %). d) Zero‐field Mössbauer spectra at 80 K of **4** obtained from oxidation of **2‐Bu_4_N** with [(4‐BrC_6_H_4_)_3_N]SbCl_6_ (*δ*=−0.20 mm s^−1^, |Δ*E*_Q_|=3.32 mm s^−1^). The red lines are fits with Lorentzian doublets whose complete set of parameters is given in Table SI‐1.

The **N_3_N′** ligand stabilizes the electrophilic Fe^IV^ cyanide complex, [(N_3_N′)Fe^IV^(CN)] (**4**) as reported by the Schrock group.^[6a]^ We hypothesized that upon selection of the appropriate cyano λ^3^‐iodane, we could achieve the synthesis of **4** directly via group transfer and consecutive SET steps. For comparative purposes, we prepared complex **4** by 1 electron oxidation of **2‐Bu_4_N** with 1 equiv of [(4‐BrC_6_H_4_)_3_N]SbCl_6_ as an outer‐sphere oxidant (*E*_1/2_=+0.80 V vs. [FeCp_2_]^+/0^ in THF at rt, Figure SI‐37, Figure 4a). The crystal structure of **4** is shown in Figure 4b along with selected bond lengths. To our knowledge, this is the only solid‐state structure of a monocyanide Fe^IV^ complex.^[16]^ Complex **4** exhibits an almost ideal TBP geometry and it contains a remarkably short Fe^IV^−CN bond length (1.897(4) Å) when compared to the analogous Fe^III^ cyanide complexes **2‐Li^THF^** (2.155(4) Å), **2‐Li^crown^** (2.162(2) Å) and **2‐Bu_4_N** (2.131(2) Å). In addition, the equatorial Fe−N_avg_ bond lengths correlate with the oxidation state of the metal center: 2.042(13) Å, 1.9627(18) Å, and 1.821(5) Å for Fe^II^−N_avg_ (**1**), Fe^III^−N_avg_ (**2‐Bu_4_N**) and Fe^IV^−N_avg_ (**4**), respectively. The isomer shift δ=−0.20 mm s^−1^ (calc.: −0.26 mm s^−1^), the large quadrupole splitting |Δ*E*_Q_|=3.32 mm s^−1^ (calc.: −2.75 mm s^−1^) of the zero field Mössbauer spectrum at 80 K (Figure 4d), the diamagnetic ^1^H and ^13^C NMR spectra of **4** indicate a low‐spin ground state (*S*=0). The calculated ground state supports *e*(d_xz_,_yz_)^4^ *e*(d_xy_,${}_{x{}^{2}\text{-}y{}^{2}}$ )^0^ *a*(d${}_{z{}^{2}}$ )^0^ electronic structure, in *C*_3_ point group (Figure SI‐46, Table SI‐10), which is 0.73 eV more stable than the lowest lying *S*=2 state. The simulation of the Mössbauer spectrum at 1.7 K under 7.0 T revealed *δ*=−0.17 mm s^−1^, *η*=0 and Δ*E*_Q_<0 (Figure SI‐7), consistent with the three‐fold symmetry axis passing by the ^57^Fe nucleus and the electric field gradient tensor elongated axially. The higher isomer shift at lower temperature is expected as a consequence of the second‐order Doppler effect. The isomer shift, symmetry of the electric field gradient and sign of the quadrupole splitting resemble those obtained for a related metastable TBP Fe^IV^ cyanide complex reported by Que and co‐workers.^[6d]^ However, the |Δ*E*_Q_| of **4** is much lower than the value reported for the later (4.45 mm s^−1^) and it may be attributed to the stronger metal‐ligand covalency imparted by the trianionic **N_3_N′** ligand in **4** as opposed to their neutral tetramethylguanidyl‐based ligand.

Remarkably, we have also developed a straightforward synthesis of the Fe^IV^ cyanide **4** from **1** upon activation of cyanobenziodoxolone (CBX), which is a stronger oxidant than CDBX. Reaction of **1** with 2.2 equiv of CBX in THF at −20 °C gives the immediate formation of a deep red solution that overnight turned into the distinctive purple colour of product **4** (Figure 4a). This suggests that the formal oxidation from Fe^II^ to Fe^IV^ with CBX could proceed through two consecutive SET processes via Fe^III^ intermediate species, rather than a concerted two electron transfer oxidation.^[17]^ The Mössbauer spectrum of a freeze‐trapped solution after reaction of **1** with 2.2 equiv of CBX in THF at −20 °C for 24 h shows one component matching a low‐spin Fe^IV^ species (51 %) and another asymmetric doublet consistent with a high‐spin ferric species (49 %) (Figure 4c). The asymmetry is due to intermediate relaxation of the *S*=5/2 Fe^III^ (see Table SI‐1). In addition, the EPR spectrum of a solution crude of the reaction of **1** with 2 equiv of CBX^F^ frozen after 15 min (Figure SI‐3) shows the same axial and rhombic *S*=5/2 species consistent with high‐spin Fe^III^ that are observed in the EPR spectrum when using CDBX^F^ reagent (Figure 3b). This supports the intermediacy of Fe^III^ cyanide species en route to Fe^IV^ cyanide **4**. We propose that reaction of Fe^II^ complex **1** with 1 equiv of CBX forms the Fe^III^ cyanide **2‐Li^THF^** intermediate proceeding via SET and group transfer processes. A second SET process between CBX and **2‐Li^THF^** gives **4**, albeit this is slower than the first SET event. Importantly, the second SET does not occur when the oxidant is CDBX, since the reaction of **1** and excess of CDBX (from 2 to 10 equiv) affords **2‐Li^THF^** as discussed above.

**Scheme 1:**
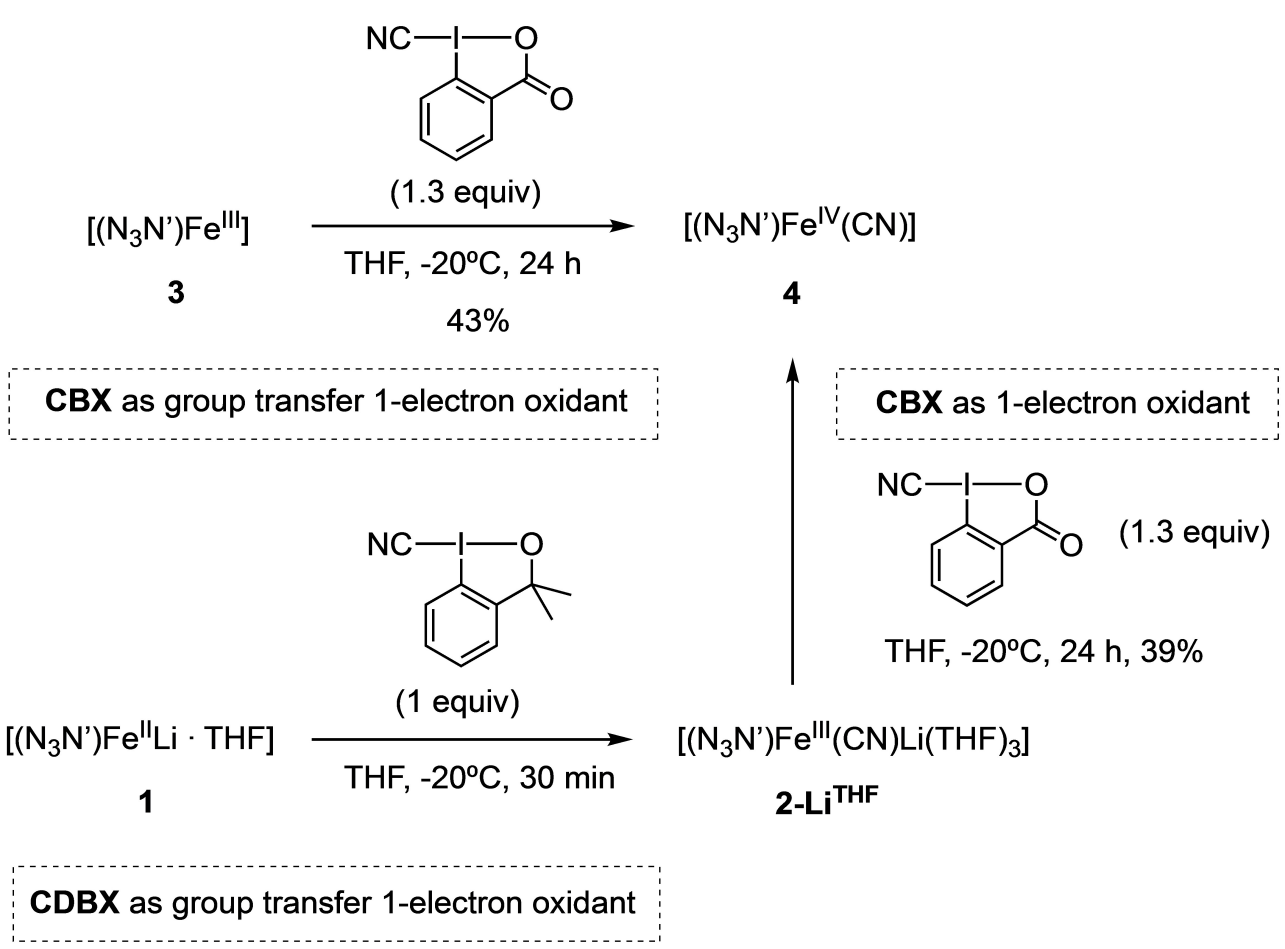
Reactivity of CBX with Fe^III^ complexes to form the Fe^IV^ complex **4**.

We further investigated the competence of CBX as cyano‐transfer and one‐electron oxidant to prepare Fe^IV^ cyanide **4** from the neutral Fe^III^ complex [(N_3_N′)Fe^III^] (**3**) (see Supporting Information).^[9]^ Reaction of **3** with 1.3 equiv of CBX in THF at −20 °C for 24 h and freeze‐trapped Mössbauer spectroscopy indicated the formation of **4** in 43 % yield (Scheme 1 and Figure SI‐10). Another synthetic strategy towards **4**, consisted on forming in situ Fe^III^ cyanide complex **2‐Li^THF^** by reaction of **1** with 1.3 equiv of CDBX, followed by addition of 1.3 equiv of CBX, which gave **4** in 39 % as determined by Mössbauer spectroscopy from the frozen crude solution (Figure SI‐11). This result shows that CBX can also react solely as one electron transfer reagent to obtain Fe^IV^.

**Figure 5:**
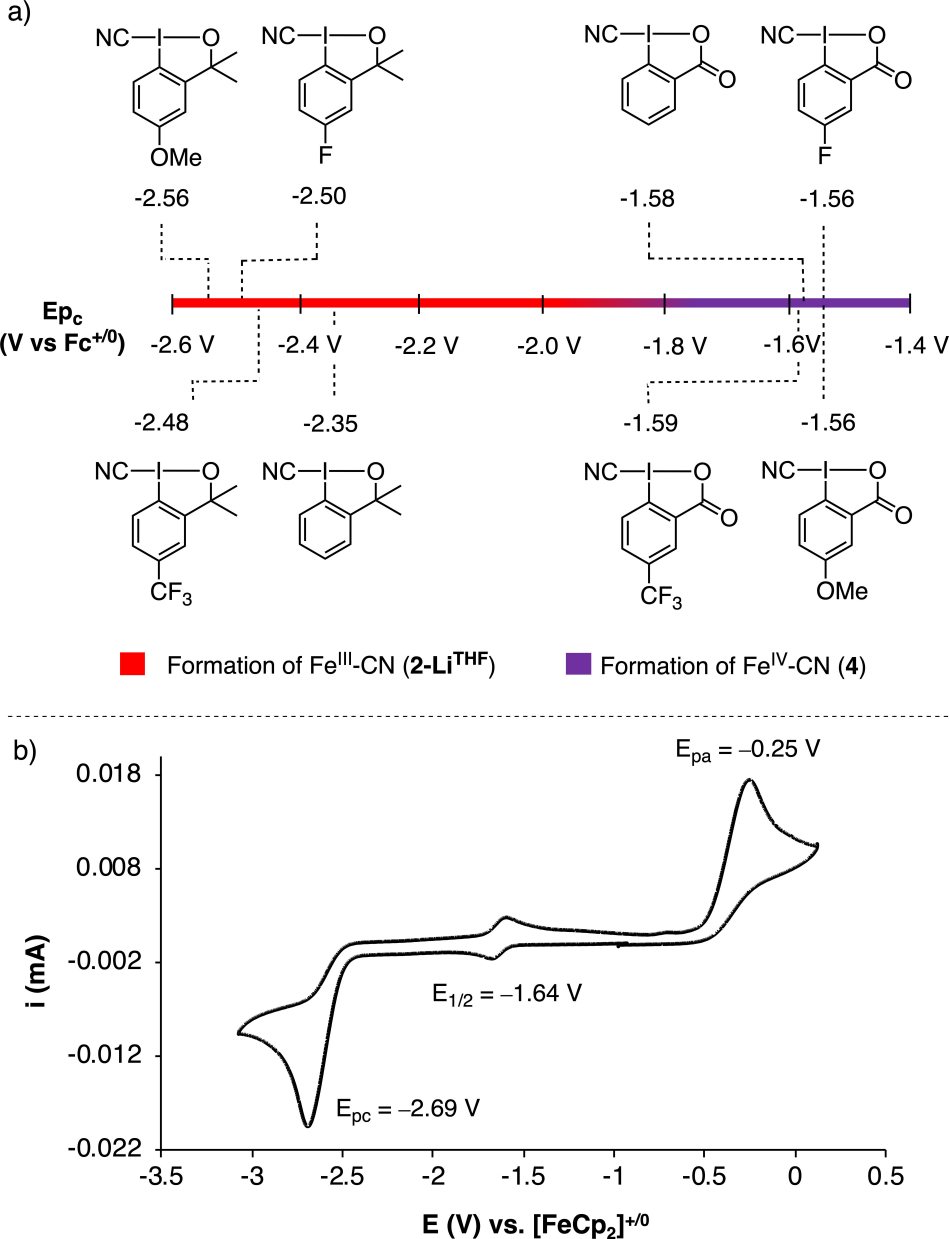
a) Measured reduction potentials (*E*_pc_) of the cyano λ^3^‐iodanes in THF vs. [FeCp_2_]^+/0^ at scan rate of 50 mV s^−1^. b) CV of **2‐Bu_4_N** in THF (3 mM) at −20 °C at scan rate of 50 mV s^−1^. A three‐electrode electrochemical cell has been used: glassy carbon as working electrode, platinum wire as auxiliary electrode, Ag/AgNO_3_ (0.01 M) as reference electrode and *n*Bu_4_NPF_6_ as electrolyte (0.1 M) and ferrocene as internal reference.

The redox potential of the cyano λ^3^‐iodanes is a key parameter for their reactivity. We measured the reduction potential (*E*_pc_) for several *p*‐substituted derivatives CBX^R^ and CDBX^R^ (R=H, OMe, CF_3_ and F) by means of cyclic voltammetry (CV, Figure 5). An irreversible reduction peak is observed at values lower than −2.35 V vs. [FeCp_2_]^+/0^ for CDBX^R^, whereas the CV measured for all CBX^R^ show an irreversible peak above −1.59 V vs [FeCp_2_]^+/0^. Thus, CBX^R^ are more powerful oxidants than CDBX^R^. This is in agreement with the observation that oxidation of **1** to **4** is achieved with CBX^R^, whereas for CDBX^R^ reagents only **2‐Li^THF^** is obtained. In addition, the CV of **2‐Bu_4_N** in THF at −20 °C shows a negative irreversible redox process at −2.69 V vs [FeCp_2_]^+/0^, which is tentatively assigned to the Fe^III^ reduction to Fe^II^ and it is associated to the irreversible oxidation peak found at −0.25 V vs [FeCp_2_]^+/0^. The reversible redox event at *E*_1/2_=−1.64 V vs [FeCp_2_]^+/0^ could be associated to the Fe^III^/Fe^IV^ redox couple.^[18]^ The DFT computed Fe^III^/Fe^IV^ redox potential at S12g/TZ2P is −1.63 V vs [FeCp_2_]^+/0^, whereas the Fe^II^/Fe^III^ redox potential is estimated at −3.27 V vs [FeCp_2_]^+/0^ (see Table SI‐13).^[19]^ These values are more negative than the ones reported for Fe^II/III^ and Fe^III/IV^ in other nitrogen‐based anionic tripodal ligands.[^12a^, ^12b^, ^20^] The exceptionally negative value observed for the Fe^III^/Fe^IV^ redox couple for **2‐Bu_4_N** is likely a result of having the metal center coordinated to three anionic amido and one cyanide ligands.

**Scheme 2:**
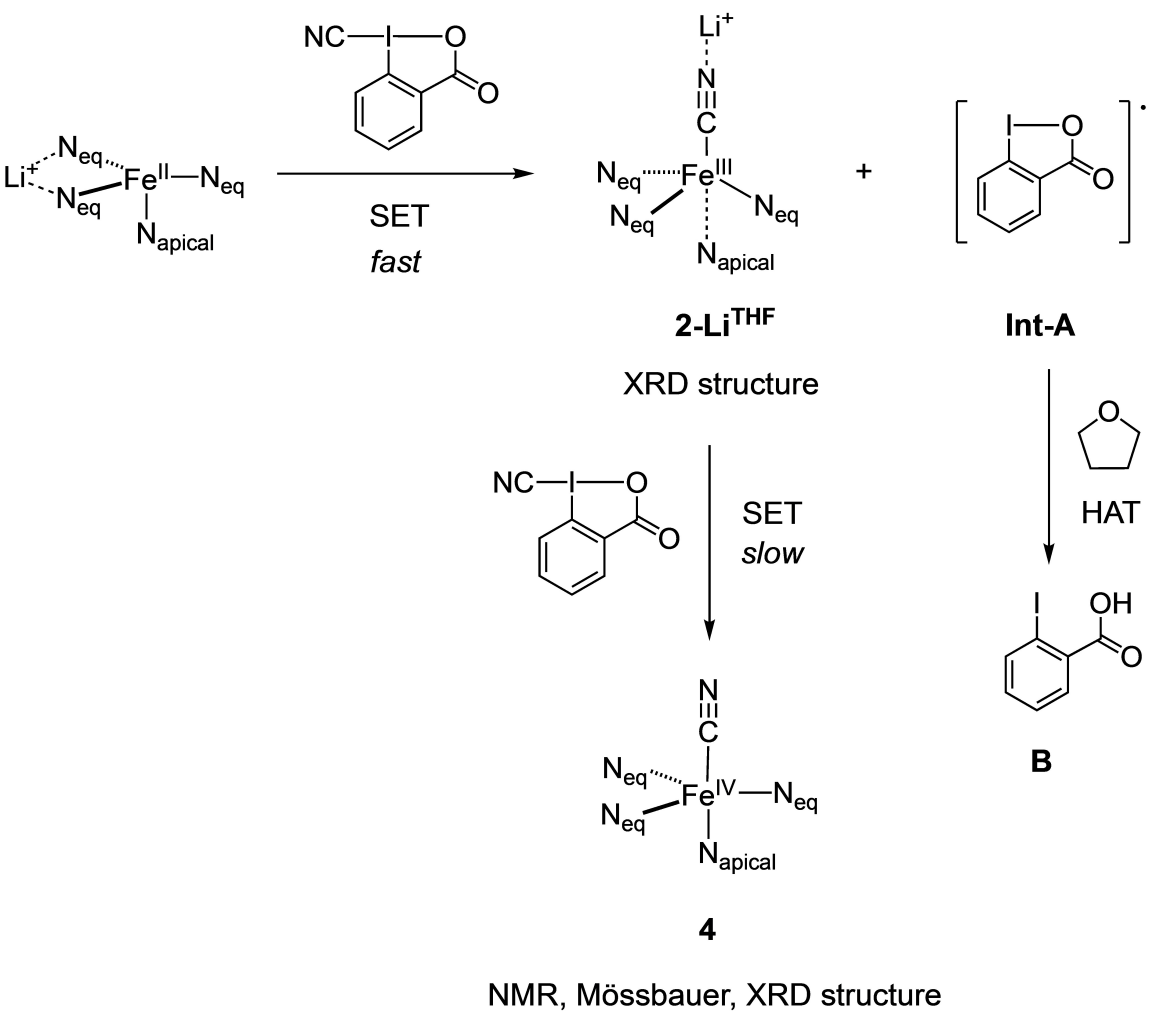
Mechanistic proposal.

Based on the data presented herein, we propose the following reaction pathway for the activation of CBX with Fe^II^ complex (Scheme 2): **1** reacts with this cyano λ^3^‐iodane through group transfer and SET step to generate a highly reactive λ^2^‐iodanyl radical **Int‐A** and iron Fe^III^ cyanide species as revealed by the EPR spectra of a freeze‐quenched reaction of **1** with CBX or CDBX reagents. In this regard, we have obtained suitable crystals for XRD analysis of **2‐Li^THF^** from the crude reaction. The iodanyl radical **Int‐A** can be quenched via HAT with THF to form **B**. Indeed, the choice of THF as solvent was key for the success of the developed reaction.^[21]^ Finally, a second SET reaction between the formed Fe^III^ cyanide species **2‐Li^THF^** and another molecule of CBX occurs at lower reaction rates to form the corresponding Fe^IV^ cyanide complex **4**. We have observed that when the metal complex is coordinatively saturated, the electron transfer and group transfer processes could be uncoupled when the hypervalent iodine reagent is CBX. This may be in part due to the stabilization of iodanyl radical intermediate with the carboxylate moiety, which could favor a delayed cleavage of I−CN bond and decouple the cyano transfer from the electron transfer events.

## Conclusion

In summary, we present an in‐depth study of the reactivity of Fe^II^ and Fe^III^ complexes with two different families of cyano λ^3^‐iodanes, CDBX^R^ and CBX^R^. Our results indicate that CDBX^R^ and CBX^R^ behave as cyano‐transfer one‐electron oxidant with an Fe^II^ compound to form the corresponding Fe^III^ cyanide complex. The selection of the more oxidant CBX^R^ enables the synthesis of unusual high‐valent Fe^IV^ cyanide complex, which can be obtained by reaction of either Fe^II^ and Fe^III^ with CBX. Our experimental findings reveal that the formal two‐electron oxidation from Fe^II^ to Fe^IV^ cyanide with CBX proceeds via two sequential single electron transfer reactions. A thorough understanding of the reactivity of iron complexes with iodine(III) compounds may guide the design of new synthetic methods to prepare elusive high‐valent iron complexes and to develop novel iron‐catalyzed group‐transfer reactions.

## Conflict of interest

The authors declare no conflict of interest.

1

## Supporting information

As a service to our authors and readers, this journal provides supporting information supplied by the authors. Such materials are peer reviewed and may be re‐organized for online delivery, but are not copy‐edited or typeset. Technical support issues arising from supporting information (other than missing files) should be addressed to the authors.

Supporting Information

## Data Availability

The data that support the findings of this study are available in the supplementary material of this article.
