# Supplementary material for: Synthesis of FeIII and FeIV Cyanide Complexes Using Hypervalent Iodine Reagents as Cyano‐Transfer One‐Electron Oxidants
Source: Angew Chem Int Ed Engl. 2022 Mar 29;61(22):e202201699. doi: 10.1002/anie.202201699 (PMC9313551; doi:10.1002/anie.202201699)
Supplement: Supplementary file 1 — Supporting Information [file ANIE-61-0-s001.pdf]

Angewandte Chemie
Eine Zeitschrift der Gesellschaft Deutscher Chemiker

Supporting Information

Synthesis of Fe^{III} and Fe^{IV} Cyanide Complexes Using Hypervalent Iodine Reagents as Cyano-Transfer One-Electron Oxidants

*C. Souilah, S. A. V. Jannuzzi, D. Demirbas, S. Ivlev, M. Swart, S. DeBeer, A. Casitas**

Table of contents

1. General Procedures 3

1.1. General 3

1.2. Synthesis and characterization of ligand $Li_3[N_3N'](THF)_x$ 8

1.3. Synthesis of iron complexes 9

1.3.1. $[(N_3N')Fe^{II}Li(THF)]$ 9

1.3.2. $[N_3N']Fe^{III}$ complex 9

1.3.3. Synthesis of $[(N_3N')Fe^{III}(CN)](^{n}Bu_4N)$ 10

1.3.4. Synthesis of $[(N_3N')Fe^{IV}(CN)]$ 11

1.4. Reactivity of Fe^{II} and Fe^{II} with cyano λ^3-iodanes 12

1.4.1. Synthesis of $[(N_3N')Fe^{III}(CN)Li(12\text{-}c\text{-}4)]$ using CDBX reagent 12

1.4.2. Synthesis of $[(N_3N')Fe^{IV}(CN)]$ using CBX reagent 13

1.4.3. EPR spectroscopic data 16

1.4.4. ^{57}Fe Mössbauer spectroscopic data 18

1.5. Synthesis of cyano Iodine(III) reagents 24

1.5.1. Synthesis of iodine(III) reagents CBX^R (R = OMe, CF_3) 24

1.5.2. Synthesis of iodine(III) reagents $CDBX^R$ (R = F, OMe, CF_3) 27

1.6. UV/Vis characterization of the iron complexes 34

1.7. Cyclic voltammetry experiments 37

1.7.1. Hypervalent iodine reagents 37

1.7.2. Iron complexes 46

2. X-Ray Data Tables 47

3. DFT Calculations 57

4. Characterization data 67

5. References 103

1. General Procedures

1.1. General

Unless otherwise stated, all reactions were performed in oven-dried (90 °C) glassware, using anhydrous solvents, inert atmosphere (Ar or N_2), either applying standard Schlenk techniques or inside an anaerobic glovebox (GS-MEGA X) with concentrations of O_2 and H_2O < 0.1 ppm.

The following solvents were purified by distillation over the indicated drying agents and transferred under inert atmosphere: tetrahydrofuran and diethyl ether (Na/benzophenone), pentane and toluene (Solvona®), dichloromethane, acetonitrile and fluorobenzene (CaH_2), methanol (Mg and stored with 3 Å M.S.). After distillation, solvents were deoxygenated by thoroughly sparkling with N_2 gas at 0°C for 1h and stored with molecular sieves (MS).

C_6D_6 was degassed by bubbling an argon stream at 0° C for 30 min and stored with activated 3 Å MS. $CDCl_3$ was distilled over CaH_2 and stored with activated 3 Å MS.

All commercially available reagents were used without purification, unless otherwise indicated. Commercial Tris(2-aminoethyl)amine (tren) was dried over CaH_2 and distilled prior to use.

NMR Spectroscopy

NMR spectra were recorded on a Bruker AV III 250 MHz, AV II 300 MHz, AV III 500 MHz and AV III HD 250 MHz spectrometer at room temperature. Chemical shifts (δ) are reported in parts per million (ppm) relative to the residual solvent signals as reference. Multiplets are designated by the following abbreviations: s for singlet, d for doublet, t for triplet, q for quartet, quint for quintet, m for complex pattern (multiplet); the abbreviation br indicates a broad signal. ^{13}C NMR and ^{19}F-NMR spectra were recorded in {^1H}-decoupled mode.

EPR Spectroscopy

Continuous wave (CW) X-band EPR spectra were collected on a Bruker E500 ELEXSYS spectrometer system equipped with an ER4116DM dual-mode cavity and an Oxford Instruments ESR 900 continuous-flow liquid helium cryostat interfaced with an ITC Mercury temperature controller (3.8-300 K range). The microwave unit was a high-

sensitivity ER049X Bruker superX bridge with integrated microwave frequency counter. A magnetic field controller ER083CS was calibrated externally using a ER035M Bruker NMR field probe. The spectra were analyzed, simulated and fitted using *eview* and *esim* programs written by Dr. Eckhard Bill and EasySpin.[1] The ratios between the simulation components were calculated by the numerically-integrated area of the simulation corrected by the respective Aasa-Vängård factors.[2]

Mössbauer Spectroscopy

The Mössbauer spectra were collected on a spectrometer with conventional alternating constant acceleration of the γ-ray source. The sample temperature was kept constant using an Oxford Instrument Variox for zero-field measurements or a Cryogen-Free Magnet (CFM) with integrated variable temperature insert (VTI) for applied-field measurements up to 7 T, perpendicular to the γ beam. The latter is a split-pair super-conducting magnet system, where the temperature can vary from 1.5 to 80 K. The ^{57}Co source in Rh matrix (1.8 GBq) rests at room temperature in the gap of the magnet system at a zero-field position, by using a re-entrant bore. Isomer shifts are quoted versus α-iron at 300 K. An error of ± 0.02 mm s^{-1} is expected as result of the convolution between the uncertainty associated with the calibration and the fitting procedure of the sample spectrum. The minimum experimental line width measured as the full width at half-maximum was 0.24 mm/s. Zero-field and applied-field Mössbauer spectra were simulated and fitted with *MF2* and *MX* programs written by Dr. Eckhard Bill using the usual nuclear Hamiltonian.[3]

IR Spectroscopy

IR measurements were obtained as solid or thin films formed by evaporation inside a glovebox under argon atmosphere. IR spectra were recorded on a Bruker Alpha FT-IR with a diamond ATR module with OPUS software. The absorption bands are given in wave numbers (cm^{-1}), intensities are reported as follows: s = strong, m = medium, w = weak, br = broad band.

UV/Visible Spectroscopy

UV-Visible absorption spectroscopy was performed with a Cary 8454 UV-Vis spectrophotometer from Agilent with ChemStation software. Low temperature control was achieved using a cryostat from Unisoku Scientific Instruments. Quartz cells with cell paths of 1, 0.5 and 0.2 cm were used.

MS spectrometry

HR-ESI and HR-FDI mass spectra were acquired with a LTQ-FT (*Thermo Fischer Scientific*) mass spectrometer and AccuTOF-GCv (JEOL) instrument, respectively. The resolution was set to 100.000.

Electrochemistry

Electrochemical measurements were carried out with a SP-50 potentiostat from BioLogic Science Instruments with EC-Lab® software. Cyclic voltammetry measurements were performed under N_2 atmosphere in a one compartment cell. A glassy carbon electrode (ϕ = 3mm) was used as the working electrode, platinum wire was used as the auxiliary electrode and $Ag/AgNO_3$ (0.01 M) electrode was used as reference electrode for measurements in MeCN, whereas Ag pseudo-reference electrode was used for measurements in THF. 99.99% tetrabutylammonium hexafluorophosphate was used as electrolyte (0.1M in solution). All potentials are given using the ferrocene couple ($Fc^{[1+/0]}$) as an internal reference.

X-Ray Crystal Structure Determination

A suitable crystal of complexes [(N_3N')FeLi(THF)] (**1**), [(N_3N')Fe^{III}(CN)(Li)(12-c-4] (**2-Li^{crown}**), [(N_3N')Fe^{III}(CN)(Li)$(THF)_3$] (**2-Li^{THF}**), [(N_3N')Fe] (**3**) and [(N_3N')Fe(CN)] (**4**) was selected under inert oil and mounted using a MiTeGen loop. Intensity data of the crystal were recorded by Bruker D8 Quest diffractometer (Bruker AXS) with a microfocus source and a CMOS PHOTON 100 detector. Data was collected at 100 K using Mo Kα radiation. The diffraction data were reduced using the APEX 3 software.[4] Multi-scan and numerical absorption corrections were applied using the SADBS program.[5] The structure was solved using dual-space methods (SHELXT-2014/5) and refined against F^2 using the SHELXL-2018/3 and the ShelXle software.[6] All non-hydrogen atoms were refined anisotropically. The hydrogen atoms were placed at geometrically calculated positions and refined using the "riding model" approach with isotropic displacement parameters 1.2 times (1.5 times for terminal methyl groups) of that of the preceding carbon atom.

Note on refinement of [(N_3N')FeLi(THF)] (**1**): One THF group was found to be disordered and was refined accordingly. One of the main molecules was found to have a disordered $Si(CH_3)_2$-$C(CH_3)_3$ part. The second species takes only 5 % volume fraction, which makes it impossible to refine the whole disordered part without using numerous hard restraints.

The highest positive residual density peak mentioned in the Checkcif report belongs to the disordered Si atom.

Note on refinement of [(N_3N')Fe^{III}(CN)(Li)(THF)$_3$] (**2-Li^{THF}**): The THF groups were found to be disordered between two positions and were refined accordingly using the DSR plugin[7] implemented in ShelXle.

Note on refinement of [(N_3N')Fe] (**3**): The whole molecule was found to be disordered in the way so that the disordered species were related almost exactly by a mirror plane perpendicular to the *c* axis. It was attempted to refine the model with disorder in space group $P6_3/m$ or as a twinned crystal in space group $P6_3$. However, the best model was obtained in the space group $P6_3$ by combining disorder and twinning.

Note on refinement of [(N_3N')Fe(CN)] (**4**): It was found during the refinement process that the crystal was pseudo-merohedrally twinned, the corresponding SHELXL twin law 1 0 0 0 0 1 0 1 0 led to a good refinement since the reflection splitting was negligible on most of the frames. An attempt to integrate the dataset as an HKLF5 file produced worse-quality model and less stable refinement.

In case of [(N_3N')Fe^{III}(CN)](Bu_4N) (**2-Bu$_4$N**) a suitable crystal was selected under inert oil and mounted using a MiTeGen loop. Intensity data of the crystal were recorded by STADIVARI diffractometer (Stoe & Cie). The diffractometer was operated with Cu-Kα radiation (1.54186 Å, microfocus source) and equipped with a Dectris PILATUS 300K detector. Evaluation, integration and reduction of the diffraction data was carried out using the X-Area software suite.[8] Multi-scan and numerical absorption corrections were applied with the X-Red32 and LANA modules of the X-Area software suite.[9] The structure was solved using dual-space methods (SHELXT-2014/5) and refined against F^2 (SHELXL-2018/3 using ShelXle interface).[6] All non-hydrogen atoms were refined with anisotropic displacement parameters. The hydrogen atoms were refined using the "riding model" approach with isotropic displacement parameters 1.2 times (for CH_3 groups 1.5 times) of that of the preceding carbon atom. Two $[C_{16}H_{36}N]^+$ cations contained partially disordered terminal carbon atoms which were refined using a corresponding disorder model.

CCDC 2144072, 2144073, 2144074, 2144075, 2144076 and 2144077 contains the supplementary crystallographic data for this paper. These data can be obtained free of

charge from The Cambridge Crystallographic Data Centre via www.ccdc.cam.ac.uk/structures.

Computational Methods

All DFT calculations were performed with the Amsterdam Density Functional (ADF)[10] and QUILD[11] programs. Molecular orbitals were expanded in an uncontracted set of Slater type orbitals (STOs) of triple-ζ quality with double polarization functions (TZ2P).[12] Core electrons were not treated explicitly during the geometry optimizations (frozen core approximation)[10b]. An auxiliary set of s, p, d, f, and g STOs was used to fit the molecular density and to represent the Coulomb and exchange potentials accurately for each SCF cycle.

Geometries of all possible spin states were optimized with the QUILD[11] program using adapted delocalized coordinates until the maximum gradient component was less than 10^{-4} a.u. Energies, gradients and Hessians[13] (for vibrational frequencies) were calculated using S12g,[14] in all cases by including solvation effects through the COSMO[15] dielectric continuum model with appropriate parameters for the solvents.[16] For computing Gibbs free energies, all small frequencies were raised to 100 cm^{-1} in order to compensate for the breakdown of the harmonic oscillator model.[17] Scalar relativistic corrections have been included self-consistently in all calculations by using the zeroth-order regular approximation (ZORA).[18] For all geometry optimizations, single-point and Mössbauer calculations carried out with S12g the Becke[19] grid of VeryGood quality was used; vibrational frequencies with S12g were computed with a Becke grid of Normal quality. Mössbauer parameters were obtained at S12g/TZ2P with COSMO(THF) included self-consistency.[20] All DFT calculations were performed using the unrestricted Kohn-Sham scheme.

All computational data have been uploaded (DOI: (DOI: 10.19061/iochem-bd-4-38) onto the IOCHEM-BD platform (www.iochem-bd.org) to facilitate data exchange and dissemination, according to the FAIR principles of OpenData sharing.[21]

1.2. Synthesis and characterization of ligand $Li_3[N_3N'](THF)_x$

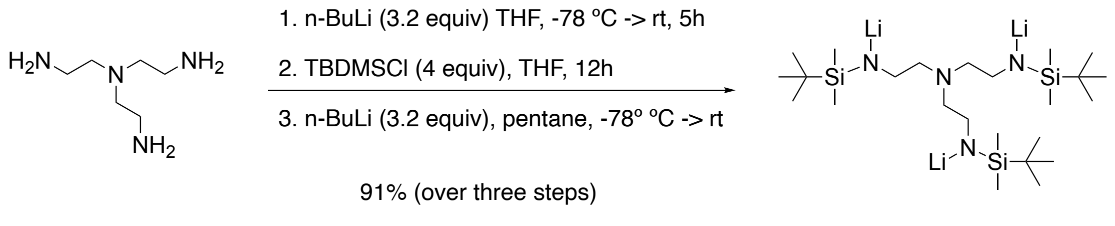

$Li_3[N_3N'](THF)_x$

The synthesis of the triamido amine ligand was prepared following the literature procedure with some modifications.[22] An oven-dried 500 mL Schlenk flask, equipped with a magnetic stirring bar and a connection to the nitrogen line, was charged with tris(2-aminoethyl)amine (10 mL, 66.74 mmol, 1 equiv) and it was dissolved in THF (175 mL). The resulting solution was cooled to –78 °C and *n*-butyllithium (133.6 mL, 213.6 mmol, 3.2 equiv, 1.6 M in hexane) was added dropwise via syringe. After 20 min the yellow mixture was allowed to warm to rt and it was stirred for 5 h. The reaction mixture was then cooled to –78 °C, and *tert*-butyldimethylsilyl chloride was added (40.3 g, 267 mmol, 4 equiv) as a solution in THF (80 mL). The orange mixture that formed was again allowed to warm to rt and it was stirred for 12 h. All volatiles were removed *in vacuo*, and the residue was extracted with pentane (100 mL x 3) and filtered through Celite® under N_2. The pentane extract was cooled to –78 °C and *n*-butyllithium (133.6 mL, 267 mmol, 3.2 equiv, 1.6 M in hexane) was added dropwise via syringe. The resulting yellowish mixture was warmed to rt and stirred for 5 h. Then, the solvent was removed *in vacuo* and addition of THF (16 mL) at –40 °C caused precipitation of a white solid. The solution was removed by canula and the resulting precipitate was dried *in vacuo* to give the title compound as a white-yellowish solid (9.2 g, 91%). The molecules of THF were calculated according to the ^{1}H-NMR spectra for each batch of $Li_3[N_3N'](THF)_x$. **^{1}H-NMR** (300 MHz, CD_3CN): δ 3.65 (THF), 2.79 (t, *J* = 6.4 Hz, 6 H), 2.38 (t, *J* = 6.4 Hz, 6 H), 1.85 (THF), 0.87 (s, 27 H), –0.03 (s, 18 H) ppm; **^{13}C-NMR** (300 MHz, CD_3CN): δ 68.4 (THF) 59.9, 41.4, 26.9, 26.3 (THF), 19.0, –4.6 ppm. **ESI(+)** (m/z): calcd for $C_{24}H_{61}N_4Si_3$ [M + H$^+$] 489.4199; found: 489.4200.

1.3. Synthesis of iron complexes

1.3.1. [(N$_3$N')Fe^{II}Li(THF)]

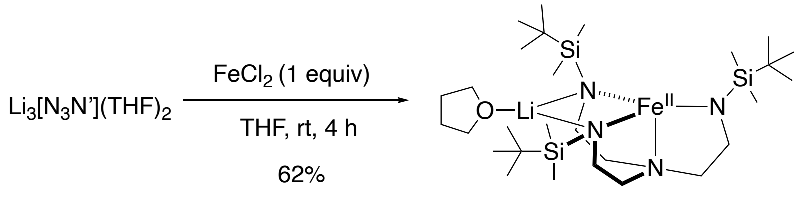

[(N$_3$N')Fe^{II}Li(THF)]

1

In an oven-dried Schlenk flask, equipped with a magnetic stirring bar and a connection to the nitrogen line, was charged with $Li_3[N_3N'](THF)_2$ (14.25 g, 21. 9 mmol, 1 equiv) and 30 mL THF. Then, the resulting solution was cooled at 0°C and $FeCl_2$ (2.5 g, 19.7 mmol, 0.9 equiv) was added as solid in several portions. After the addition of the iron(II) salt, the resulting mixture was allowed to warm to rt and stirred for 4h, forming a dark brown solution. The solvent was removed *in vacuo* and the solid residue was extracted with 30 mL pentane. The extract was filtered through a plug of Celite® under N_2 and the solvent was removed under vacuum (10.9 g, 98%). The brown residue was recrystallized from a saturated solution of pentane at –78 °C to give the title compound as a dark brown crystalline solid (7.3 g, 62%). **^{1}H-NMR** (500 MHz, C_6D_6): δ 209.19, 162.18, 130.68, 108.16, 99.20, 85.12, 45.74, 25.69, 8.18, 3.96, 3.24, 2.42, 1.60, 1.13, 0.87, 0.19, –1.62, –6.66 ppm. **^{57}Fe Mössbauer (80 K, 0T)** δ = 0.78 mm s^{-1}, $|\Delta E_Q|$ = 1.83 mm s^{-1}. **UV/Visible** (THF, 25 °C: λ_{max} 390 nm {1100 M^{-1} cm^{-1}}. **EA** calcd (%) for $C_{24}H_{57}FeLiN_4Si_3$: C 52.53; H 10.47; N 10.21; found: C 52.07, H 9.89, N 8.36.

1.3.2. [N$_3$N']Fe^{III} complex

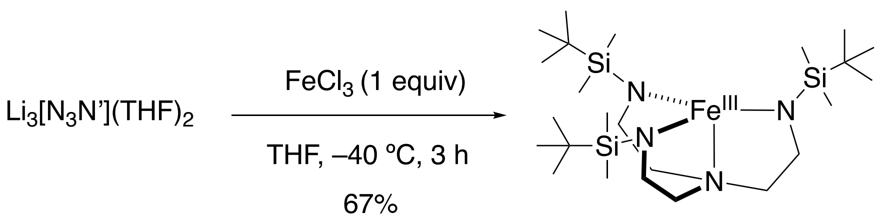

[(N$_3$N')Fe^{III}]

3

The following compound was prepared according to the literature procedure.[23] In an oven-dried Schlenk flask, equipped with a magnetic stirring bar and a connection to the nitrogen line, was charged with anhydrous $FeCl_3$ (0.20 g, 0.1 mmol, 1 equiv) and 70 mL THF. A solution of $Li_3[N_3N'](THF)_2$ (0.6434 g, 0.1 mmol, 1 equiv) in THF (8 mL) was

added via canula over the iron(III) solution at –40 °C. Then, the reaction mixture was allowed to reach rt and after stirring for 3 h, all volatiles were removed *in vacuo*. The brown residue was extracted with 60 mL pentane and the extract was filtered through Celite® under N_2. The solvent was removed under vacuum to give the title compound as a brown solid (0.55 g, 98%). The iron complex was recrystallized from a saturated solution in pentane at –40 °C (0.45 g, 67%). **^{1}H-NMR** (300 MHz, C_6D_6): δ 36.19 (br), 13.78 (br), 3.67, 2.07, 1.73, 1.46, 0.92, 0.29, 0.14, 0.08, 0.02, –1.58, –2.77 ppm. **FD(+)** (m/z): calcd for $C_{24}H_{57}FeN_4Si_3$ [$M^{+\cdot}$] 541.32404; found: 541.32446. **UV/Visible** (THF, 25°C: λ_{max} 398 nm {3122 M^{-1} cm^{-1}}. **EA** calcd (%) for $C_{24}H_{57}FeN_4Si_3$: C 53.20; H 10.60; N 10.34; found: C 50.90, H 10.49, N 9.20.

1.3.3. Synthesis of [$(N_3N')Fe^{III}(CN)$]($^nBu_4N$)

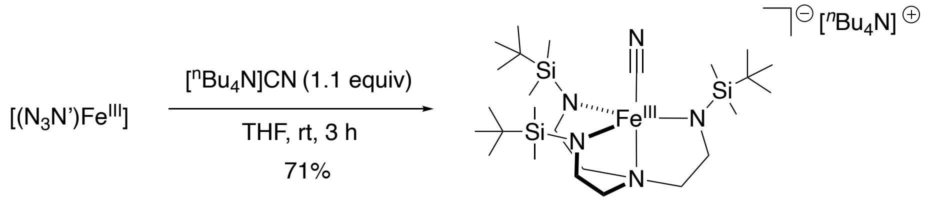

[$(N_3N')Fe^{III}(CN)$]($^nBu_4N$)

2-Bu_4N

Inside a glovebox, tetrabutylammonium cyanide (0.58 g, 2.1 mmol, 1.1 equiv) was added to a solution of [$(N_3N')Fe^{III}$] (**3**) (1 g, 1.8 mmol, 1 equiv) in THF (10 mL), and the solution was stirred at rt for 3h. The colour of the reaction mixture changed immediately from brown to deep red. Then, THF was removed *in vacuo*, and the residue was extracted with the minimum amount of toluene and filtered through a plug of Celite®. The clear red solution was concentrated *in vacuo*, it was cooled at –78 °C and addition of pentane caused precipitation of a red solid. The solvent was removed via cannula and the red solid was rinsed with some pentane and it was dried under vacuum (1.1 g, 71% yield). **^{1}H-NMR** (300 MHz, C_6D_6): δ 10.97 (br), 2.81, 2.36, 2.11, 0.99, 0.09 ppm; **FT-IR (ATR)** υ; cm^{-1}: 2134 (w), 2096 (w), 2014 (s, $\upsilon_{Fe\text{-}CN}$). **LTQ-FT-ESI** (-) (m/z): calcd. for $C_{25}H_{57}FeN_5Si_3$ 567.3271; found: [$M\text{-}^nBu_4N$]$^-$ 567.3290. **UV/Visible** (THF, 25 °C: λ_{max} 380 nm {3685 M^{-1} cm^{-1}}. **EA** calcd (%) for $C_{41}H_{93}FeN_6Si_3$: C 60.77; H 11.57; N 10.37; found: C 60.39, H 11.05, N 9.30.

1.3.4. Synthesis of [(N$_3$N')Fe^{IV}(CN)]

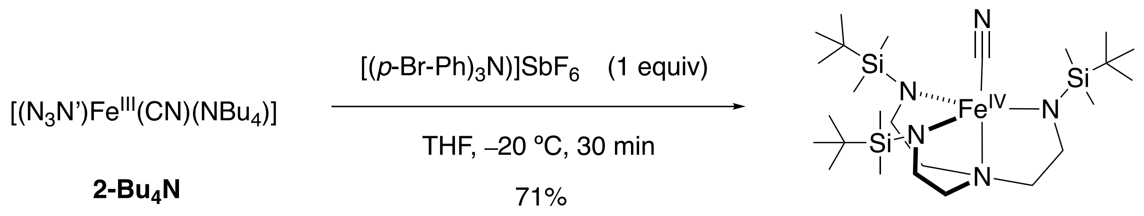

[(N$_3$N')Fe^{IV}(CN)]

4

Inside a glovebox, an oven-dried Schlenk flask, equipped with a magnetic stirring bar, was charged with [(N$_3$N')Fe^{III}Li(CN)(^{n}Bu$_4$N)] (**2-Bu$_4$N**) (0.15 g, 0.18 mmol, 1 equiv). The Schlenk flask was closed, placed outside the glovebox and connected to a N$_2$ inlet. Then, the complex was solubilized in THF (3 mL) at –20 °C and [(*p*-Br-Ph)$_3$N)]SbF$_6$ (0.15 g, 0.18 mmol, 1 equiv) was added as a solid under N$_2$ atmosphere. The colour of the solution immediately changed from red to dark purple. After stirring for 30 min at -20 °C, the solvent was removed *in vacuo*, and the purple residue was extracted with Et$_2$O at –78 °C and filtered through a plug of Celite® at the same temperature until the washings were colourless. The solvent was removed under vacuum and a second filtration was carried out using THF:Pentane (1:1). The solution was placed in the chiller at –78 °C overnight and white-grey precipitate appeared. The remaining purple solution was filtered and the solvent was removed to dryness to give the title compound as a purple solid (75 mg, 71%). **^{1}H-NMR** (500 MHz, CDCl$_3$): δ 3.32 (m, 6 H, CH$_2$), 2.03 (m, 6 H, CH$_2$), 1.06 (s, 27 H,), 0.35 (s, 18 H) ppm; **^{13}C-NMR** (126 MHz, CDCl$_3$): δ 64.5, 57.4, 27.9, 21.3, –0.23 ppm. **FT-IR (ATR)** υ; cm^{-1}: 2114 (υ_{Fe-CN}). **FD(+)** (m/z): calcd for $C_{25}H_{57}FeN_5Si_3$ [M$^{+\cdot}$] 567.32712; found: 567.32644. **^{57}Fe Mössbauer** 80 K, 0 T**:** δ = – 0.20 mm s^{-1}, $|\Delta E_Q|$ = 3.32 mm s^{-1}; 1.7 K, 7 T: δ = – 0.17 mm s^{-1}, ΔE_Q = –3.31 mm s^{-1}, η = 0. **UV/Visible** (THF, 25 °C: λ^1_{max} 395 nm {4518 M^{-1} cm^{-1}}, λ^2_{max} 556 {2550M^{-1} cm^{-1}}.

1.4. Reactivity of Fe^{II} and Fe^{II} with cyano λ^3-iodanes

1.4.1. Synthesis of [(N_3N')Fe^{III}(CN)Li(12-c-4)] using CDBX reagent

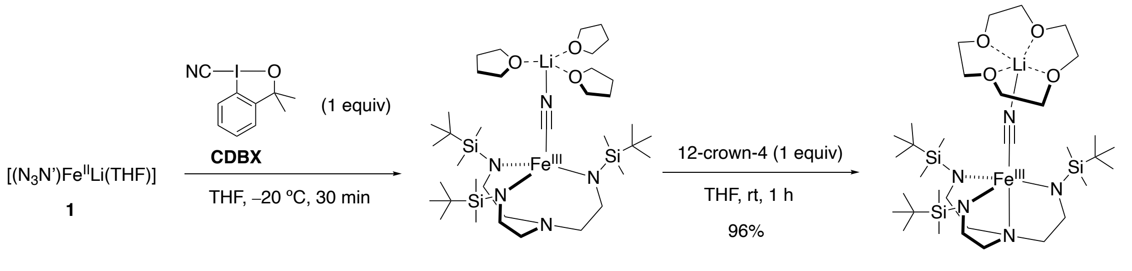

[(N_3N')Fe^{III}(CN)Li(THF)$_3$] **[(N_3N')Fe^{III}(CN)Li(12-c-4)]**

2-Li^{THF} **2-Li^{crown}**

Inside a glovebox, an oven-dried Schlenk flask was charged with 1-cyano-3,3-dimethyl-3-(1H)-1,2-benziodoxole (CDBX) (0.09 g, 0.32 mmol, 1 equiv) and [(N_3N')Fe^{II}Li(THF)] (**1**) (0.2 g, 0.32 mmol, 1 equiv) as solids. The Schlenk flask was closed, placed outside the glovebox and connected to a N_2 inlet. Then, THF (2 mL) was added at –20 °C causing immediately colour change from brown to deep red. After stirring at –20 °C for 30 min, 12-crown-4 (52 µL, 0.32 mmol, 1 equiv) was added over the [(N_3N')Fe^{III}(CN)Li(THF)$_3$] (**2-Li^{THF}**) solution and the mixture was stirred for 1 h at rt. Then, the solvent was removed *in vacuo* and the red solid was extracted with cold Et_2O and filtered through a plug of Celite® at –78 °C under N_2. The solvent was removed under vacuum at –20 °C to give the title compound as a deep red solid (0.23 g, 96%). **^{1}H-NMR** (300 MHz, C_6D_6): 36.19 (br), 13.78 (br), 3.67, 2.07, 1.73, 1.46, 0.92, 0.29, 0.14, 0.08, 0.02, -1.58, -2.77 ppm. **FT-IR (ATR)** υ; cm^{-1}: 2162-2032 ($\upsilon_{Fe\text{-}CN}$). **UV/Visible** (25 °C, nm: λ^1_{max} 385 nm {2101 M^{-1} cm^{-1}}, λ^2_{max} 516 nm {671 M^{-1} cm^{-1}}.

Complex [(N_3N')Fe^{III}(CN)Li(THF)$_3$] (**2-Li^{THF}**) could not be isolated as solid due to the low stability of the compound in solid state when drying it for prolongated times. On the other hand, single crystals suitable for X-Ray diffraction of **2-Li^{THF}** were grown from a saturated solution of THF with some drops of pentane at –78 °C.

Synthesis of **2-Li^{crown}** *using CDBX^R (R = F, CF_3, OMe) reagents:*

Following an analogous procedure than the one described above, 169 mg of **2-Li^{crown}** (93%) were isolated from the reaction of CDBX^F (85.8 mg, 0.24 mmols) and **1** (150 mg, 0.24 mmols); 136 mg of **2-Li^{crown}** (87%) were isolated from the reaction of CDBX^{CF3} (73.7 mg, 0.24 mmols) and **1** (150 mg, 0.24 mmols), 148 mg of **2-Li^{crown}** (82%) were isolated from the reaction of CDBX^{OMe} (76.6 mg, 0.16 mmols) and **1** (150 mg, 0.24 mmols).

1.4.2. Synthesis of [(N_3N')Fe^{IV}(CN)] using CBX reagent

*a) synthesis from [(N_3N')Fe^{II} Li(THF)] (**1**) precursor:*

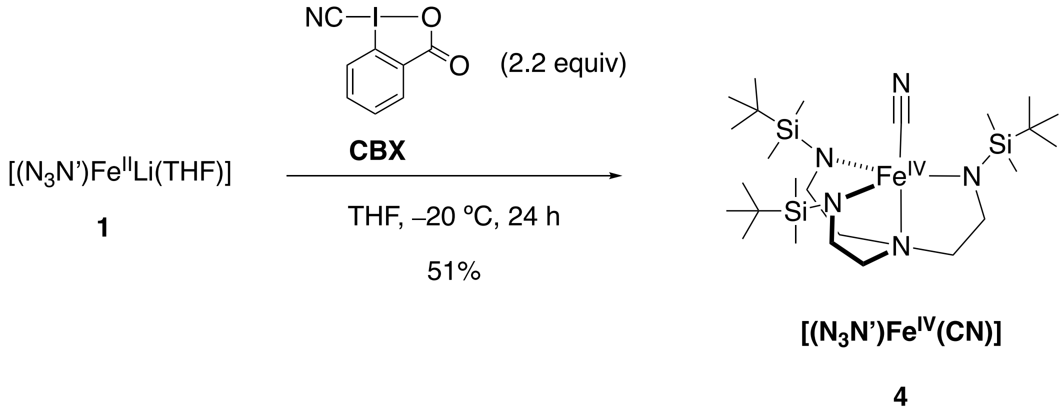

Inside a glovebox, an oven-dried Schlenk flask, equipped with a magnetic stirring bar, was charged with 1-cyano-1,2-benziodoxol-3-(1H)-one (CBX) (0.14 g, 0.44 mmol, 2.2 equiv) and [(N_3N')Fe^{II}Li(THF)] (**1**) (0.12 g, 0.2 mmol, 1 equiv). The Schlenk flask was closed, placed outside the glovebox and connected to a N_2 inlet. Then, THF (4 mL, 50 mM) was added slowly at –20 °C. The colour of the solution immediately changed from brown to red and gradually to a dark solution. After stirring for 24 h an intense purple solution was obtained. Then, 1 mL of stock solution was transferred to a Mössbauer cell, which was previously frozen at liquid nitrogen temperature. The sample was frozen for 15 – 20 min and stored in liquid nitrogen until the measurement of the yield by Mössbauer spectroscopy.

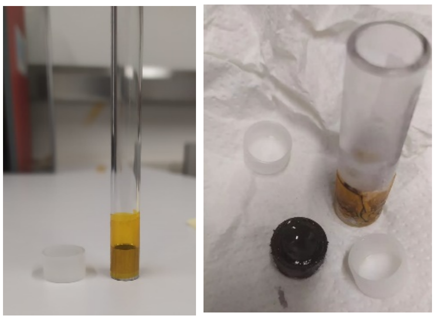

Figure SI-1. Preparation of the frozen solution from the crude reaction for Mössbauer spectroscopy.

*Isolation of **4** prepared by the reaction of **1** with CBX:*

Inside a glovebox, an oven-dried Schlenk flask, equipped with a magnetic stirring bar, was charged with 1-cyano-1,2-benziodoxol-3-(1H)-one (CBX) (0.18 g, 0.64 mmol, 2 equiv) and [(N_3N')Fe^{II}Li(THF)] (**1**) (0.2 g, 0.32 mmol, 1 equiv). The Schlenk flask was

closed, placed outside the glovebox and connected to a N_2 inlet. Then, THF (3 mL) was added slowly at –20 °C. The colour of the solution immediately changed from brown to red and gradually to a dark solution. After stirring for 24h an intense purple solution was obtained. Then, THF was removed *in vacuo* at –20 °C, and the purple residue was extracted with cold Et_2O and filtered through a plug of Celite® at –78 °C under N_2. The solvent was removed under vacuum at –20 °C, then 10-20 mL of pentane was added. Et_2O and pentane were removed to dryness to obtain a powder purple solid (0.17 g). The purity of a solid sample of **4** was 70% as determined by Mössbauer spectroscopy since it was contaminated with Fe^{III} species. Moreover, single crystals suitable for X-Ray diffraction of **4** were grown from a saturated solution of toluene with a layer of pentane by slow diffusion at –40 °C.

*b) Synthesis from [(N_3N')Fe^{III}] (**3**) precursor:*

[(N_3N')Fe^{III}]

3

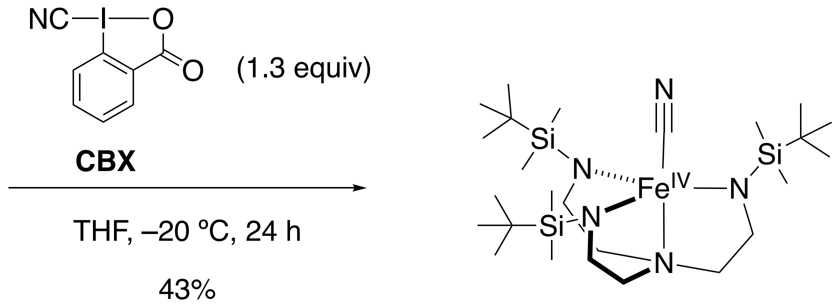

[(N_3N')Fe^{IV}(CN)]

4

Inside a glovebox, an oven-dried Schlenk flask, equipped with a magnetic stirring bar, was charged with 1-cyano-1,2-benziodoxol-3-(1H)-one (CBX) (0.072 g, 0.26 mmol, 1.3 equiv) and [(N_3N')Fe^{III}] (**3**) (0.11 g, 0.2 mmol, 1 equiv). The Schlenk flask was closed, placed outside the glovebox and connected to a N_2 inlet. Then, THF (4 mL, 50 mM) was added slowly at –20 °C. The colour of the solution immediately changed from brown to red and gradually to a dark solution. After stirring for 24 h an intense purple solution was obtained. Then, 1 mL of stock solution was transferred to a Mössbauer cell, which was previously frozen at liquid nitrogen temperature. The sample was frozen for 15 – 20 min and stored in liquid nitrogen until the measurement of the yield by Mössbauer spectroscopy.

*c) Synthesis from [(N$_3$N')Fe^{III}(CN)Li(THF)$_3$] (**2-Li^{THF}**) precursor:*

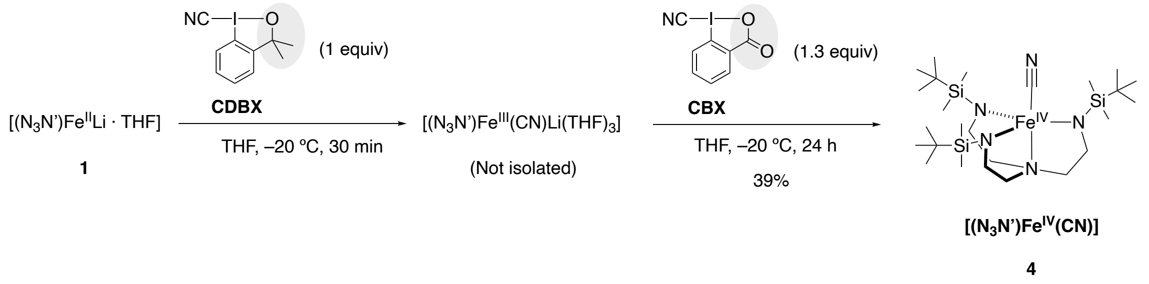

Inside a glovebox, an oven-dried Schlenk flask, equipped with a magnetic stirring bar, was charged with 1-cyano-3,3-dimethyl-1,2-benziodoxole (CDBX) (0.055 g, 0.2 mmol, 1 equiv) and [(N$_3$N')Fe^{II}(Li)(THF)] (**1**) (0.12 g, 0.2 mmol, 1 equiv). The Schlenk flask was closed, placed outside the glovebox and connected to a N_2 inlet. Then, THF (4 mL, 50 mM) was added slowly at –20 °C. The colour of the solution immediately changed from brown to red. After stirring for 30 min, 1-cyano-1,2-benziodoxol-3-(1H)-one (CBX) (0.072 g, 0.26 mmol, 1.3 equiv) was added under inert conditions and the mixture was stirred for 24 h at –20 °C. Then, 1 mL of stock solution was transferred to a Mössbauer cell, which was previously frozen at liquid nitrogen temperature. The sample was frozen for 15 – 20 min and stored in liquid nitrogen until the measurement of the yield by means of Mössbauer spectroscopy.

*d) Synthesis from [(N$_3$N')Fe^{III}(CN)(^{n}Bu$_4$N)] (**2-Bu$_4$N**) precursor:*

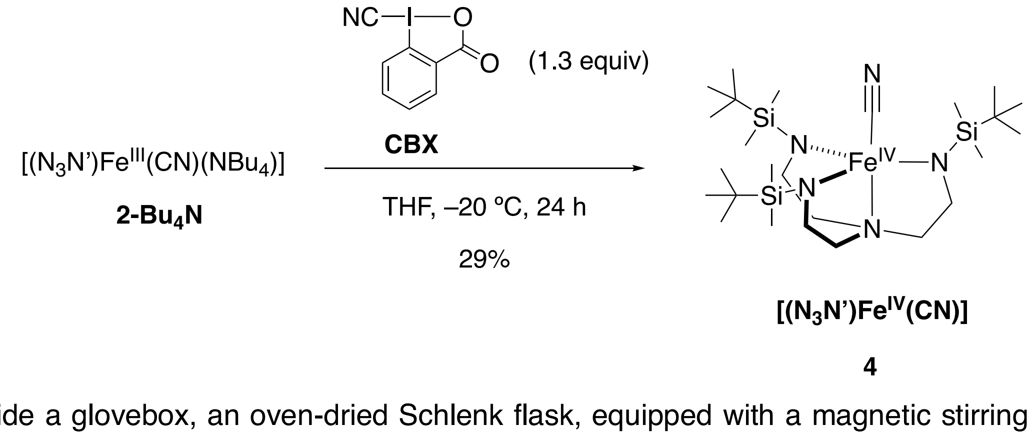

Inside a glovebox, an oven-dried Schlenk flask, equipped with a magnetic stirring bar, was charged with 1-cyano-1,2-benziodoxol-3-(1H)-one (CBX) (0.071 g, 0.26 mmol, 1.3 equiv) and [(N$_3$N')Fe^{III}(CN)(NBu$_4$)] (**2-Bu$_4$N**) (0.16 g, 0.2 mmol, 1 equiv). The Schlenk flask was closed, placed outside the glovebox and connected to a N_2 inlet. Then, THF (4 mL, 50 mM) was added slowly at –20 °C. The colour of the solution changed from red to dark purple gradually overnight. After stirring for 24 h an intense purple solution was

obtained. Then, 1 mL of stock solution was transferred to a Mössbauer cell, which was previously frozen at liquid nitrogen temperature. The sample was frozen for 15 – 20 min and stored in liquid nitrogen until the measurement of the yield by means of Mössbauer spectroscopy.

1.4.3. EPR spectroscopic data

Reaction of [(N_3N')Fe^{II}Li(THF)] with CDBX^F:

CDBX^F reagent was chosen for EPR experiments due to its higher solubility in comparison with CBX. Inside a glovebox, an oven-dried Schlenk flask, equipped with a magnetic stirring bar, was charged with 1-cyano-3,3-dimethy-(1*H*)-4-fluoro-1,2-benziodoxole (CDBX^F) (0.012 g, 0.26 mmol, 1 equiv) and [(N_3N')Fe^{II}Li(THF)] (**1**) (0.025 g, 0.04 mmol, 1 equiv). The Schlenk flask was closed, placed outside the glovebox and connected to a N_2 inlet. Then, THF (2 mL, 20 mM) was added slowly at –20 °C. The colour of the solution immediately changed from brown to an intense red. After stirring for 15 min, an aliquot of the crude (200 µL) was taken and transferred into an EPR tube under inert conditions. The tube was sealed with a septum and the solution was immediately frozen in liquid nitrogen. Afterwards, the sample was measured by EPR spectroscopy.

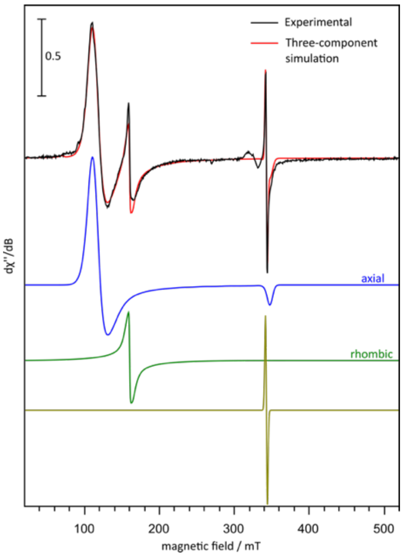

Figure SI-2. Perpendicular-mode X-band EPR spectra at 11.6 K of a freeze-quenched reaction of **1** (20 mM) and 1 equiv of **CDBX^F** in THF after 15 min (black line, 9.63 GHz, power 40 μW, modulation frequency 100 kHz, modulation amplitude 0.75 mT); three-component simulation for an axial (g_{eff} = [5.77, 6.05, 1.98], 81%), rhombic (g_{eff} = 4.29, 18%) and radical species (g_{eff} = 2.007, 1%).

Reaction of [(N_3N')Fe^{II}Li(THF)] with CBX^F:

Inside a glovebox, an oven-dried Schlenk flask, equipped with a magnetic stirring bar, was charged with 4-Fluoro-1-Cyano-1,2-benziodoxol-3-(1*H*)-one (**CBX^F**) (0.046 g, 0.16 mmol, 1 equiv) and [(N_3N')Fe^{II}Li(THF)] (0.05 g, 0.08 mmol, 1 equiv). The Schlenk flask was closed, placed outside the glovebox and connected to a N_2 inlet. Then, THF (4 mL, 20 mM) was added slowly at –20 °C. The colour of the solution immediately changed from brown to an intense red. After stirring for 15 min, an aliquot of the crude (200 µL) was taken and transferred into an EPR tube under inert conditions. The tube was sealed with a septum and the solution was frozen in liquid nitrogen. Then, the sample was measured by EPR spectroscopy.

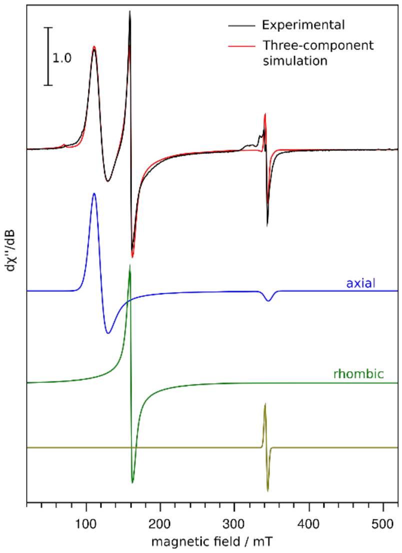

Figure SI-3. Perpendicular-mode X-band EPR spectra at 12.4 K of a freeze-quenched reaction of **1** (20 mM) and 1 equiv of **CBX^F** in THF after 15 min (black line, 9.63 GHz, power 40 μW, modulation frequency 100 kHz, modulation amplitude 0.75 mT); three-component simulation for an axial (g_{eff} = [5.83, 6.05, 1.99], 58%), rhombic (g_{eff} = 4.29, 41%) and radical species (g_{eff} = 2.007, 1%).

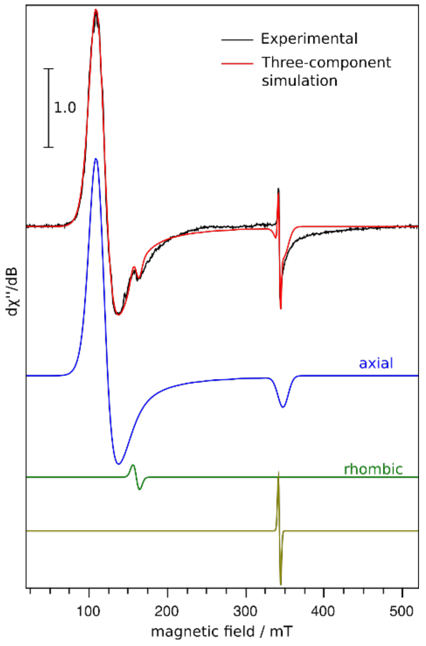

Figure SI-4. Perpendicular-mode X-band EPR spectra at 10.0 K of a frozen solution of $[(N_3N)Fe^{III}Li(CN)(^nBu_4N)]$ (**2-Bu$_4$N**) (3 mM) in THF (black line, 9.63 GHz, power 0.40 mW, modulation frequency 100 kHz, modulation amplitude 0.75 mT); three-component simulation for an axial (g_{eff} = [5.65, 6.10, 1.98], 99.4%), rhombic (g_{eff} = 4.29, 0.3%) and radical species (g_{eff} = 2.006, 0.3%).

1.4.4. ^{57}Fe Mössbauer spectroscopic data

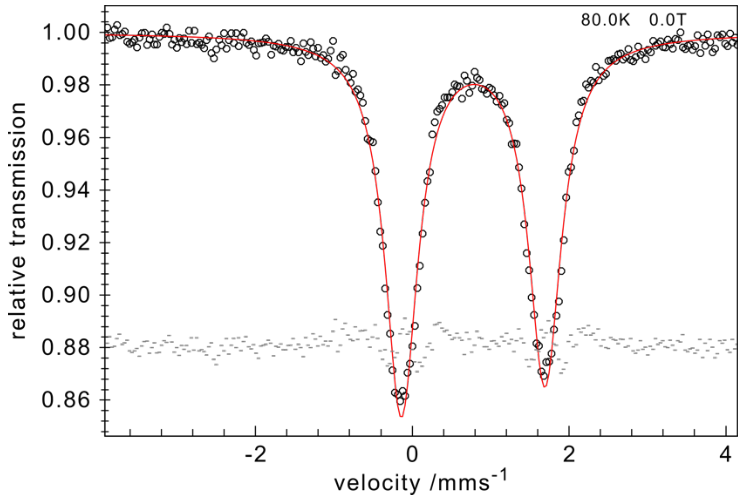

Figure SI-5. Zero-field ^{57}Fe Mössbauer spectrum of $[(N_3N')Fe^{II}Li(THF)]$ (**1**) solid at 80 K. Red line is the Lorenzian quadrupole doublet obtained by fitting with δ = 0.78 mm s^{-1}, $|\Delta E_Q|$ = 1.83 mm s^{-1}, w_L = 0.56 mm s^{-1}.

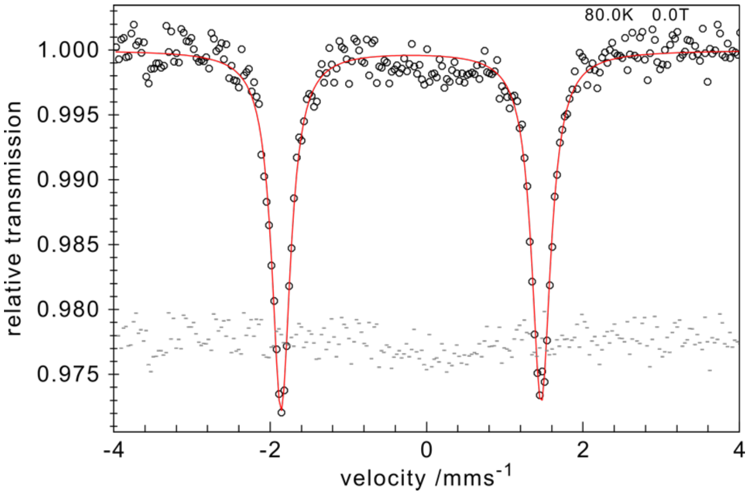

Figure SI-6. Zero-field ^{57}Fe Mössbauer spectrum of [(N_3N')Fe^{IV}CN] (**4**) solid at 80 K. Red line is the Lorenzian quadrupole doublet obtained by fitting with δ = –0.20 mm s^{-1}, $|\Delta E_Q|$ = 3.32 mm s^{-1}, w_L = 0.29 mm s^{-1}.

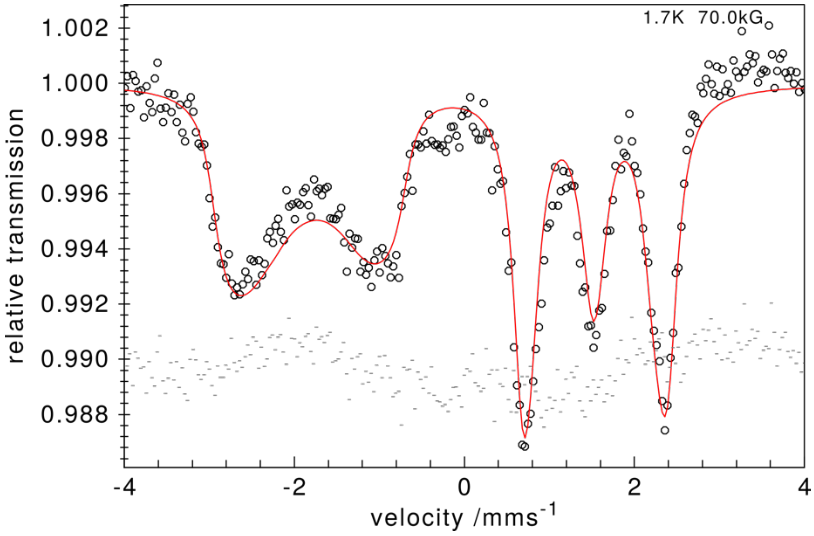

Figure SI-7. Magnetic ^{57}Fe Mössbauer spectrum of [(N_3N')Fe^{IV}CN] (**4**) solid at 1.7 K and 7.0 T. Red line is the Lorenzian quadrupole doublet obtained by fitting with S = 0, δ = –0.17 mm s^{-1}, ΔE_Q = –3.31 mm s^{-1}, w_L = 0.27 mm s^{-1} and asymmetry parameter η = 0.

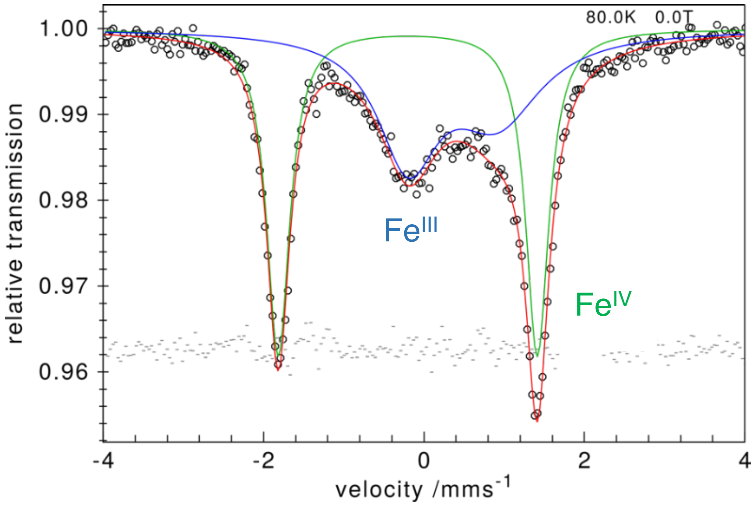

Figure SI-8. Zero-field ^{57}Fe Mössbauer spectrum of a frozen solution of a reaction of 50 mM [(N$_3$N')Fe^{II}Li(THF)] (**1**) with 2.2 equiv of **CBX** in THF after 24h at 80 K. Fit parameters: see Table SI-1.

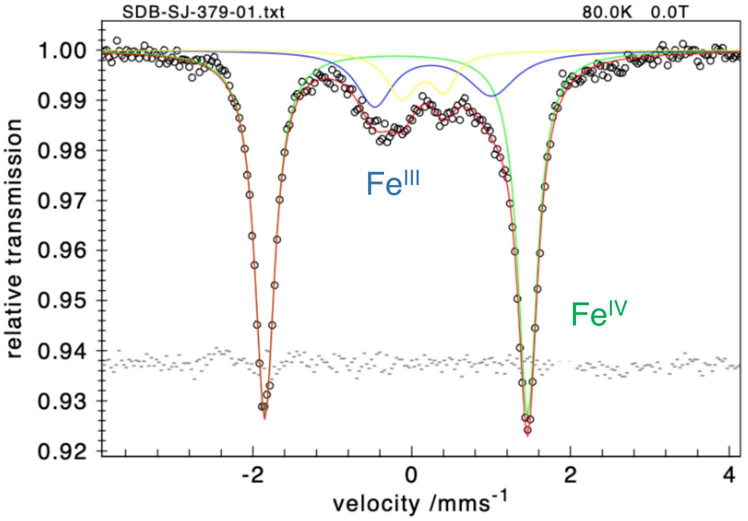

Figure SI-9. Zero-field ^{57}Fe Mössbauer spectrum at 80 K of a solid sample of [(N$_3$N')Fe^{IV}CN] (**4**) synthesized by reaction of 1 with CBX and after work-up. Red line is the sum of three-component fit. Green (target Fe^{IV}): δ = – 0.20 mm s^{-1}, $|\Delta E_Q|$ = 3.31 mm s^{-1}, w_L = 0.30 mm s^{-1}, 70%; blue (Fe^{III}): δ = 0.27 mm s^{-1}, $|\Delta E_Q|$ = 1.47 mm s^{-1}, w_L = 0.55 mm s^{-1}, $w_{2/1}$ = 1.34, 19%; yellow δ = 0.14 mm s^{-1}, $|\Delta E_Q|$ = 0.53 mm s^{-1}, w_L = 0.47 mm s^{-1}, $w_{2/1}$ = 0.76, 11%.

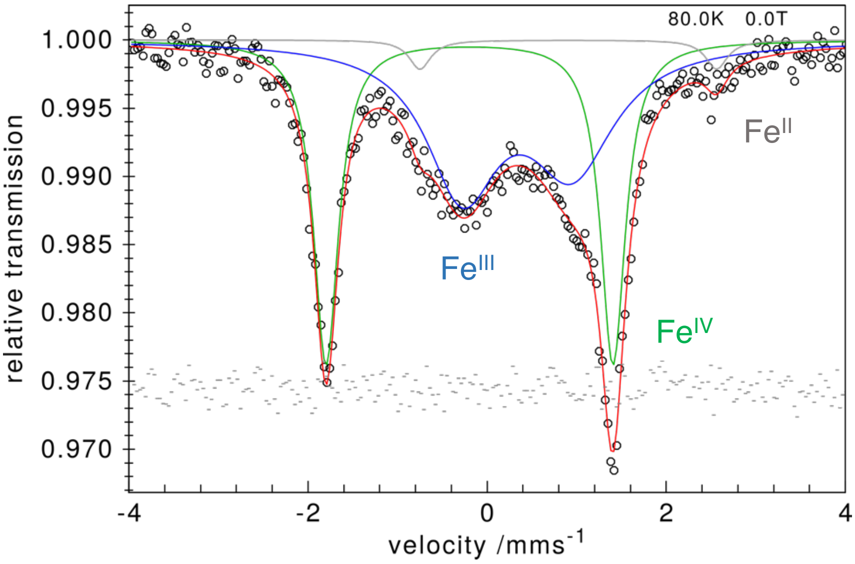

Figure SI-10. Zero-field ^{57}Fe Mössbauer spectrum at 80 K of frozen solution of a reaction of [(N$_3$N')Fe^{III}] (**3**), 50 mM, with 1.3 equiv of **CBX** in THF after 24h. Fit parameters: see Table SI-1.

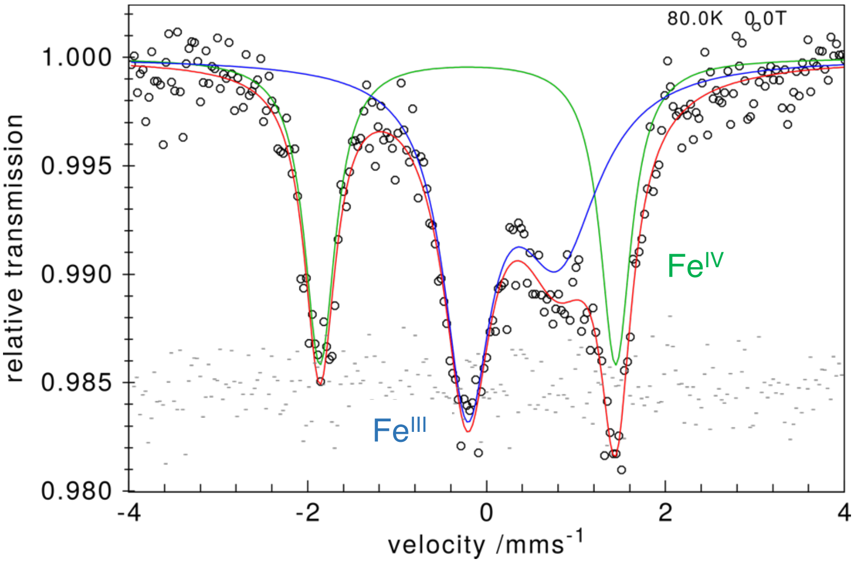

Figure SI-11. Zero-field ^{57}Fe Mössbauer spectrum of frozen solution of a reaction of *in situ* formed [(N$_3$N')Fe^{III}CNLi(THF)$_3$] (**2-Li^{THF}**), 50 mM, with 1.3 equiv of **CBX** in THF after 24h. Fit parameters: see Table SI-1.

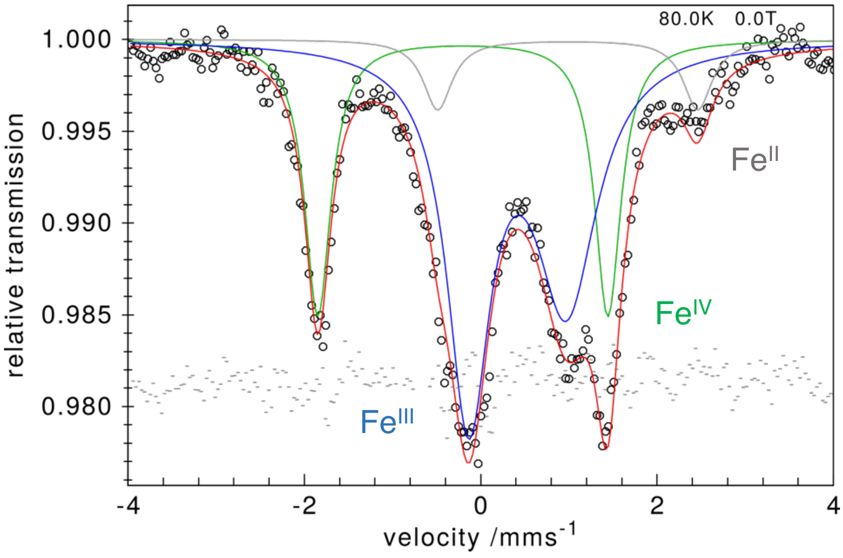

Figure SI-12. Zero-field ^{57}Fe Mössbauer spectrum of frozen solution of a reaction of $[(N_3N)Fe^{III}Li(CN)(^{n}Bu_4N)]$ (**2-Bu$_4$N**), 50 mM, with 1.3 equiv of **CBX** in THF after 24h Fit parameters: see Table SI-1.

Table SI-1. Summary of the ^{57}Fe Mössbauer spectroscopy data from crude reactions of Fe^{II} and Fe^{III} complexes with **CBX** reagent in THF at –20 °C.[a]

Iron complex	eq. of CBX	Spectrum in Figure	iron component after reaction		
			Fe^{IV}	Fe^{III}	Fe^{II}
**1**	2.2	SI-8	δ = –0.20 mm s^{-1}<br>$\lvert\Delta E_Q\rvert$ = 3.23 mm s^{-1}<br>w_L = 0.39 mm s^{-1}<br>**Rel. 51%**	δ = 0.36 mm s^{-1}<br>$\lvert\Delta E_Q\rvert$ = 1.12 mm s^{-1}<br>w_L = 1.35 mm s^{-1}<br>$w_{2/1}$ = 0.68<br>**Rel. 49%**	
**3**	1.3	SI-10	δ = –0.20 mm s^{-1}<br>$\lvert\Delta E_Q\rvert$ = 3.20 mm s^{-1}<br>w_L = 0.34 mm s^{-1}<br>**Rel. 43%**	δ = 0.34 mm s^{-1}<br>$\lvert\Delta E_Q\rvert$ = 1.22 mm s^{-1}<br>w_L = 1.55 mm s^{-1}<br>$w_{2/1}$ = 0.87<br>**Rel. 53%**	δ = 0.91 mm s^{-1}<br>$\lvert\Delta E_Q\rvert$ = 3.31 mm s^{-1}<br>w_L = 0.30 mm s^{-1}<br>**Rel. 3%**
**2-Li^{THF}**	1.3	SI-11	δ = –0.21 mm s^{-1}<br>$\lvert\Delta E_Q\rvert$ = 3.31 mm s^{-1}<br>w_L = 0.42 mm s^{-1}<br>**Rel. 39%**	δ = 0.29 mm s^{-1}<br>$\lvert\Delta E_Q\rvert$ = 1.02 mm s^{-1}<br>w_L = 1.12 mm s^{-1}<br>$w_{2/1}$ = 0.57<br>**Rel. 61%**	
**2-Bu$_4$N**	1.3	SI-12	δ = –0.20 mm s^{-1}<br>$\lvert\Delta E_Q\rvert$ = 3.29 mm s^{-1}<br>w_L = 0.36 mm s^{-1}<br>**Rel. 29%**	δ = 0.42 mm s^{-1}<br>$\lvert\Delta E_Q\rvert$ = 1.11 mm s^{-1}<br>w_L = 0.85 mm s^{-1}<br>$w_{2/1}$ = 0.70<br>**Rel. 62%**	δ = 0.99 mm s^{-1}<br>$\lvert\Delta E_Q\rvert$ = 2.95 mm s^{-1}<br>w_L = 0.40 mm s^{-1}<br>**Rel. 8%**

[a] δ: isomer shift (± 0.02 mm s^{-1}); $\lvert\Delta E_Q\rvert$: absolute value of quadrupole splitting; w_L: FWHM of Lorenzian quadrupole doublet; $w_{2/1}$: FWHM ratio of the two sides of the quadrupole doublet, thus representing its asymmetry caused by relaxation effects.

1.5. Synthesis of cyano Iodine(III) reagents

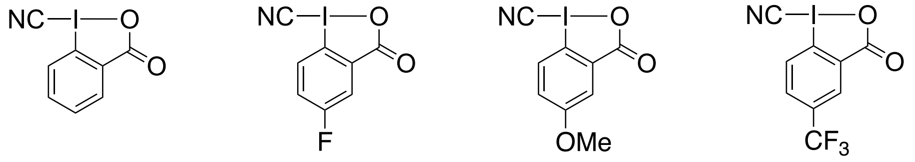

CBX **CBX^{F}** **CBX^{OMe}** **CBX^{CF3}**

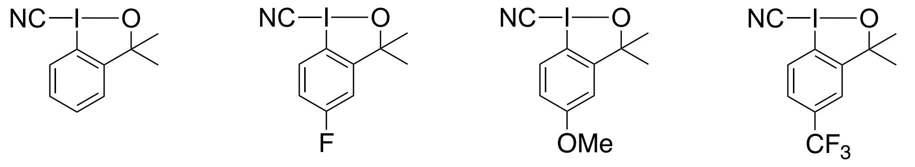

CDBX **CDBX^{F}** **CDBX^{OMe}** **CDBX^{CF3}**

Iodine(III) reagents **CBX**, **CBX^{F}** and **CDBX** were synthesized according to literature procedures.[24] Reagents **CBX^{OMe}**, **CBX^{CF3}**, **CDBX^{F}, CDBX^{OMe} and CDBX^{CF3}** have not been previously reported and their synthesis is described herein.

1.5.1. Synthesis of iodine(III) reagents CBX^{R} (R = OMe, CF$_3$)

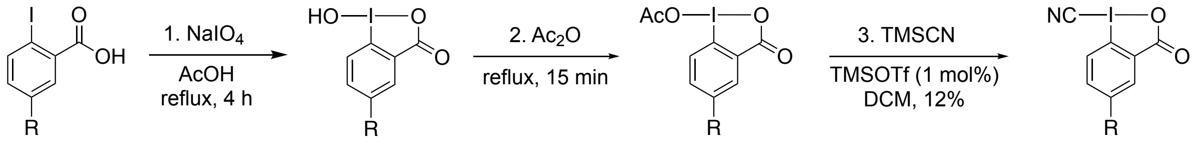

R = OMe, CF$_3$ **Int-1^{R}** **Int-2^{R}** **CBX^{R}**

General procedure:

Step 1: 2-iodobenzoic acid derivative (1 equiv) and $NaIO_4$ (1.05 equiv) were suspended in 30% (v:v) aq. AcOH (60 mL). The mixture was vigorously stirred and refluxed for 4 h. The reaction mixture was cooled to room temperature and quenched by addition of cold water (200 mL). After 1 h, the resulting crude was collected by filtration, washed on the filter with water (3 x 30 mL) and acetone (3 x 30 mL), and dried with N_2 flow in the dark and then *in vacuo* to give the product as a solid.

Step 2: 1-hydroxy-1,2-benziodoxol-3-(1*H*)-one derivative (1 equiv) was suspended in acetic anhydride (40 mL) and refluxed for 30 min. The resulting slightly yellow solution was allowed to cool down to room temperature and then it was cooled to 0°C for 1 h.

The white precipitate was filtered, and the mother liquor was again cooled to 0°C overnight. The suspension was once again filtered and the two batches of solid were combined and washed with pentane (2 x 30 mL) and dried *in vacuo* to give a white solid.

Step 3: 1-acetoxy-1,2-benziodoxol-3-(1*H*)-one derivative (1 equiv) was dissolved in dry dichloromethane (50 mL) under N_2. To this colourless solution trimethylsilyl cyanide (2 equiv) was added via syringe dropwise over a period of 5 min, followed by the addition of trimethylsilyl trifluoromethanesulfonate (1 mol%). Immediate precipitation occurred within 5 min and the reaction mixture was stirred at room temperature for 30 min to ensure the completion of the the reaction. The thick white suspension was diluted with dry pentane (20 mL) before filtration. The resulting mixture was filtered *via* cannula and the solid was washed with hexane (3 x 40 mL) until a colourless solution was observed. The solid was dried *in vacuo*, affording the product as a white solid.

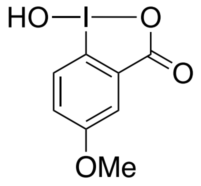

Int-1^{OMe}

4-Methoxy-1-hydroxy-1,2-benziodoxol-3-(1H)-one (**Int-1^{OMe}**). 2.15 g, 95% yield. White solid. **^{1}H-NMR** (300 MHz, DMSO-d_6): δ 8.01 (s, 1H), 7.66 (d, *J* = 8.7 Hz, 1H), 7.60-7.42 (m, 1H), 3.88 (s, 3H) ppm. **^{13}C-NMR** (75 MHz, DMSO-d_6): δ 167.4, 161.5, 133.0, 127.1, 121.6, 115.0, 109.0, 55.9 ppm. **IR** (film, cm^{-1}): 3075 (w), 2902 (w), 2413 (w), 1736 (w), 1612 (w), 1587 (w), 1565 (m), 1460 (s), 1415 (m), 1328 (m), 1269 (m), 1226 (m), 1139 (m), 1113 (w), 1103 (w), 1039 (m), 1009 (w), 960 (w), 918 (w), 901 (w). **HRMS (ESI):** m/z calcd for $C_8H_7IO_4$ [M + Na]$^+$: 316.9287, found: 316.9289.

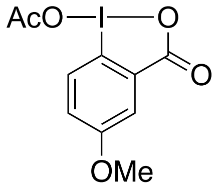

Int-2^{OMe}

4-Methoxy-1-acetoxy-1,2-benziodoxol-3-(1*H*)-one (**Int-2^{OMe}**). 0.85 g, 82% yield. White solid. **^{1}H-NMR** (300 MHz, $CDCl_3$): δ 7.80 (d, *J* = 9.1 Hz, 1H), 7.73 (d, *J* = 2.9 Hz, 1H), 7.46 (dd, *J* = 9.1, 2.9 Hz, 1H), 3.93 (s, 3H), 2.24 (s, 3H). **^{13}C-NMR** (126 MHz, $CDCl_3$): δ 176.5, 168.3,162.8, 130.7, 129.9, 124.6, 116.0, 106.9, 56.3, 20.4 ppm. **IR** (film, cm^{-1}): 2961 (w), 2932 (m), 2834 (w), 2648 (w), 1696 (s), 1591 (w), 1558 (m), 1468 (m), 1439 (w), 1411 (w), 1309 (w), 1282 (w), 1258 (w), 1223 (s), 1180 (w), 1101 (w), 1047 (s), 1007 (w), 880 (m), 815 (m), 670 (m). **HRMS (ESI):** m/z calcd for $C_{10}H_9IO_5$ [M + H]$^+$: 336.9573, found: 336.9581.

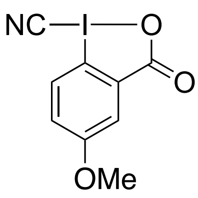

CBX^{OMe}

4-Methoxy-1-cyano-1,2-benziodoxol-3-(1*H*)-one (**CBX^{OMe}**). 0.66 g, 85% yield. White solid. **^{1}H-NMR** (300 MHz, DMSO-d$_6$): δ 8.15-8.05 (m, 1H), 7.65-7.54 (m, 2H), 3.90 (s, 3H). **^{13}C-NMR** (75 MHz, DMSO-d$_6$): δ 166.2, 162.6, 131.9, 128.6, 123.4, 115.7, 105.6, 87.4, 56.1 ppm. **IR** (film, cm^{-1}): 3095 (w), 2950 (w), 2159 (w), 1629 (s), 1582 (m), 1466 (m), 1440 (w), 1416 (w), 1324 (w), 1302 (s), 1276 (w), 1230 (m), 1195 (w), 1148 (w), 1113 (m), 1032 (m), 899 (m), 785 (s). **HRMS (ESI):** m/z calcd for $C_9H_6INO_3$ [M + Na]$^+$: 303.9465, found: 303.9471.

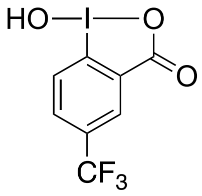

Int-1^{CF3}

4-Trifluoromethyl-1-hydroxy-1,2-benziodoxol-3-(1H)-one (**Int-1^{CF3}**). 0.89 g, 82% yield. White solid. **^{1}H-NMR** (300 MHz, DMSO-d$_6$): δ 8.39 (s, 1H) 8.33 (d, *J* = 8.3 Hz, 1H), 8.20 (s, 1H), 8.07 (d, *J* = 8.4 Hz, 1H) ppm. **^{13}C-NMR** (126 MHz, DMSO-d$_6$): δ 166.5, 133.0, 131.3 (q, J_{CF} = 32.8 Hz), 130.7 (q, J_{CF} = 3.0 Hz), 128.0 (q, J_{CF} = 1 Hz), 127.2 (d, J_{CF} = 3.6 Hz), 125.7, 123.5 (q, J_{CF} = 272.0 Hz) ppm. **^{19}F-NMR** (282 MHz, DMSO-d$_6$): δ –61.10 ppm. **IR** (film, cm^{-1}): 2812 (w), 2407 (w), 1596 (m), 1552 (w), 1461 (w), 1419 (w), 1304 (s), 1242 (w), 1167 (w), 1128 (s), 1065 (m), 1016 (w), 976 (w), 918 (w), 839 (m), 810 (w), 790 (w), 774 (m), 701 (s), 665 (m), 586 (s). **HRMS (ESI):** m/z calcd for $C_{10}H_6F_3IO_4$ [M + Na]$^+$: 354.9049, found: 354.9055.

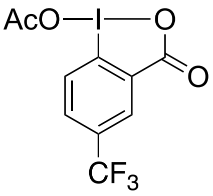

Int-2^{CF3}

4-Trifluoromethyl-1-acetoxy-1,2-benziodoxol-3-(1*H*)-one (**Int-2^{CF3}**). 1.01 g, 72 % yield. White solid. **^{1}H-NMR** (300 MHz, $CDCl_3$): δ 8.52 (s, 1H), 8.22 - 8.07 (m, 2H), 2.28 (s, 3H) ppm. **^{13}C-NMR** (126 MHz, $CDCl_3$): δ 176.7, 166.8, 134.4 (q, J_{CF} = 33.7 Hz), 132.5 (q, J_{CF} = 3.4 Hz), 130.6 (q, J_{CF} = 1 Hz), 130.6 (q, J_{CF} = 1Hz), 130.5, 130.2 (q, J_{CF} = 3.8 Hz), 122.5 (q, J_{CF} = 3.8 Hz), 122.0 20.4 ppm. **^{19}F-NMR** (282 MHz, $CDCl_3$): δ –62.76 ppm. **IR** (film, cm^{-1}): 2849 (w), 2421 (w), 1688 (w), 1595 (w), 1557 (s), 1461 (w), 1420 (w), 1306 (s), 1261 (w), 1243 (w), 1169 (w), 1131 (s), 1081 (w), 1066 (m), 1017 (w), 919 (w), 879 (w), 834 (w), 790 (w), 775 (s), 702 (s), 665 (m). **HRMS (ESI):** m/z calcd for $C_{10}H_6F_3IO_4$ [M + Na]$^+$: 396.9155, found: 396.9161.

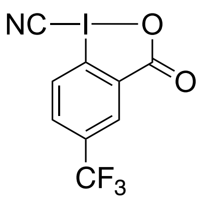

CBX^{CF3}

4-Trifluoromethyl-1-cyano-1,2-benziodoxol-3-(1*H*)-one (**CBX^{CF3}**). 0.46 g, 74% yield. White solid. **^{1}H-NMR** (300 MHz, DMSO-d$_6$): δ 8.51 (d, *J* = 8.5 Hz, 1H), 8.38 (d, *J* = 8.5 Hz, 1H), 8.30 (s, 1H) ppm. **^{13}C-NMR** (300 MHz, DMSO--d$_6$): δ 165.4,132.6 (q, J_{CF} = 3.6 Hz), 132.5 (q, J_{CF} = 32.7 Hz), 131.8 (q, J_{CF} = 1 Hz), 129.7, 127.7 (q, J_{CF} = 3.8 Hz), 123.6 (q, J_{CF} = 272.2 Hz), 122.6, 87.4 ppm. **^{19}F-NMR** (282 MHz, DMSO-d$_6$): δ –61.29 ppm. **IR** (film) (cm^{-1}): 452 (m), 3078 (w), 2980 (w), 2167 (w), 1631 (s), 1600 (w), 1578 (w), 1466 (w), 1419 (w), 1340 (w), 1328 (w), 1289 (s), 1253 (w), 1181 (m), 1147 (s), 1072 (s), 1004 (w), 928 (w), 880 (m), 837 (m), 780 (s), 739 (w), 703 (s), 664 (w), 601 (w), 550 (w), 506 (w), 452 (m). **HRMS (ESI):** m/z calcd for $C_9H_3F_3INO_2$ [M + H]$^+$: 341.9244, found: 341.9233.

1.5.2. Synthesis of iodine(III) reagents CDBX^R (R = F, OMe, CF_3)

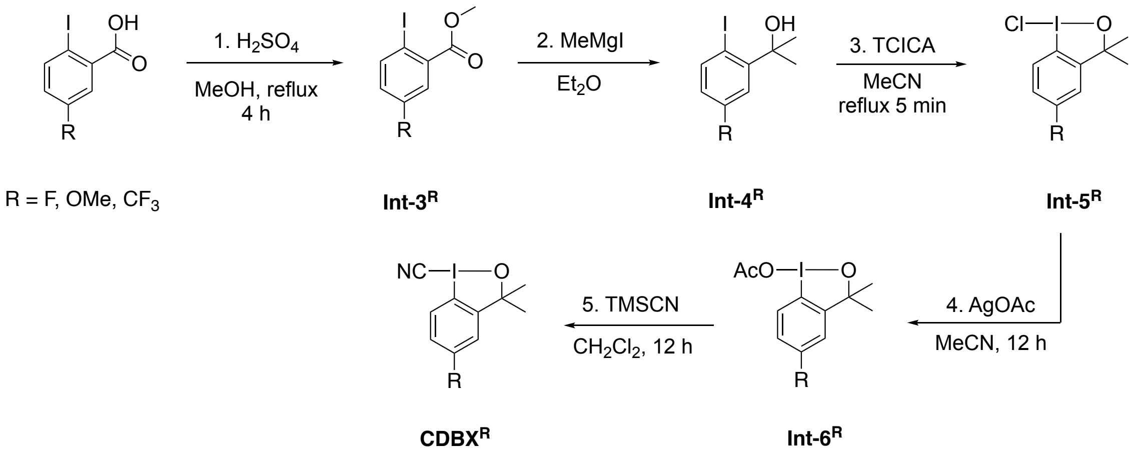

General procedure:

Step 1: The 2-iodobenzoic acid derivative (1 equiv) was dissolved in 40 mL of MeOH. To the stirred solution 12 equiv. of concentrated H_2SO_4 were added dropwise at 0 °C (carefully is exothermic). The reaction mixture was stirred at reflux (75 °C) for 4h. Then, the resulting mixture was cooled to room temperature and concentrated under reduced pressure. The residue was dissolved in EtOAc (10 mL) and washed with saturated aqueous $NaHCO_3$ (3 x 15 mL). The organic phase was dried over Na_2SO_4, filtered and the solvent was removed at reduced pressure to afford an oil, which was used without further purification in the next step.

Step 2: An oven-dried 25 mL three-necked round-bottom flask, equipped with a reflux condenser, a stirring bar and a 25 mL dropping funnel is charged with magnesium turnings (3.4 equiv) under argon atmosphere. The flask is charged with anhydrous Et_2O (5 mL). The dropping funnel is charged with a solution of methyl iodide (2.4 equiv) in Et_2O (3 mL). The methyl iodide solution is added dropwise over the suspension of magnesium turnings in Et_2O. The reaction is initiated, as evidenced by reflux, upon the addition of 3 mL of methyl iodide solution. The reaction mixture is immediately diluted with additional 3 mL of Et_2O that are added through the dropping funnel. After the addition is complete the reaction mixture is allowed to cool to room temperature. The brownish solution is allowed to stand until the remaining magnesium turnings have settled down. The Grignard is transferred to a solution of methyl 2-iodobenzoate derivative (1.1 equiv) in Et_2O (5 mL) at –78°C dropwise under vigorous stirring over 10 min. Additional Et_2O (2-4 mL) is used to rinse the flask. The mixture is left in the cooling bath and allowed to warm to room temperature overnight (15 h). The brown-green suspension is heated to reflux for 1.5h, then cooled to 0°C (ice water bath) and treated carefully with a saturated aqueous ammonium chloride solution (70 mL). A thick yellow precipitate forms and water (2 x 50 mL) is added until most of solid material dissolves. Then 100 mL of Et_2O is added, and the yellow suspension is filtered through a pad of Celite®. The organic phase is separated from the aqueous phase, and the aqueous phase is extracted with Et_2O (4 x 100 mL). The combined ethereal phases are dried over magnesium sulfate, filtered and the solvent is evaporated *in vacuo* to yield the title compound as a brownish oil. Due to problems of stability, the obtained compound is immediately used in the following step without purification.

Step 3: In 100 mL a three-necked round-bottom flask under argon atmosphere the 2-(2-iodo-phenyl)propan-2-ol derivative (3 equiv) is dissolved in MeCN (30 mL) and the solution is heated to 75°C. At this point the TCICA (1 equiv) in MeCN (20 mL) is added dropwise. The mixture is stirred at reflux 5 min and a yellow precipitate is formed. The crude was filtered over a short pad of Celite® while was still hot and the filtrate concentrated under reduced pressure. The crystallization is performed overnight at -20 °C. The yellow solid is collected by filtration washing with cold acetonitrile to afford the title compound**.**

Step 4: 1-chloro-3,3-dimethyl-3-(1H)-1,2-benziodoxole derivative (1 equiv) and silver acetate (1.05 equiv) were suspended under nitrogen in acetonitrile (30 mL). The mixture

was stirred in the dark at room temperature for 15 hours. The thin precipitate that appeared was removed by filtration and the clear solution was concentrated under reduce pressure to obtain the title compound.

Step 5: To a solution of acetoxy-3.3-dimethyl-3-(1H)-1,2-benziodoxole derivative (1 equiv) and CH_2Cl_2 (15 mL) was added dropwise trimethylsilyl cyanide (2 equiv) at rt under nitrogen. The clear colorless solution was stirred at rt for 20 hours. Solvent removal afforded a white solid, which was suspended in pentane (10 mL), filtered and dried *in vacuo*, affording the title compound.

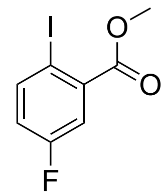

Int-3^F^

Methyl 5-Fluoro-2-iodobenzoate (**Int-3^F**). 2.80 g, 89% yield. Brown oil. **^{1}H-NMR** (300 MHz, $CDCl_3$): δ 7.91 (dd, *J* = 8.7, 5.4 Hz, 1H), 7.52 (dd, *J* = 8.9, 4.0 Hz, 1H), 6.91 (dt, *J* = 8.4, 4.2 Hz, 1H), 3.92 (s, 3H) ppm. **^{13}C-NMR** (75 MHz, $CDCl_3$) δ (ppm): 165.7 (d, J_{CF} = 2.5 Hz), 162.4 (d, J_{CF} = 249.3 Hz), 142.8 (d, J_{CF} = 7.2 Hz), 136.6, (d, J_{CF} = 7.1 Hz), 120.4 (d, J_{CF} = 21.5 Hz), 118.5 (d, J_{CF} = 24.1 Hz), 87.2 (d, J_{CF} = 3.6 Hz), 52.6 ppm. **^{19}F-NMR** (282 MHz, $CDCl_3$): δ –113.25 ppm. **IR** (film, cm^{-1}): 3071 (w), 2981 (w), 2952 (w), 1730 (s), 1598 (w), 1573 (m), 1462 (w), 1434 (s), 1396 (m), 1294 (m), 1264 (w), 1244 (s), 1198 (m), 1127 (m), 1084 (m), 1018 (s), 978 (m), 883 (m), 817 (m), 793 (w), 775 (s), 669 (w), 596 (m), 554 (w), 483 (w), 436 (w). **HRMS (ESI):** m/z calculated for $C_8H_6FIO_2$ [M + Na]$^+$: 302.9291, found: 302.9294.

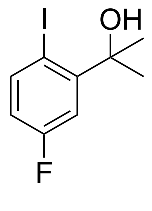

Int-4^F

2-(2-Iodo-5-Fluoro-phenyl)propan-2-ol (**Int-4^F**). 2.7 g, 70% yield. Brown oil. **^{1}H-NMR** (300 MHz, $CDCl_3$): δ 7.82 (dd, *J* = 8.3, 6.2 Hz, 1H), 7.39 (dd, *J* = 11.2, 2.8 Hz, 1H), 6.61 (td, *J* = 8.3, 2.8 Hz,1H), 2.98 (br, 1H), 1.70 (s, 6H) ppm. **^{13}C-NMR** (75 MHz, $CDCl_3$): δ 163.0 (d, J_{CF} = 245.3 Hz), 151.6 (d, J_{CF} = 7.3 Hz), 143.8 (d, J_{CF} = 7.4 Hz), 115.8 (d, J_{CF} = 21.7 Hz), 114.7 (d, J_{CF} = 23.9 Hz), 85.7 (d, J_{CF} = 3.2 Hz), 73.4, 29.5 ppm. **^{19}F-NMR** (282 MHz, $CDCl_3$): δ –113.16 ppm. **IR** (film, cm^{-1}): 3387 (w), 3072 (w), 2979 (m), 2931 (w), 1613 (w), 1585 (m), 1574 (w), 1484 (w), 1450 (m), 1389 (w), 1365 (w), 1260 (s), 1229 (w), 1197 (w), 1167 (m). **HRMS (ESI +):** m/z calculated for $C_9H_{10}FIO$ [M + Na]$^+$: 302.9658 found: 302.9660.

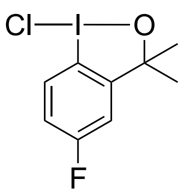

Int-5^F

1-Chloro-3,3-dimethyl-3-(1H)-5-Fluoro-1,2-benziodoxole (**Int-5^F**). 1.36 g, 45% yield. Yellow solid. **^{1}H-NMR** (300 MHz, $CDCl_3$): δ 7.96 (dd, *J* = 9.1, 4.7 Hz, 1H), 7.34-7.20 (m, 1H), 6.89 (dd, *J* = 8.4, 2.7 Hz, 1H), 1.54 (s, 6H) ppm. **^{13}C-NMR** (126 MHz, $CDCl_3$): δ 165.3 (d, J_{CF} = 250.1 Hz), 152.4 (d, J_{CF} = 7.2 Hz), 130.3 (d, J_{CF} = 9.0 Hz), 117.8 (d, J_{CF} = 23.8 Hz), 113.6.4 (d, J_{CF} = 24.2 Hz), 107.4 (d, J_{CF} = 1.7 Hz), 84.53 (d, J_{CF} = 1.9 Hz), 29.1 ppm. **^{19}F-NMR** (282 MHz, $CDCl_3$): δ –111.25 ppm. **IR** (film, cm^{-1}): 2970 (w), 2923 (w), 1590 (w), 1561 (w), 1461 (w), 1436 (m), 1378 (w), 1363 (w), 1275 (w), 1254 (w), 1179 (w), 1151 (m), 1109 (w), 1030 (w), 1001 (w), 940 (m), 862 (m), 758 (s), 713 (m), 650 (w), 620 (s). **HRMS (ESI +):** m/z calculated for C_9H_9ClFIO $[M + H]^+$: 314.9449, found 314.9471.

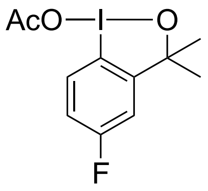

Int-6^F

1-Acetoxy-3,3-dimethyl-3-(1H)-5-Fluoro-1,2-benziodoxole (**Int-6^F**). 1.46 g, 77% yield. White solid. **^{1}H-NMR** (300 MHz, $CDCl_3$): δ 7.74 (dd, *J* = 9.0, 4.9 Hz, 1H), 7.20 *J* = 8.6, 2.6 Hz (td, 1H), 6.89 (dd, *J* = 8.7, 2.6 Hz, 1H), 2.10 (s, 3H), 1.51 (s, 6H) ppm. **^{13}C-NMR** (75 MHz, $CDCl_3$): δ 177.6, 164.9 (d, J_{CF} = 250.9 Hz), 152.7 (d, J_{CF} = 7.5 Hz), 131.6 (d, J_{CF} = 8.8 Hz), 117.4 (d, J_{CF} = 23.7 Hz), 113.7 (d, J_{CF} = 23.9 Hz), 109.0 (d, J_{CF} = 1.9 Hz), 84.3 (d, J_{CF} = 1.9 Hz), 29.2, 21.6 ppm. **^{19}F-NMR** (282 MHz, $CDCl_3$): δ – 111.74 ppm. **IR** (film, cm^{-1}): 2971 (w), 1699 (w), 1633 (s), 1588 (w), 1565 (w), 1466 (w), 1436 (w), 1423 (w), 1400 (w), 1368 (m), 1295 (s), 1257 (w), 1182 (w), 1155 (m), 1045 (w), 1033 (w), 1008 (m), 941 (m), 859 (m), 761 (s), 722 (m), 665 (s), 620 (s). **HRMS (ESI):** m/z calculated for $C_{11}H_{12}FIO_3$ $[M + H]^+$: 338.9893, found: 338.9872.

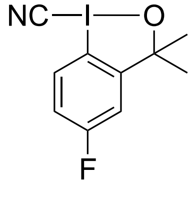

CDBX^F

1-Cyano-3,3-dimethyl-3-(1H)-5-Fluoro-1,2-benziodoxole (**CDBX^F**). 0.62 g, 58% yield. White solid. **^{1}H-NMR** (300 MHz, C_6D_6): δ 7.71 (dd, *J* = 8.7, 4.6 Hz, 1H), 6.51-6.36 (m, 2H), 1.06 (s, 6H). **^{13}C-NMR** (75 MHz, C_6D_6): δ 165.5 (d, J_{CF} = 249.0 Hz) , 151.4 (d, J_{CF} = 7.3 Hz), 130.3 (d, J_{CF} = 8.9 Hz), 117.8 (d, J_{CF} = 22.8 Hz), 113.8 (d, J_{CF} = 21.9 Hz), 105.1 (d, J_{CF} = 2.8 Hz), 97.0, 79.2 (d, J_{CF} = 3.4 Hz), 29.8 ppm **^{19}F-NMR** (282 MHz, C_6D_6): δ –111.48. **IR** (film, cm^{-1}): 3041 (w), 2988 (w), 2936 (w), 2139 (w), 1602 (w), 1573 (w), 1459 (s), 1449 (w), 1403 (w), 1375 (w), 1356 (w), 1268 (s), 1249 (w), 1189 (m), 1159 (w), 1135 (w), 1096 (w), 953 (s), 871 (m), 844 (m), 824 (s), 626 (s). **HRMS (ESI):** m/z calculated for $C_{10}H_9FINO$ $[M + H]^+$: 305.9791, found: 305.9788.

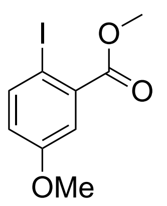

Int-3^{OMe}

Methyl 5-Methoxy-2-iodobenzoate (**Int-3^{OMe}**). 1.02 g, 97% yield. Yellow oil. ^{1}**H-NMR** (300 MHz, $CDCl_3$): δ 7.80 (d, *J* = 8.7 Hz, 1H), 7.32 (d, *J* = 3.1 Hz, 1H), 6.73 (dd, *J* = 8.7, 3.1 Hz, 1H), 3.91 (s, 3H), 3.79 (s, 3H) ppm. ^{13}**C-NMR** (75 MHz, $CDCl_3$): δ 166.8, 159.6, 141.9, 135.9, 119.4, 116.5, 82.5, 55.6, 52.6 ppm. **IR** (film, cm^{-1}): 2981 (w), 1737 (s), 1752 (s), 1564 (w), 1587 (m), 1462 (w) 1372 (m), 1233 (s), 1043 (s), 608 (w). **HRMS (ESI):** m/z calculated for $C_9H_9IO_3$ $[M + Na]^+$: 314.9494, found: 314.9495.

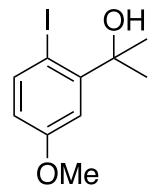

Int-4^{OMe}

2-(2-Iodo-5-methoxy-phenyl)propan-2-ol (**Int-4^{OMe}**). 2.50 g, 78% yield. Brown oil. ^{1}**H-NMR** (300 MHz, $CDCl_3$): δ 7.82 (d, *J* = 8.6 Hz, 1H), 7.25 (d, *J* = 3.1 Hz, 1H), 6.50 (dd, *J* = 8.6, 3.1 Hz, 1H), 3.79 (s, 3H), 2.50 (br, 1H), 1.74 (s, 6H) ppm. ^{13}**C-NMR** (75 MHz, $CDCl_3$): δ 143.3, 116.9, 114.1, 113.8, 112.0, 110.7, 73.6, 55.5, 31.9, 29.6 ppm. **IR** (film) (cm^{-1}): 3416 (w), 2972 (m), 2934 (w), 2835 (w), 1584 (s), 1564 (w), 1487 (w), 1461 (s), 1433 (w), 1400 (w), 1383 (w), 1363 (w), 1286 (s), 1235 (m), 1216 (w) **HRMS (ESI):** m/z calculated for $C_{10}H_{13}IO_2$ $[M + Na]^+$: 314.9858, found: 314.9861. Low stability, decompose in silica gel.

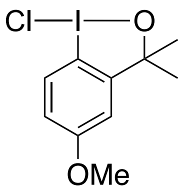

Int-5^{OMe}

1-chloro-3,3-dimethyl-3-(1H)-5-methoxy-1,2-benziodoxole (**Int-5^{OMe}**). 1.6 g, 57% yield. Yellow solid. ^{1}**H-NMR** (300 MHz, $CDCl_3$): δ 7.85 (d, J = 9.1 Hz, 1H), 7.08 (dd, *J* = 9.1, 2.7 Hz, 1H), 6.68 (d, *J* = 2.7 Hz, 1H), 3.87 (s, 3H), 1.53 (s, 6H) ppm. ^{13}**C-NMR** (75 MHz, $CDCl_3$): δ 162.7, 151.3, 129.4, 115.7, 112.6, 103.4, 84.8, 56.1, 29.2 ppm. **IR** (film, cm^{-1}): 3278 (m), 1597 (w), 1438 (w), 1323 (s), 1147 (s), 1061 (w), 1026 (s), 804 (m), 783 (m), 661 (m), 508 (m). **HRMS (ESI):** m/z calculated for $C_{10}H_{12}ClINO_2$ $[M + H]^+$: 326.9649, found: 326.9650.

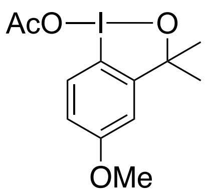

Int-6^{OMe}

1-Acetoxy-3.3-dimethyl-3-(1H)-5-methoxy-1,2-benziodoxole (**Int-6^{OMe}**). 1.7 g, 87% yield. White solid. ^{1}**H-NMR** (300 MHz, $CDCl_3$): δ 7.63 (d, *J* = 9.0 Hz,1H), 7.02 (dd, *J* = 9.0, 2.6 Hz, 1H), 6.69 (d, *J* = 2.6 Hz, 1H), 3.86 (s, 3H), 2.10 (s, 3H), 1.50 (s, 6H) ppm. ^{13}**C-NMR** (75 MHz, $CDCl_3$): δ 177.4, 162.1, 151.4, 130.6, 115.6, 112.4, 104.7, 84.3, 55.9, 29.2, 21.6 ppm. **IR** (film, cm^{-1}): 2966 (w), 1634 (s), 1588 (w), 1566 (w), 1465 (w), 1436 (w), 1361 (s), 1297 (s), 1257 (w), 1181 (w), 1155 (m), 1111 (w), 1035 (w), 1008 (m), 941 (m), 926 (w), 859 (m), 762 (w), 752 (s), 721 (m), 666 (s). **HRMS (ESI):** m/z calculated for $C_{12}H_{15}INO_4$ $[M + H]^+$: 351.0093, found: 351.0097.

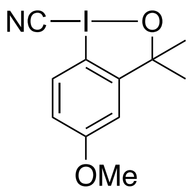

CDBX^{OMe}

1-Cyano-3,3-dimethyl-3-(1H)-5-methoxy-1,2-benziodoxole (**CDBX^{OMe}**). 1.46 g, 82% yield. White powder. **^{1}H-NMR** (300 MHz, C_6D_6): δ 7.86 (d, *J* = 9.1 Hz, 1H), 6.53 (d, *J* = 2.7 Hz, 1H), 6.33 (dd, *J* = 9.1, 2.7 Hz, 1H), 3.12 (s, 3H), 1.20 (s, 6H) ppm. **^{13}C-NMR** (75 MHz, C_6D_6): δ 163.1, 150.3, 129.4, 115.7, 133.3, 100.8, 97.5, 79.4, 55.3, 30.1 ppm. **IR** (film, cm^{-1}): 3001 (w), 2982 (w), 2965 (w), 2920 (w), 2845 (w), 2131 (w), 1578 (m), 1470 (m), 1457 (w), 1442 (w), 1397 (w), 1377 (w), 1358 (w), 1315 (m), 1280 (w), 1258 (m), 1227 (s), 1185 (w), 1155 (m), 1032 (s), 1001 (w), 952 (m), 905 (w), 877 (m), 838 (m), 809 (s). **HRMS (ESI):** m/z calcd for $C_{11}H_{12}INO_2$ [M + H]$^+$: 317.9991, found: 317.9992.

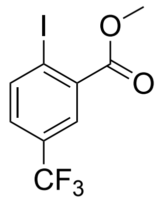

Int-3^{CF3}

Methyl 5-Trifluoromethyl-2-iodobenzoate (**Int-3^{CF3}**). 0.92 g, 85% yield. Yellow oil. **^{1}H-NMR** (300 MHz, $CDCl_3$): δ 8.14 (d, *J* = 8.2 Hz, 1H), 8.05 (d, *J* = 1.8 Hz, 1H), 7.38 (dd, *J* = 8.3, 1.8 Hz, 1H), 3.97 (s, 3H) ppm. **^{13}C-NMR** (75 MHz, $CDCl_3$): δ 165.7, 142.3, 135.8, 130.8 (q, J_{C-F} = 33.6 Hz), 128.9 (q, J_{C-F} = 4 Hz), 127.7 (q, J_{C-F} = 4 Hz), 123.5 (q, J_{C-F} = 271.1 Hz), 98.7, 52.9 ppm. **^{19}F-NMR** (282 MHz, $CDCl_3$): δ – 63.16 ppm. **IR** (film, cm^{-1}): 3440 (w), 2980 (w), 2250 (w), 2125 (w), 1664 (w), 1382 (w), 1252 (w), 1154 (w), 1053 (w), 1024 (s), 1005 (w), 820 (m), 758 (m). **HRMS (ESI):** m/z calculated for $C_9H_6F_3IO_2$ [M + Na]$^+$: 352.9262, found: 352.9268.

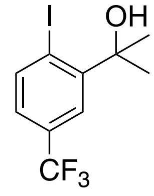

Int-4^{CF3}

Methyl 5-Trifluoromethyl-2-iodobenzoate (**Int-4^{CF3}**). 0.8 g, 86 % (50% purity) yield. Brown oil. **^{1}H-NMR** (300 MHz, $CDCl_3$): δ 7.86 – 7.72 (m, 1H), 6.92 (dd, *J* = 11, 3.1 Hz, 1H), 6.63 - 6.52 (m, 1H), 2.42 (br, 1H), 1.64 (s, 6H) ppm. **^{19}F-NMR** (282 MHz, $CDCl_3$): δ –63.48 ppm. **IR** (film, cm^{-1}): 3206 (w), 3082 (m), 2970 (w), 2886 (w), 2832 (w), 2780 (w), 1756 (w), 1702 (s), 1561 (w), 1460 (m), 1437 (w), 1414 (w), 1401 (s), 1363 (w), 1275 (w), 1254 (w), 1179 (w), 1150 (w), 1108 (w), 1061 (w), 1052 (w), 1030 (w), 1001 (w), 940 (w), 861 (w), 780 (w), 758 (s) **HRMS (ESI):** m/z calcd for $C_{10}H_{10}F_3IO$ [M + Na]$^+$: 352.9626, found: 352.9631. This compound is unstable and it decomposed during the column chromatography. The next step was carried out without purification.

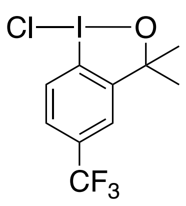

Int-5^{CF3}

1-Chloro-1,2-benziodoxol-3-(1*H*)-one (**Int-5^{CF3}**). 0.66 g, 75 % yield. Yellow solid. **^{1}H-NMR** (300 MHz, $CDCl_3$): δ 7.74 (d, *J* = 8.7 Hz, 1H), 7.38 – 7.18 (m, 1H), 6.90 (d, *J* = 9.4 Hz,1H), 1.28 (s, 6H) ppm. **^{13}C-NMR** (126 MHz, $CDCl_3$): δ 150.2, 130.6 (q, $J_{C\text{-}F}$ = 32 Hz), 128.8, 128.1 (q, $J_{C\text{-}F}$ = 2.3 Hz), 124.5 (q, $J_{C\text{-}F}$ = 272.8 Hz), 123.7 (q, $J_{C\text{-}F}$ = 3.8 Hz), 121.5 (q, $J_{C\text{-}F}$ = 4.1 Hz), 72.6, 31.9 ppm. **^{19}F-NMR** (282 MHz, $CDCl_3$): δ –63.12 ppm. **IR** (film, cm^{-1}): 2769 (w), 2829 (w), 1533 (m), 1488 (w), 1465 (w), 1365 (w), 1336 (w), 1321 (m), 1296 (m), 1257 (w), 1182 (w), 1156 (m), 1112 (w), 1045 (w), 1034 (w), 1009 (w), 941 (m), 927 (w), 859 (m), 762 (m), 722 (w), 665 (m), 620 (m), 558 (w), 498 (w), 485 (m), 439 (w). **HRMS (ESI):** m/z calcd for $C_{10}H_9ClF_3IO$ $[M + H]^+$: 364.9417, found: 364.9423.

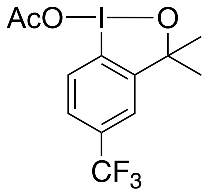

Int-6^{CF3}

1-Acetoxy-3.3-dimethyl-3-(1H)-5-Trifluoromethyl-1,2-benziodoxole (**Int-6^{CF3}**). 0.67 g, 95% yield. White solid. **^{1}H-NMR** (300 MHz, $CDCl_3$): δ 7.81 – 7.78 (m, 1H), 7.48 – 7.46 (m, 1H), 7.18 (d, *J* = 1.6 Hz, 1H), 2.11 (s, 3H), 1.53 (s, 6H) ppm. **^{13}C-NMR** (126 MHz, $CDCl_3$): δ 176.6, 166.8, 134.6 (q, $J_{C\text{-}F}$ = 34.4 Hz), 132.5 (q, $J_{C\text{-}F}$ = 5.5 Hz), 130.5 (q, $J_{C\text{-}F}$ = 3.6 Hz), 130.1 (q, $J_{C\text{-}F}$ = 3.6 Hz), 122.9 (q, $J_{C\text{-}F}$ = 274.8 Hz), 122.0, 71.8, 31.9, 20.3 ppm **^{19}F-NMR** (282 MHz, $CDCl_3$): δ –62.99 ppm. **IR** (film, cm^{-1}): 2963 (w), 2934 (w), 2872 (w), 1593 (w), 1488 (s), 1463 (w), 1428 (w), 1397 (w), 1381 (w), 1339 (w), 1283 (w), 1215 (m), 1136 (w), 1090 (s), 1014 (m), 1003 (w), 826 (s), 788 (w), 762 (w), 752 (w), 733 (w), 637 (w), 607 (m), 586 (w), 572 (w), 523 (s), 477 (w). **HRMS (ESI):** m/z calcd for $C_{12}H_{12}F_3IO_3$ $[M + H]^+$: 388.9861, found: 388.9865.

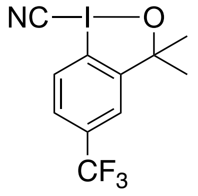

CDBX^{CF3}

1-Cyano-3,3-dimethyl-3-(1H)-5-Trifluoromethyl-1,2-benziodoxole (**CDBX^{CF3}**). 0.45 g, 76% yield. White solid. **^{1}H-NMR** (75 MHz, $CDCl_3$): δ 8.03 (d, *J* = 8.1 Hz, 1H), 7.69 – 7.49 (m, 1H), 7.32 (d, *J* = 7.0 Hz, 1H), 1.47 (s, 6H) ppm. **^{13}C-NMR** (126 MHz, $CDCl_3$): δ 163.2, 138.9, 132.3, 127.3 (q, $J_{C\text{-}F}$ = 34 Hz), 125.4 (q, $J_{C\text{-}F}$ = 3.4 Hz), 124.3 (q, $J_{C\text{-}F}$ = 2.8 Hz) 120.0 (q, $J_{C\text{-}F}$ = 272.4 Hz), 95.2, 56.1, 31.9 ppm. **^{19}F-NMR** (282 MHz, $CDCl_3$): δ –63.56 ppm. **IR** (film, cm^{-1}): 3650 (w), 3048 (w), 2971 (w), 2921 (w), 2138 (w), 1560 (w), 1458 (w), 1432 (w), 1357 (w), 1265 (w), 1249 (w), 1156 (m), 1110 (w), 1033 (w), 1002 (w), 952 (m), 866 (m). **HRMS (ESI):** m/z calcd for $C_{11}H_9F_3INO$ $[M + H]^+$: 355.0949, found: 355.0953.

1.6. UV/Vis characterization of the iron complexes

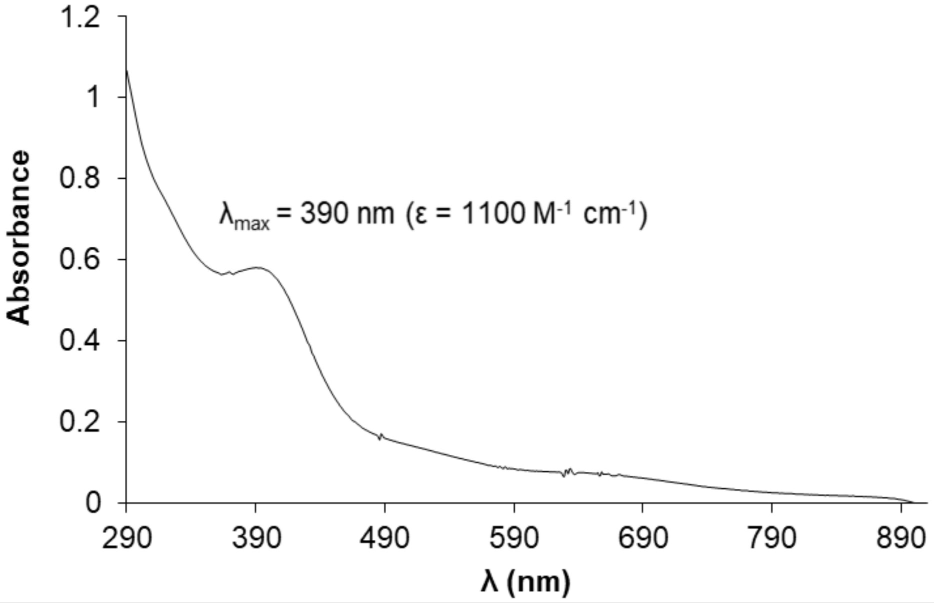

Figure SI-13. UV/Vis spectra of a 2 mM solution of [(N_3N')Fe^{II}Li(THF)] (**1**) in THF at 25ºC.

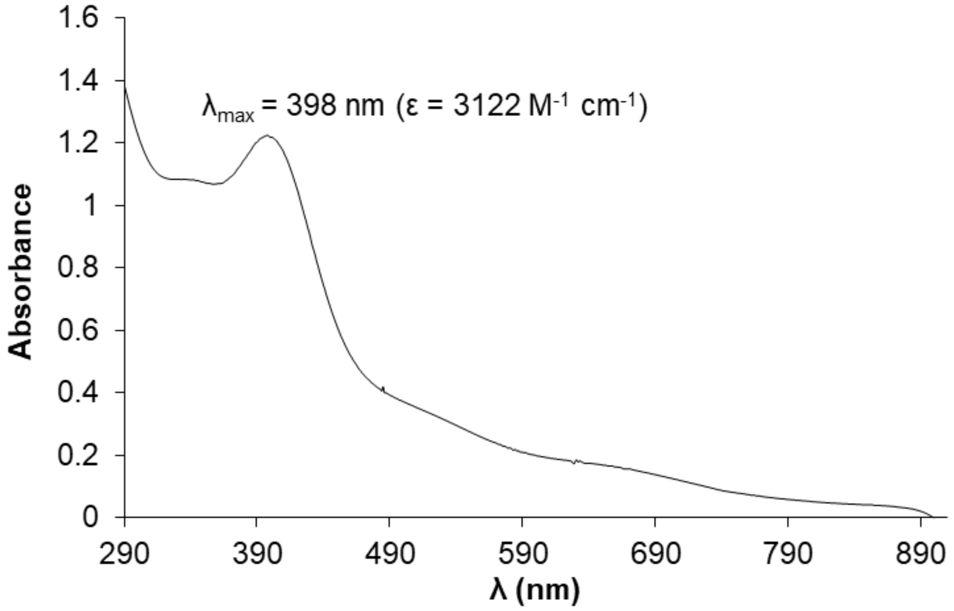

Figure SI-14. UV/Vis spectra of a 2 mM solution of [(N_3N')Fe^{III}] (**3**) in THF at 25ºC.

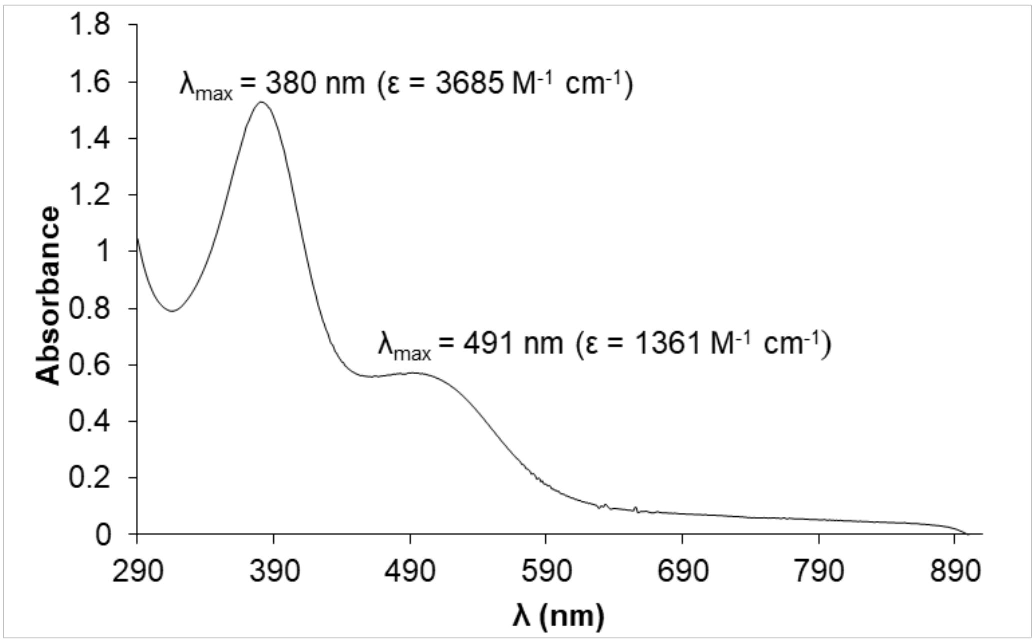

Figure SI-15. UV/Vis spectra of a 2 mM solution of $[(N_3N')Fe^{III}(CN)(^nBu_4N)]$ (**2-Bu$_4$N**) in THF at 25ºC.

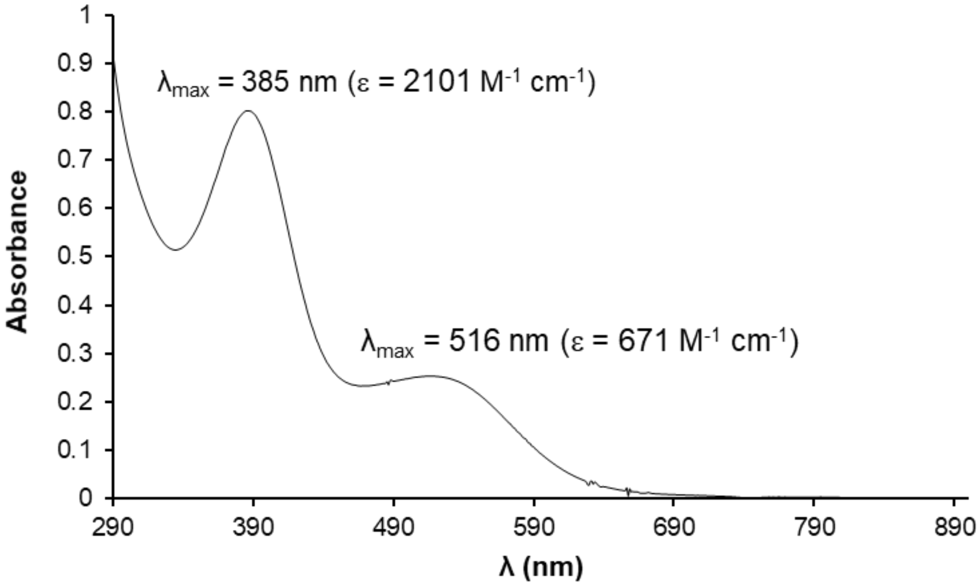

Figure SI-16. UV/Vis spectra of a 2 mM solution of $[(N_3N')Fe^{III}(CN)Li(12\text{-c-}4)]$ (**2-Li^{crown}**) in THF at 25ºC.

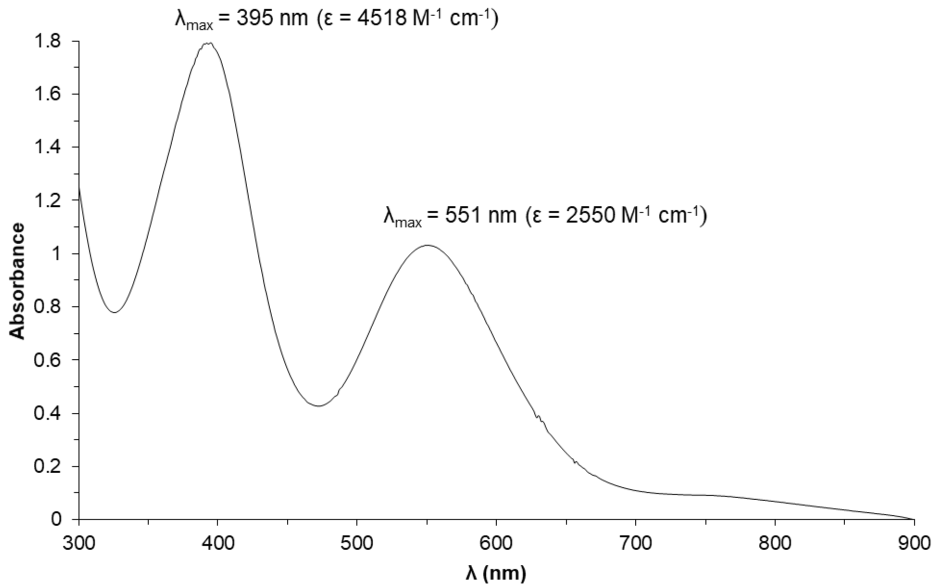

Figure SI-17. UV/Vis spectra of a 2 mM solution of [(N_3N')Fe^{IV}(CN)] (**4**) in THF at -20 °C.

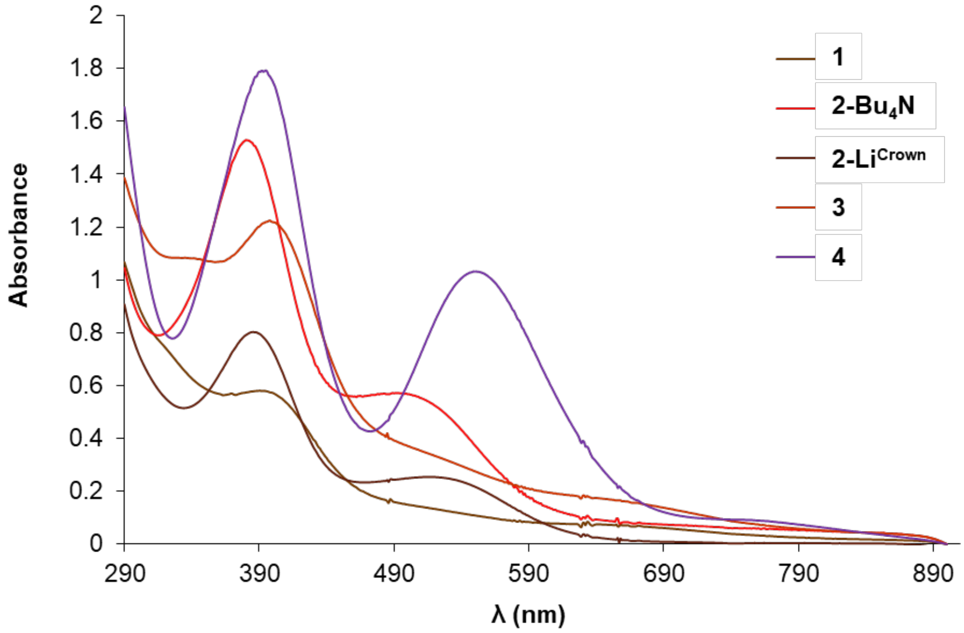

Figure SI-18. UV-Vis absorption spectra of a 2 mM solution of complexes **1, 2, 3-Bu$_4$N** and **3-Li^{crown}** complexes measured at 2 mM in THF at 25°C except for compound **4** at –20 °C with a cell path of 0.2 cm.

1.7. Cyclic voltammetry experiments

1.7.1. Hypervalent iodine reagents

We have measured the reduction potential of a variety of CBX^R and $CDBX^R$ (R = H, F, OMe, CF_3) derivatives by means of cyclic voltammetry in THF and MeCN solvents at 25°C.

Table SI-2. Table of reduction potential in THF

**I(III) reagent**		**E_{pc} (V vs. Fc^+/Fc)[a]**
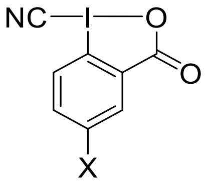	X = H	-1.58
	X = F	-1.56
	X = OMe	-1.56
	X = CF_3	-1.59
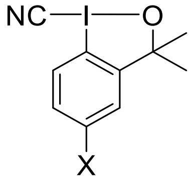	X = H	-2.35
	X = F	-2.50
	X = OMe	-2.56
	X = CF_3	-2.48

[a] Cyclic Voltammetry performed at 3 mM of the cyano λ^3-iodane reagent in THF at 25°C using as electrolyte $^nBu_4NPF_6$ (0.1M), at scan rate of 50 mV/s and using ferrocene as internal reference. A three-electrode electrochemical cell has been used: glassy carbon as working electrode, platinum wire as auxiliary electrode and $Ag/AgNO_3$ (0.01M) as reference electrode.

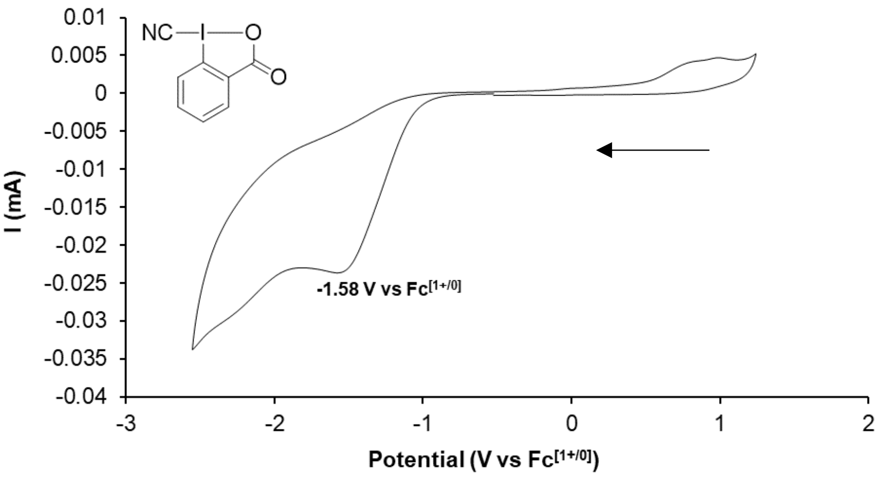

Figure SI-19. Cyclic voltammogram of 1-cyano-1,2-benziodoxol-3-(1*H*)-one (CBX) (3 mM) in THF at 25ºC.

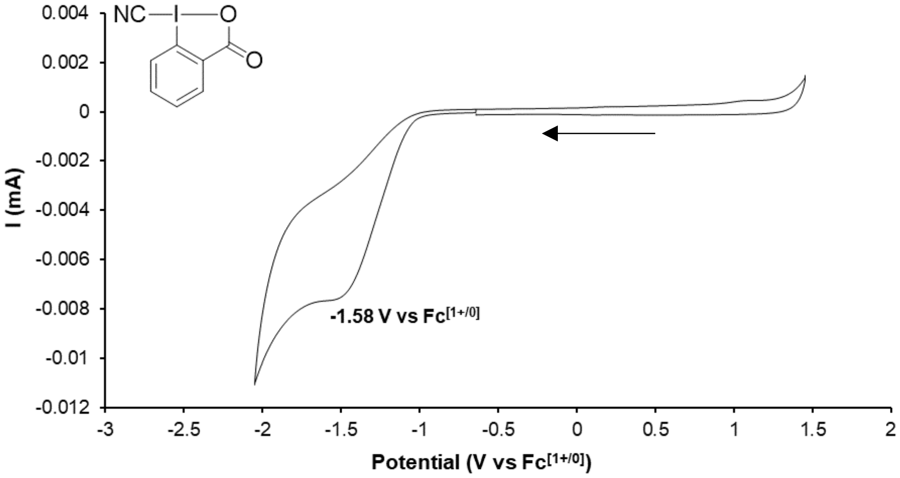

Figure SI-20. Cyclic voltammogram of 1-cyano-1,2-benziodoxol-3-(1*H*)-one (**CBX**) (3 mM) in THF at -40 ºC.

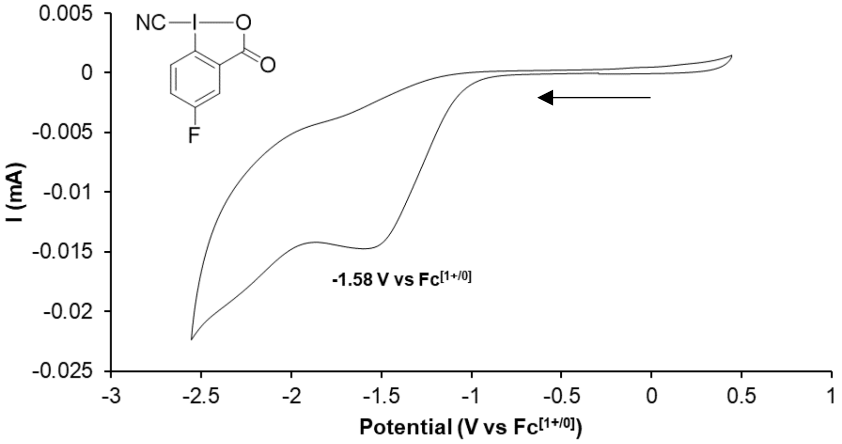

Figure SI-21. Cyclic voltammogram of 4-Fluoro-1-Cyano-1,2-benziodoxol-3-(1*H*)-one (**CBX**^{F}) (3 mM) in THF at 25°C.

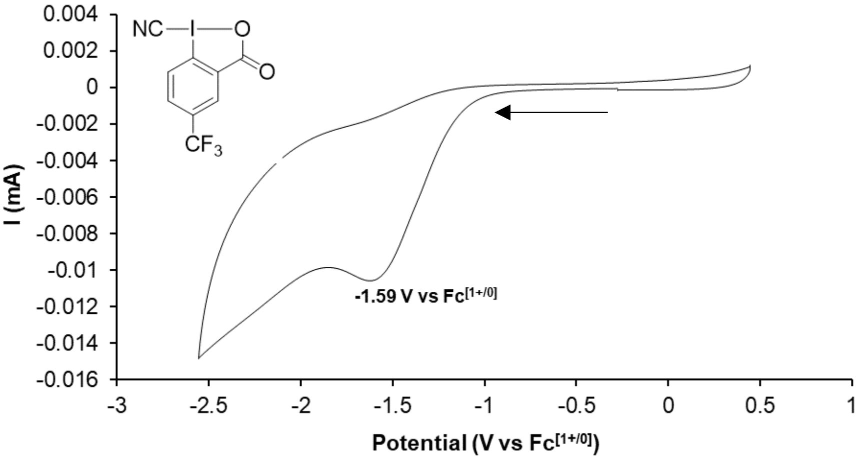

Figure SI-22. Cyclic voltammogram of 4-Trifluoromethyl-1-Cyano-1,2-benziodoxol-3-(1*H*)-one (**CBX**^{CF3}) (3 mM) in THF at 25°C.

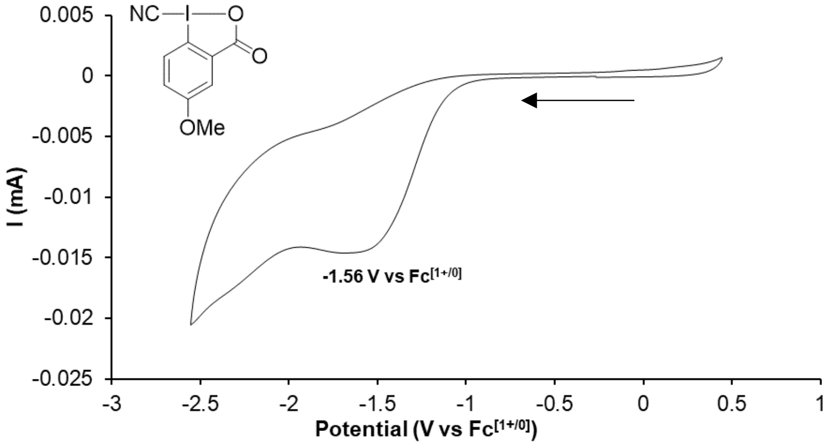

Figure SI-23. Cyclic voltammogram of 4-Methoxyl-1-Cyano-1,2-benziodoxol-3-(1*H*)-one (**CBX**^{OMe}) (3 mM) in THF at 25°C.

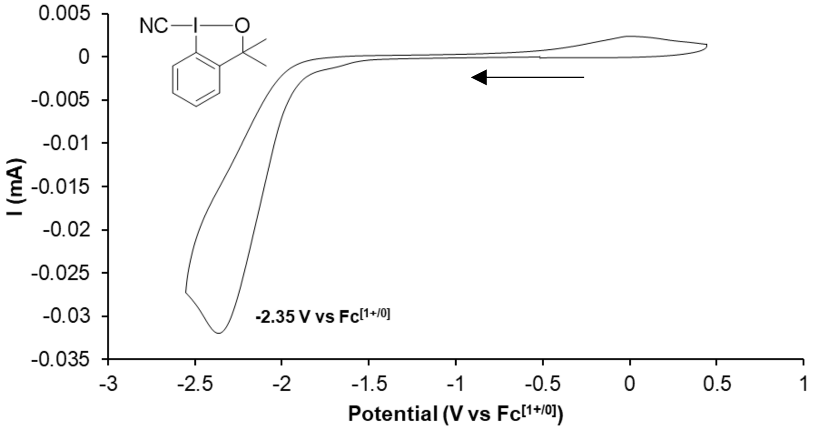

Figure SI-24. Cyclic voltammogram of 1-cyano-3,3-dimethy-(1*H*)-1,2-benziodoxole (**CDBX**) (3 mM) in THF at 25°C.

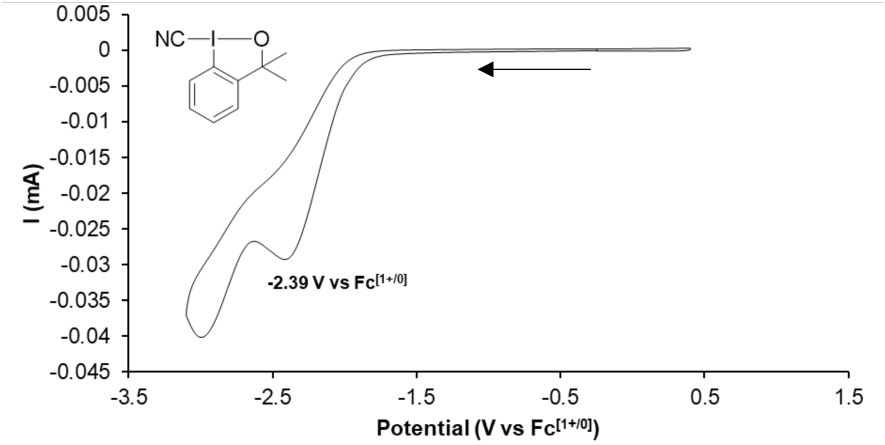

Figure SI-25. Cyclic voltammogram of 1-cyano-3,3-dimethy-(1*H*)-1,2-benziodoxole (**CDBX**) at (3 mM) in THF at -40°C.

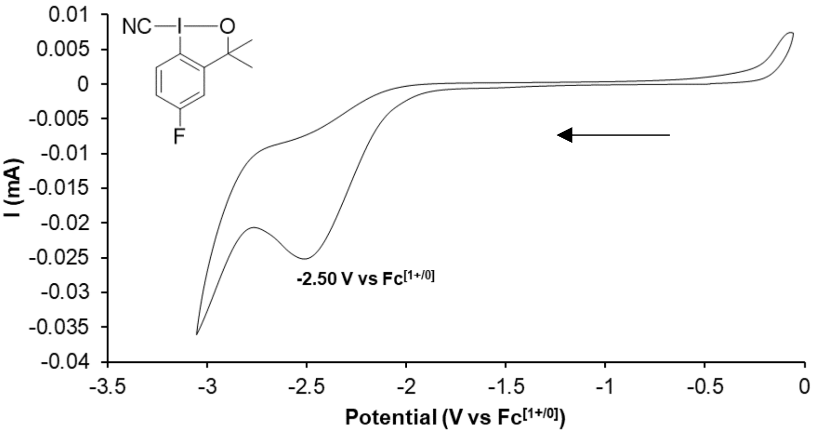

Figure SI-26. Cyclic voltammogram of 1-cyano-3,3-dimethy-(1*H*)-4-fluoro-1,2-benziodoxole (**CDBX^F**) (3 mM) in THF at 25°C.

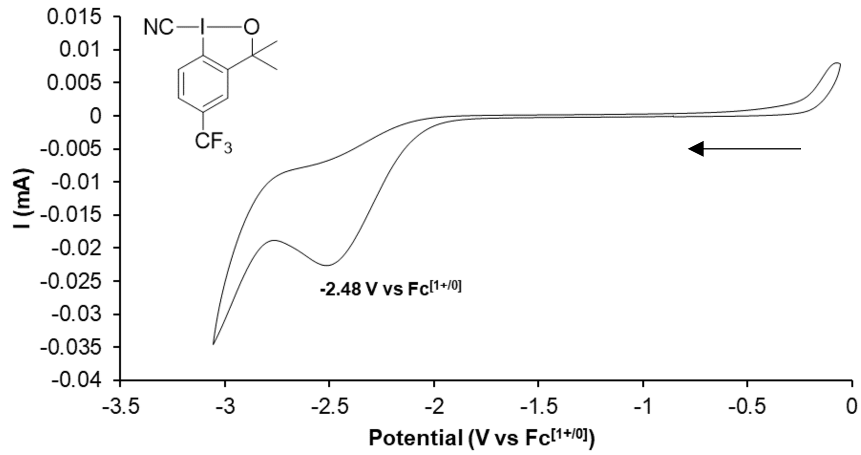

Figure SI-27. Cyclic voltammogram of 1-cyano-3,3-dimethy-(1*H*)-4-trifluromethyl-1,2-benziodoxole (**CDBX**^{CF3}) (3 mM) in THF at 25°C.

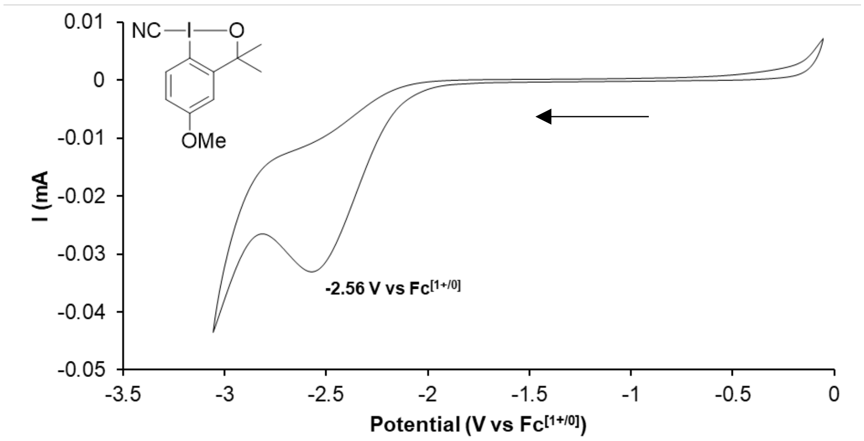

Figure SI-28. Cyclic voltammogram of 1-cyano-3,3-dimethy-(1*H*)-4-methoxy-1,2-benziodoxole (**CDBX**^{OMe}) (3 mM) in THF at 25°C.

Table SI-3. Table of reduction potential in MeCN

I(III) reagent		E_{pc} (V vs. Fc+/Fc)[a]	E_{pc} (V vs. SCE)
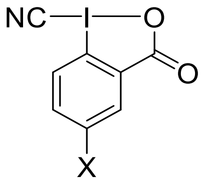	X = H	-1.20	-0.82
	X = F	-1.20	-0.82
	X = OMe	-1.19	-0.81
	X = CF_3	-1.28	-0.90
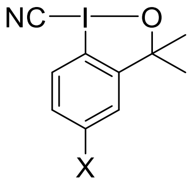	X = H	-1.84	-1.46
	X = F	-1.76	-1.38
	X = OMe	-1.90	-1.52
	X = CF_3	-1.85	-1.47

[a] Cyclic Voltammetry performed at 3 mM of the cyano λ^3-iodane reagent in MeCN at 25°C using as electrolyte $n$$Bu_4NPF_6$ (0.1M), at scan rate of 50 mV/s and using ferrocene as internal reference. A three-electrode electrochemical cell has been used: glassy carbon as working electrode, platinum wire as auxiliary electrode and $Ag/AgNO_3$ (0.01M) as reference electrode.

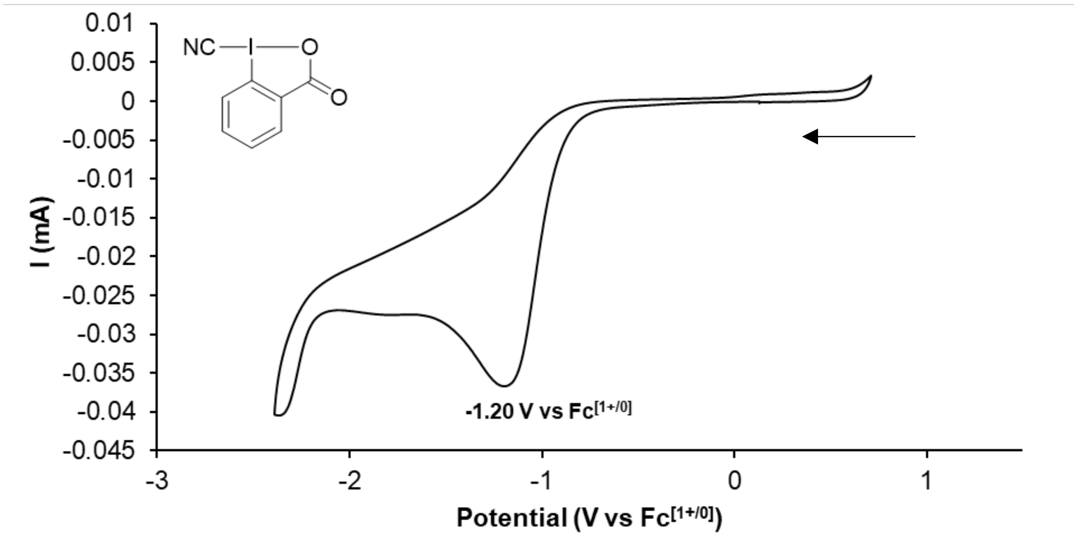

Figure SI-29. Cyclic voltammogram of 1-cyano-1,2-benziodoxol-3-(1*H*)-one (**CBX**) (3 mM) in MeCN at 25°C.

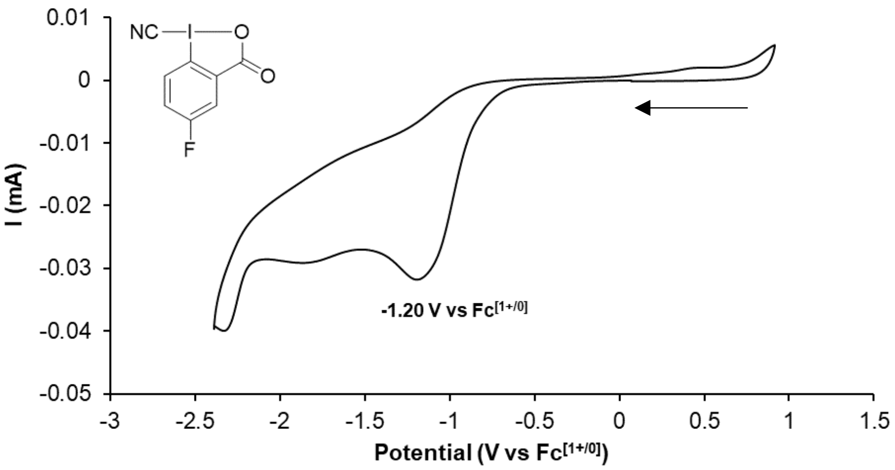

Figure SI-30. Cyclic voltammogram of 4-Fluor-1-Cyano-1,2-benziodoxol-3-(1*H*)-one (**CBX^{F}**) (3 mM) in MeCN at 25°C.

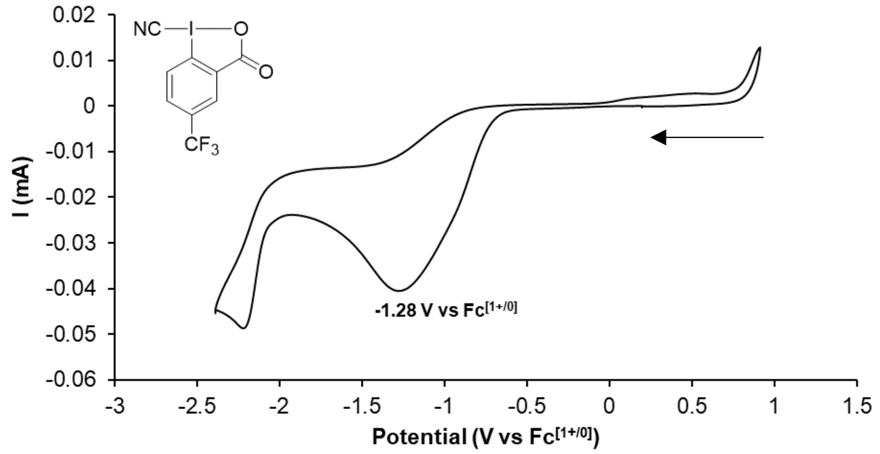

Figure SI-31. Cyclic voltammogram of 4-Trifluoromethyl-1-Cyano-1,2-benziodoxol-3-(1*H*)-one (**CBX^{CF3}**) (3 mM) in MeCN at 25°C.

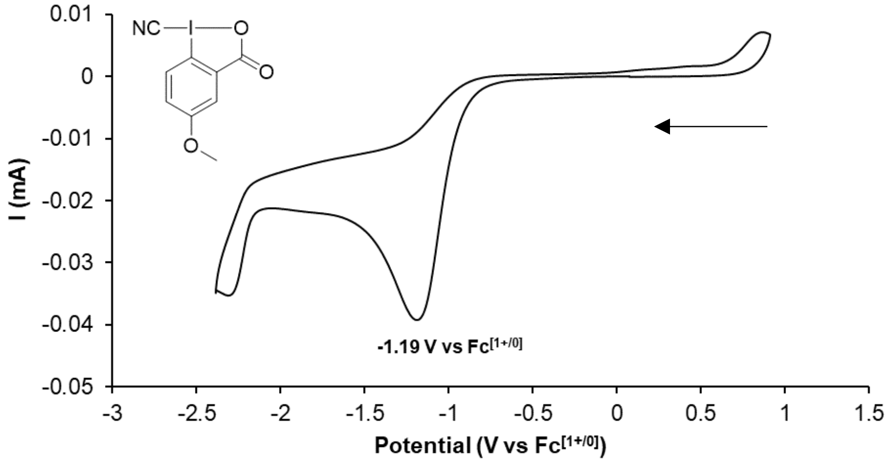

Figure SI-32. Cyclic voltammogram of 4-Methoxy-1-Cyano-1,2-benziodoxol-3-(1*H*)-one (**CBX^{OMe}**) (3 mM) in MeCN at 25ºC.

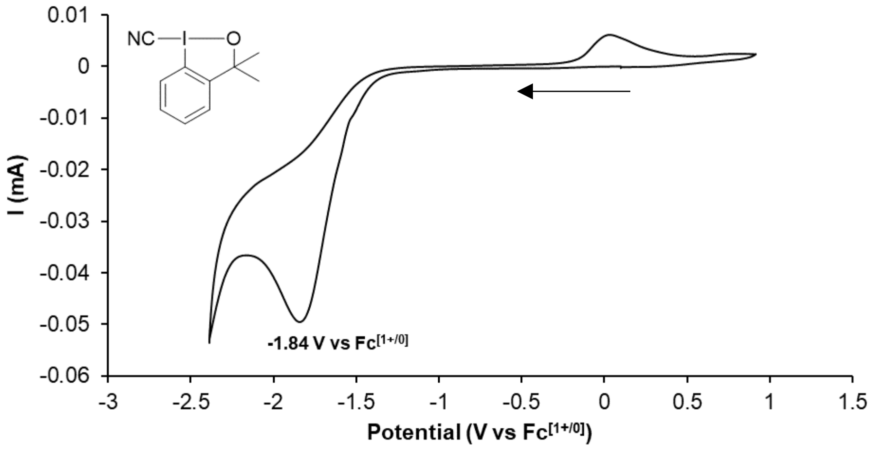

Figure SI-33. Cyclic voltammogram of 1-cyano-3,3-dimethy-(1*H*)-1,2-benziodoxole (**CDBX**) (3 mM) in MeCN at 25ºC.

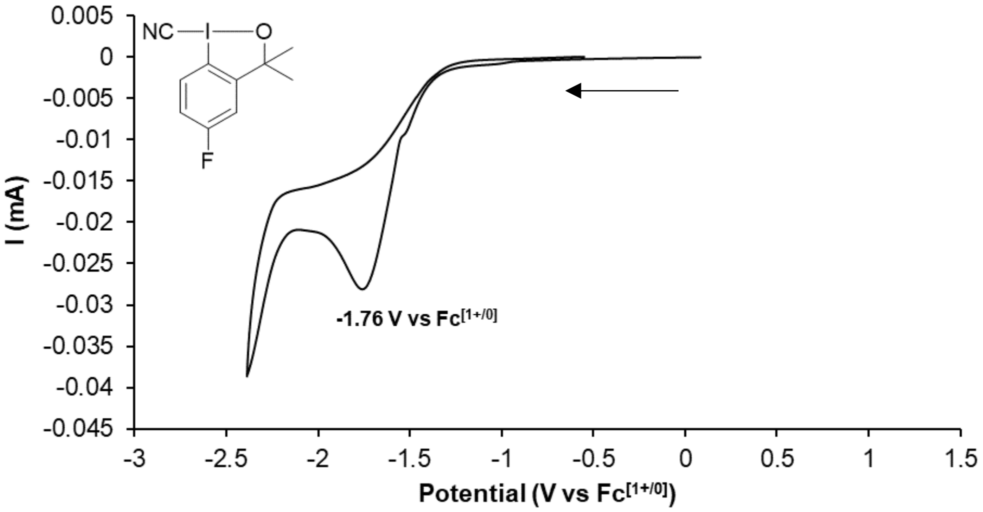

Figure SI-34. Cyclic voltammogram of 1-cyano-3,3-dimethy-(1*H*)-4-fluoro-1,2-benziodoxole (**CDBX^F**) (3 mM) in MeCN at 25ºC.

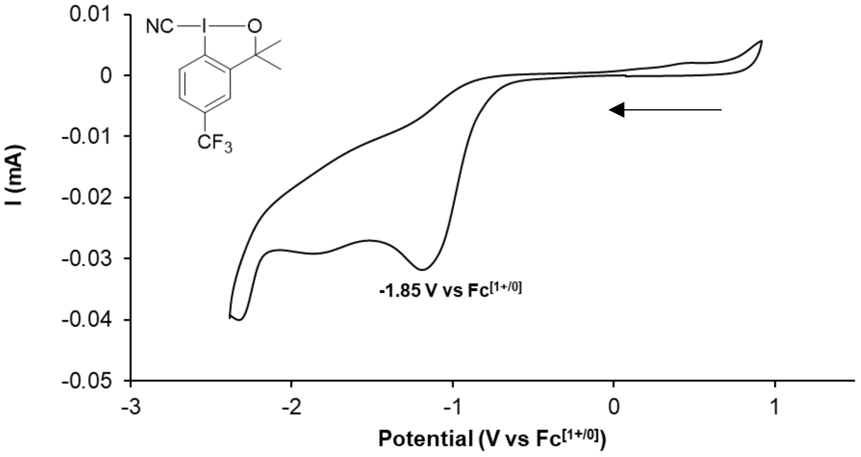

Figure SI-35. Cyclic voltammogram of 1-cyano-3,3-dimethy-(1*H*)-4-trifluromethyl-1,2-benziodoxole (**CDBX^{CF3}**) (3 mM) in MeCN at 25°C.

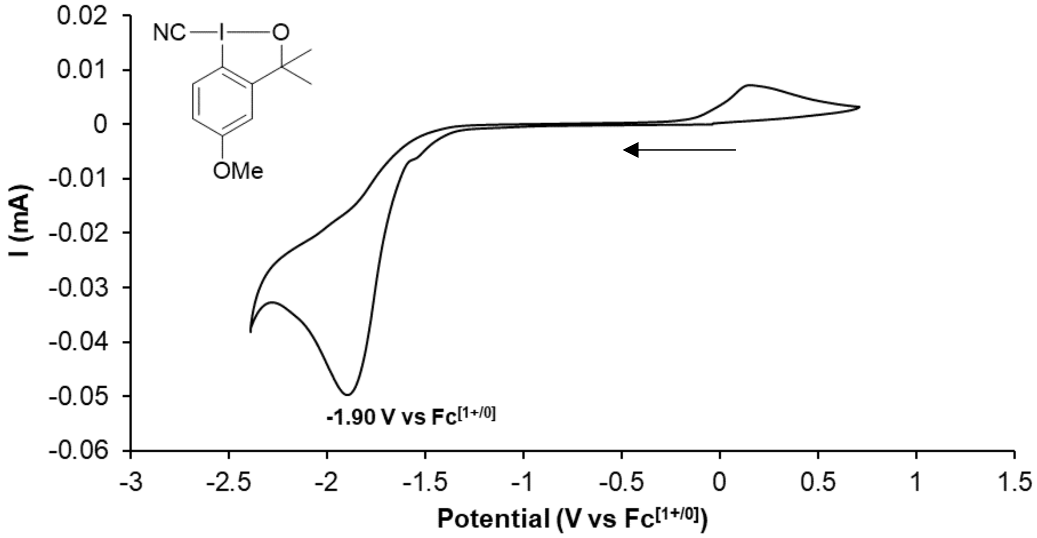

Figure SI-36. Cyclic voltammogram of 1-Cyano-3,3-dimethy-(1*H*)-4-Methoxy-1,2-benziodoxole (**CDBX^{OMe}**) (3 mM) in MeCN at 25°C.

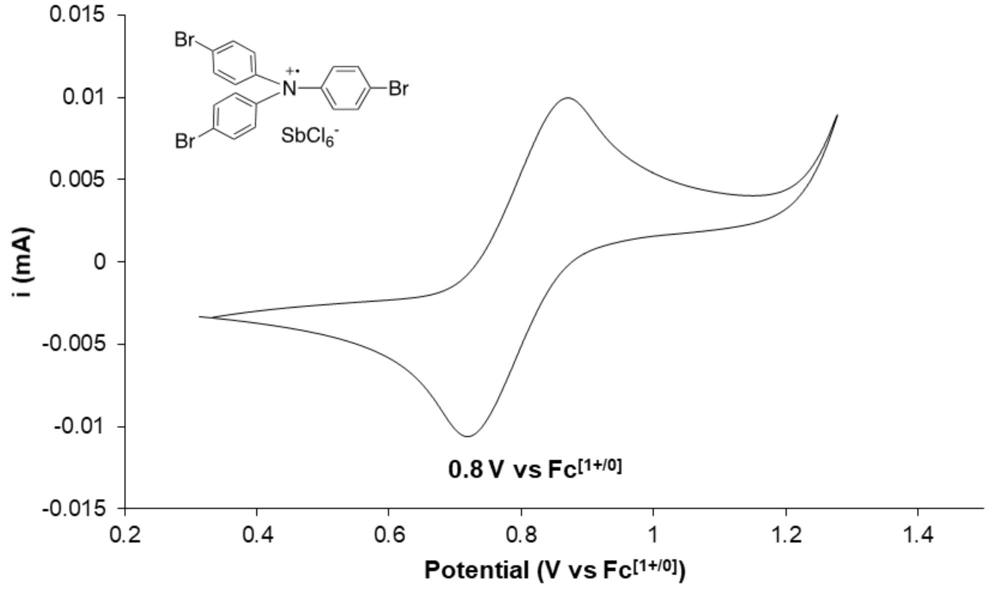

Figure SI-37. Cyclic voltammogram of Tris(4-bromophenyl)ammoniumyl hexachloroantimonate (3 mM) in THF at 25°C.

1.7.2. Iron complexes

Cyclic voltammetry was measured at -20°C in THF solution of iron complex (3 mM) containing 0.1 M of [TBA][PF_6]. Blank solution of TBAP with ferrocene (Fc) was measured as an internal standard. The potential is referenced to the ferrocene/ferrocenium couple. The best scan rate was set at 50 mV^{-1} s^{-1}.

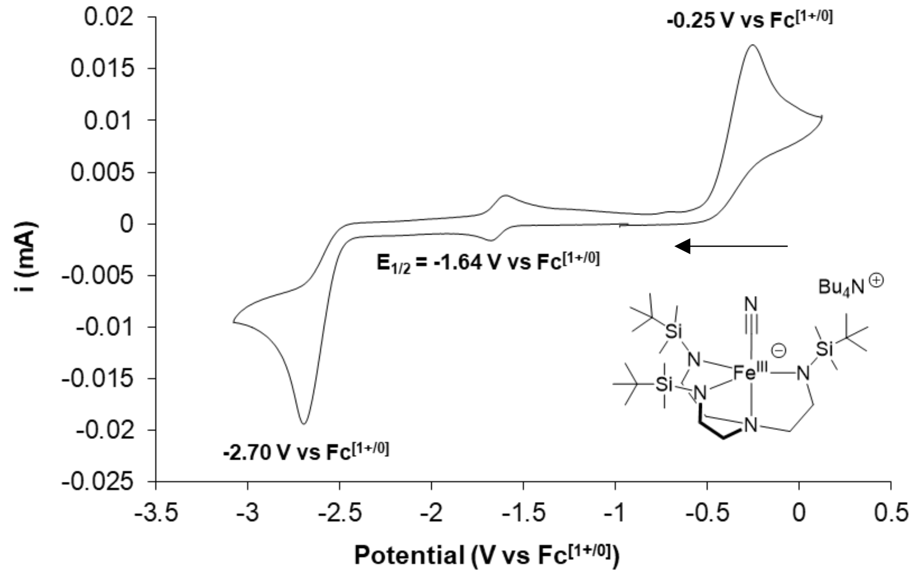

Figure SI-38. Cyclic voltammogram of [(N_3N')Fe^{III}(CN)($^nBu_4N$)] (**2-Bu₄N**, 3 mM) in THF at -20°C.

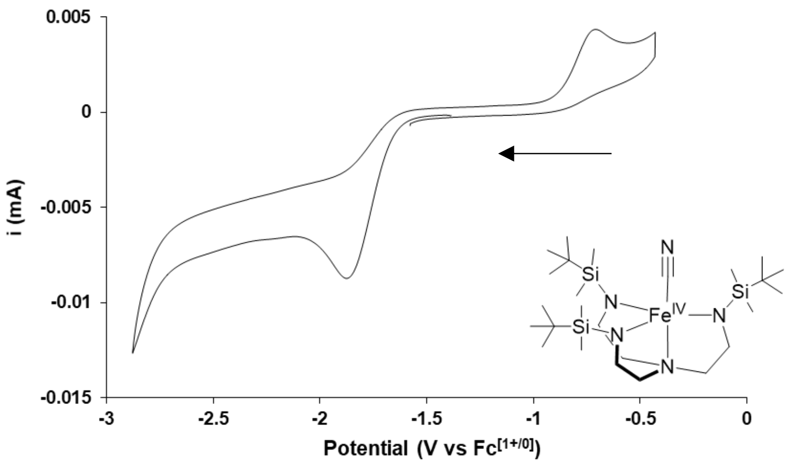

Figure SI-39. Cyclic voltammogram of [(N_3N')Fe^{IV}CN] (**4**, 3 mM) in THF at -20 °C.

2. X-Ray Data Tables

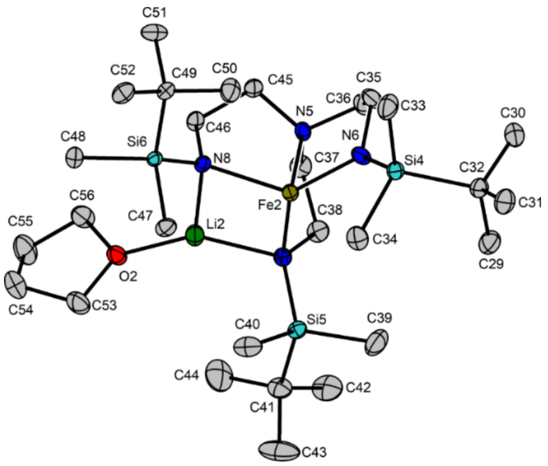

Figure SI-40. Fragment of the X-ray crystal structure for [(N_3N')Fe^{II}Li(THF)] (**1**). Only one molecule in the asymmetric unit is shown. The hydrogen atoms are not shown for clarity. Displacement ellipsoids are shown at 50 % probability level at 100 K.

Table SI-4. Selected crystallographic data and details of the structure determination for **1**. (CCDC 2144076)

Identification code	CS02159
Empirical formula	$C_{40}H_{93}ClFeN_5Si_3$
Molar mass / g·mol^{-1}	620.90
Space group (No.)	$P\bar{1}$ (2)
a / Å	18.2055(8)
b / Å	19.4985(9)
c / Å	19.5981(9)
α / °	112.3170(10)
β / °	104.027(2)
γ / °	109.5300(10)
volume / Å^3	5494.5(4)
Z	6
$\rho_{calc.}$ / g·cm^{-3}	1.126
μ / mm^{-1}	0.535
Color	yellow
Crystal habitus	block
Crystal size / mm^3	0.681 x 0.388 x 0.332

T / K	100		
λ / Å	0.71073 (Mo-K_{α})		
θ range / °	2.142 to 29.231		
Range of Miller indices	$-24 \leq h \leq 25$	$-25 \leq k \leq 26$	$-26 \leq l \leq 26$
Absorption correction	multi-scan		
T_{min}, T_{max}	0.7276, 0.9760		
R_{int}, R_{σ}	0.0398, 0.0369		
Completeness of the data set	0.999		
No. of measured reflections	157883		
No. of independent reflections	29775		
No. of parameters	1091		
No. of restrains	0		
S (all data)	1.032		
$R(F)$ ($I \geq 2\sigma(I)$, all data)	0.0391, 0.0608		
$wR(F^2)$ ($I \geq 2\sigma(I)$, all data)	0.0879, 0.0959		
Extinction coefficient	not refined		
$\Delta\rho_{max}$, $\Delta\rho_{min}$ / e·Å^{-3}	1.302, –0.514		

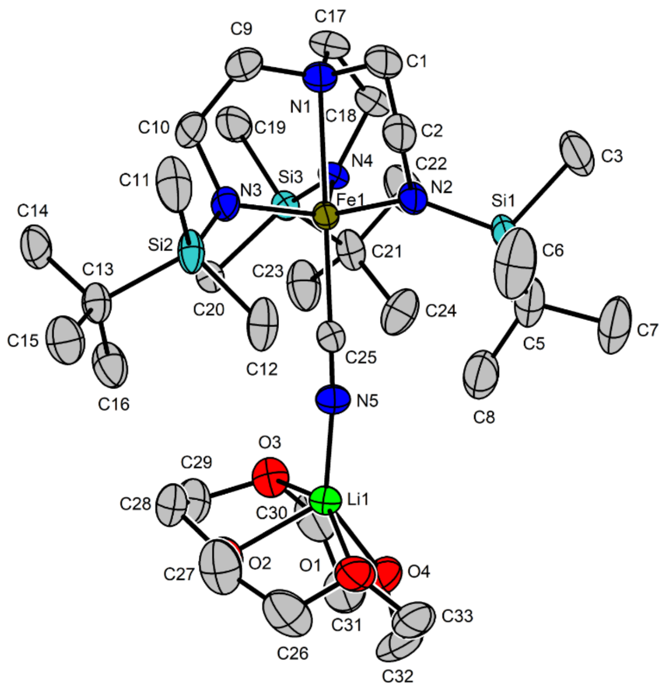

Figure SI-41. Fragment of the X-ray crystal structure of [(N_3N')Fe^{III}(CN)Li(12-c-4)] (**2-Li**^{crown}). The diethyl ether solvent molecule and the hydrogen atoms are not shown. Displacement ellipsoids are shown at 50 % probability level at 100 K.

Table SI-5. Selected crystallographic data and details of the structure determination for **2-Li**^{crown}. (CCDC 2144072)

Identification code	CS05025
Empirical formula	$C_{70}H_{156}Fe_2Li_2N_{10}O_9Si_6$
Molar mass / g·mol^{-1}	1576.16
Space group (No.)	*C*2/*c* (15)
a / Å	28.4026(16)
b / Å	12.8782(7)
c / Å	25.5226(14)
β / °	95.319(2)
V / Å^3	9295.3(9)
Z	4
$\rho_{calc.}$ / g·cm^{-3}	1.126
μ / mm^{-1}	0.440
Color	red
Crystal habitus	plate
Crystal size / mm^3	0.266 x 0.218 x 0.072

T / K	100
λ / Å	0.71073 (Mo-K$_\alpha$)
θ range / °	1.941 to 28.316
Range of Miller indices	$-37 \leq h \leq 37$
	$-17 \leq k \leq 17$
	$-34 \leq l \leq 34$
Absorption correction	multi-scan and numerical
T_{min}, T_{max}	0.8812, 0.9838
R_{int}, R_σ	0.0488, 0.0371
Completeness of the data set	0.999
No. of measured reflections	77286
No. of independent reflections	11553
No. of parameters	486
No. of restraints	48
S (all data)	1.092
$R(F)$ ($I \geq 2\sigma(I)$, all data)	0.0558, 0.0768
$wR(F^2)$ ($I \geq 2\sigma(I)$, all data)	0.1188, 0.1279
Extinction coefficient	not refined
$\Delta\rho_{max}$, $\Delta\rho_{min}$ / e·Å^{-3}	0.564, –0.562

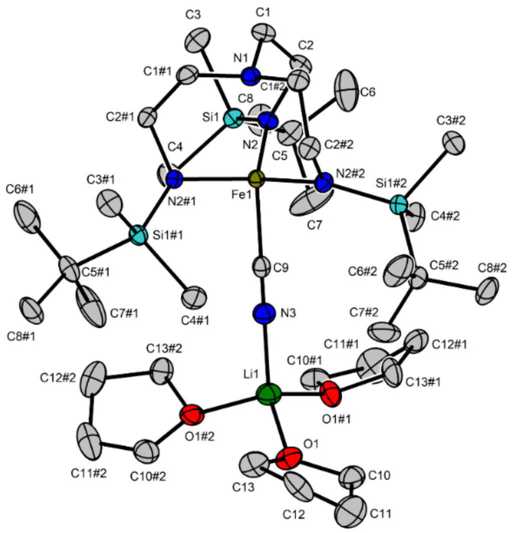

Figure SI-42. Fragment of the X-ray crystal structure of [(N$_3$N')Fe^{III}(CN)Li(THF)$_3$] (**2-Li^{THF}**). The hydrogen atoms are not shown. Only one disordered species of the THF groups is shown. Displacement ellipsoids are shown at 50 % probability level at 100 K. Symmetry operations used to generate equivalent atoms: #1: 1/2–y, 1–z, 1/2+x; #2: –1/2+z, 1/2–x, 1–y.

Table SI-6. Selected crystallographic data and details of the structure determination for **2-Li^{THF}**. (CCDC 2144073)

Identification code	CS03015
Empirical formula	$C_{37}H_{81}FeLiN_5O_3Si_3$
Molar mass / g·mol^{-1}	791.12
Space group (No.)	$Pa\overline{3}$ (205)
a / Å	21.1030(5)
volume / Å^3	9397.9(7)
Z	8
$\rho_{calc.}$ / g·cm^{-3}	1.118
μ / mm^{-1}	0.434
Color	dark violet
Crystal habitus	block
Crystal size / mm^3	0.116 x 0.107 x 0.095
T / K	100
λ / Å	0.71073 (Mo-K$_\alpha$)
θ range / °	1.930 to 26.396
Range of Miller indices	$-22 \le h \le 26$ $-25 \le k \le 22$ $-26 \le l \le 26$
Absorption correction	multi-scan and numerical
T_{min}, T_{max}	0.9449, 0.9752
R_{int}, R_σ	0.0711, 0.0273
Completeness of the data set	1.000
No. of measured reflections	67087
No. of independent reflections	3221
No. of parameters	202
No. of restrains	184
S (all data)	1.088
$R(F)$ ($I \ge 2\sigma(I)$, all data)	0.0476, 0.0658
$wR(F^2)$ ($I \ge 2\sigma(I)$, all data)	0.0968, 0.1036
Extinction coefficient	not refined
$\Delta\rho_{max}$, $\Delta\rho_{min}$ / e·Å^{-3}	0.313, –0.388

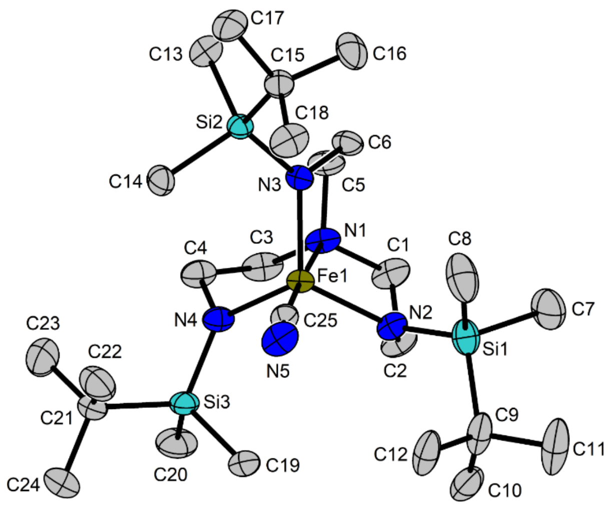

Figure SI-43. Fragment of the X-ray crystal structure of [(N$_3$N')Fe^{III}(CN)(^{n}Bu$_4$N)] (**2-Bu$_4$N**). One out of three symmetry independent anions is shown. The cation and hydrogen atoms are omitted for clarity. Displacement ellipsoids are shown at 50 % probability level at 100 K.

Table SI-7. Selected crystallographic data and details of the structure determination for **2-Bu$_4$N**. (CCDC 2144077)

Identification code	CS02124
Empirical formula	$C_{41}H_{93}FeN_6Si_3$
Molar mass / g·mol^{-1}	810.33
Space group (No.)	$P2_1/c$ (14)
a / Å	11.48680(10)
b / Å	20.29920(10)
c / Å	65.8964(5)
β / °	94.4440(10)
volume / Å^3	15319.05(19)
Z	12
$\rho_{calc.}$ / g·cm^{-3}	1.054
μ / mm^{-1}	3.266
Color	red
Crystal habitus	plate
Crystal size / mm^3	0.292 x 0.142 x 0.051
T / K	100
λ / Å	1.54186 (Cu-K$_\alpha$)

θ range / °	3.860 to 72.128
Range of Miller indices	$-9 \le h \le 14$ $-22 \le k \le 25$ $-81 \le l \le 81$
Absorption correction	multi-scan and numerical
T_{min}, T_{max}	0.1311, 0.5816
R_{int}, R_{σ}	0.1311, 0.5816
Completeness of the data set	0.964
No. of measured reflections	143134
No. of independent reflections	28777
No. of parameters	1475
No. of restrains	0
S (all data)	1.038
$R(F)$ ($I \ge 2\sigma(I)$, all data)	0.0466, 0.0578
$wR(F^2)$ ($I \ge 2\sigma(I)$, all data)	0.1151, 0.1200
Extinction coefficient	not refined
$\Delta\rho_{max}$, $\Delta\rho_{min}$ / e·Å^{-3}	0.624, –0.471

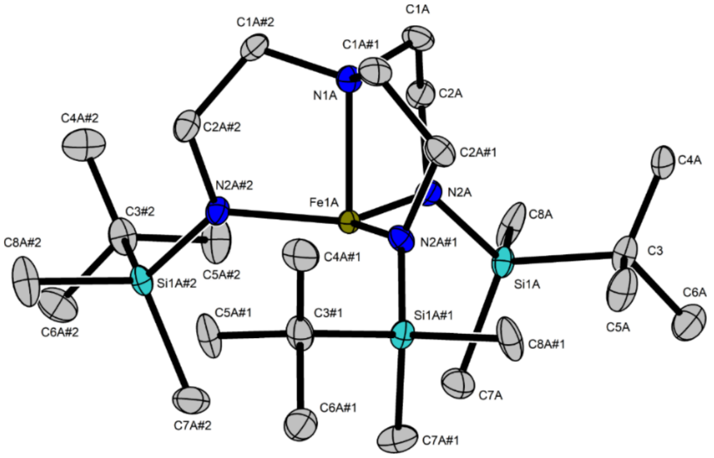

Figure SI-44. Fragment of the X-ray crystal structure of [(N_3N')Fe^{III} (**3**). The hydrogen atoms are not shown. Only one disordered species is shown. Displacement ellipsoids are shown at 50 % probability level at 100 K. Symmetry operations used for the generation of equivalent atoms: #1: $-x+y$, $1-x$, z; #2: $1-y$, $1+x-y$, z.

Table SI-8. Selected crystallographic data and details of the structure determination for **3**. (CCDC 2144075)

Identification code	CS01138b
Empirical formula	$C_{24}H_{57}FeN_4Si_3$
Molar mass / g·mol^{-1}	541.85
Space group (No.)	$P6_3$ (No. 173)
a / Å	13.1630(6)
b / Å	10.7894(7)
volume / Å^3	1618.97(18)
Z	2
$\rho_{calc.}$ / g·cm^{-3}	1.112
μ / mm^{-1}	0.594
Color	dark red
Crystal habitus	block
Crystal size / mm^3	0.216 x 0.208 x 0.102
T / K	100
λ / Å	0.71073 (Mo-K$_\alpha$)
θ range / °	2.599 to 30.562
Range of Miller indices	$-18 \le h \le 18$ $-18 \le k \le 18$ $-15 \le l \le 15$
Absorption correction	multi-scan and numerical
T_{min}, T_{max}	0.8675, 0.9636
R_{int}, R_σ	0.0498, 0.0235
Completeness of the data set	0.999
No. of measured reflections	77734
No. of independent reflections	3324
No. of parameters	191
No. of restrains	1
S (all data)	1.189
$R(F)$ ($I \ge 2\sigma(I)$, all data)	0.0324, 0.0373
$wR(F^2)$ ($I \ge 2\sigma(I)$, all data)	0.0733, 0.0749
Extinction coefficient	0.0063(17)
Volume fraction of the inverted twin component (BASF)	0.12(3)

$\Delta\rho_{max}$, $\Delta\rho_{min}$ / e·Å^{-3}	0.329, –0.259

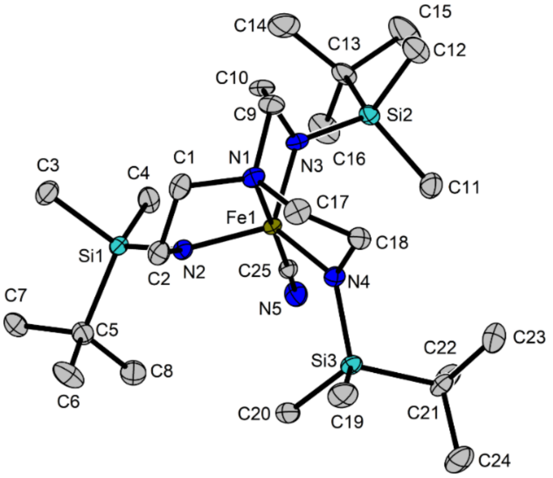

Figure SI-45. Fragment of the X-ray crystal structure of [(N_3N')Fe^{IV}(CN)] (**4**). The hydrogen atoms are not shown. Displacement ellipsoids are shown at 50 % probability level at 100 K. One of the four crystallographically independent molecules is shown.

Table SI-9. Selected crystallographic data and details of the structure determination for **4**. (CCDC X2144074)

Identification code	CS02032
Empirical formula	$C_{25}H_{57}FeN_5Si_3$
Molar mass / g·mol^{-1}	567.87
Space group (No.)	$P\bar{1}$ (2)
a / Å	17.937(5)
b / Å	19.775(5)
c / Å	19.899(5)
α / °	108.543(8)
β / °	91.801(9)
γ / °	91.606(9)
volume / Å^3	6683(3)
Z	8
$\rho_{calc.}$ / g·cm^{-3}	1.129
μ / mm^{-1}	0.579
Color	black
Crystal habitus	block
Crystal size / mm^3	0.167 x 0.143 x 0.051

T / K		100	
λ / Å		0.71073 (Mo-K_α)	
θ range / °		2.086 to 27.269	
Range of Miller indices	$-22 \le h \le 23$	$-25 \le k \le 25$	$-25 \le l \le 25$
Absorption correction		multi-scan	
T_{min}, T_{max}		0.9129, 1.0000	
R_{int}, R_σ		0.0647, 0.0688	
Completeness of the data set		0.997	
No. of measured reflections		129544	
No. of independent reflections		29700	
No. of parameters		1287	
No. of restrains		0	
S (all data)		1.061	
$R(F)$ ($I \ge 2\sigma(I)$, all data)		0.0557, 0.0811	
$wR(F^2)$ ($I \ge 2\sigma(I)$, all data)		0.1154, 0.1275	
Extinction coefficient		0.00073(7)	
Volume fraction of the 2nd twin component		0.3201(6)	
$\Delta\rho_{max}$, $\Delta\rho_{min}$ / e·Å^{-3}		0.715, –1.079	

3. DFT Calculations

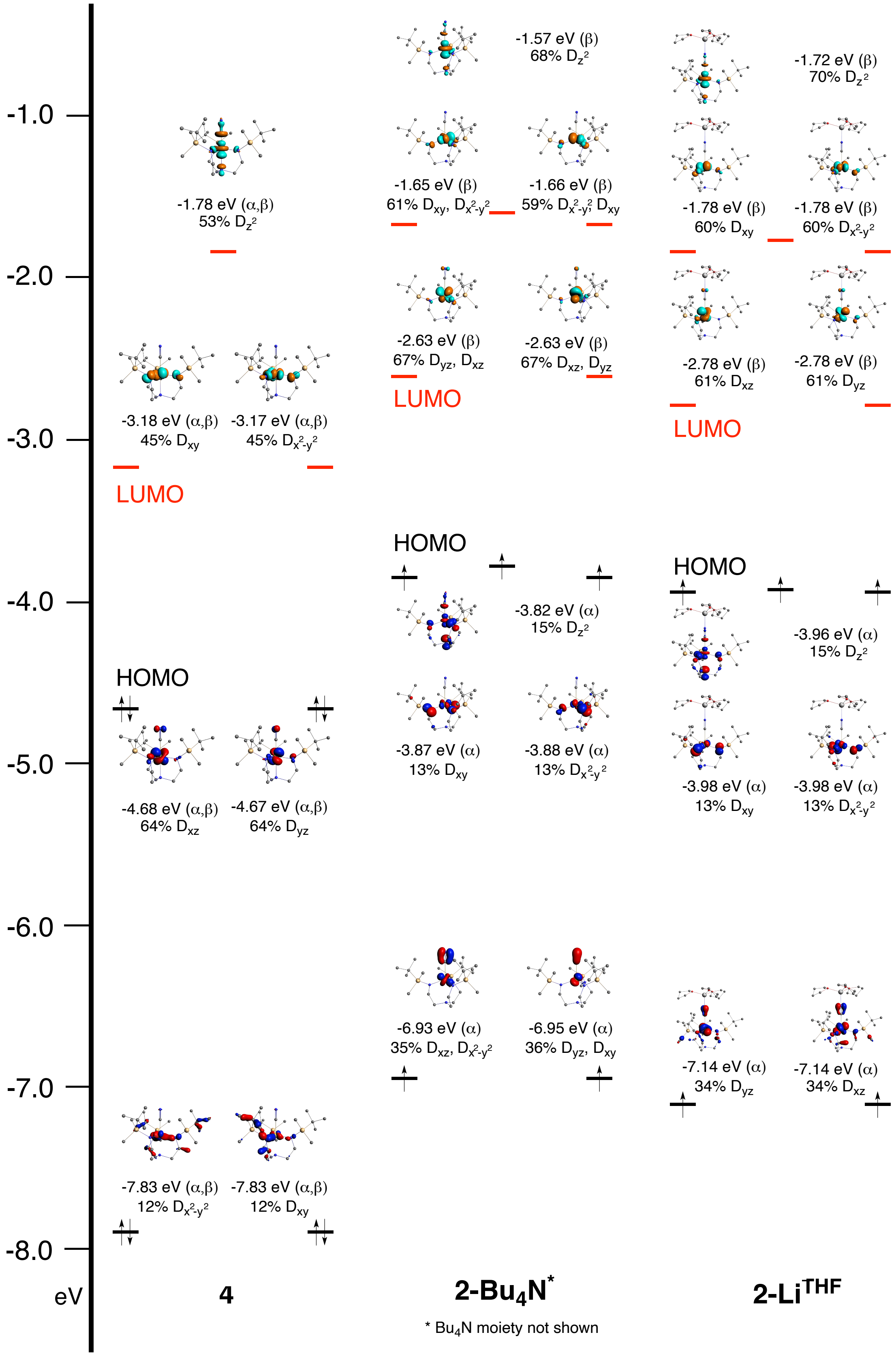

Figure SI-46. Molecular orbital scheme for compounds **4**, **2-Bu$_4$N** and **2-Li^{THF}**.

Table SI-10. Molecular orbitals for compound **4**, which contain contributions from iron.

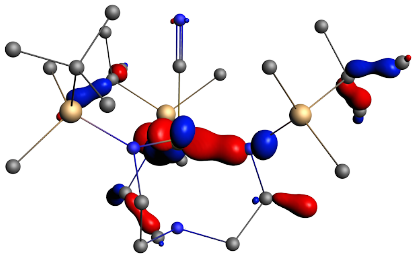	**MO 101(αβ)** (occupied)<br><br>-7.83 eV<br><br>12.17% D(xy)
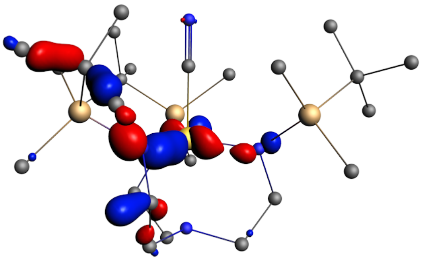	**MO 102(αβ)** (occupied)<br><br>-7.83 eV<br><br>12.27% D(x^2-y^2)
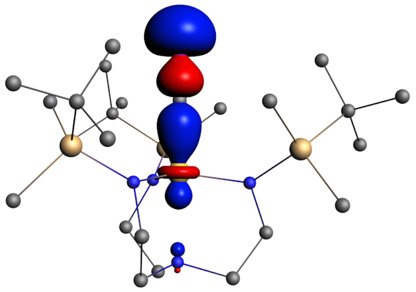	**MO 103(αβ)** (occupied)<br><br>-7.70 eV<br><br>9.17% D(z^2)
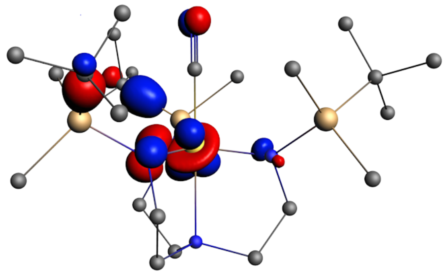	**MO 113(αβ)** (occupied)<br><br>-5.99 eV<br><br>10.95% D(xz)
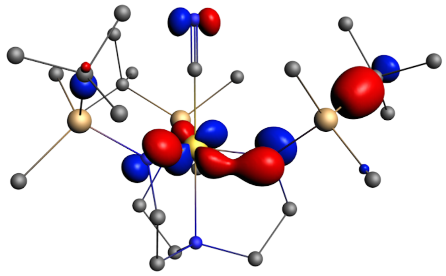	**MO 114(αβ)** (occupied)<br><br>-5.99 eV<br><br>10.93% D(yz)

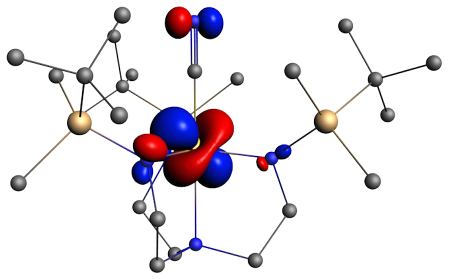	**MO 116(αβ)** (occupied)<br><br>-4.68 eV<br><br>52.22% D(xz)<br>11.75% D(yz)
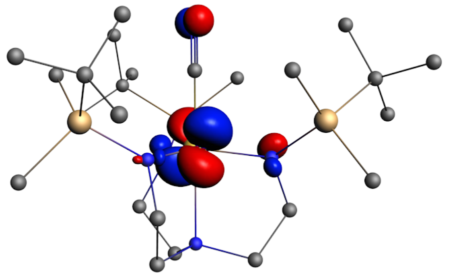	**MO 117(αβ)** (occupied)<br><br>-4.67 eV<br><br>52.29% D(yz)<br>11.83% D(xz)
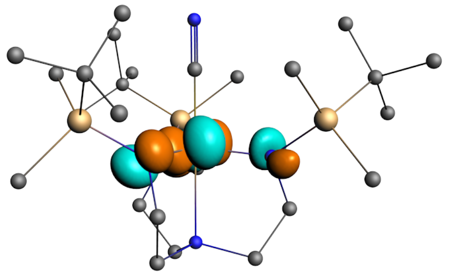	MO 118(αβ) (unoccupied)<br><br>-3.18 eV<br><br>45.06% D(xy)
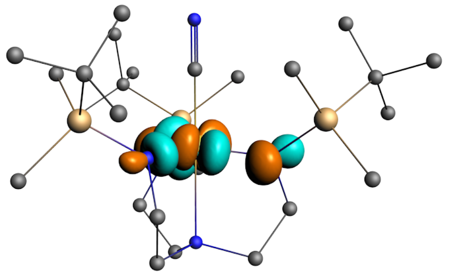	MO 119(αβ) (unoccupied)<br><br>-3.17 eV<br><br>45.13% D(x^2-y^2)
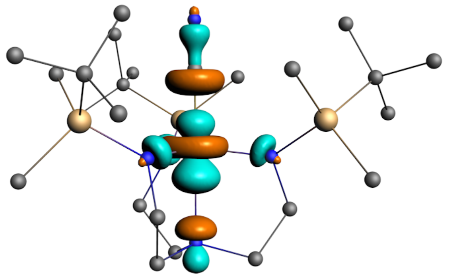	MO 120(αβ) (unoccupied)<br><br>-1.78 eV<br><br>52.66% D(z^2)

(isosurface 0.07 a.u.)

Table SI-11. Molecular orbitals for compound **2-Bu₄N**, which contain contributions from iron.

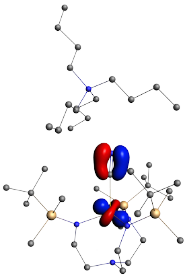	**MO 158(α)** (occupied)<br><br>-6.95 eV<br><br>18.93% D(yz)<br>16.80% D(xy)<br>5.08% D(xz)
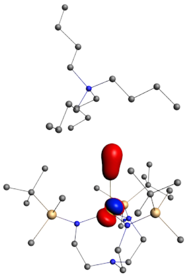	**MO 159(α)** (occupied)<br><br>-6.93 eV<br><br>20.16% D(xz)<br>14.53% D(x^2-y^2)<br>6.55% D(yz)
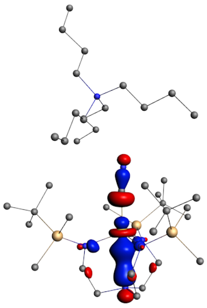	**MO 166(α)** (occupied)<br><br>-5.32 eV<br><br>20.23% D(z^2)
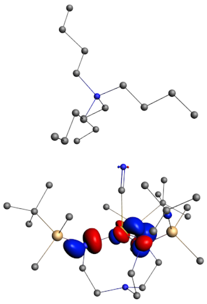	**MO 167(α)** (occupied)<br><br>-4.90 eV<br><br>10.29% D(xy)<br>5.75% D(xz)

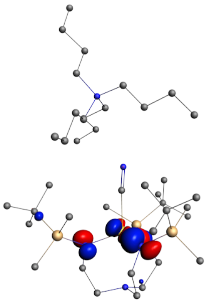	**MO 168(α)** (occupied)<br><br>-4.89 eV<br><br>11.08% D(x^2-y^2)
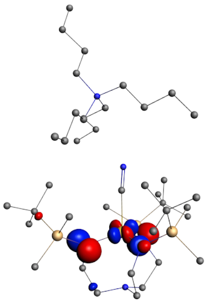	**MO 170(α)** (occupied)<br><br>-3.88 eV<br><br>13.43% D(x^2-y^2)
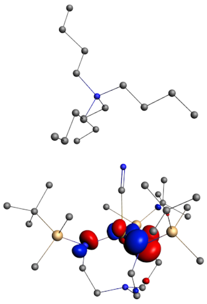	**MO 171(α)** (occupied)<br><br>-3.87 eV<br><br>13.04% D(xy)
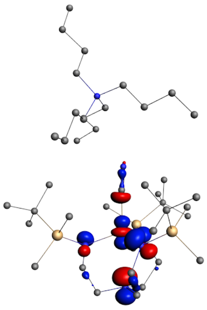	**MO 172(α)** (occupied)<br><br>-3.82 eV<br><br>14.90% D(z^2)
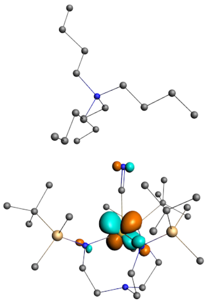	MO 168(β) (unoccupied)<br><br>-2.63 eV<br><br>45.91% D(xz)<br>20.60% D(yz)<br>8.62% D(x^2-y^2)

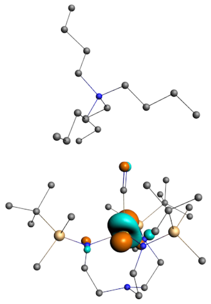	MO 169(β) (unoccupied)<br><br>-2.63 eV<br><br>44.58% D(yz)<br>22.29% D(xz)<br>9.13% D(xy)
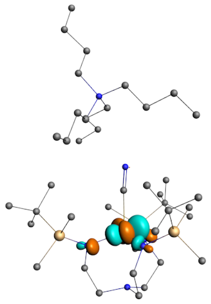	MO 170(β) (unoccupied)<br><br>-1.66 eV<br><br>34.52% D(x^2-y^2)<br>24.33% D(xy)
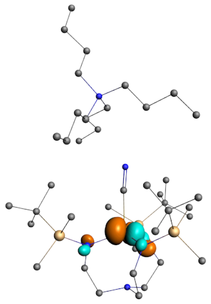	MO 171(β) (unoccupied)<br><br>-1.65 eV<br><br>35.09% D(xy)<br>25.79% D(x^2-y^2)
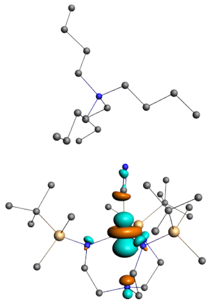	MO 172(β) (unoccupied)<br><br>-1.57 eV<br><br>68.09% D(z^2)

(isosurface 0.07 a.u.)

Table SI-12. Molecular orbitals for compound **2-Li^{THF}**, which contain contributions from iron.

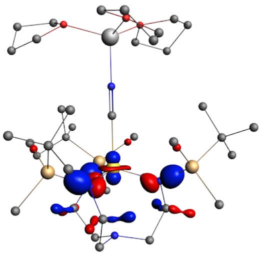	**MO 147(α)** (occupied)<br><br>-7.29 eV<br><br>7.85% D(z^2)
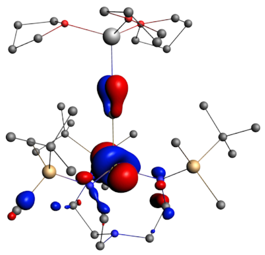	**MO 148(α)** (occupied)<br><br>-7.14 eV<br><br>28.14% D(xy)<br>35.43% D(xz)
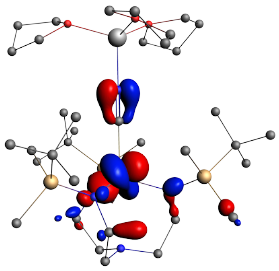	**MO 149(α)** (occupied)<br><br>-7.14 eV<br><br>28.11% D(x^2-y^2)<br>35.34% D(yz)
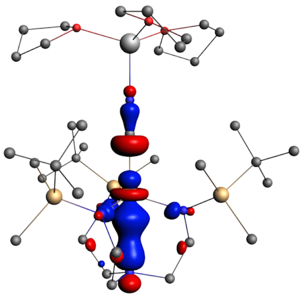	**MO 159(α)** (occupied)<br><br>-5.51 eV<br><br>23.07% D(z^2)
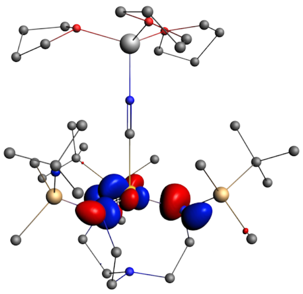	**MO 160(α)** (occupied)<br><br>-5.02 eV<br><br>10.46% D(xy)<br>6.20% D(xz)

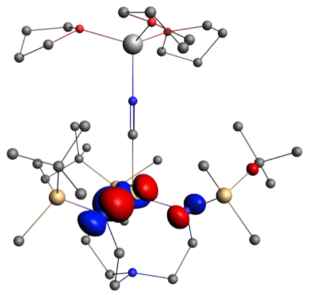	**MO 161(α)** (occupied)<br><br>-5.02 eV<br><br>10.45% D(x^2-y^2)<br>6.20% D(yz)
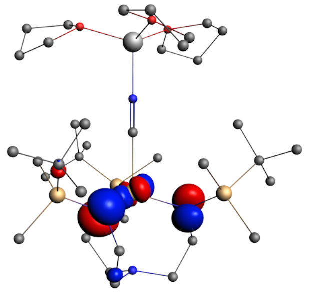	**MO 163(α)** (occupied)<br><br>-3.98 eV<br><br>12.93% D(x^2-y^2)
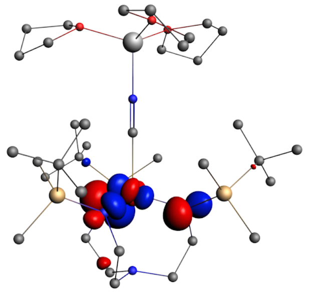	**MO 164(α)** (occupied)<br><br>-3.98 eV<br><br>12.92% D(xy)
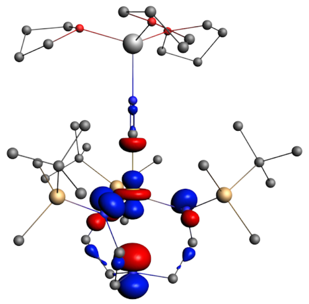	**MO 165(α)** (occupied)<br><br>-3.96 eV<br><br>14.51% D(z^2)
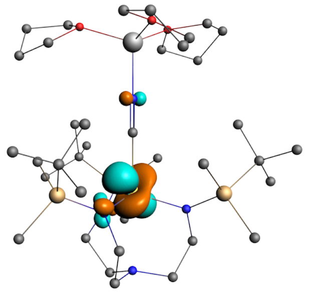	MO 161(β) (unoccupied)<br><br>-2.78 eV<br><br>8.88% D(x^2-y^2)<br>60.85% D(xz)

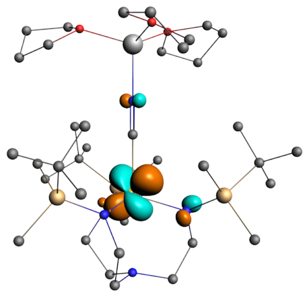	MO 162(β) (unoccupied)<br><br>-2.78 eV<br><br>8.88% D(xy)<br>60.86% D(yz)
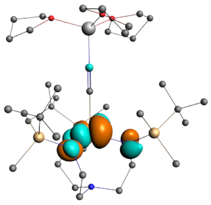	MO 163(β) (unoccupied)<br><br>-1.78 eV<br><br>60.15% D(xy)
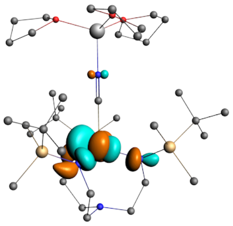	MO 164(β) (unoccupied)<br><br>-1.78 eV<br><br>60.15% D(x^2-y^2)
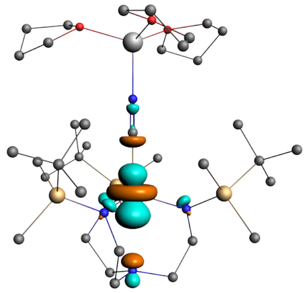	MO 165(β) (unoccupied)<br><br>-1.72 eV<br><br>69.62% D(z^2)

(isosurface 0.07 a.u.)

Redox potentials

For the calculation of the redox potentials we follow the protocol by Namazian, Lin, and Coote,[25] who computed the absolute redox potential of ferrocene/ferrocenium couple in different solvents with several computational methods. The absolute reduction potential at S12g/TZ2P in acetonitrile is then 4.838 eV (4.876 eV directly from Fc vs. Fc^+, and a correction term of -0.0376 eV for the Gibbs free energy of an electron[26] from Fermi-Dirac statistics of the "electron convention"), which is reasonably close to the "experimental" value of 4.980 eV.[25] In THF, the absolute reduction potential is then 4.992 eV at S12g/TZ2P for the Fc/Fc^+ couple.

Table SI-13. Energies and redox potential calculated for **2·Bu₄N**.

		E^{elec} kcal·mol^{-1}	ΔGibbs kcal·mol^{-1}	Gibbs kcal·mol^{-1}	Gibbs eV	V^{red} eV	$V^{red,a}$ eV
^{5}[**2·Bu$_4$N**]$^{-1}$	Fe^{II}	-18446.301	732.126	-17714.175	-1.752	1.714	-3.278
^{6}[**2·Bu$_4$N**]^0	Fe^{III}	-18410.982	737.209	-17673.773	0	3.358	-1.634
^{1}[**2·Bu$_4$N**]^1	Fe^{IV}	-18340.336	744.880	-17595.456	3.396		

a) relative to Fc^+/Fc^0

4. Characterization data

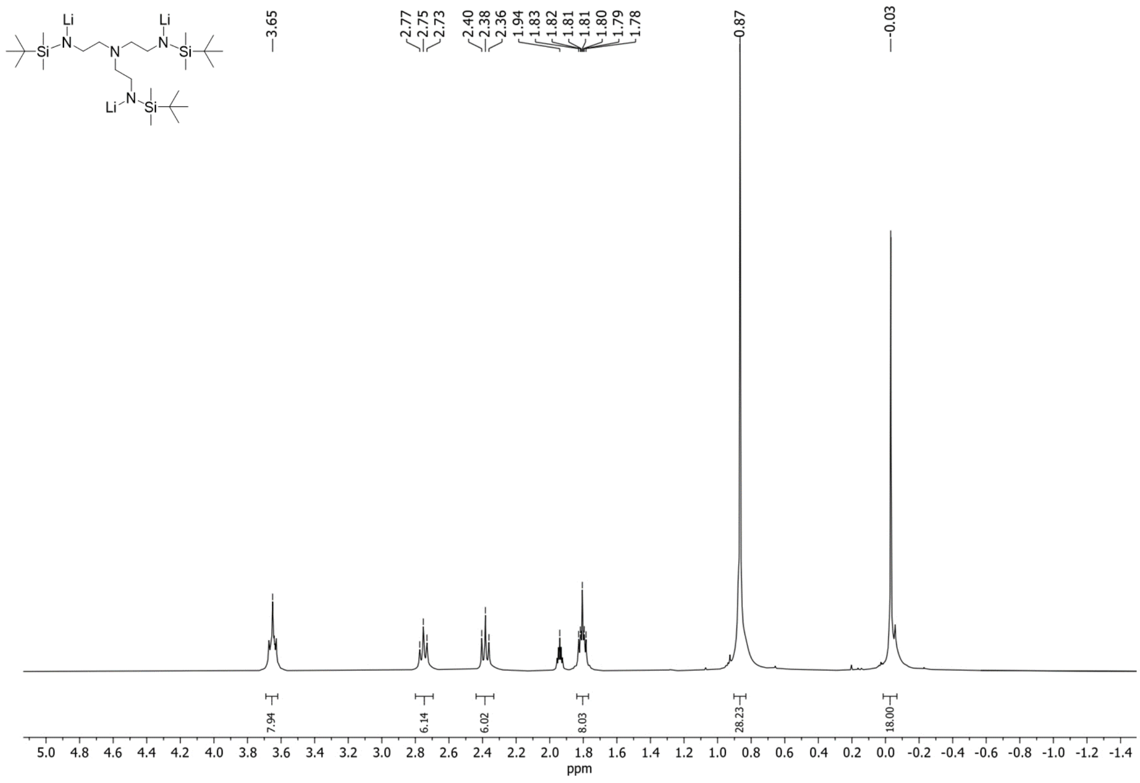

Figure SI-47. ^{1}H-NMR spectrum (300 MHz, CD_3CN, 298K) of $Li_3[N_3N'](THF)_2$.

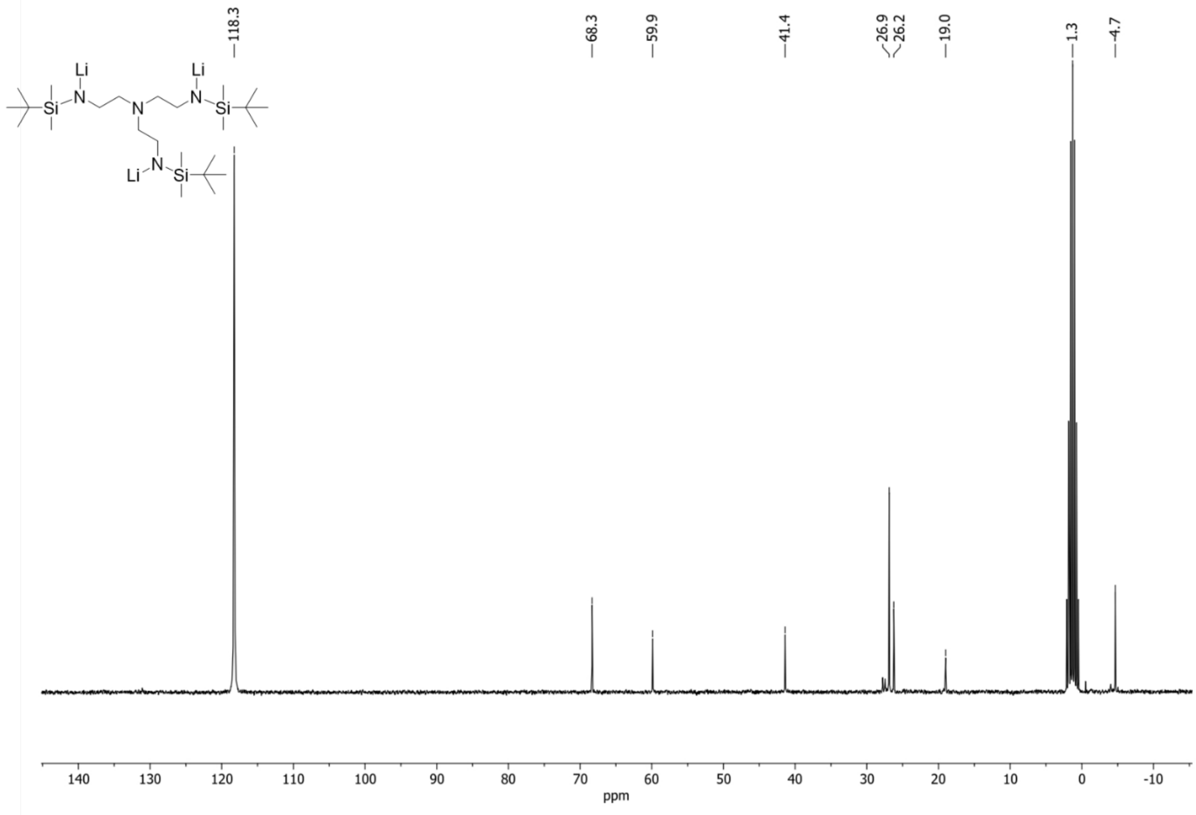

Figure SI-48. ^{13}C-NMR spectrum (75 MHz, CD_3CN, 298K) of $Li_3[N_3N'](THF)_2$.

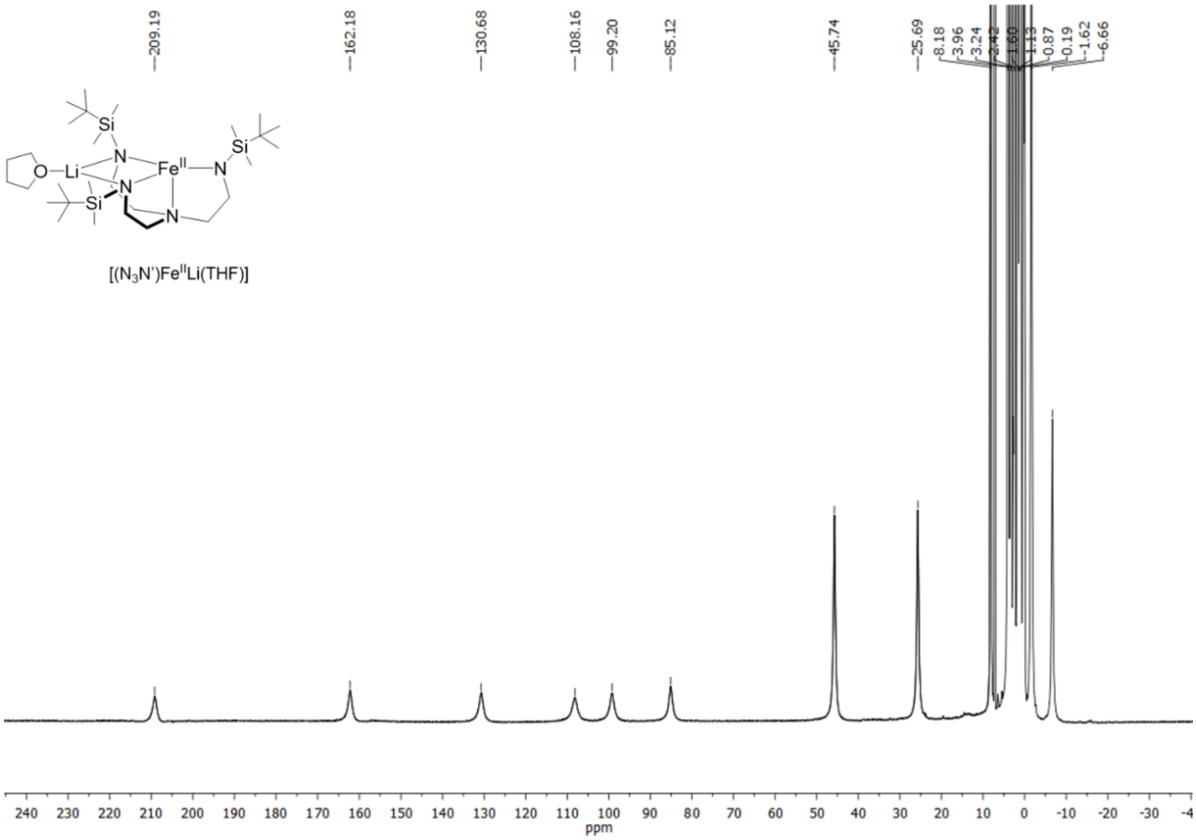

Figure SI-49. ^{1}H-NMR spectrum (500 MHz, C_6D_6, 298K) of [(N_3N')Fe^{II}Li(THF)] (**1**).

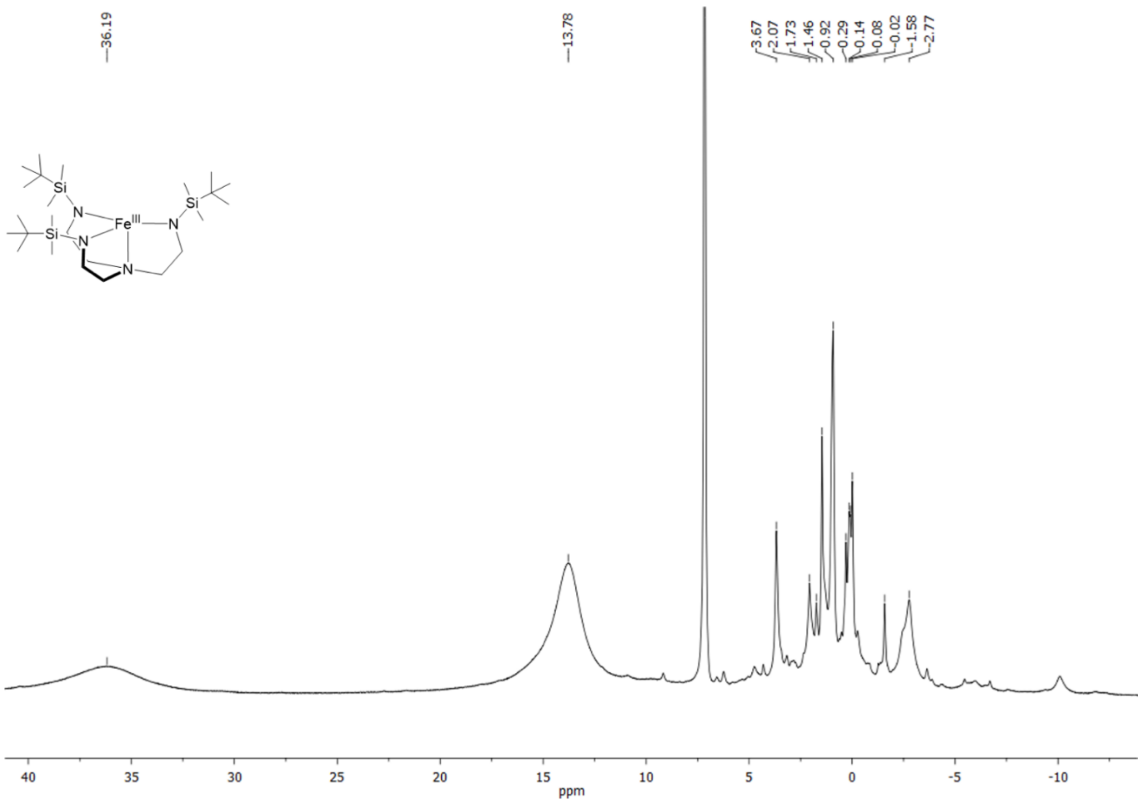

Figure SI-50. ^{1}H-NMR spectrum (500 MHz, C_6D_6, 298K) of [(N_3N')Fe^{III}] (**3**).

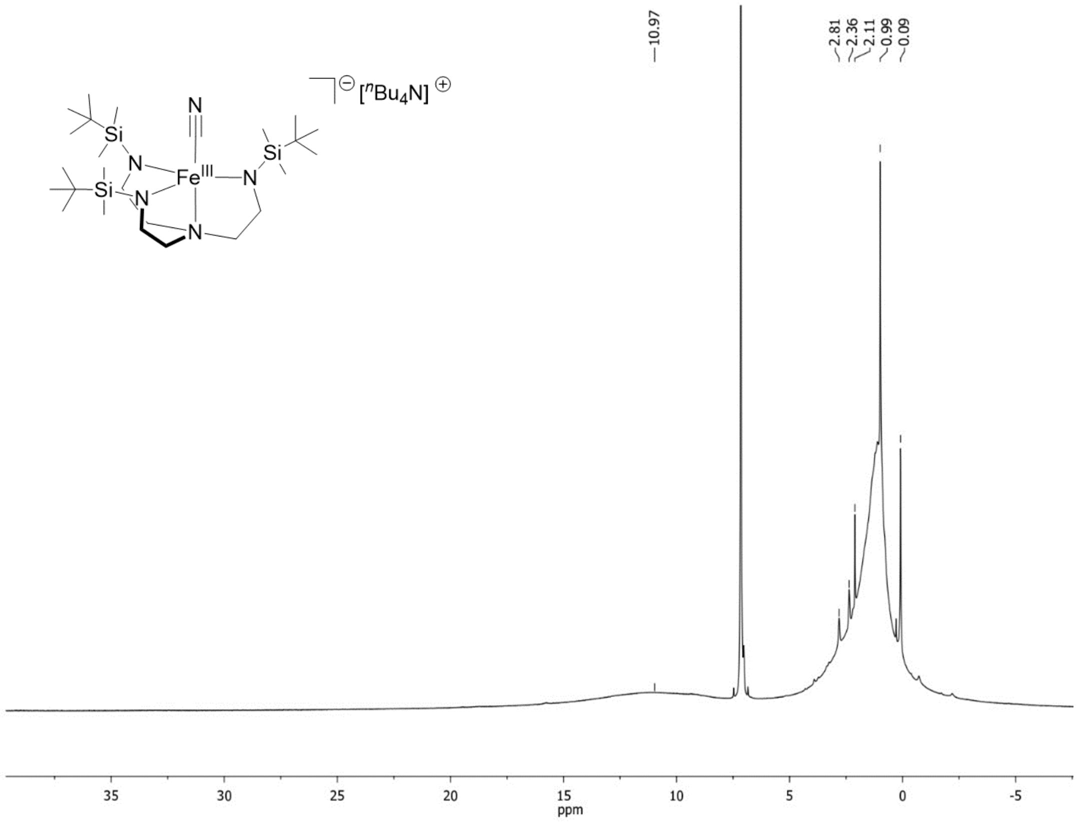

Figure SI-51. ^{1}H-NMR spectrum (250 MHz, C_6D_6, 298K) of [(N_3N')Fe^{III}(CN)]($^{n}Bu_4N$) (**2-Bu$_4$N**).

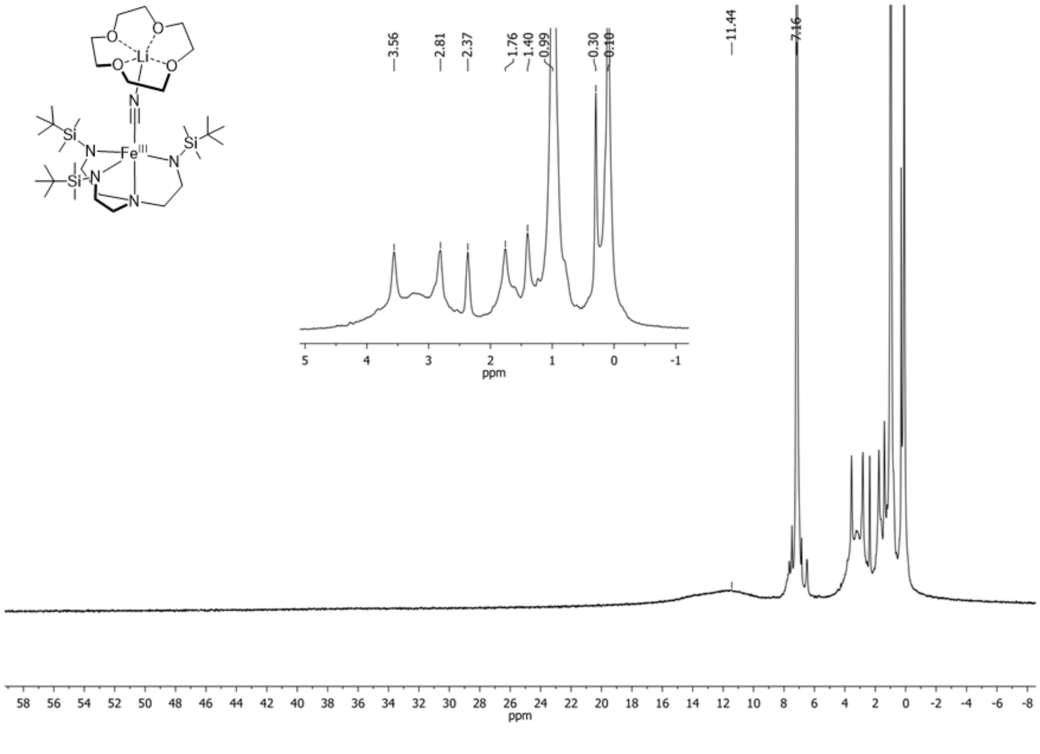

Figure SI-52. ^{1}H-NMR spectrum (250 MHz, C_6D_6, 298K) of [(N_3N')Fe^{III}(CN)Li(12-c-4)] (**2-Li^{crown}**).

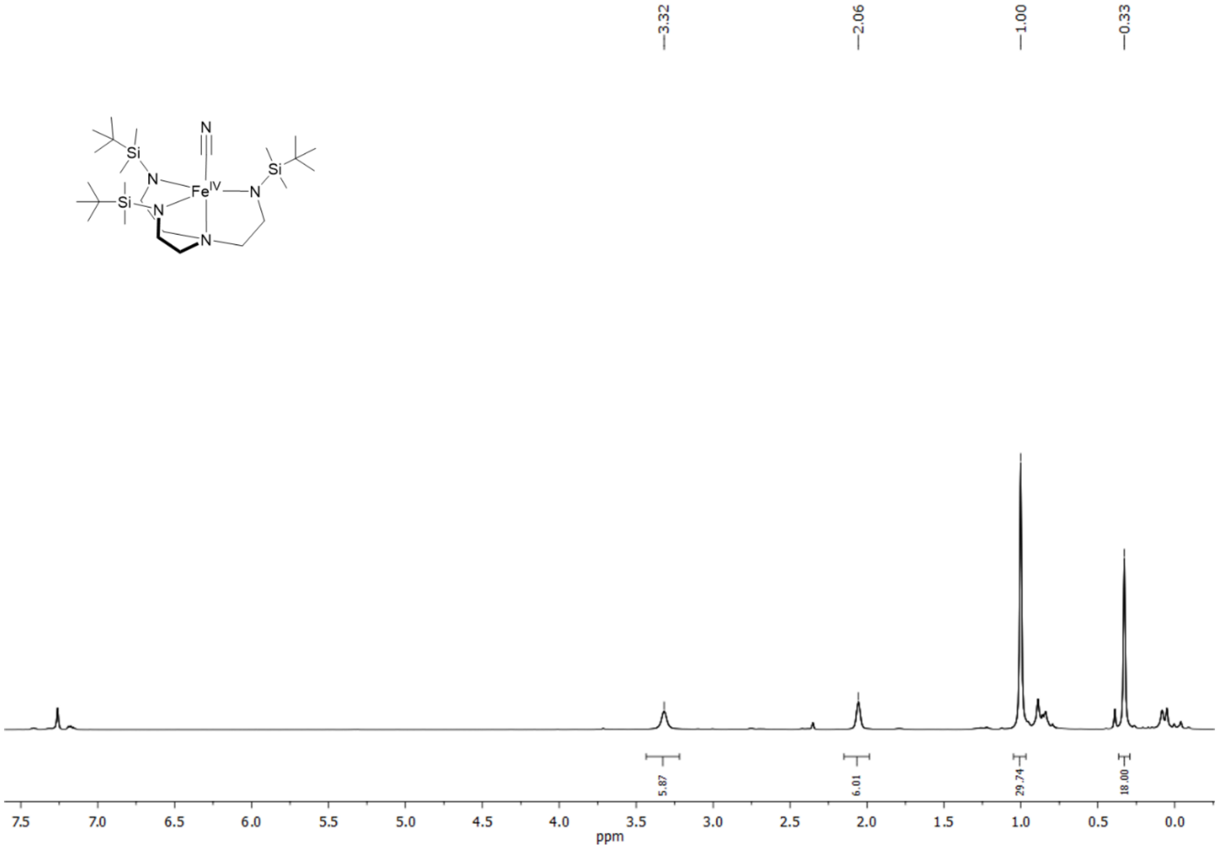

Figure SI-53. ^{1}H-NMR spectrum (500 MHz, $CDCl_3$, 233K) of [(N_3N')Fe^{IV}(CN)] (**4**).

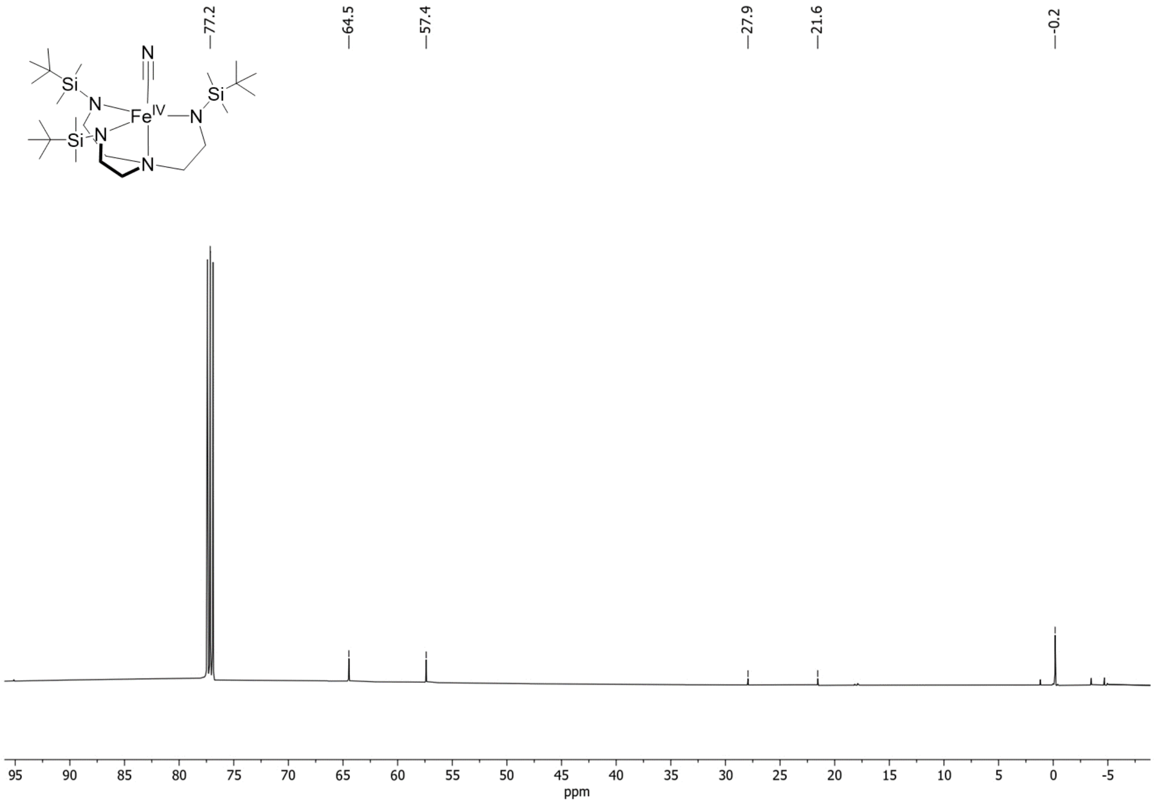

Figure SI-54. ^{13}C-NMR spectrum (126 MHz, $CDCl_3$, 233K) of [(N_3N')Fe^{IV}(CN)] (**4**).

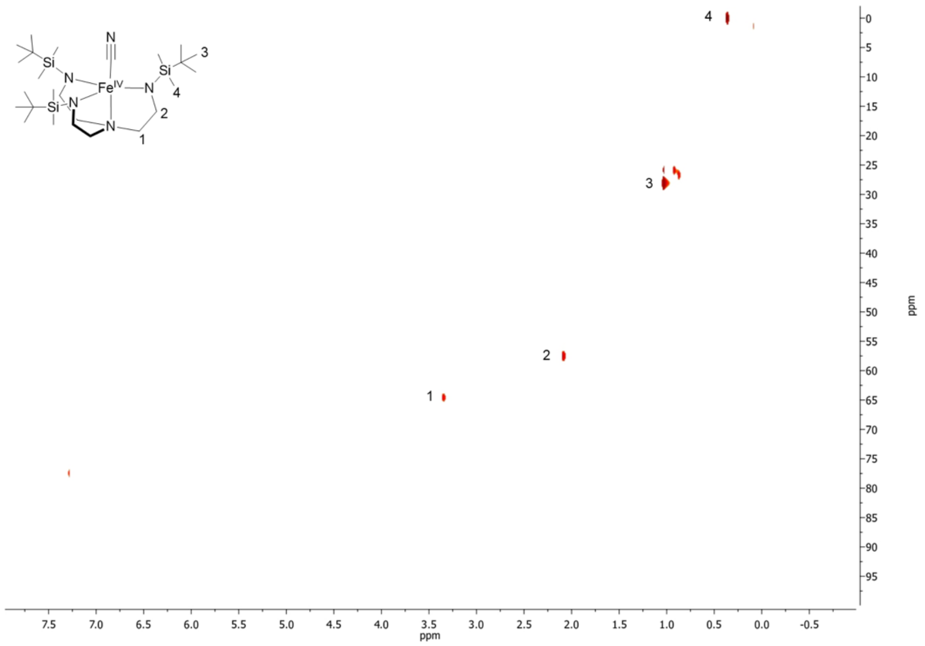

Figure SI-55. HMBC spectrum (126 MHz, $CDCl_3$, 233K) of [(N_3N')Fe^{IV}(CN)] (**4**).

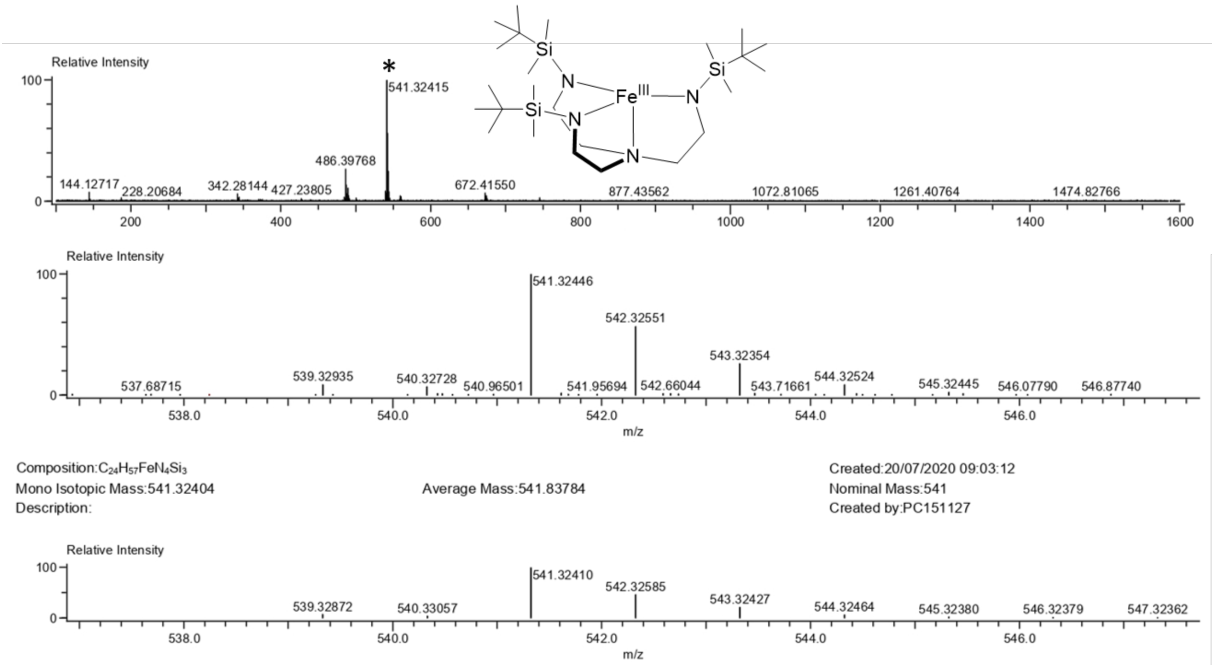

Figure SI-56. HRMS-FD of the solution of [(N_3N')Fe^{III}] (**3**) in THF at rt.

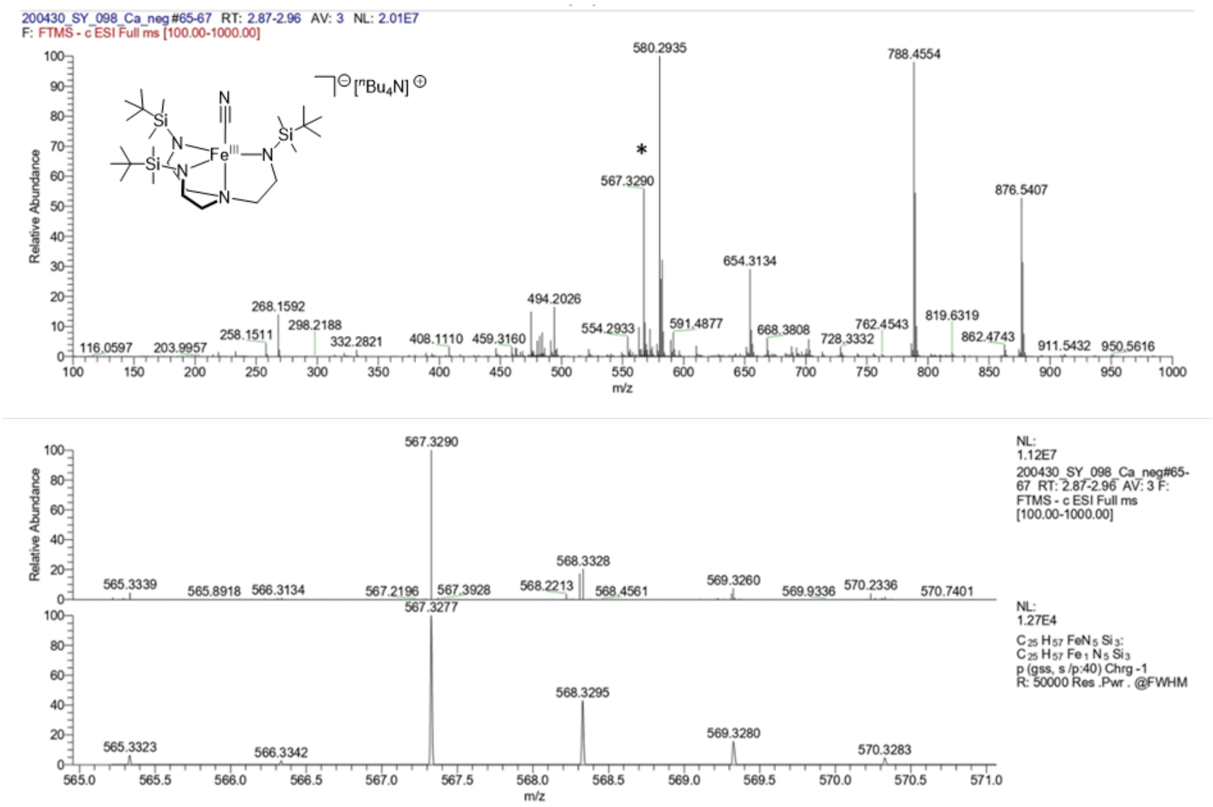

Figure SI-57. HRMS-ESI(-) of the solution of $[(N_3N')Fe^{III}(CN)](^nBu_4N)$ (**2-Bu₄N**) in THF at rt.

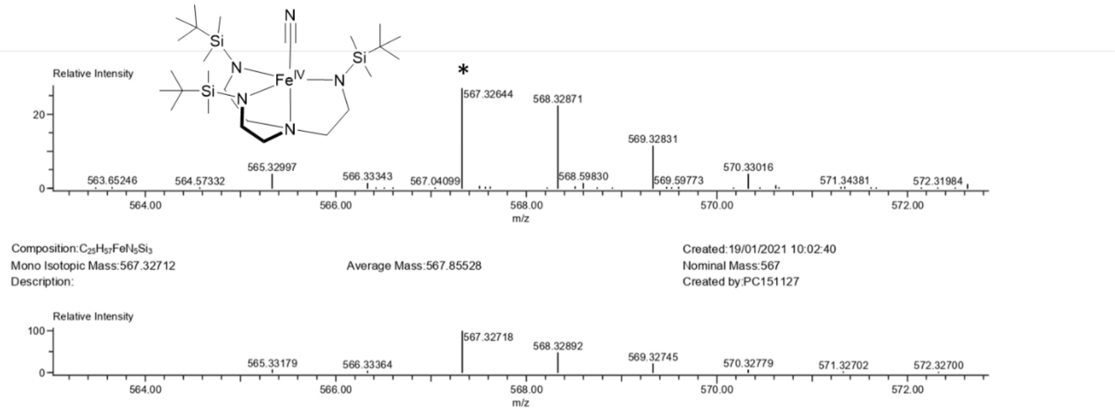

Mass	Intensity	Relative Intensity	Calc. Mass	Mass Difference [mDa]	Mass Difference [ppm]	Possible Formula	Unsaturation Number
567.32644	17435.78	46.14	567.32712	-0.68	-1.19	$^{12}C_{25}{}^{1}H_{57}{}^{56}Fe_{1}{}^{14}N_{5}{}^{28}Si_{3}$	3.5

Figure SI-58. HRMS-FD of the solution of $[(N_3N')Fe^{IV}(CN)]$ (**4**) in THF at rt.

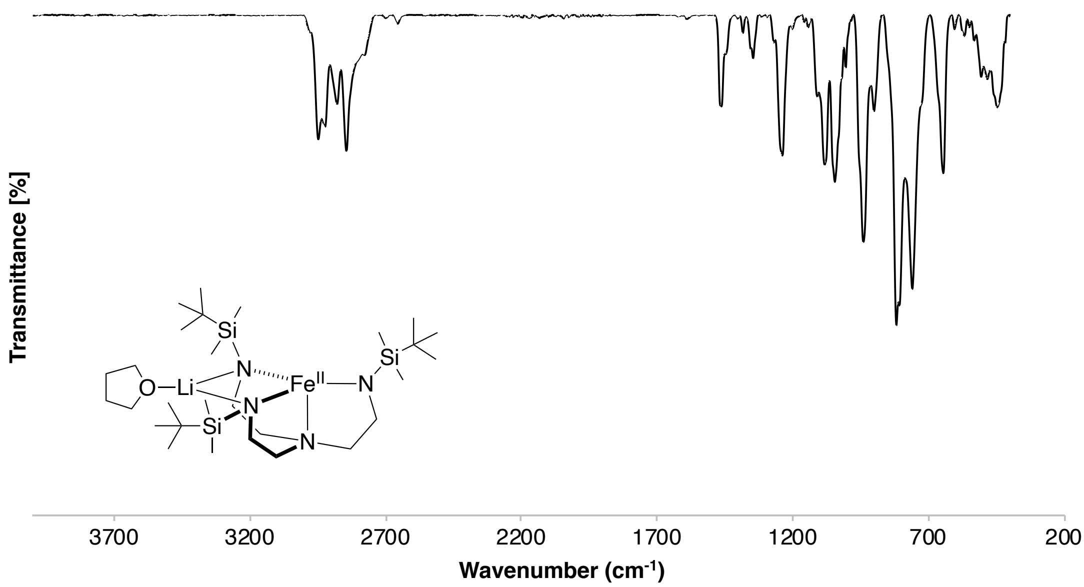

Figure SI-59. IR spectrum of [(N_3N')Fe^{II}Li(THF)] (**1**) complex deposited as a solid.

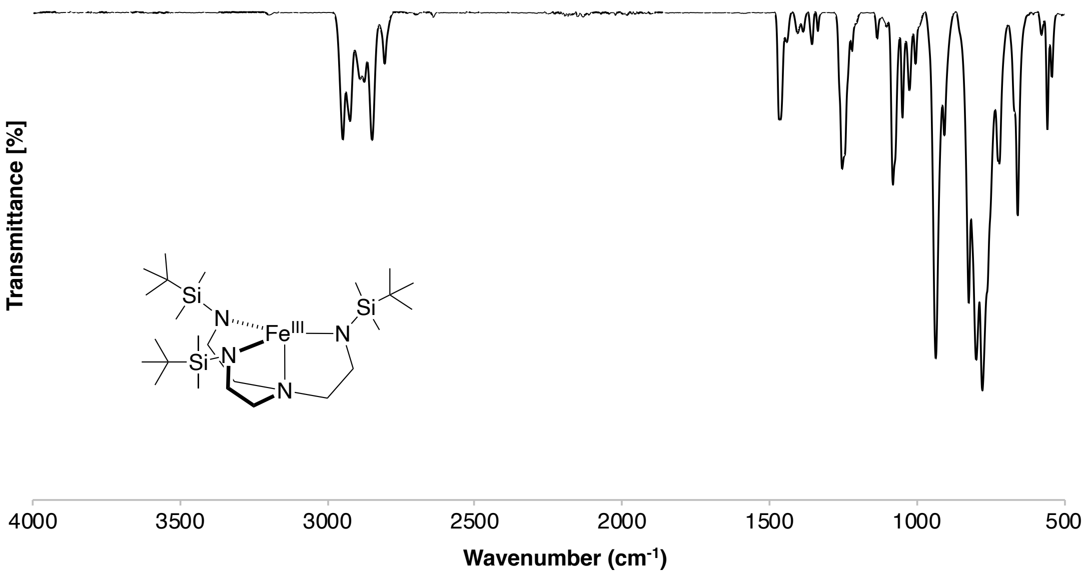

Figure SI-60. IR spectrum of [(N_3N')Fe^{III}] (**3**) deposited as a solid.

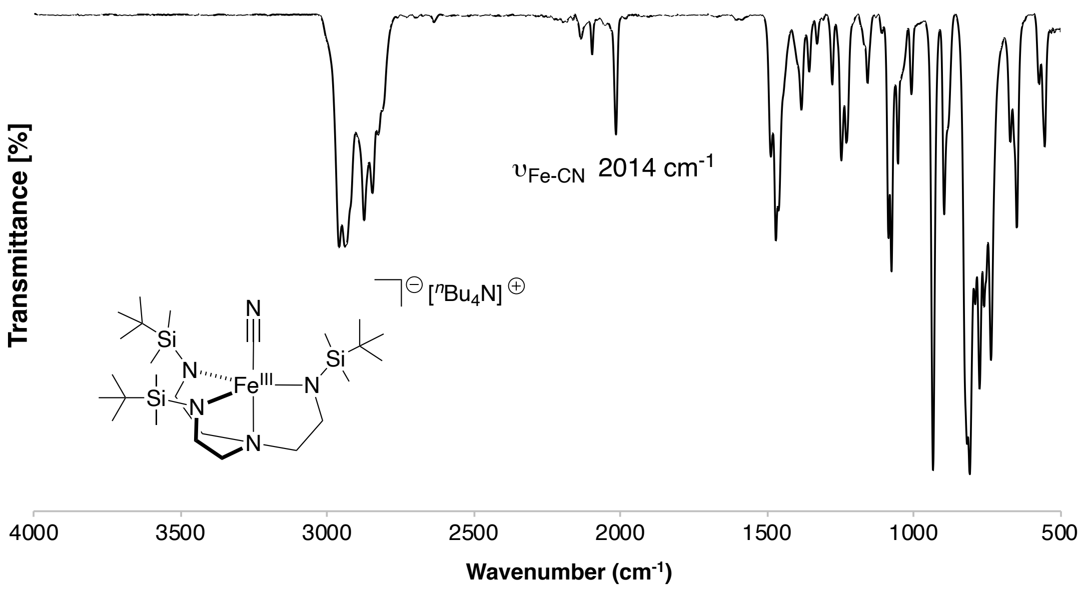

Figure SI-61. IR spectrum of [(N_3N')Fe^{III}(CN)(^{n}Bu_4N)] (**2-Bu$_4$N**) deposited as a solid.

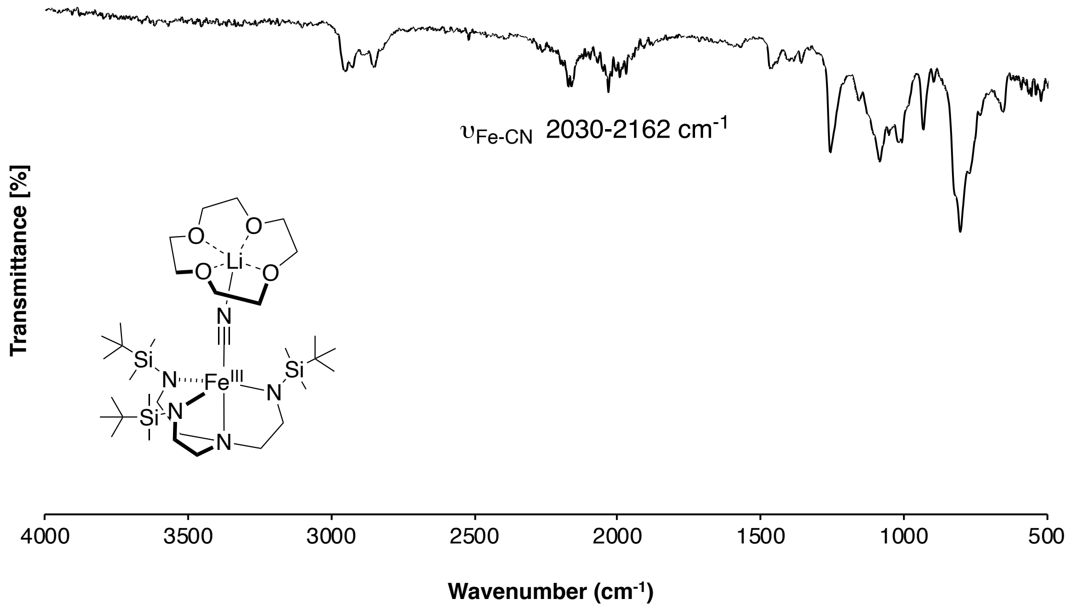

Figure SI-62. IR spectrum of [(N_3N')Fe^{III}Li(12-c-4)] (**2-Li^{crown}**) deposited as a solid.

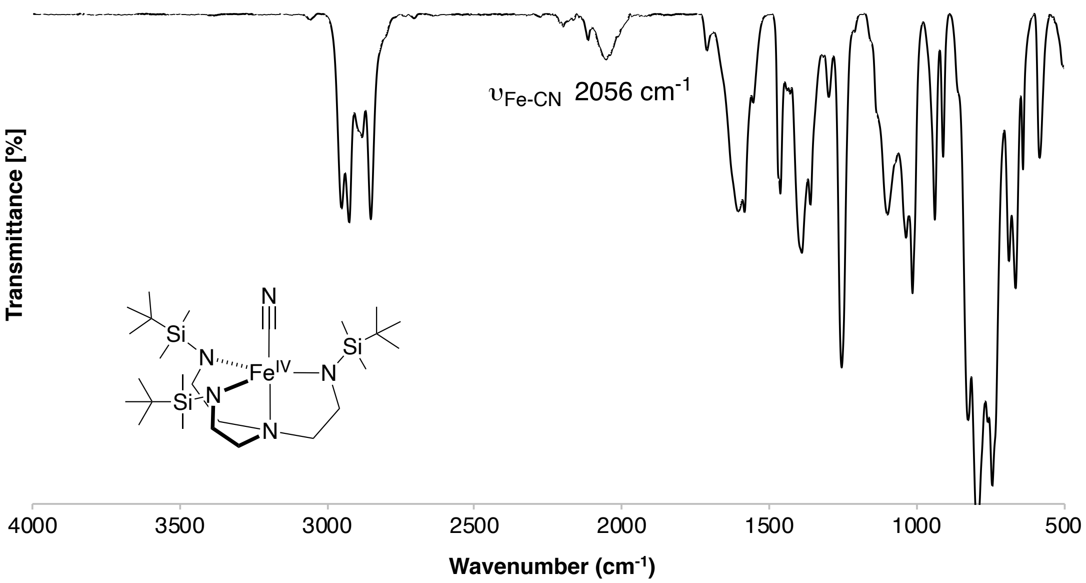

Figure SI-63. IR spectrum of [(N_3N')Fe^{IV}(CN)] (**4**) deposited as a solid.

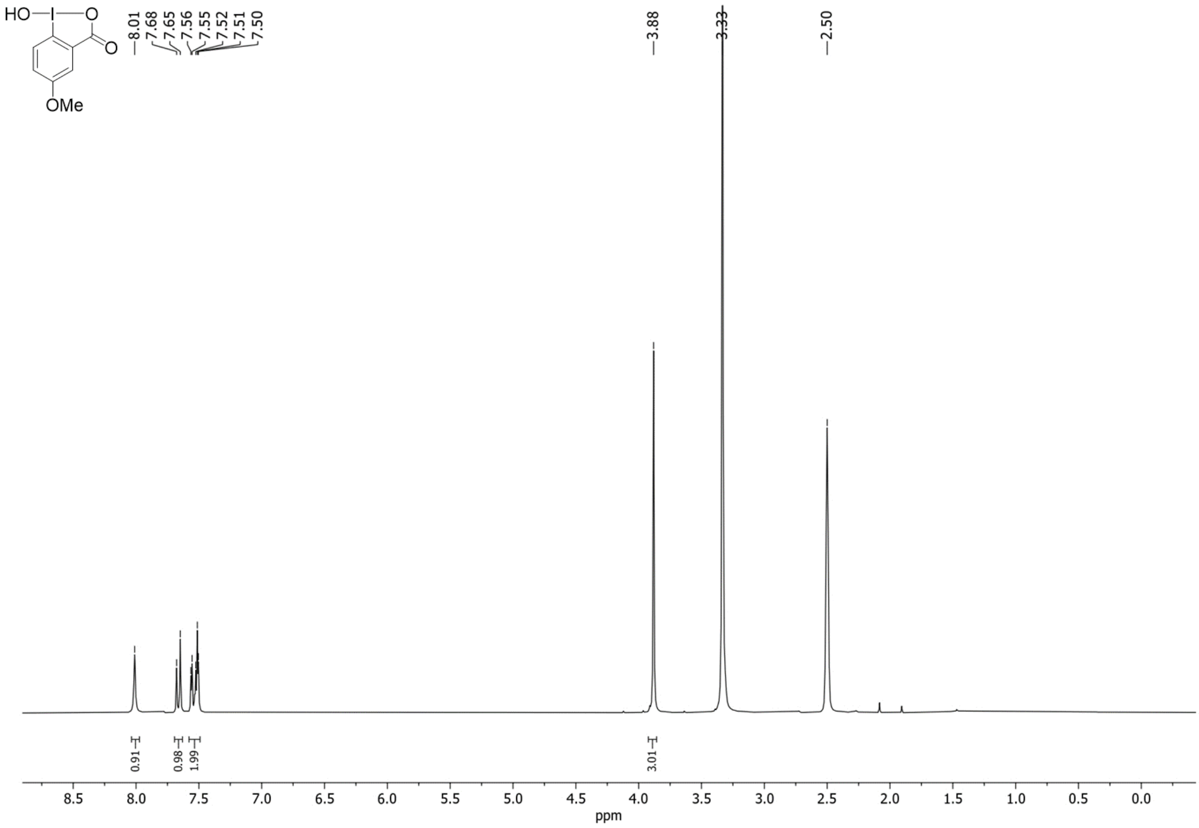

Figure SI-64. ^{1}H-NMR spectrum (300 MHz, DMSO-d$_6$, 298K) of **Int-1^{OMe}**.

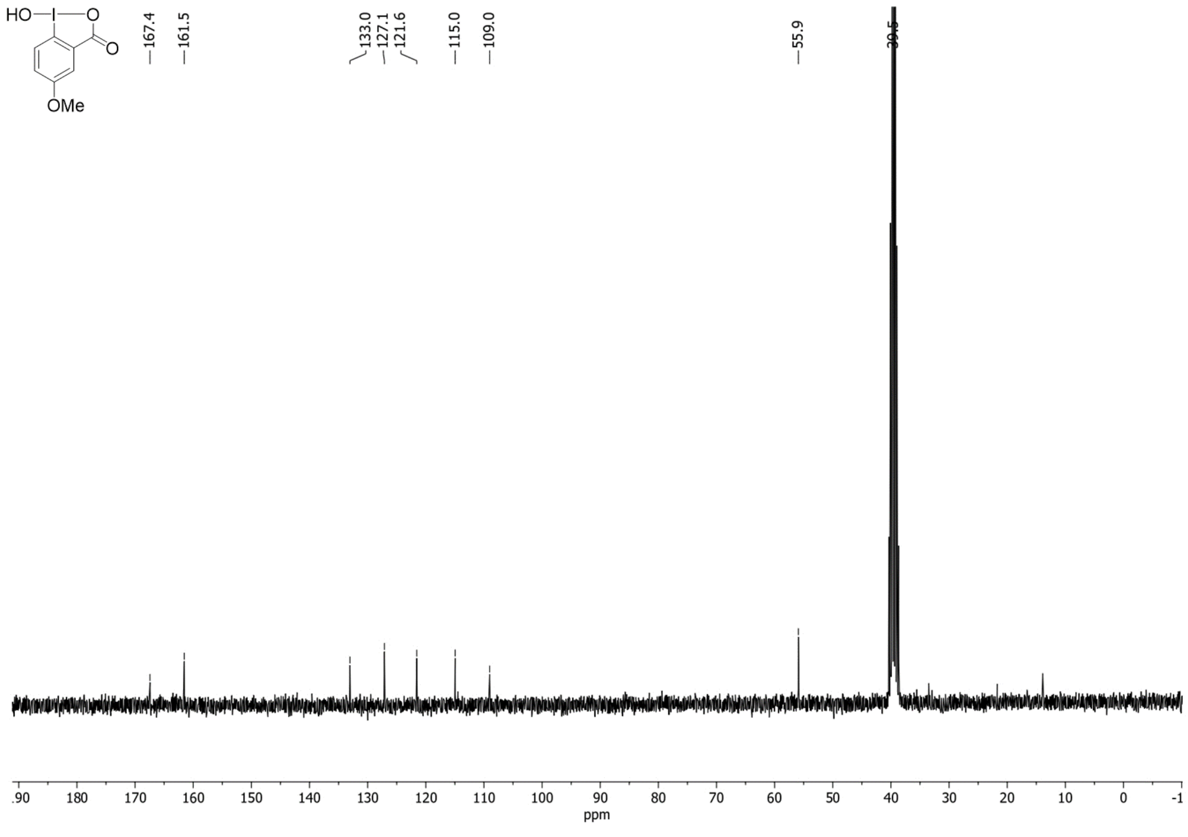

Figure SI-65. ^{13}C-NMR spectrum (75 MHz, DMSO-d$_6$, 298K) of **Int-1^{OMe}**.

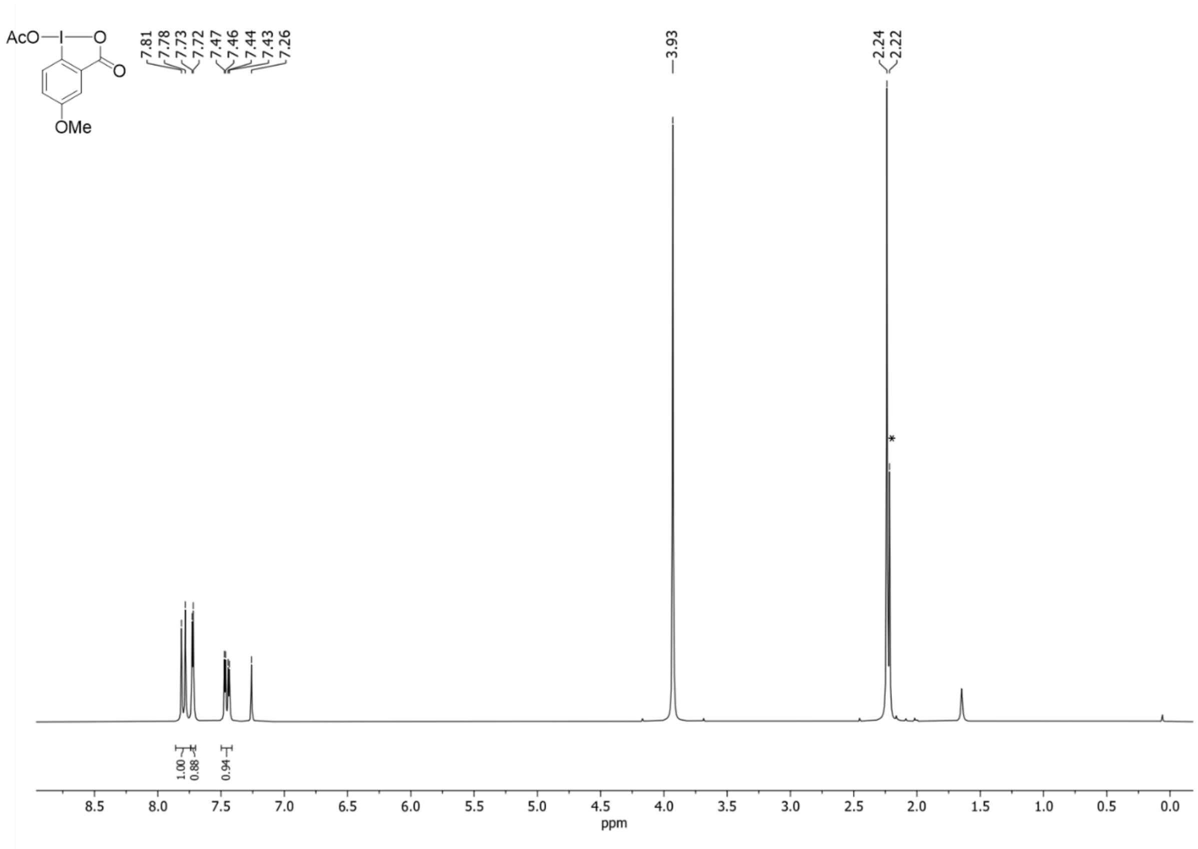

Figure SI-66. ^{1}H-NMR spectrum (300 MHz, $CDCl_3$, 298K) of **Int-2^{OMe}**.

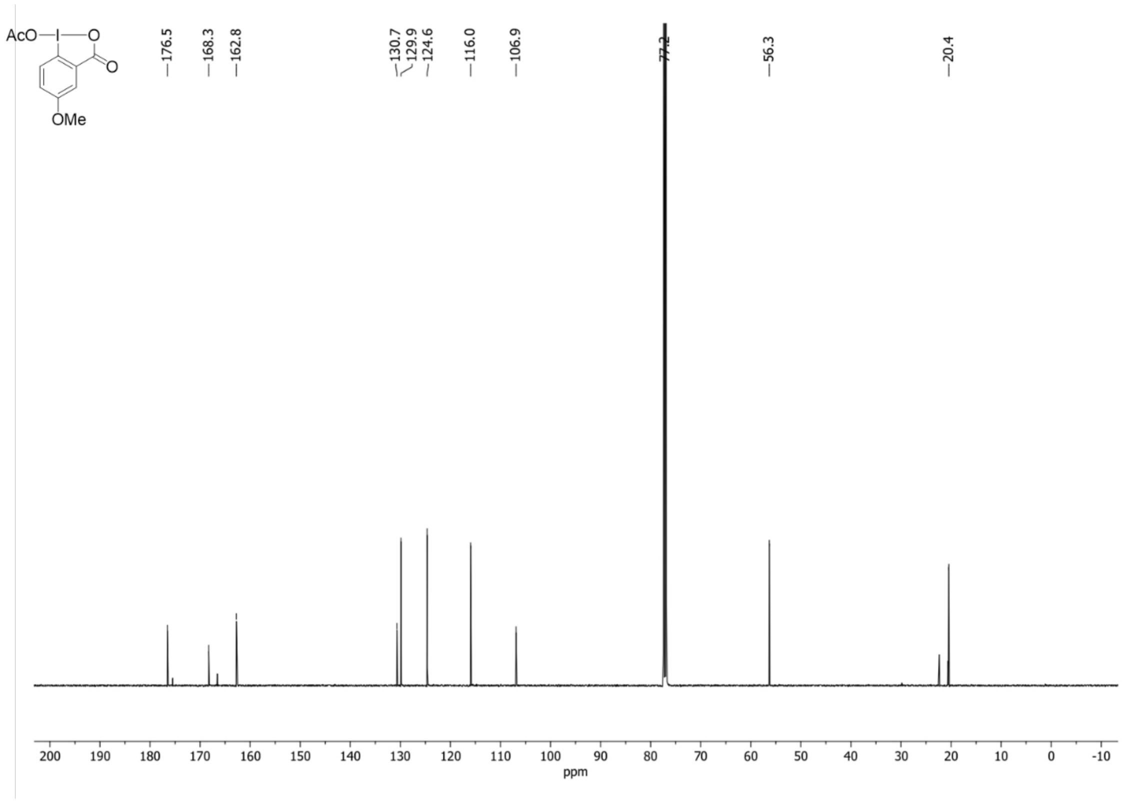

Figure SI-67. ^{13}C-NMR spectrum (126 MHz, $CDCl_3$, 298K) of **Int-2^{OMe}**.

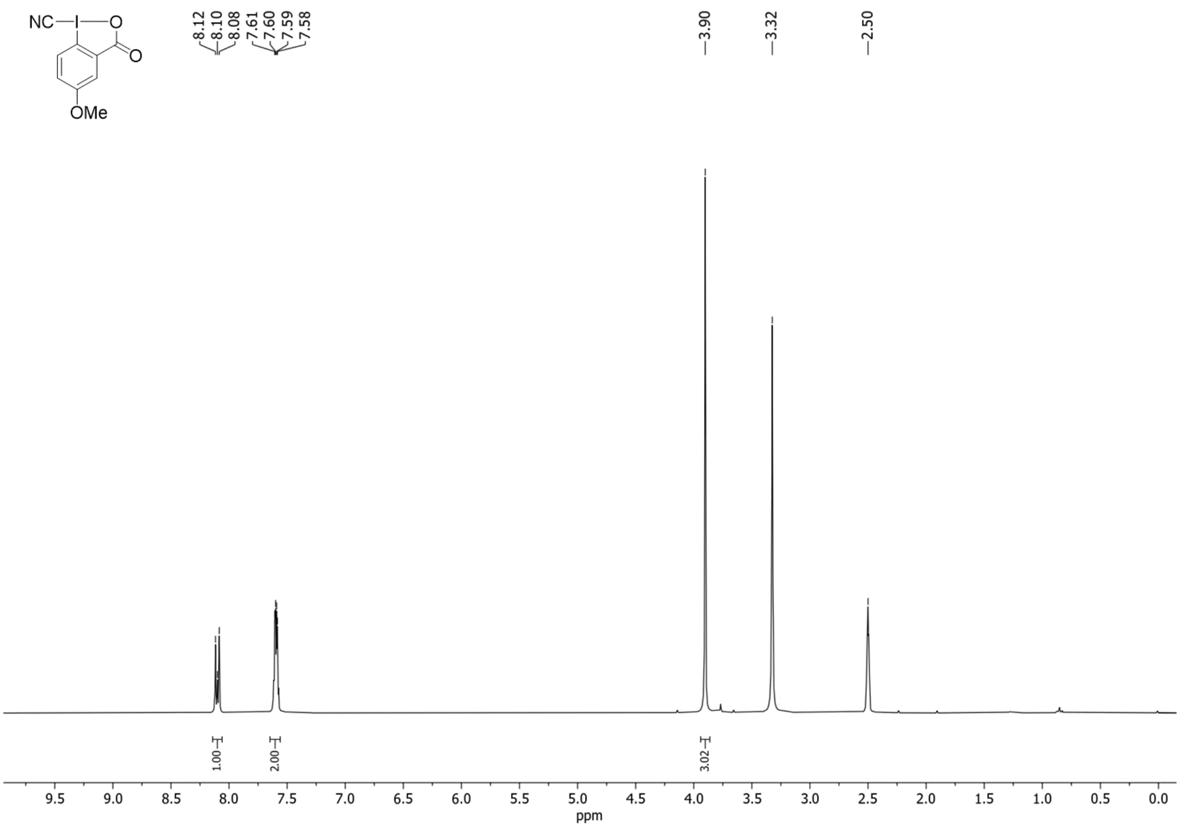

Figure SI-68. ^{1}H-NMR spectrum (300 MHz, DMSO-d_6, 298K) of **CBX^{OMe}**.

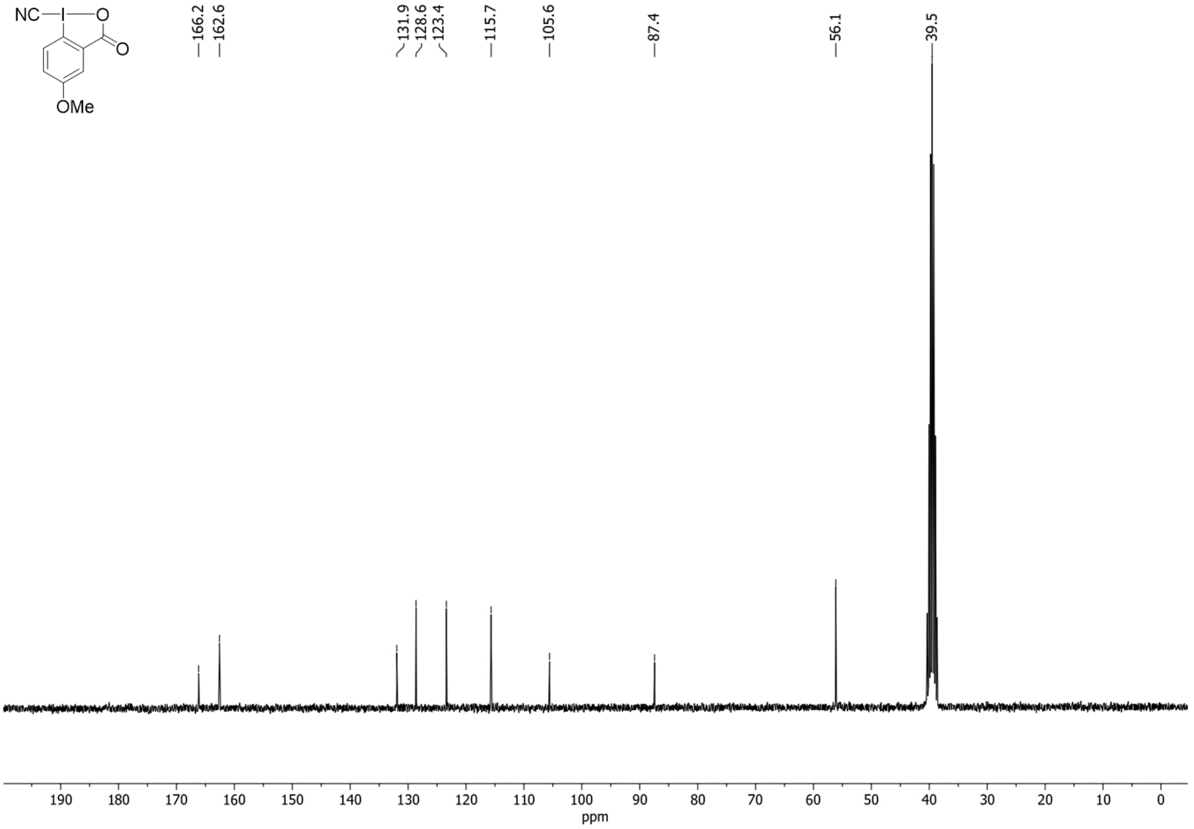

Figure SI-69. ^{13}C-NMR spectrum (75 MHz, DMSO-d_6, 298K) of **CBX^{OMe}**.

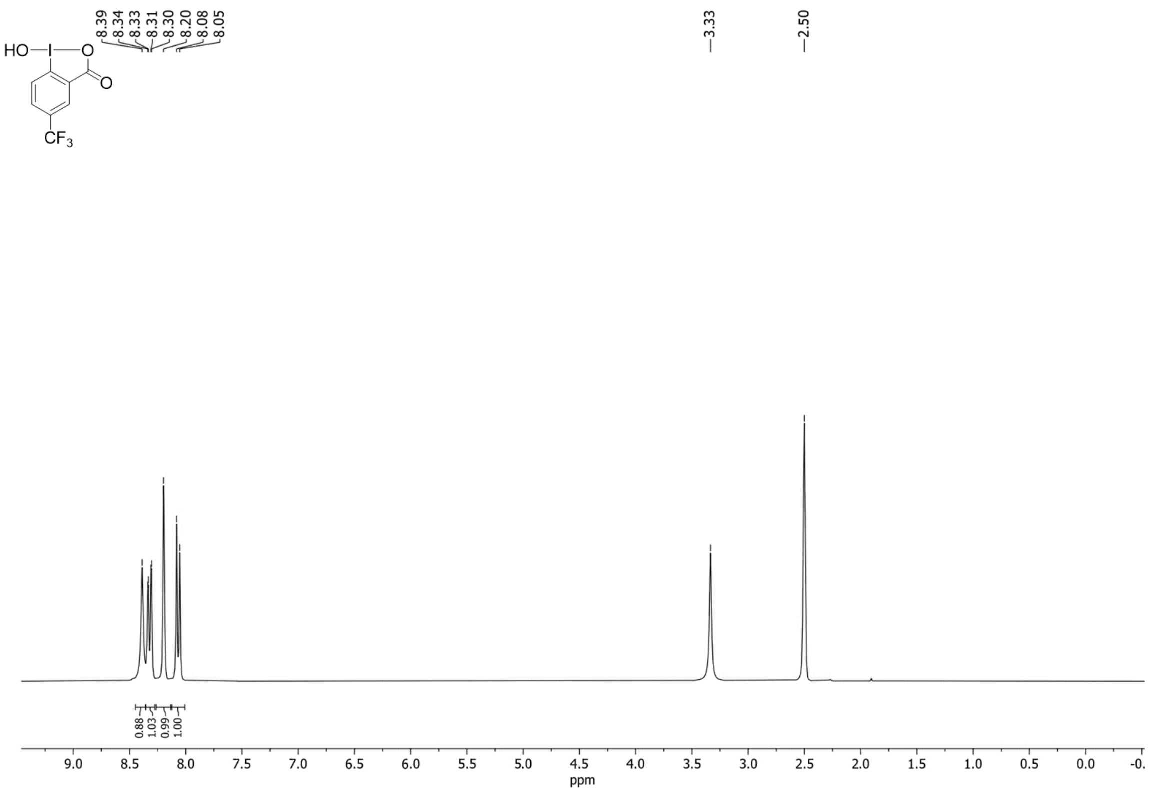

Figure SI-70. ^{1}H-NMR spectrum (300 MHz, DMSO-d$_6$, 298K) of **Int-1^{CF3}**.

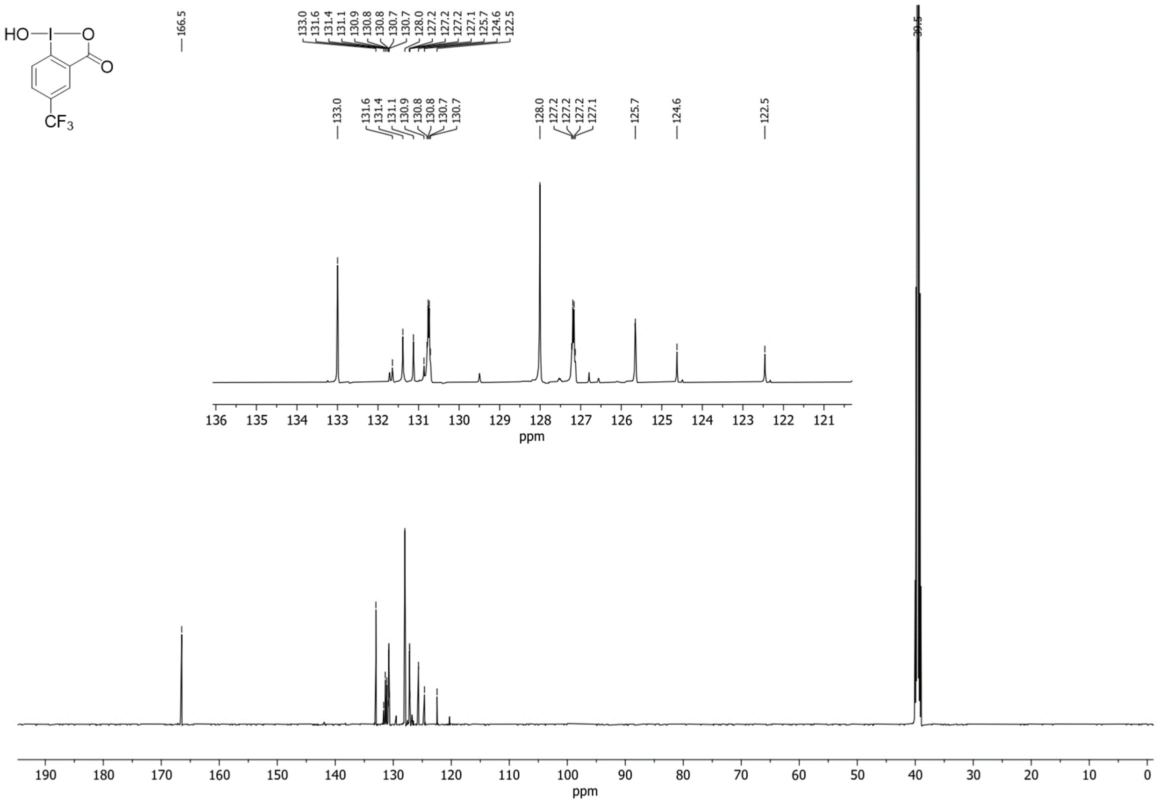

Figure SI-71. ^{13}C-NMR spectrum (126 MHz, DMSO-d$_6$, 298K) of **Int-1^{CF3}**.

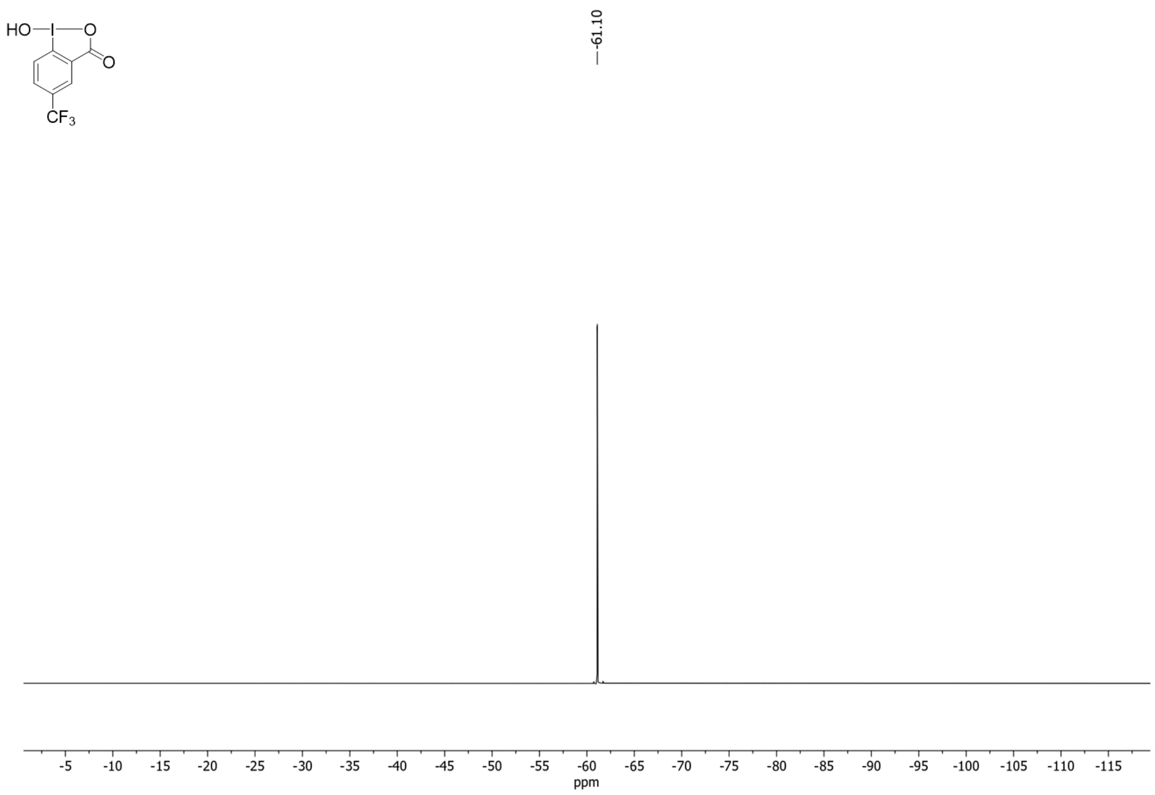

Figure SI-72. ^{19}F-NMR spectrum (282 MHz, DMSO-d_6, 298K) of **Int-1^{CF3}**.

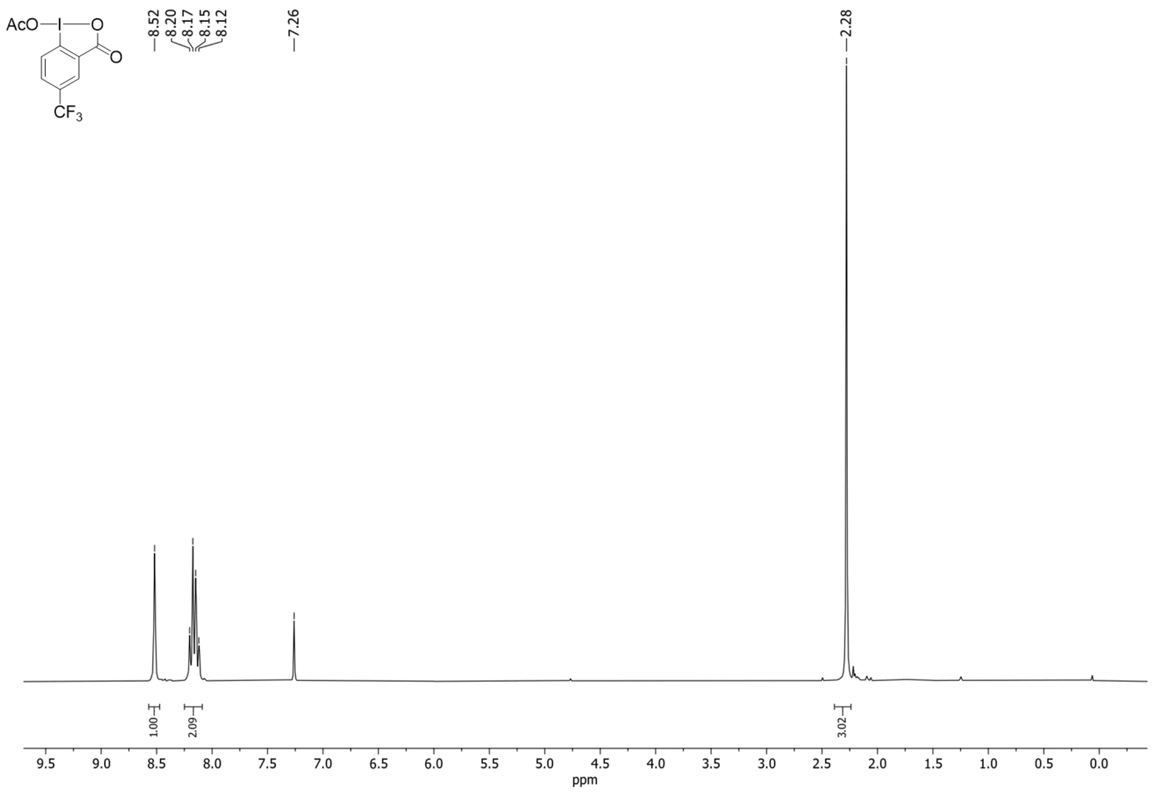

Figure SI-73. ^{1}H-NMR spectrum (300 MHz, $CDCl_3$, 298K) of **Int-2^{CF3}**.

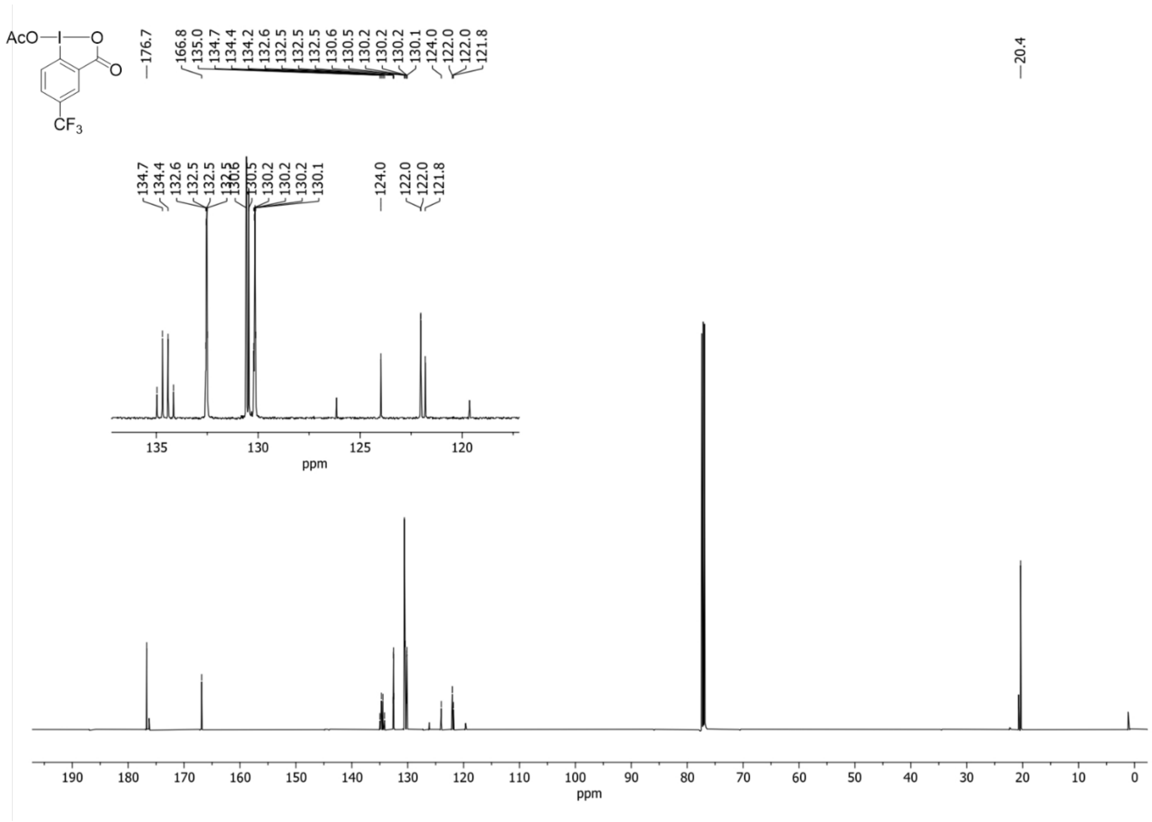

Figure SI-74. ^{13}C-NMR spectrum (75 MHz, $CDCl_3$, 298K) of **Int-2^{CF3}**.

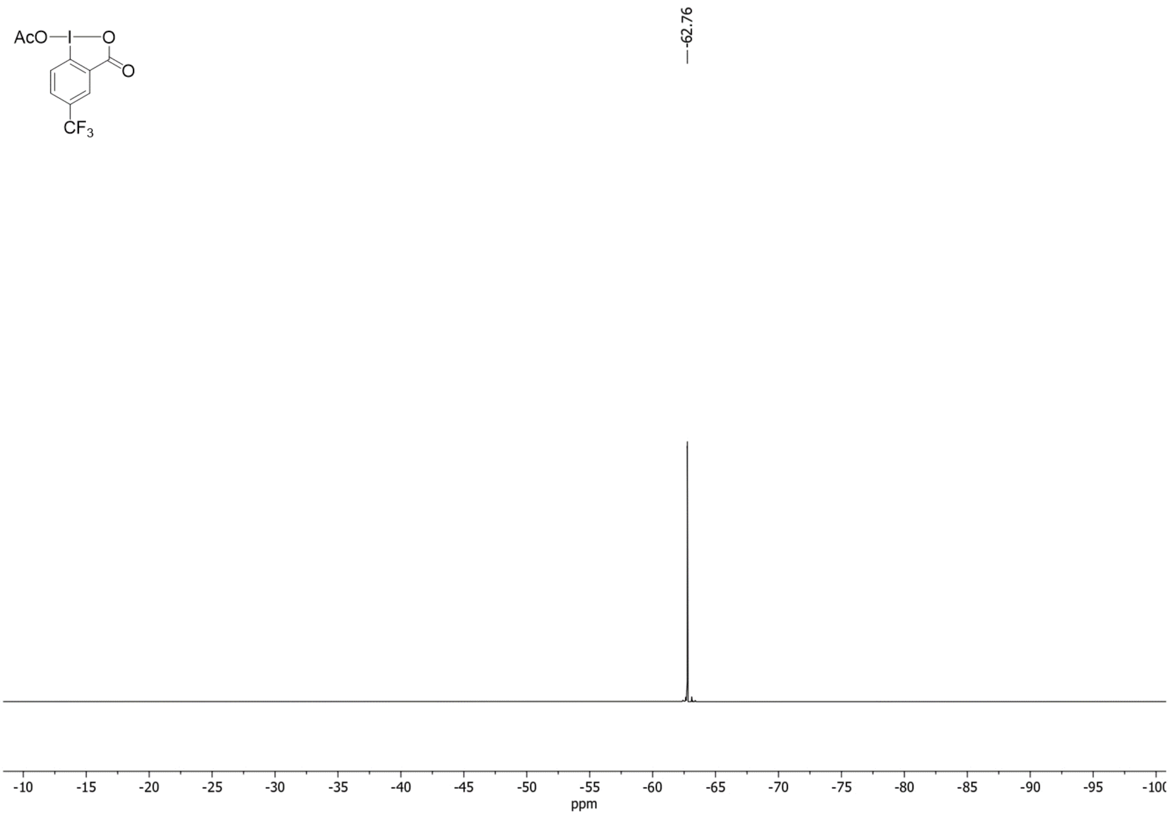

Figure SI-75. ^{19}F-NMR spectrum (282 MHz, $CDCl_3$, 298K) of **Int-2^{CF3}**.

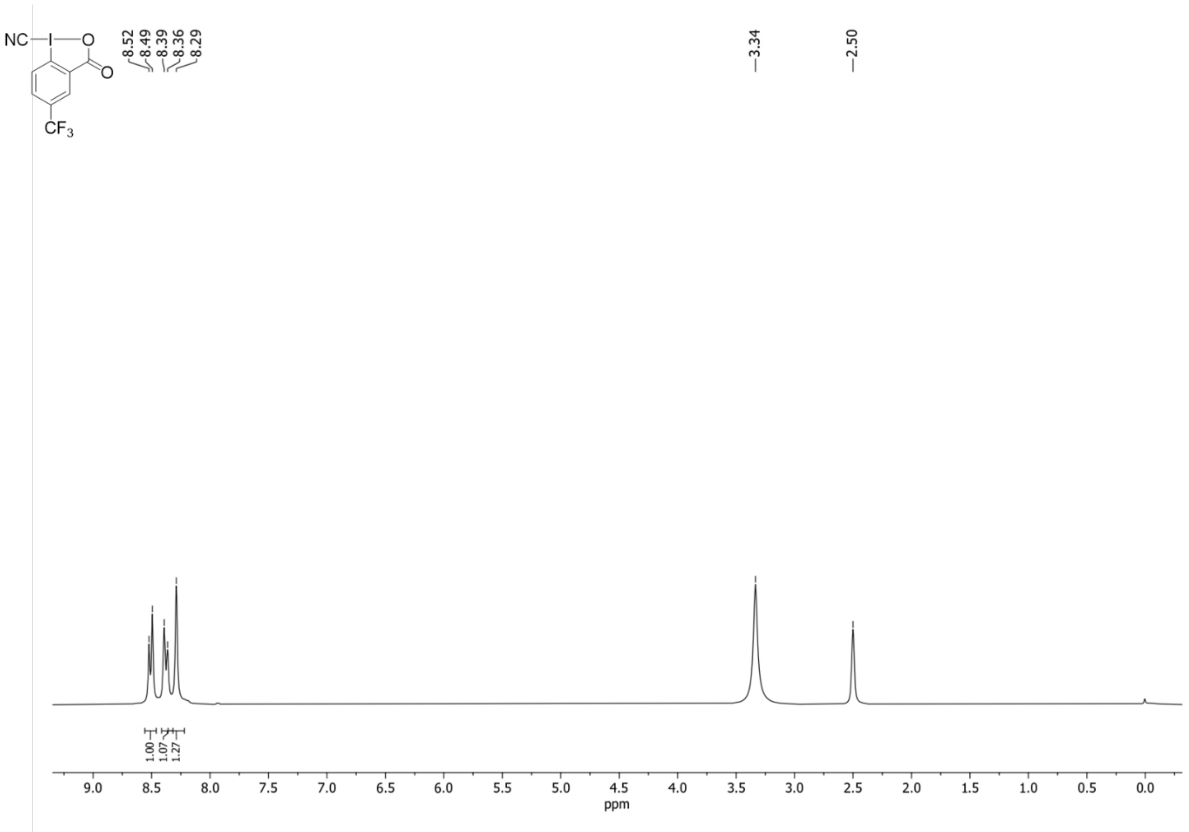

Figure SI-76. ^{1}H-NMR spectrum (300 MHz, DMSO-d_6, 298K) of **CBX^{CF3}**.

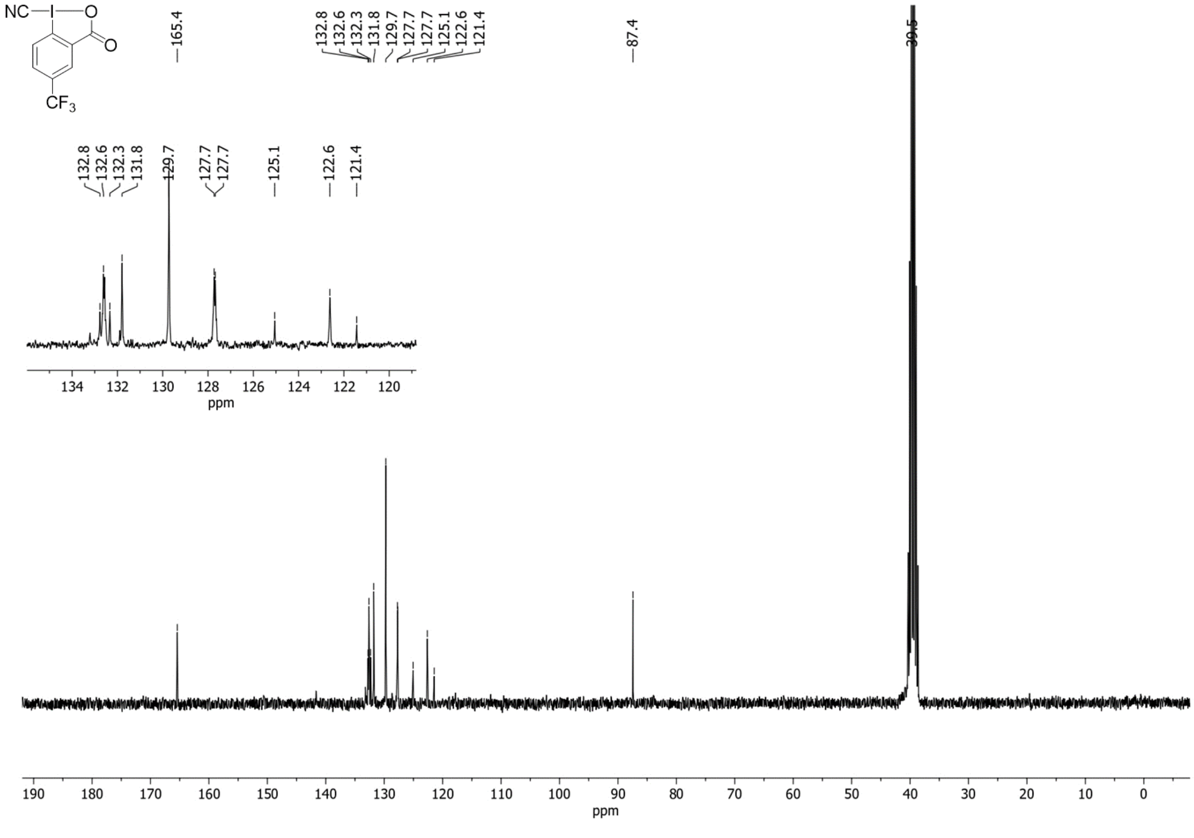

Figure SI-77. ^{13}C-NMR spectrum (75 MHz, DMSO-d_6, 298K) of **CBX^{CF3}**.

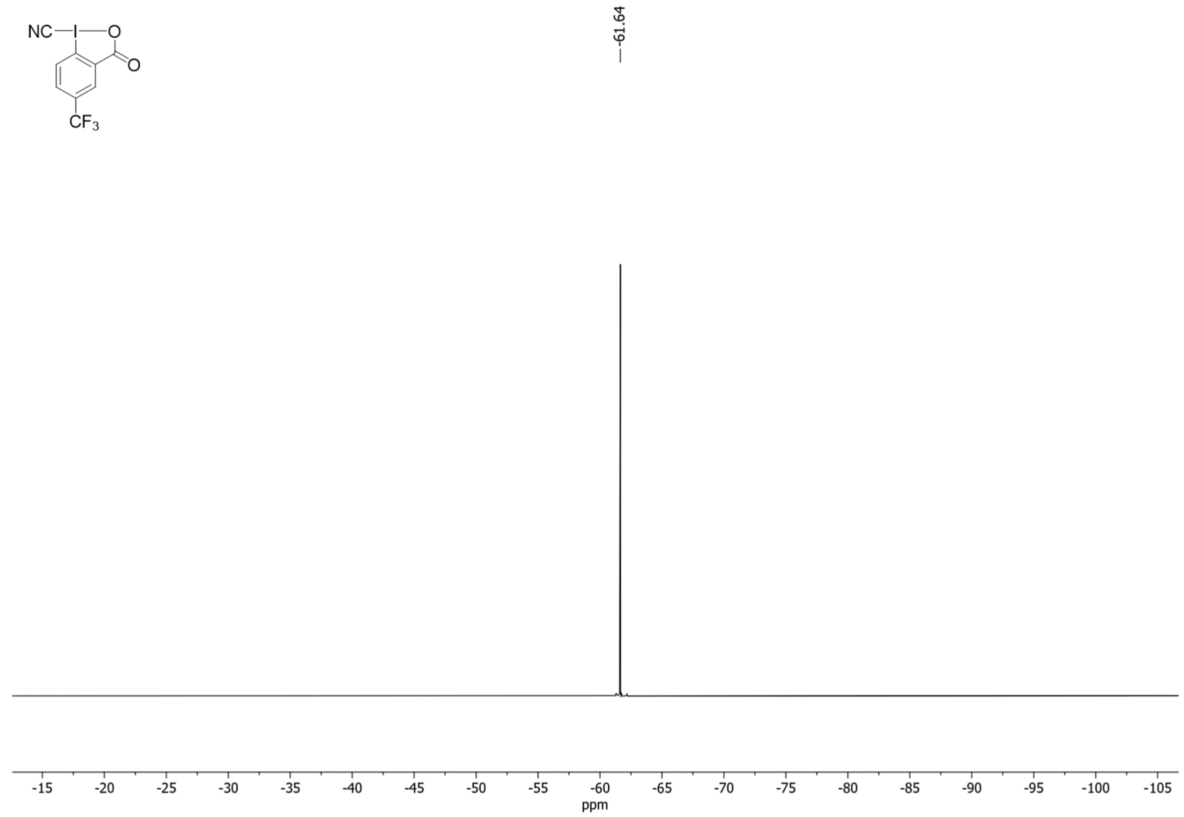

Figure SI-78. ^{19}F-NMR spectrum (282 MHz, DMSO-d$_6$, 298K) of **CBX^{CF3}**.

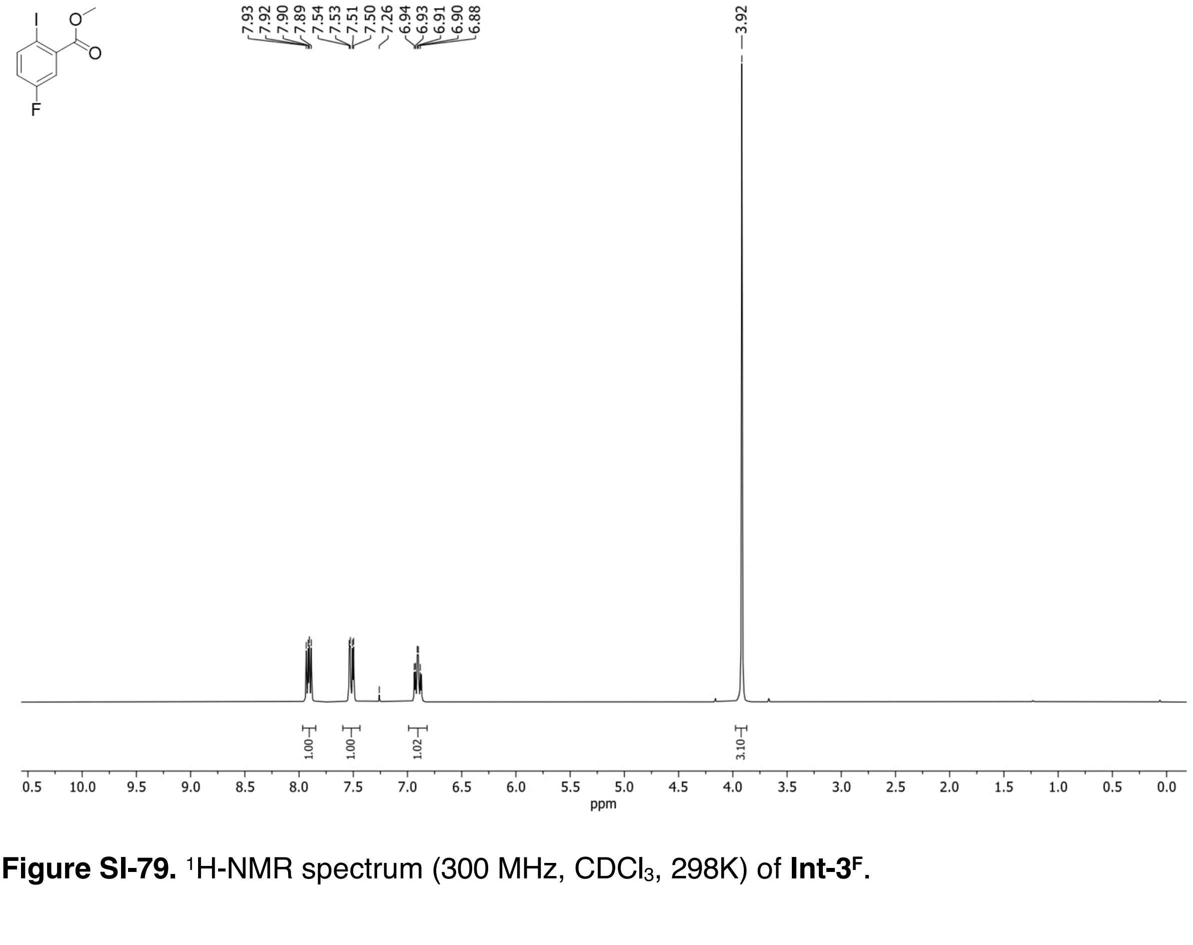

Figure SI-79. ^{1}H-NMR spectrum (300 MHz, CDCl$_3$, 298K) of **Int-3^{F}**.

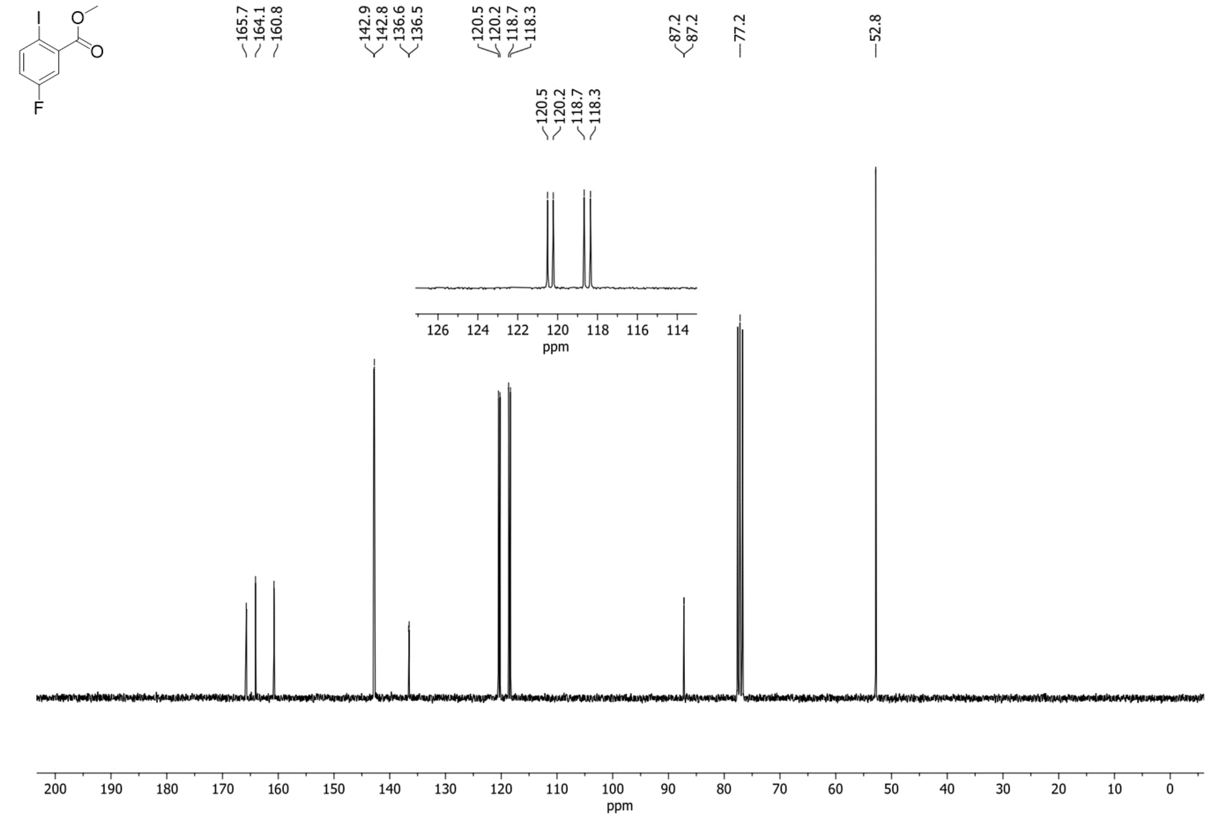

Figure SI-80. ^{13}C-NMR spectrum (75 MHz, $CDCl_3$, 298K) of **Int-3^{F}**.

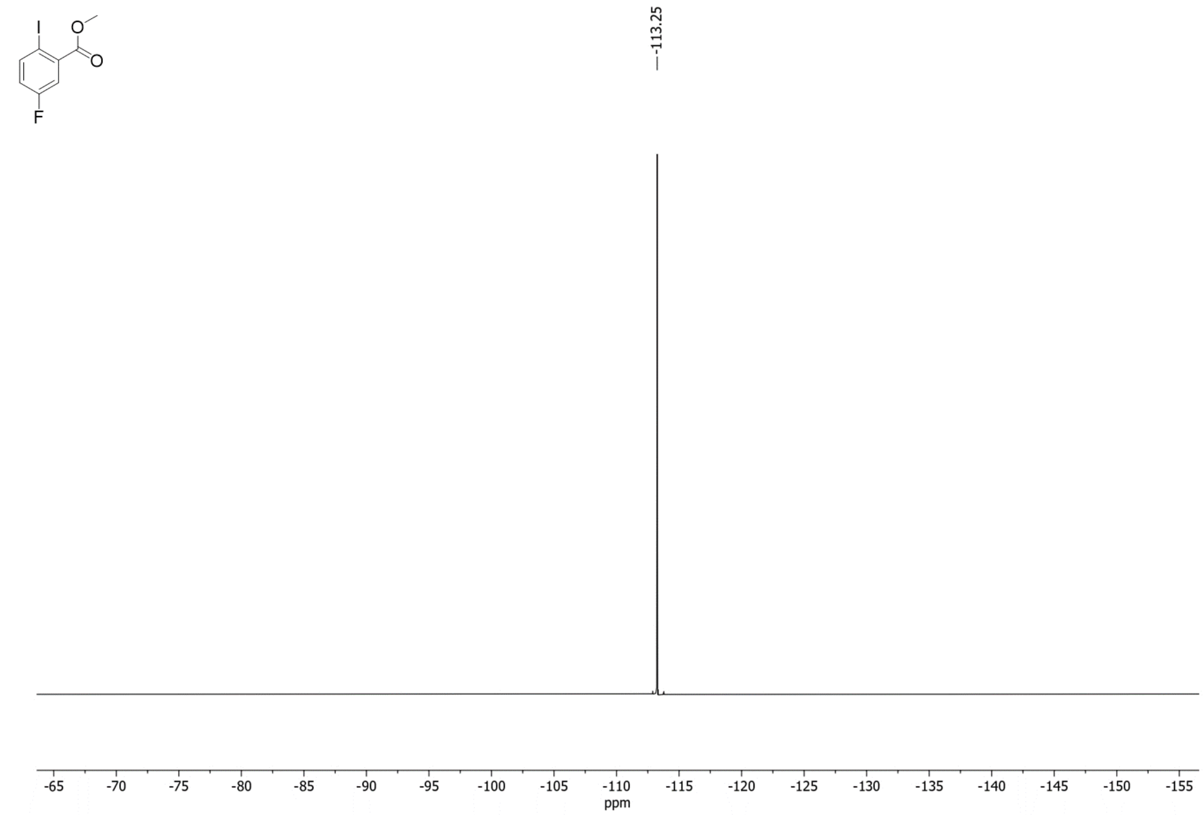

Figure SI-81. ^{19}F-NMR spectrum (282 MHz, $CDCl_3$, 298K) of **Int-3^{F}**.

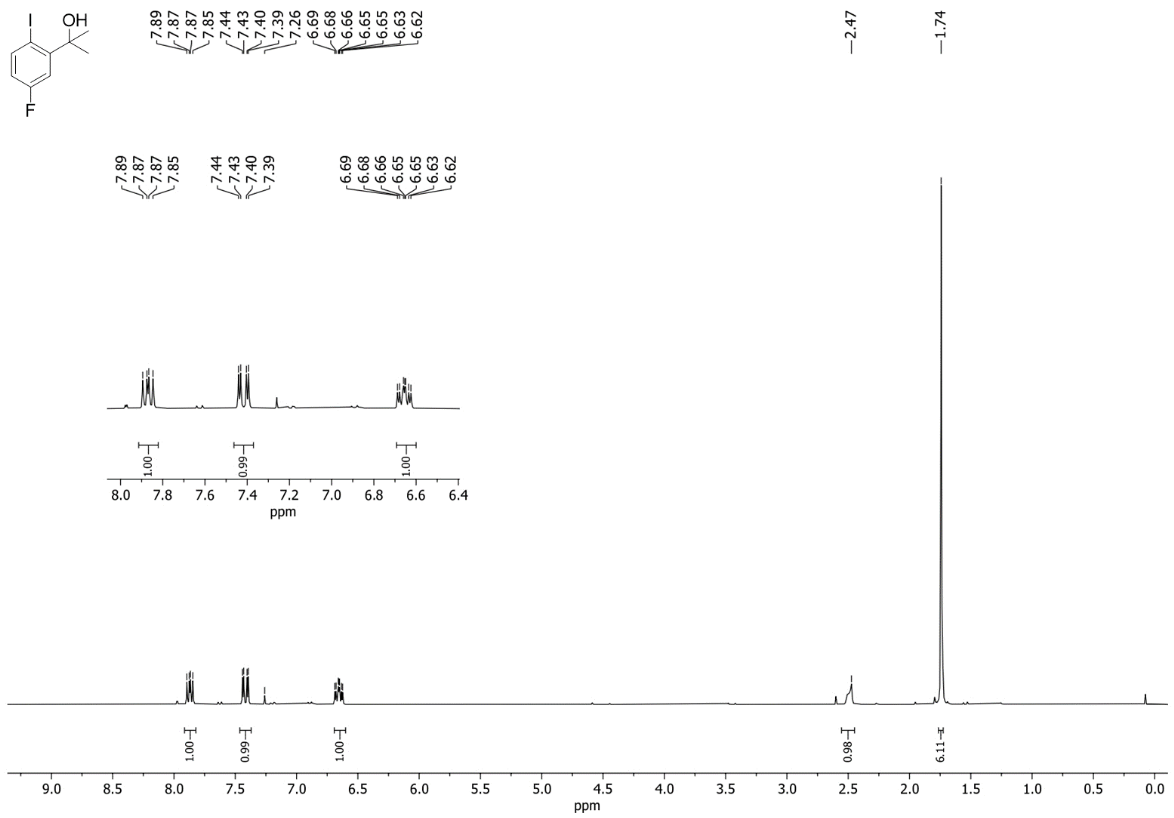

Figure SI-82. ^{1}H-NMR spectrum (300 MHz, $CDCl_3$, 298K) of **Int-4^{F}**.

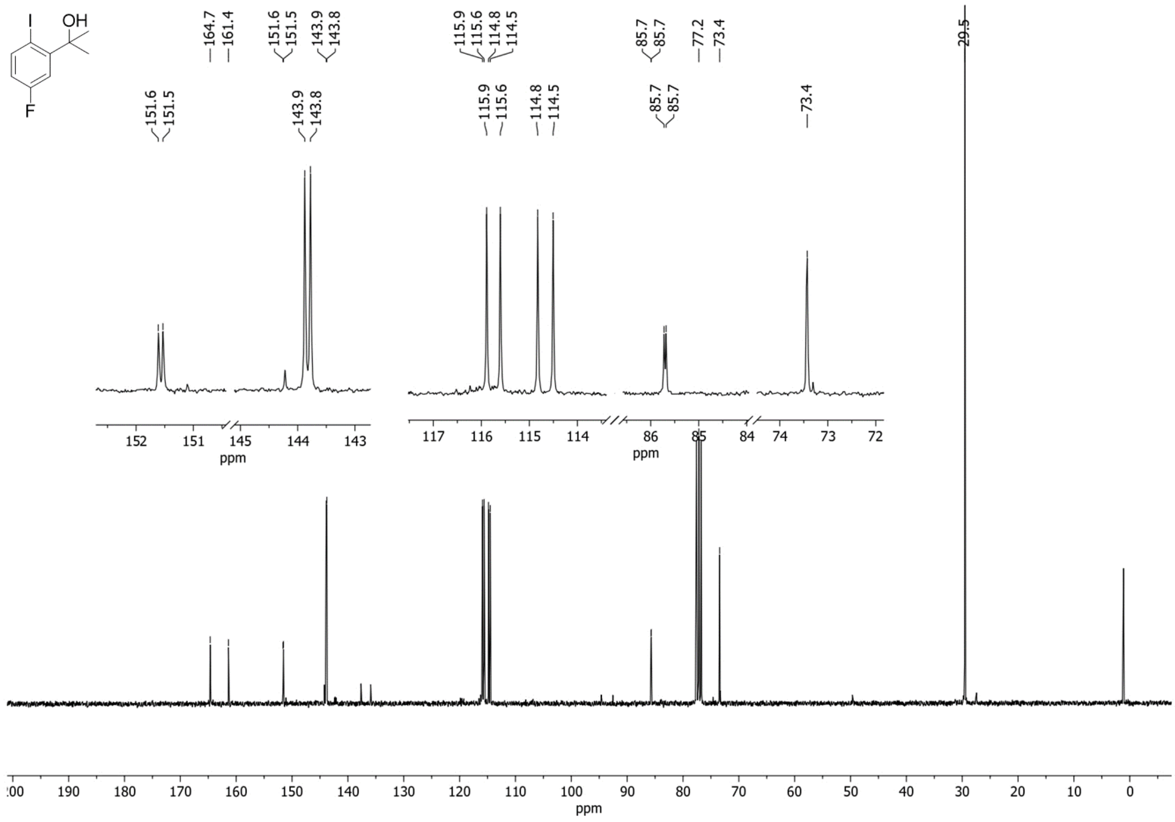

Figure SI-83. ^{13}C-NMR spectrum (75 MHz, $CDCl_3$, 298K) of **Int-4^{F}**.

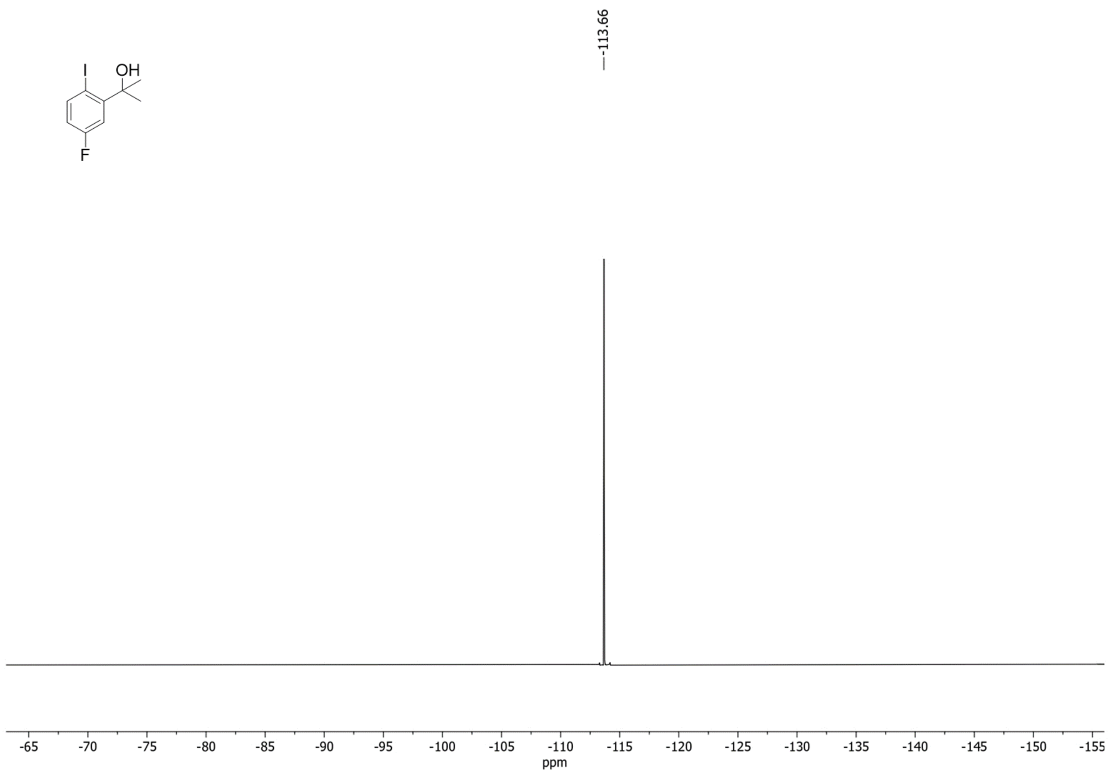

Figure SI-84. ^{19}F-NMR spectrum (282 MHz, $CDCl_3$, 298K) of **Int-4^F**.

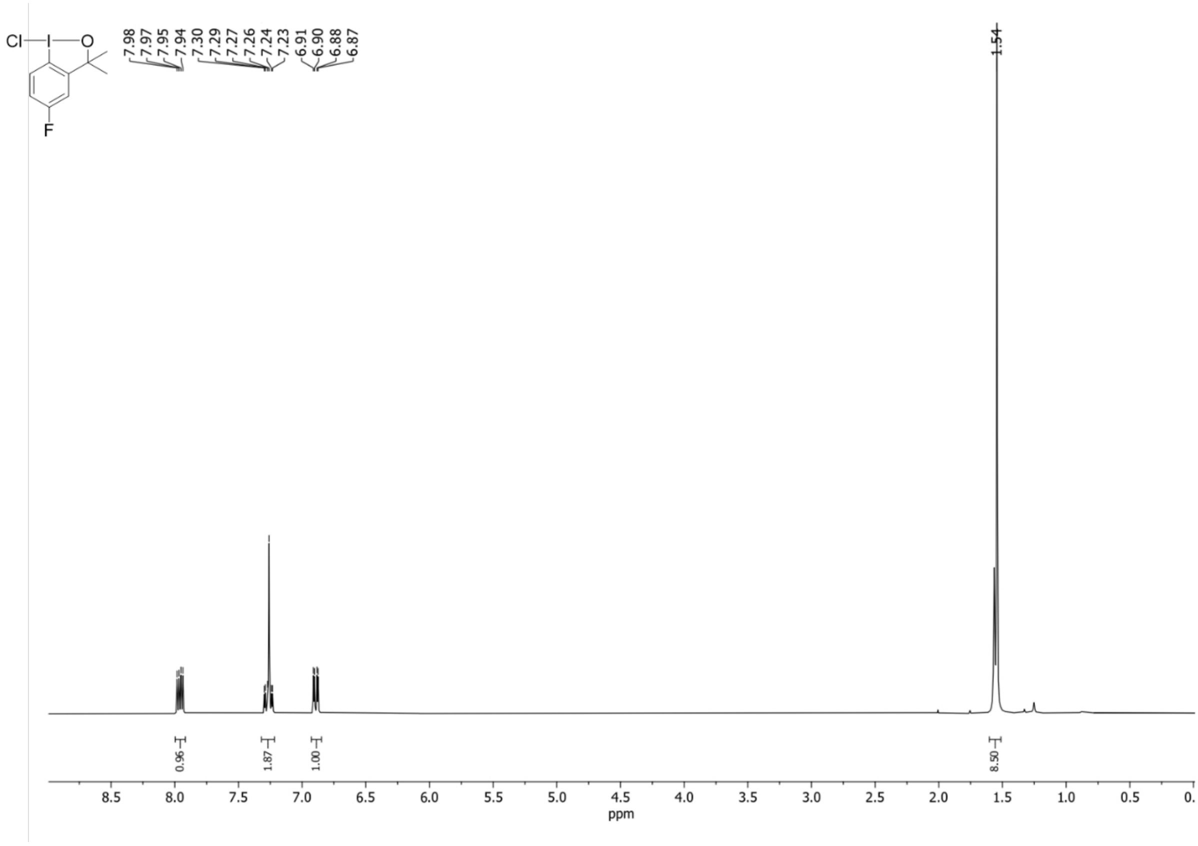

Figure SI-85. ^{1}H-NMR spectrum (300 MHz, $CDCl_3$, 298K) of **Int-5^F**.

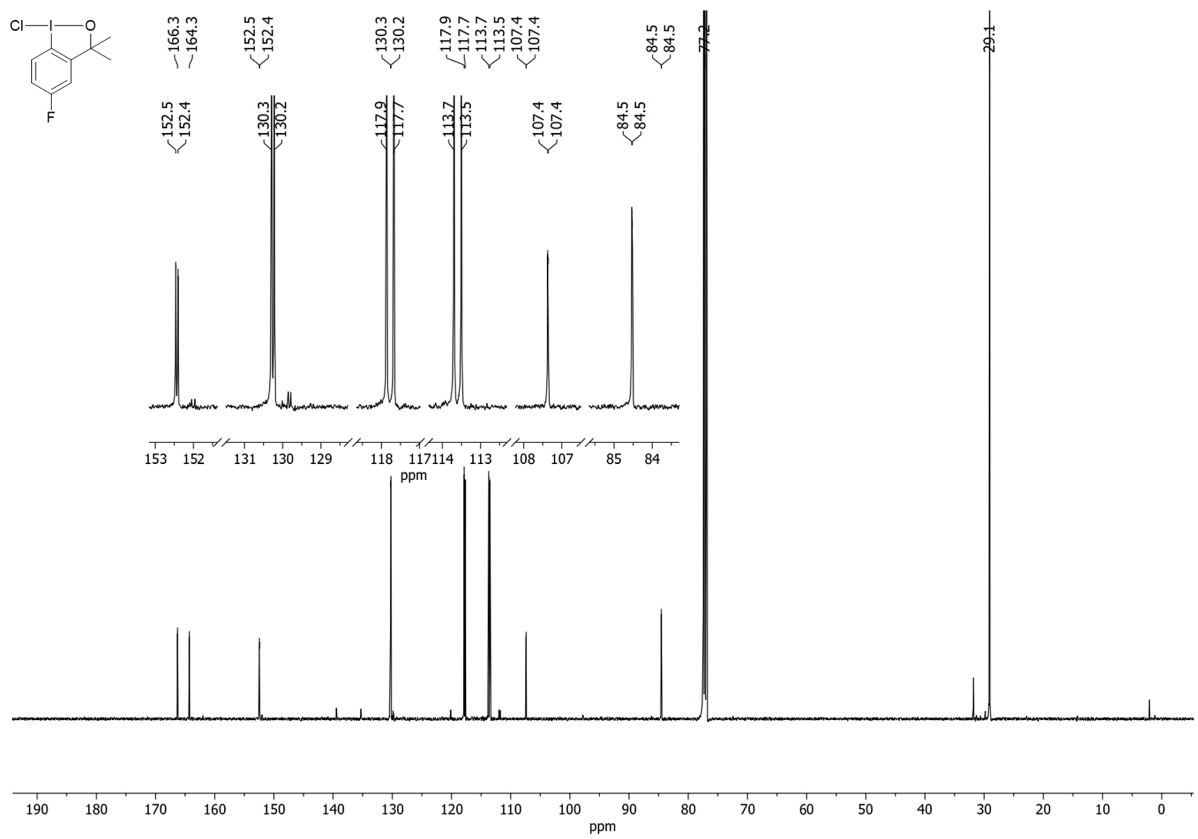

Figure SI-86. ^{13}C-NMR spectrum (126 MHz, $CDCl_3$, 298K) of **Int-5^F**.

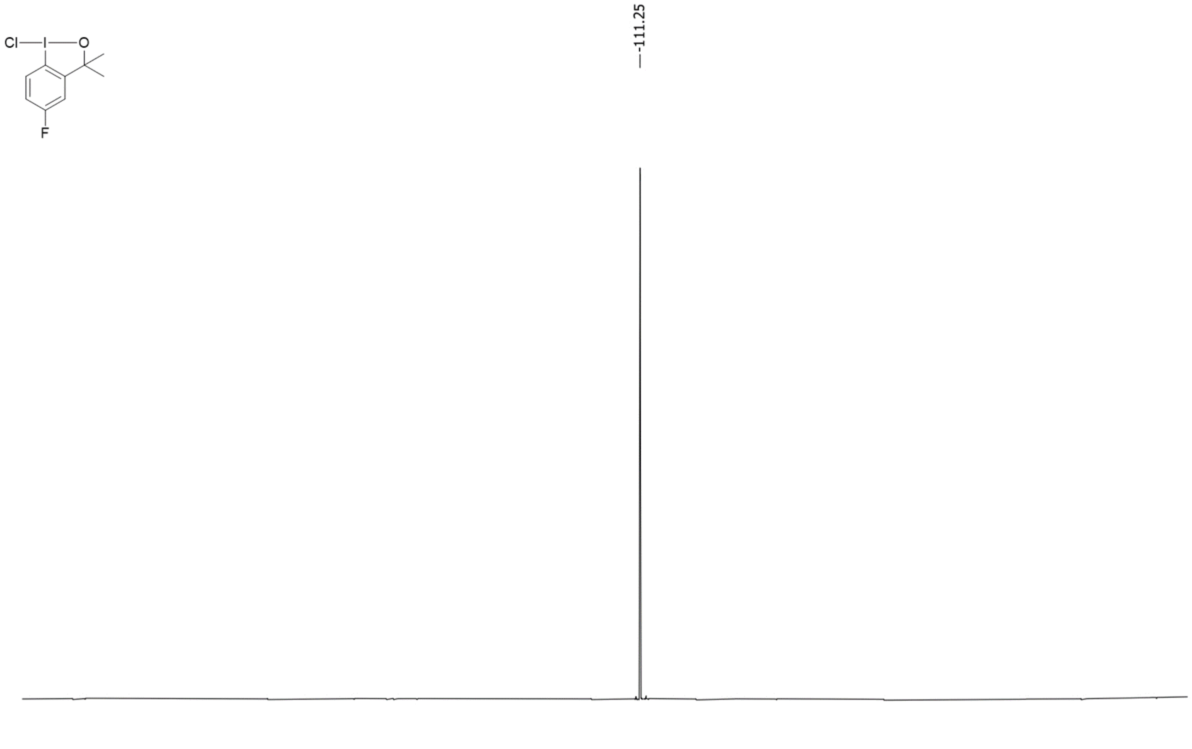

Figure SI-87. ^{19}F-NMR spectrum (282 MHz, $CDCl_3$, 298K) of **Int-5^F**.

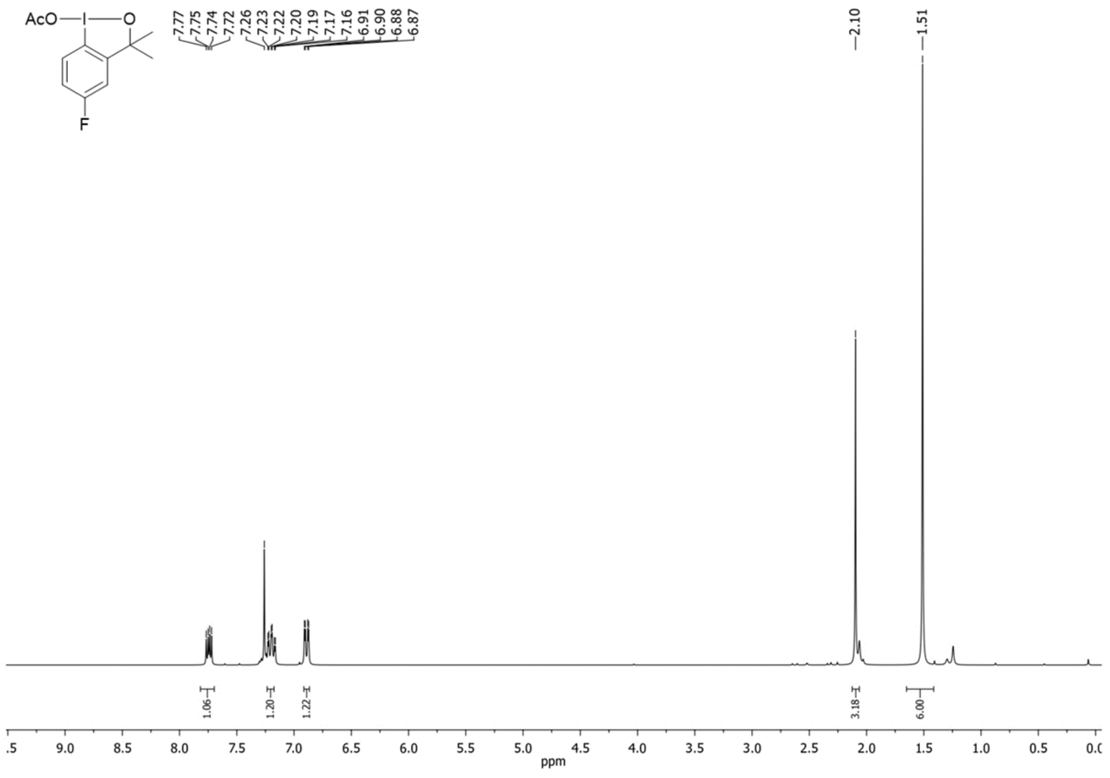

Figure SI-88. ^{1}H-NMR spectrum (300 MHz, $CDCl_3$, 298K) of **Int-6^{F}**.

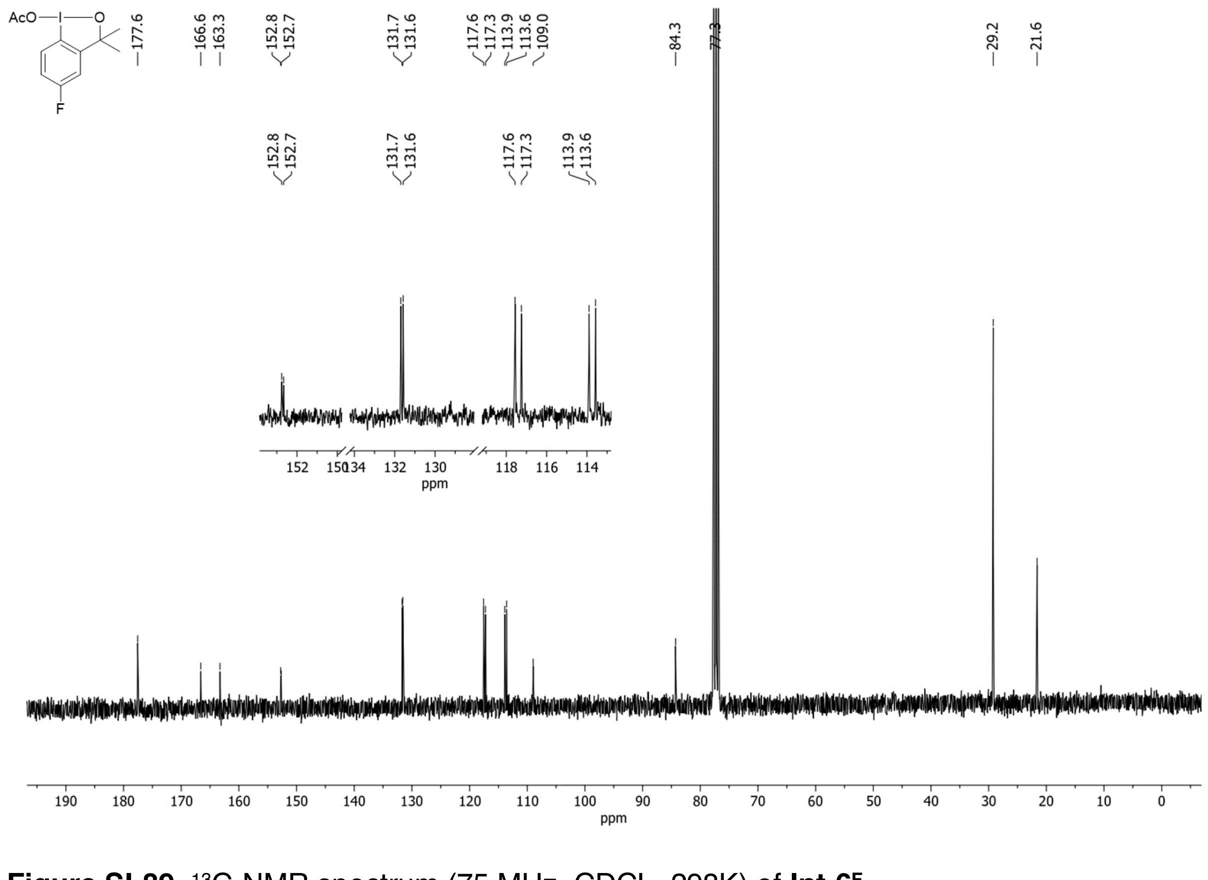

Figure SI-89. ^{13}C-NMR spectrum (75 MHz, $CDCl_3$, 298K) of **Int-6^{F}**.

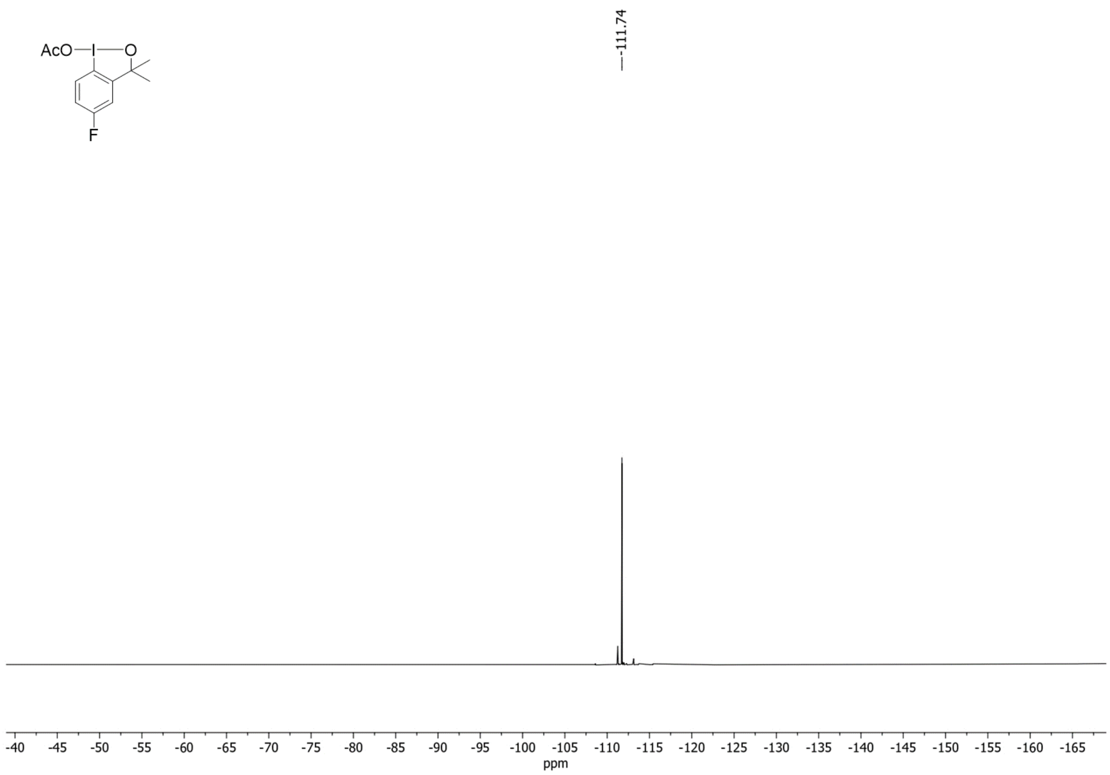

Figure SI-90. ^{19}F-NMR spectrum (282 MHz, $CDCl_3$, 298K) of **Int-6^{F}**.

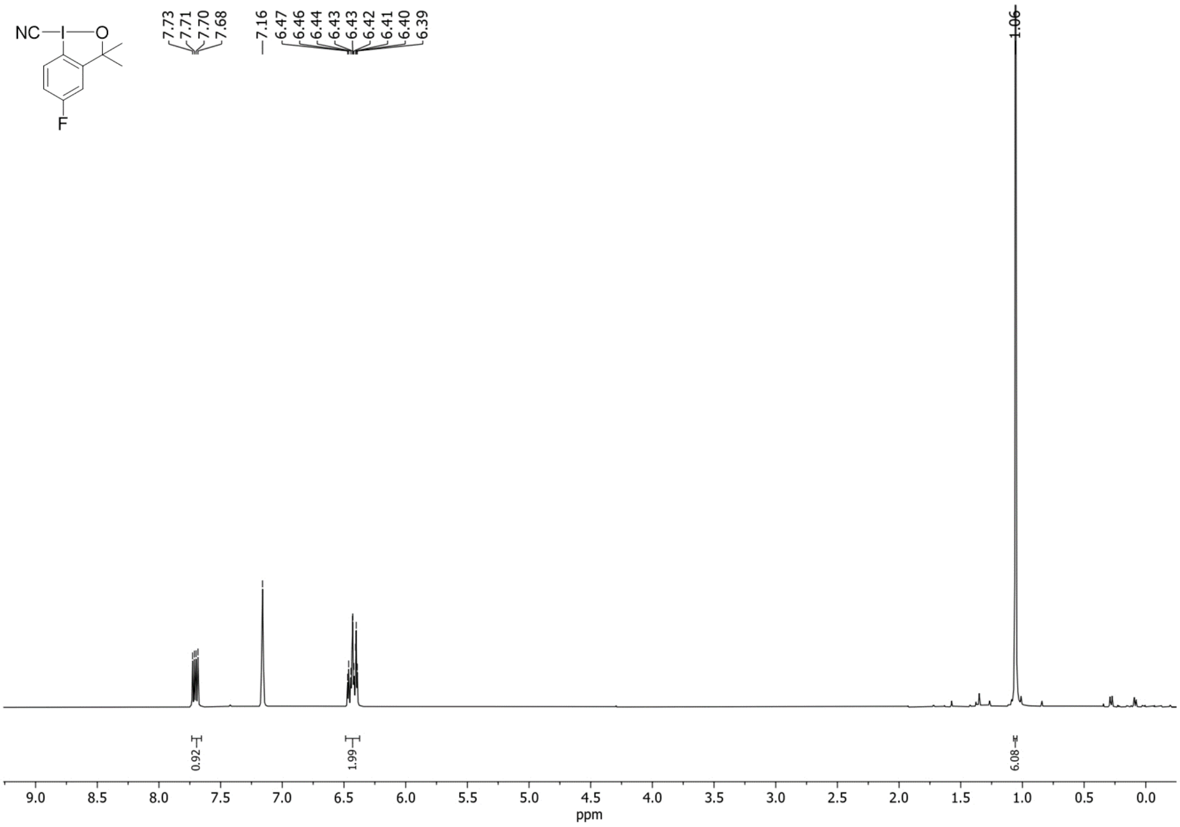

Figure SI-91. ^{1}H-NMR spectrum (300 MHz, C_6D_6, 298K) of **CDBX^{F}**.

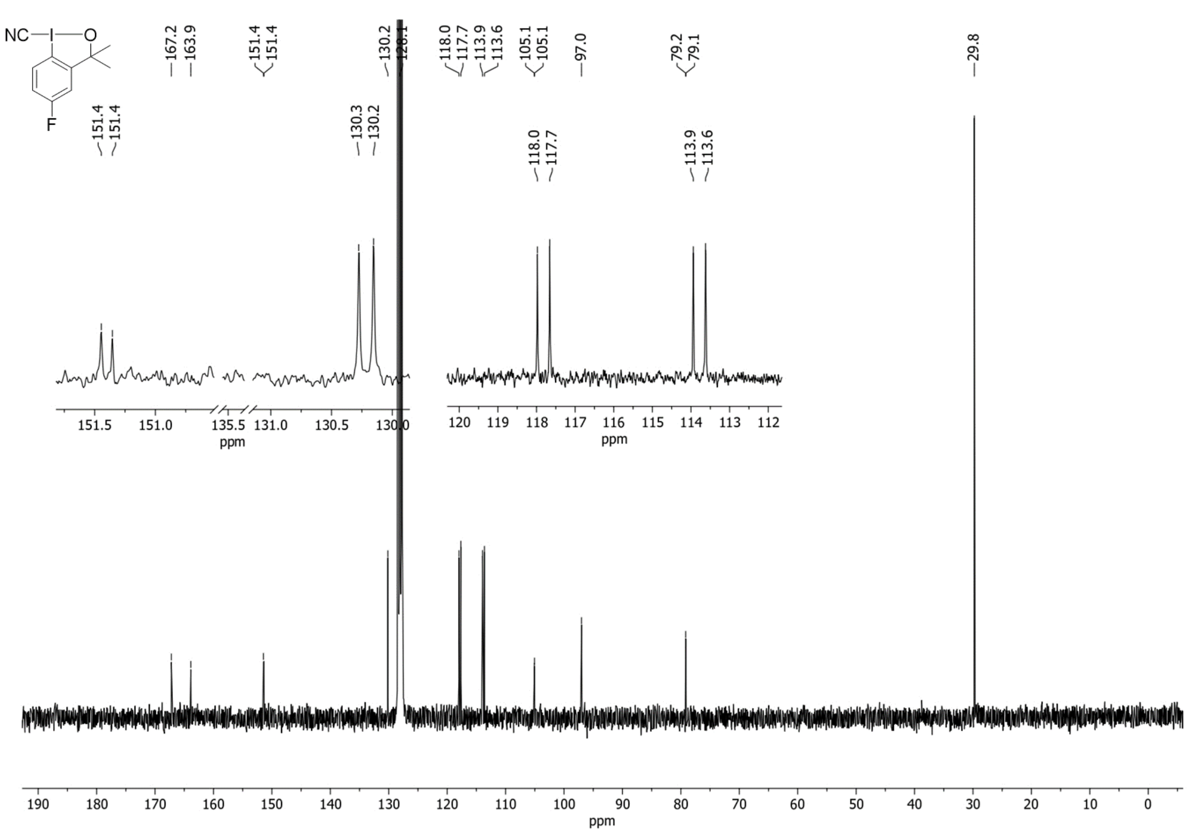

Figure SI-92. ^{13}C-NMR spectrum (75 MHz, C_6D_6, 298K) of **CDBX^F**.

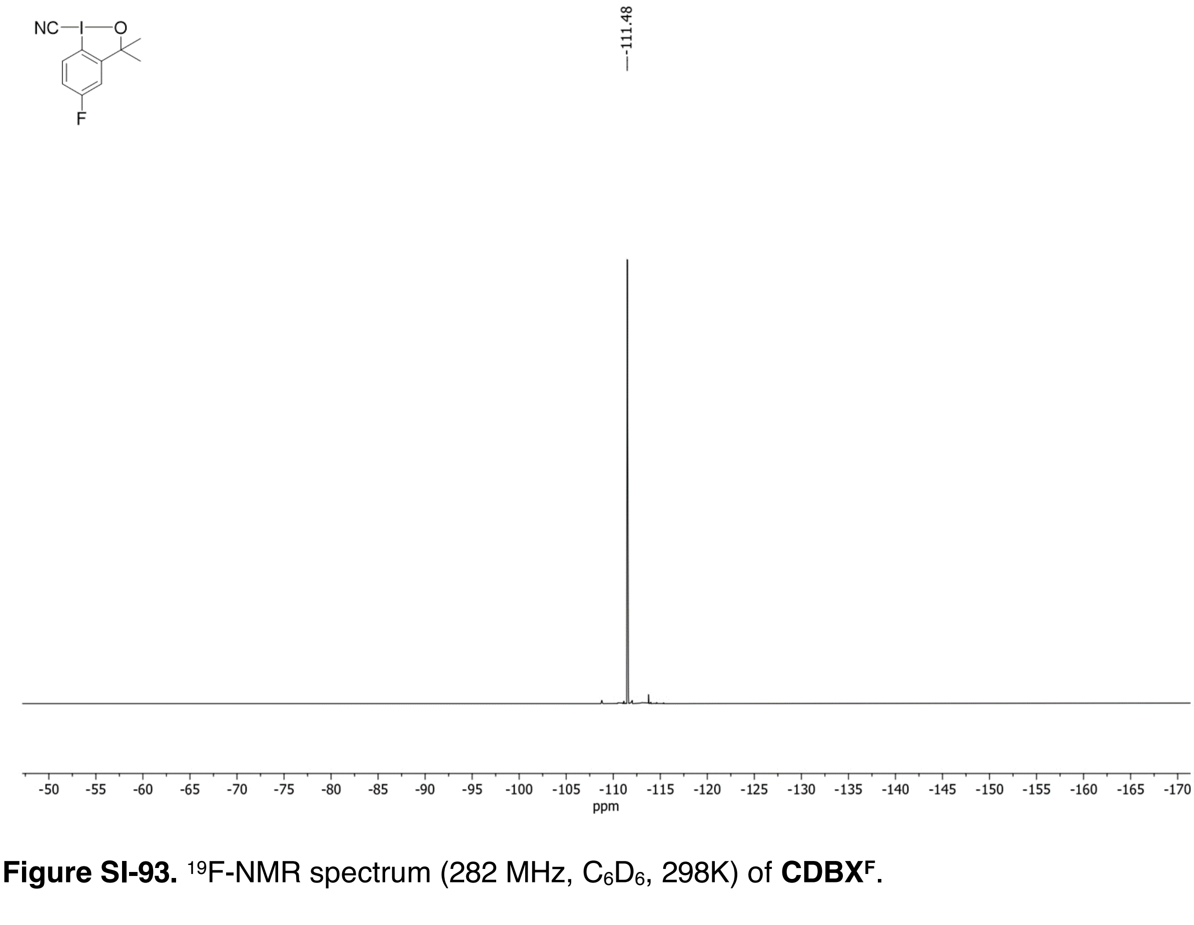

Figure SI-93. ^{19}F-NMR spectrum (282 MHz, C_6D_6, 298K) of **CDBX^F**.

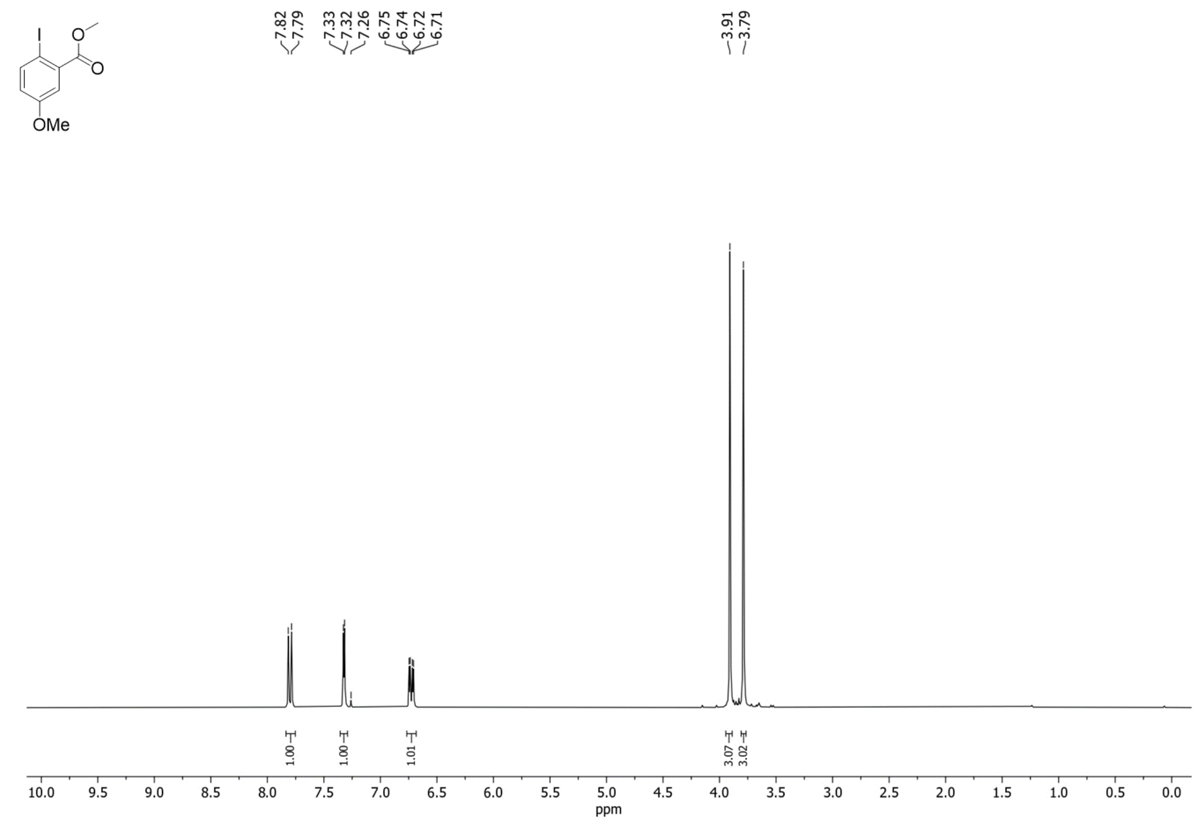

Figure SI-94. ^{1}H-NMR spectrum (300 MHz, $CDCl_3$, 298K) of **Int-3^{OMe}**.

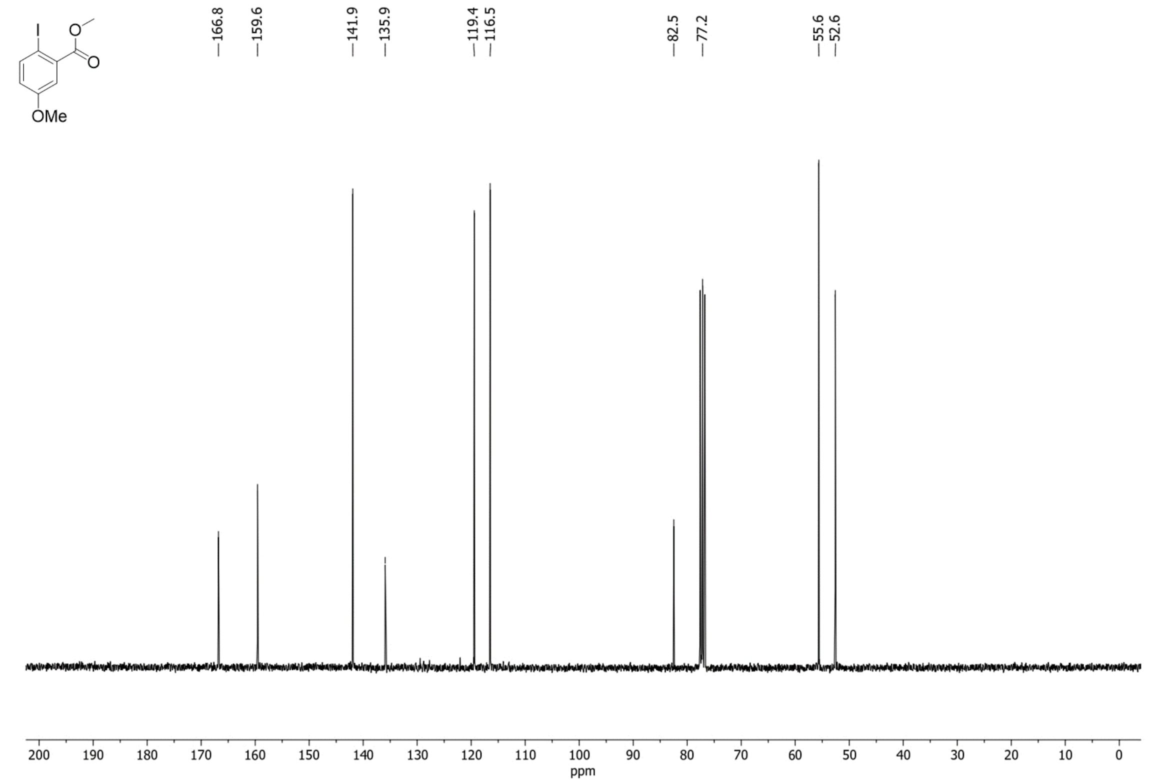

Figure SI-95. ^{13}C-NMR spectrum (75 MHz, $CDCl_3$, 298K) of **Int-3^{OMe}**.

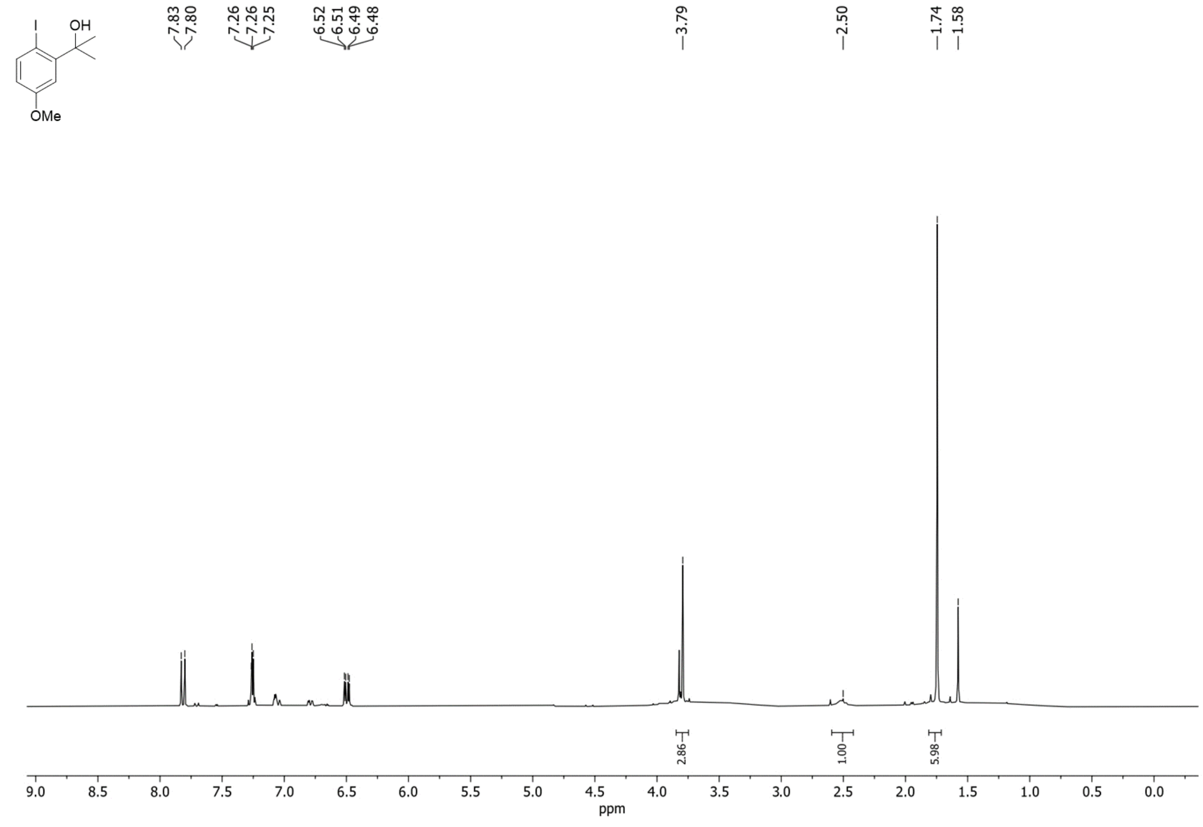

Figure SI-96. ^{1}H-NMR spectrum (300 MHz, $CDCl_3$, 298K) of **Int-4^{OMe}**.

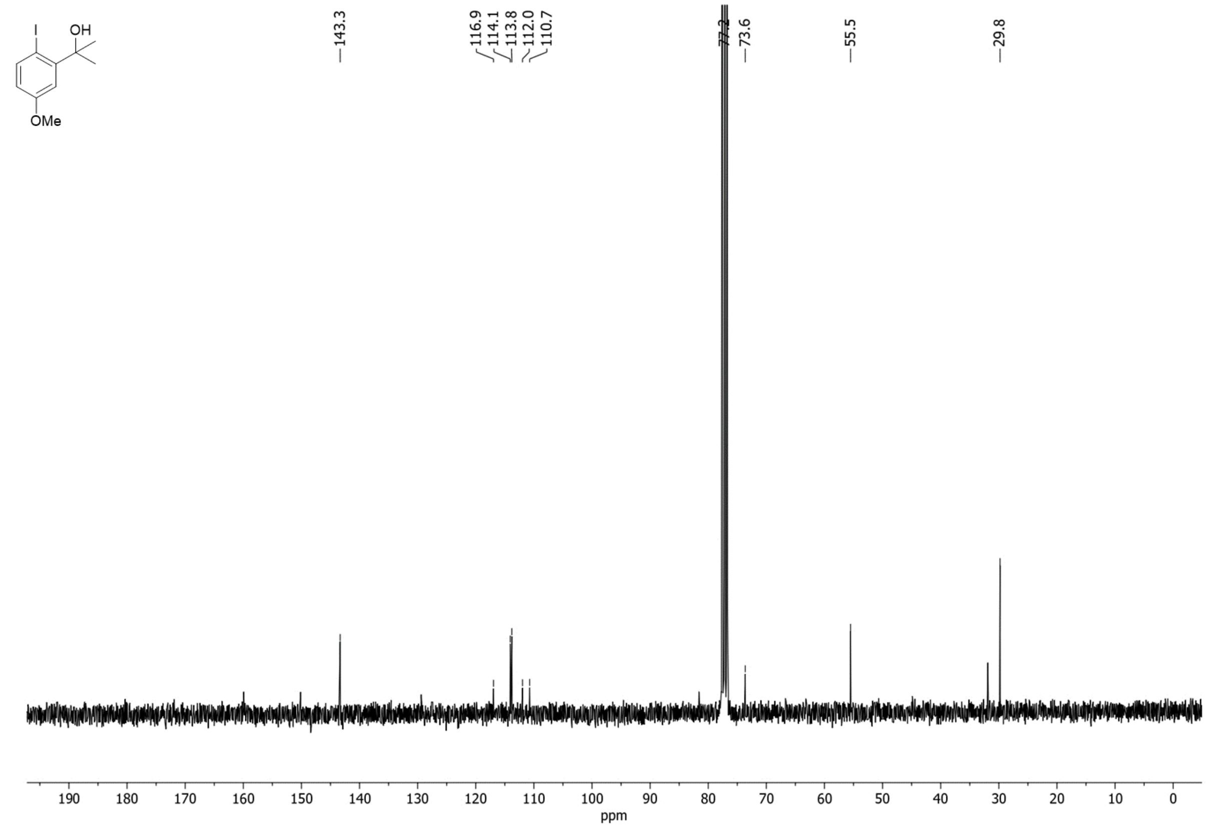

Figure SI-97. ^{13}C-NMR spectrum (75 MHz, $CDCl_3$, 298K) of **Int-4^{OMe}**.

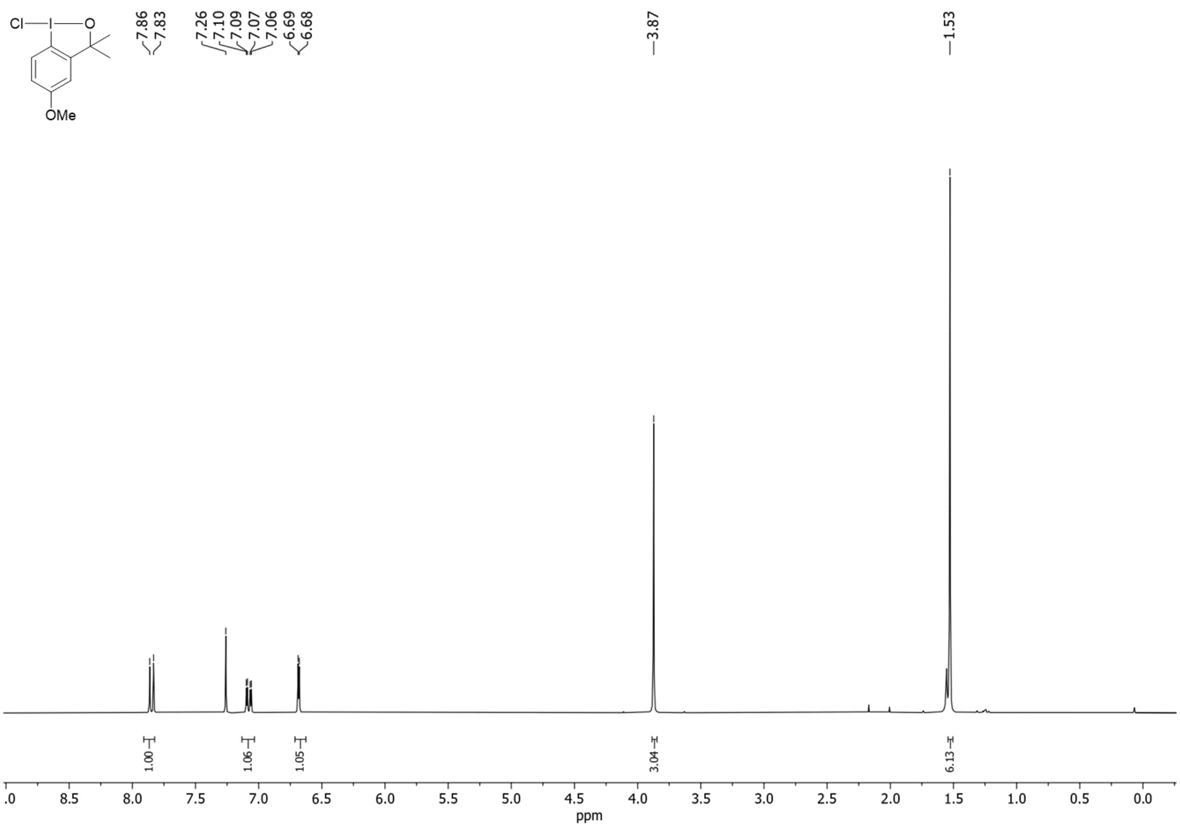

Figure SI-98. ^{1}H-NMR spectrum (300 MHz, $CDCl_3$, 298K) of **Int-5^{OMe}**.

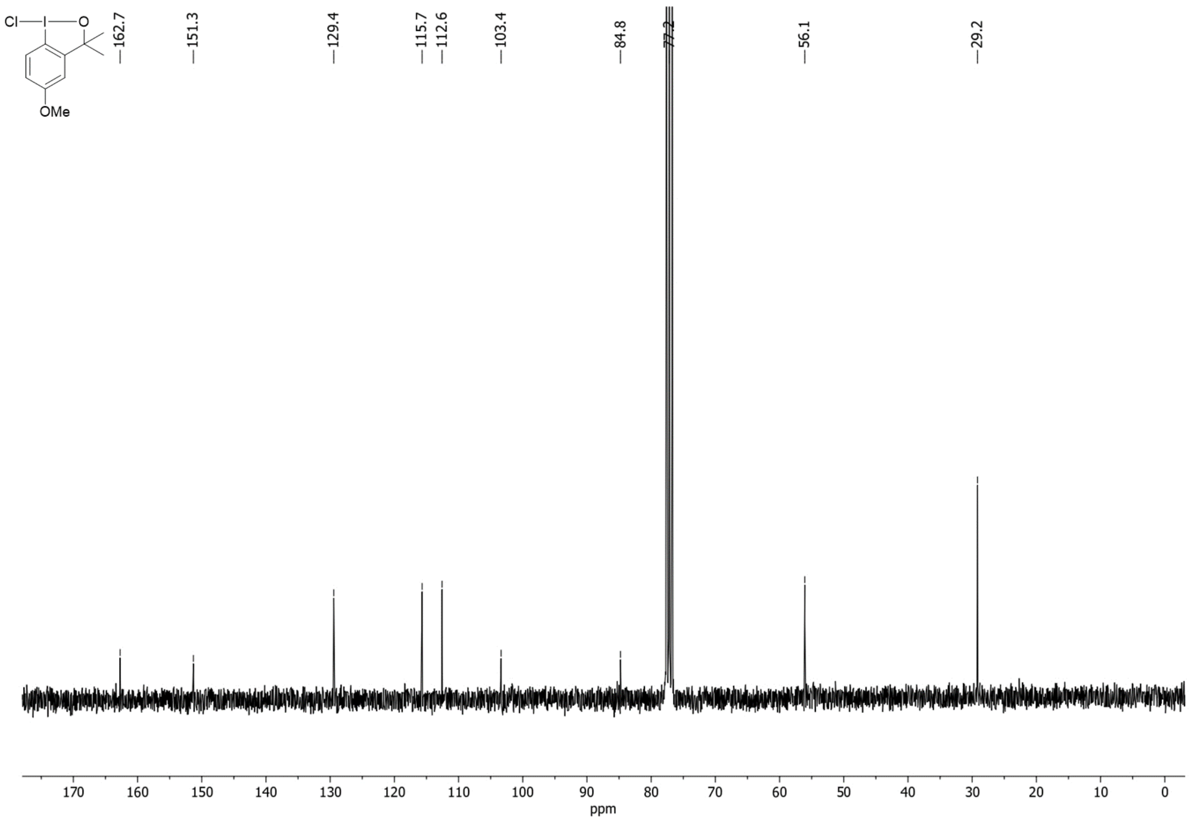

Figure SI-99. ^{13}C-NMR spectrum (75 MHz, $CDCl_3$, 298K) of **Int-5^{OMe}**.

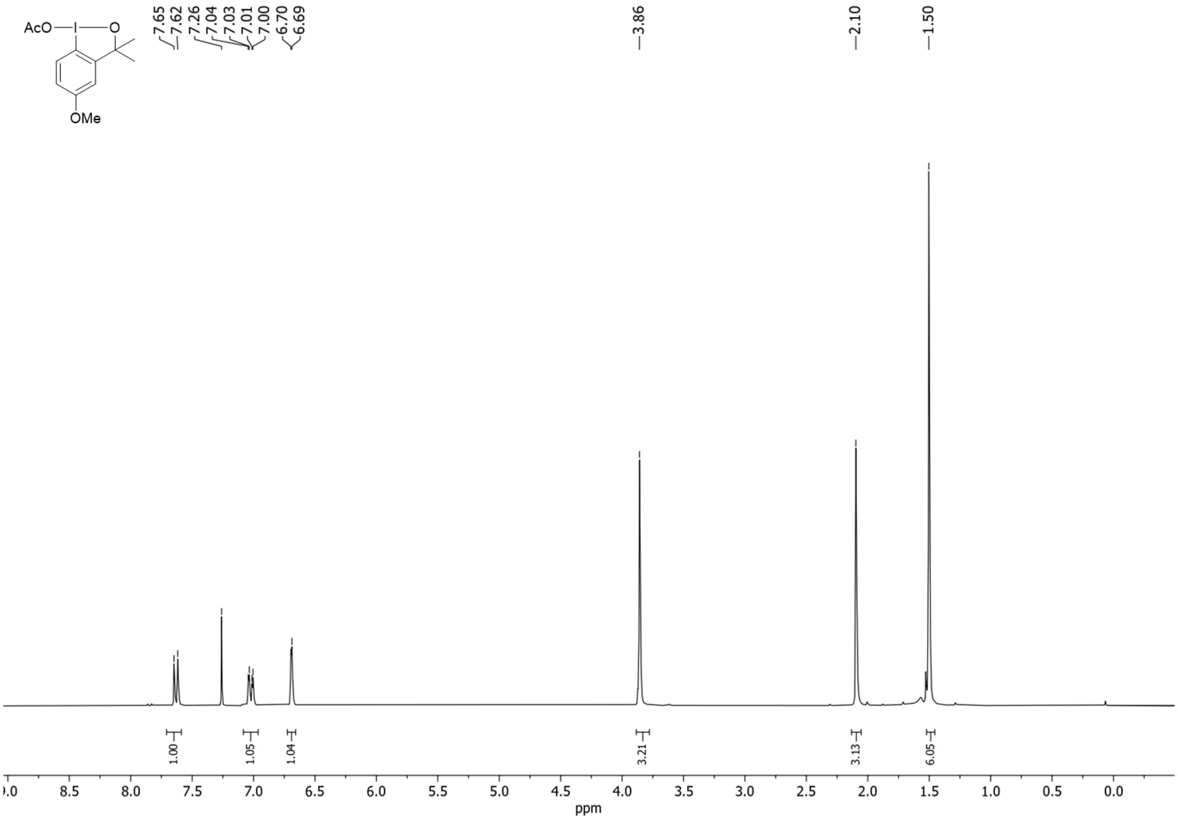

Figure SI-100. ^{1}H-NMR spectrum (300 MHz, $CDCl_3$, 298K) of **Int-6^{OMe}**.

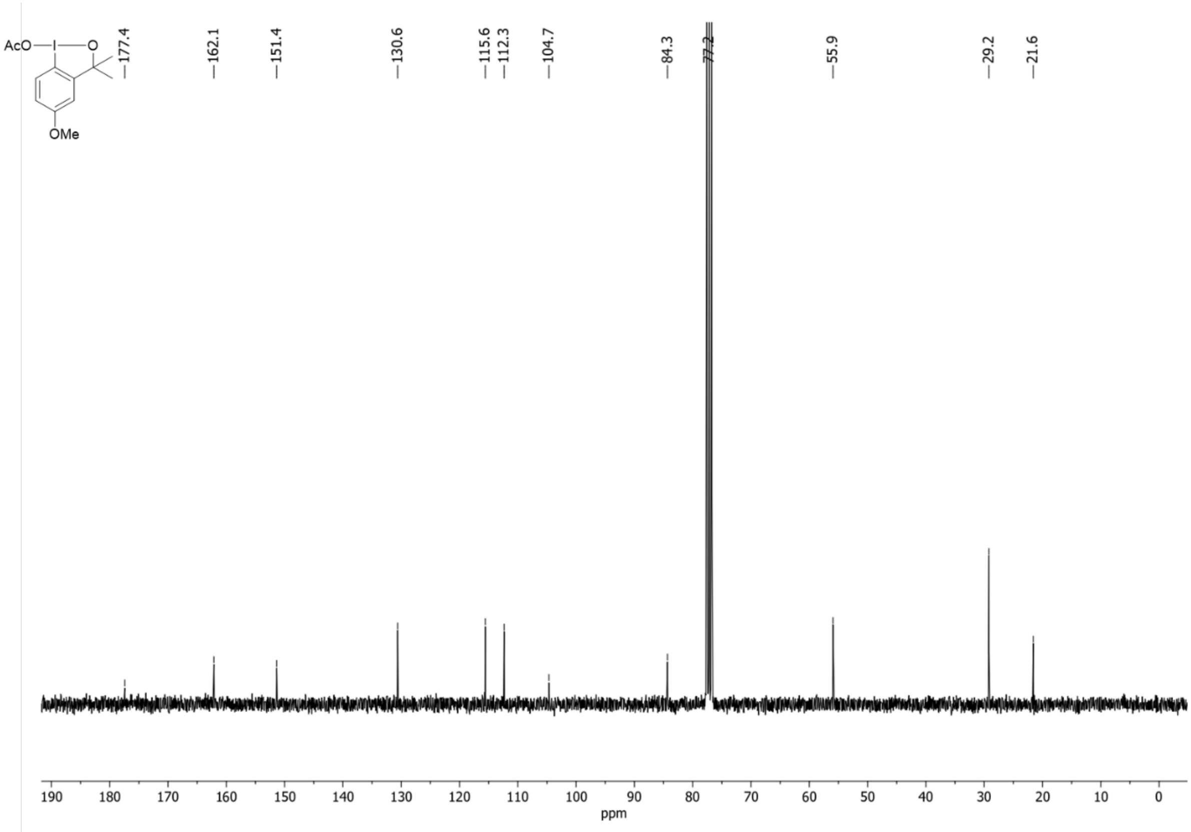

Figure SI-101. ^{13}C-NMR spectrum (75 MHz, $CDCl_3$, 298K) of **Int-6^{OMe}**.

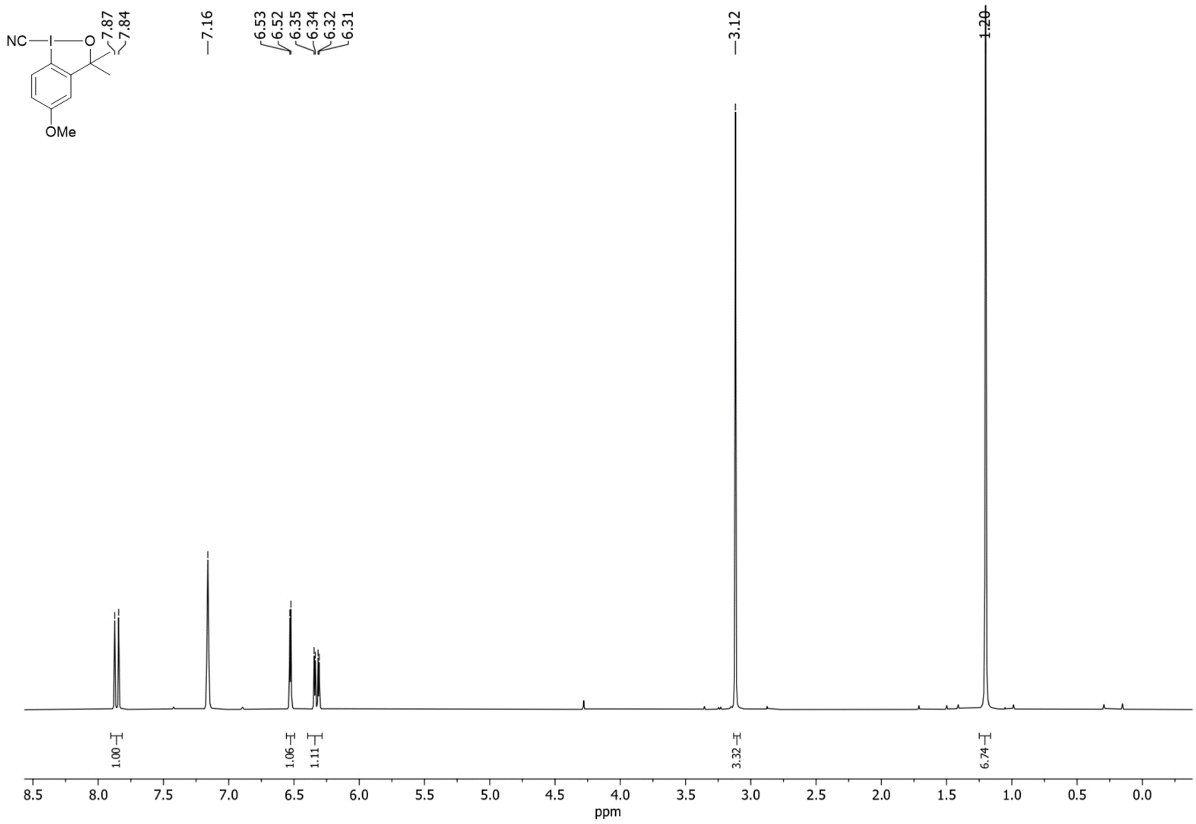

Figure SI-102. ^{1}H-NMR spectrum (300 MHz, C_6D_6, 298K) of **CDBX^{OMe}**.

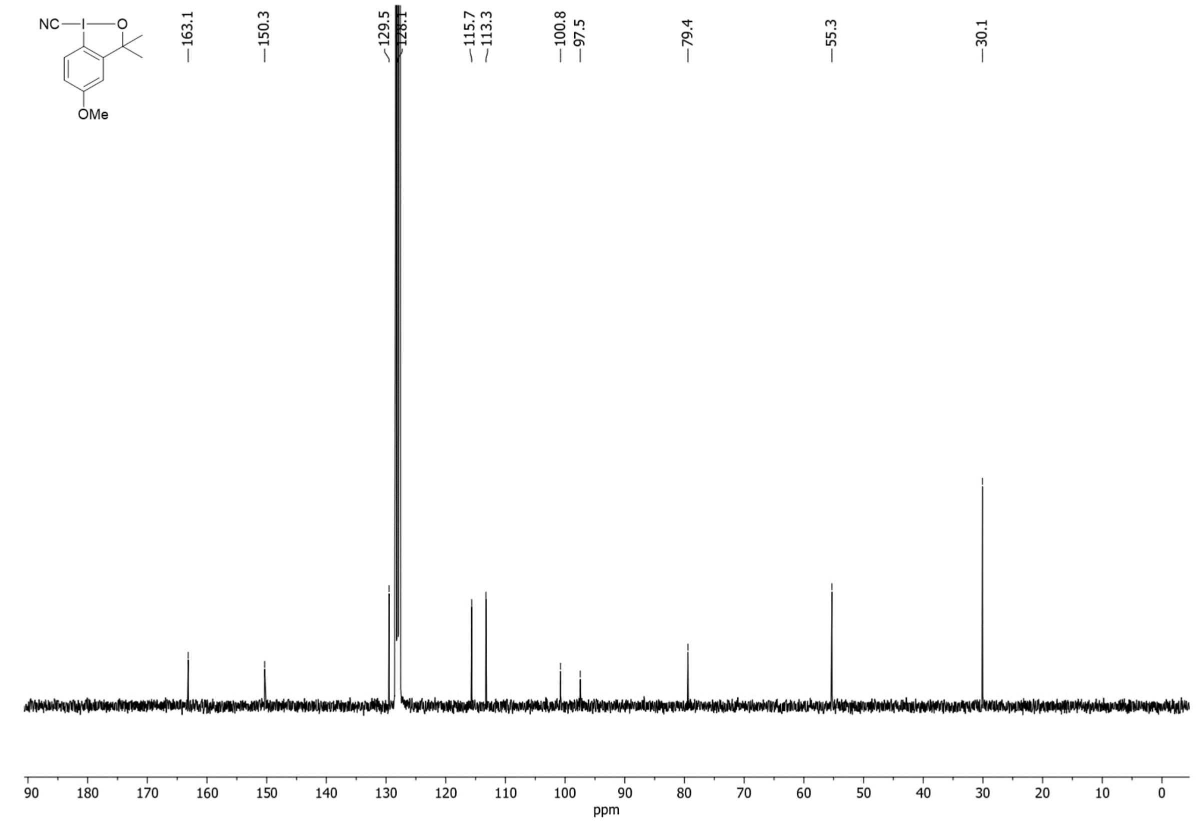

Figure SI-103. ^{13}C-NMR spectrum (75 MHz, C_6D_6, 298K) of **CDBX^{OMe}**.

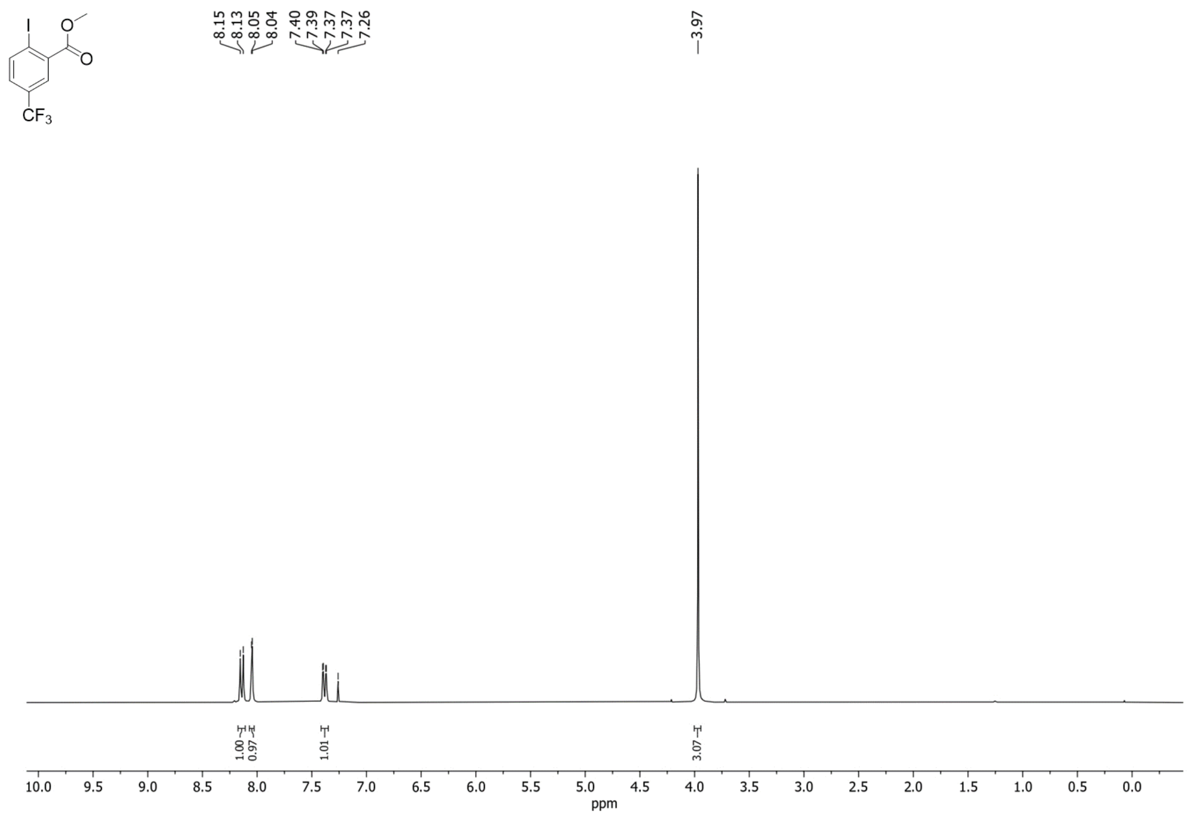

Figure SI-104. ^{1}H-NMR spectrum (300 MHz, $CDCl_3$, 298K) of **Int-3^{CF3}**.

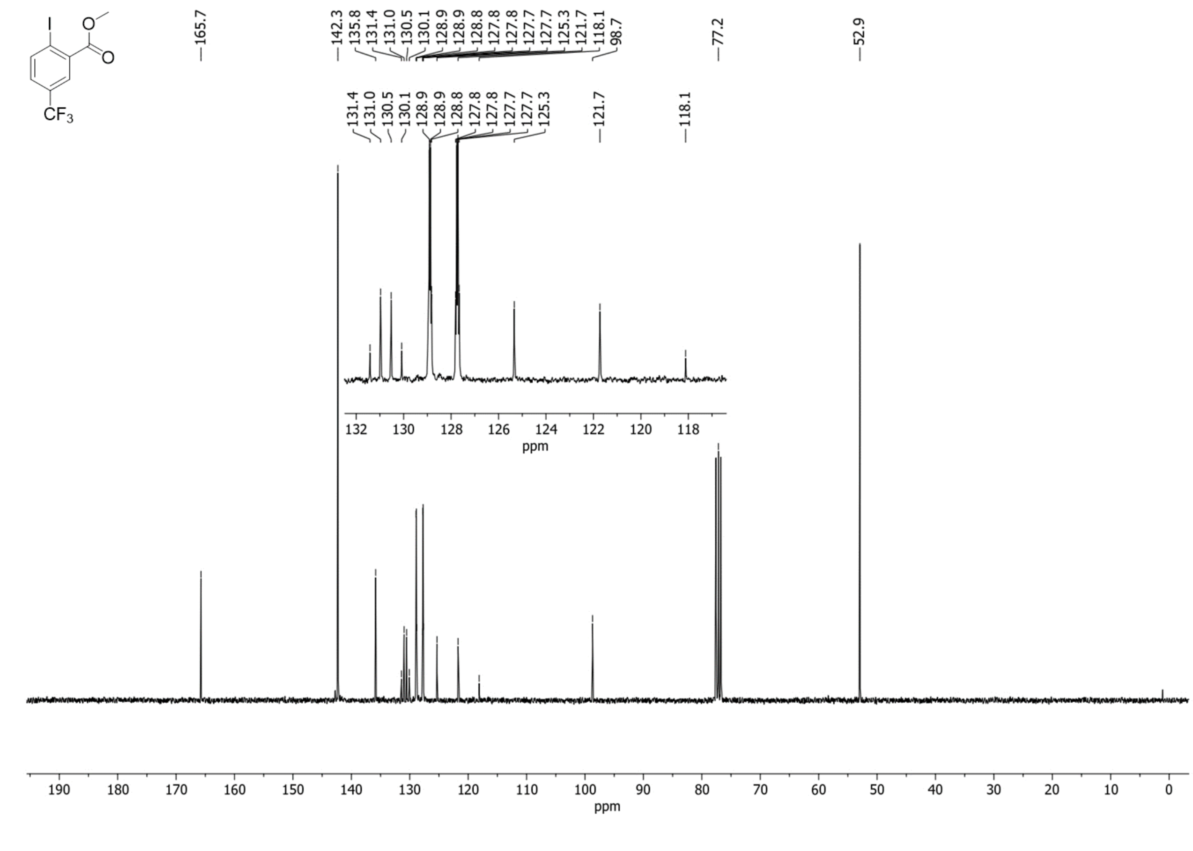

Figure SI-105. ^{13}C-NMR spectrum (75 MHz, $CDCl_3$, 298K) of **Int-3^{CF3}**.

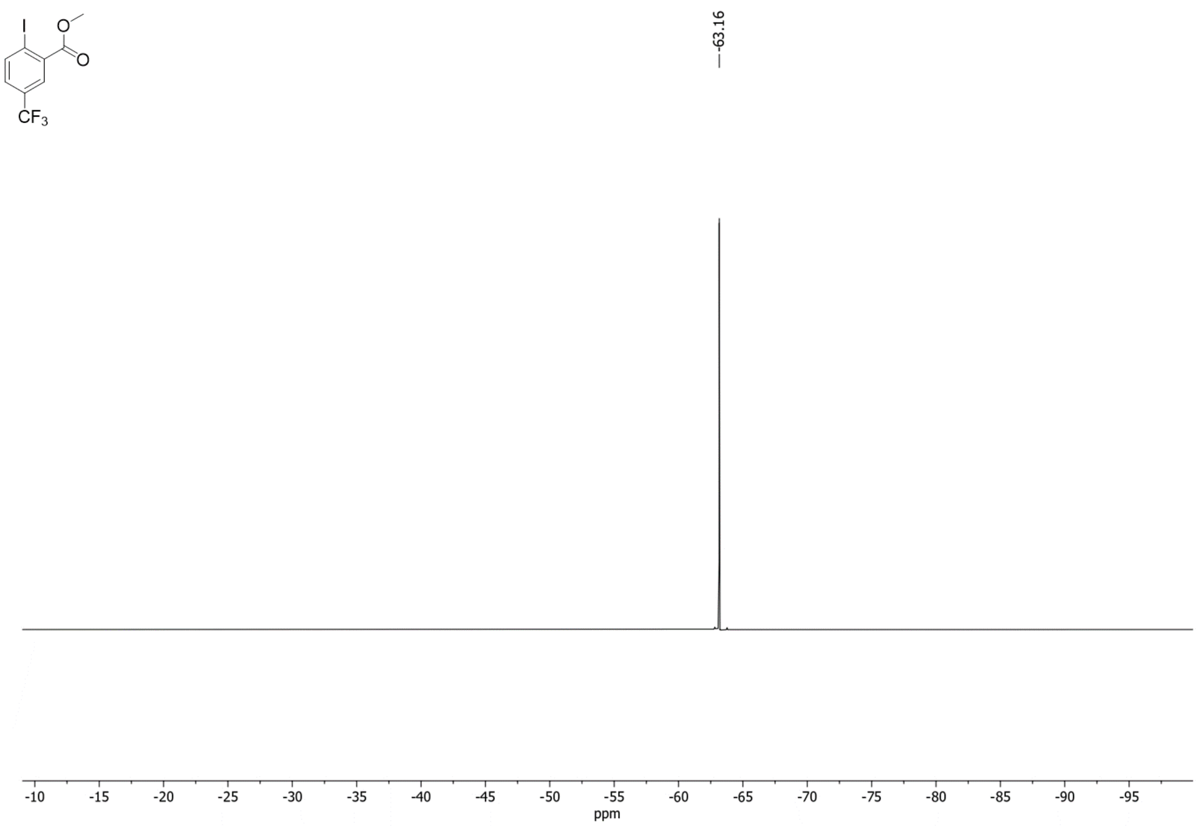

Figure SI-106. ^{19}F-NMR spectrum (282 MHz, $CDCl_3$, 298K) of **Int-3^{CF3}**.

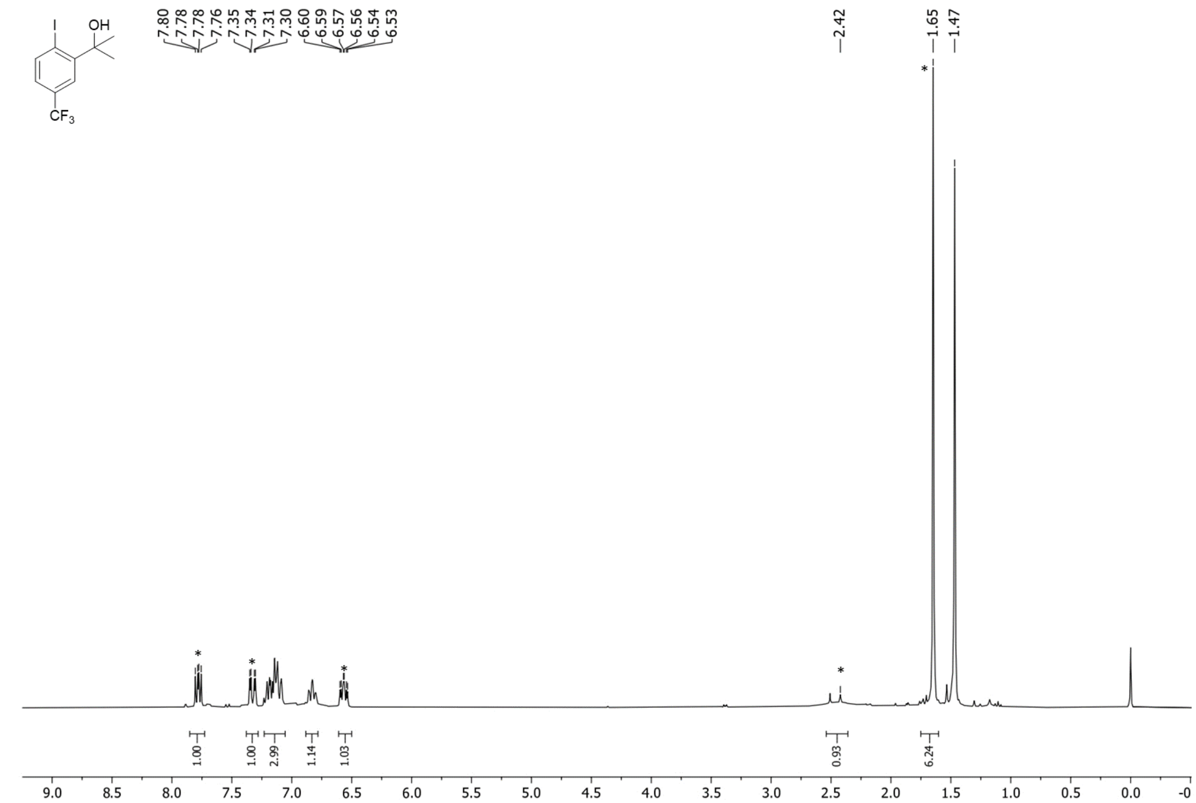

Figure SI-107. ^{1}H-NMR spectrum (300 MHz, $CDCl_3$, 298K) of **Int-4^{CF3}** (indicated with *).

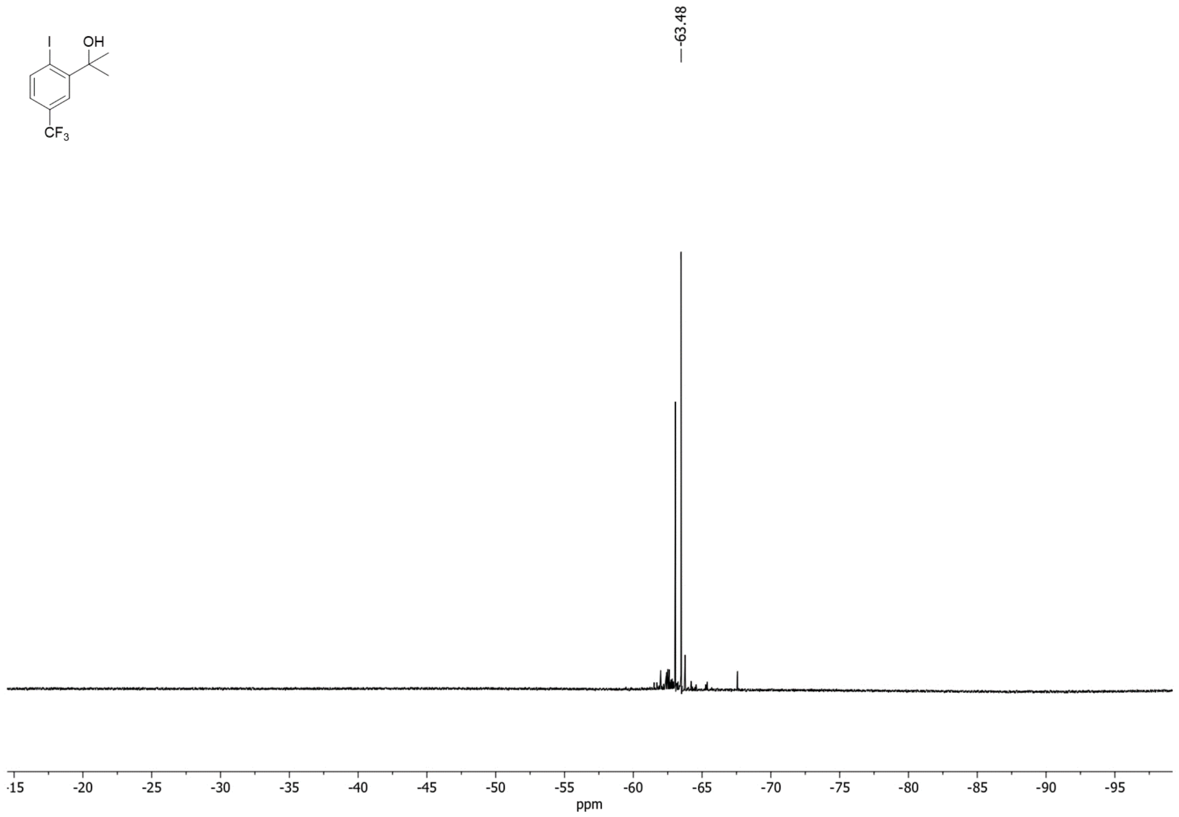

Figure SI-108. ^{19}F-NMR spectrum (282 MHz, $CDCl_3$, 298K) of **Int-4^{CF3}**.

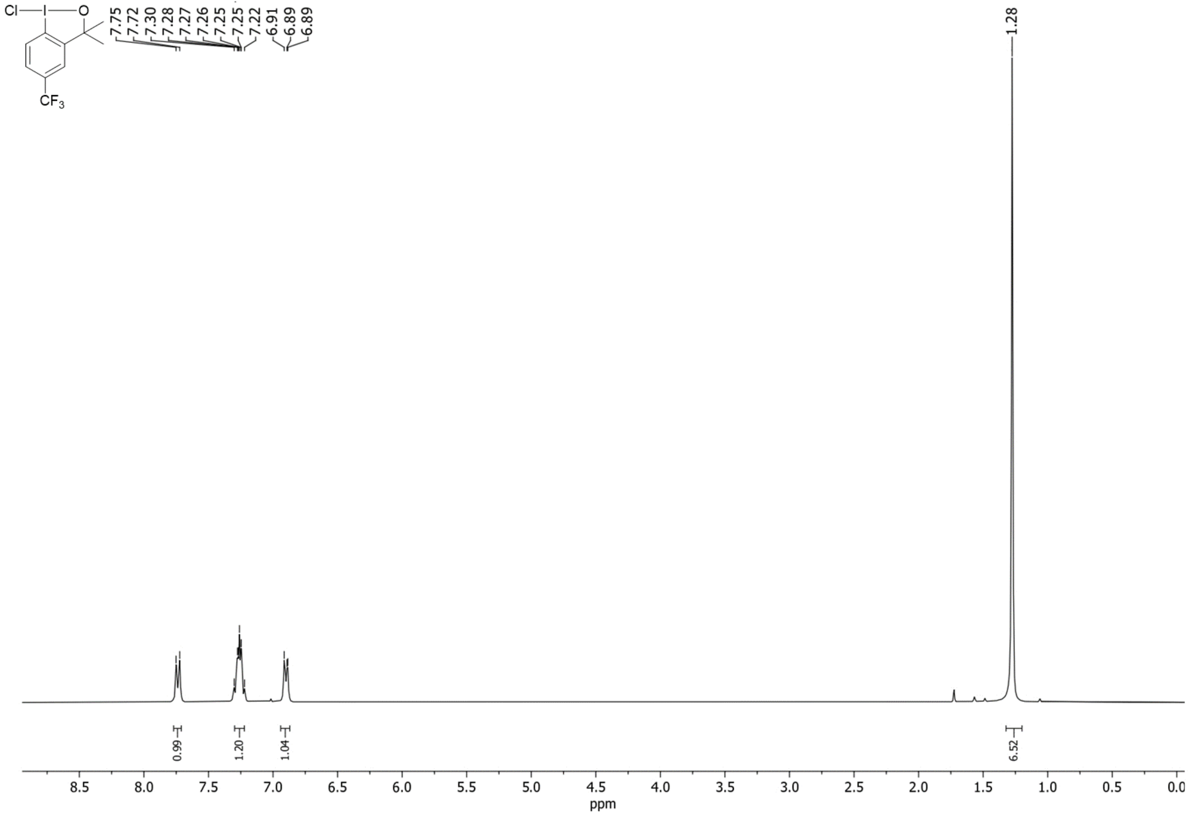

Figure SI-109. ^{1}H-NMR spectrum (300 MHz, $CDCl_3$, 298K) of **Int-5^{CF3}**.

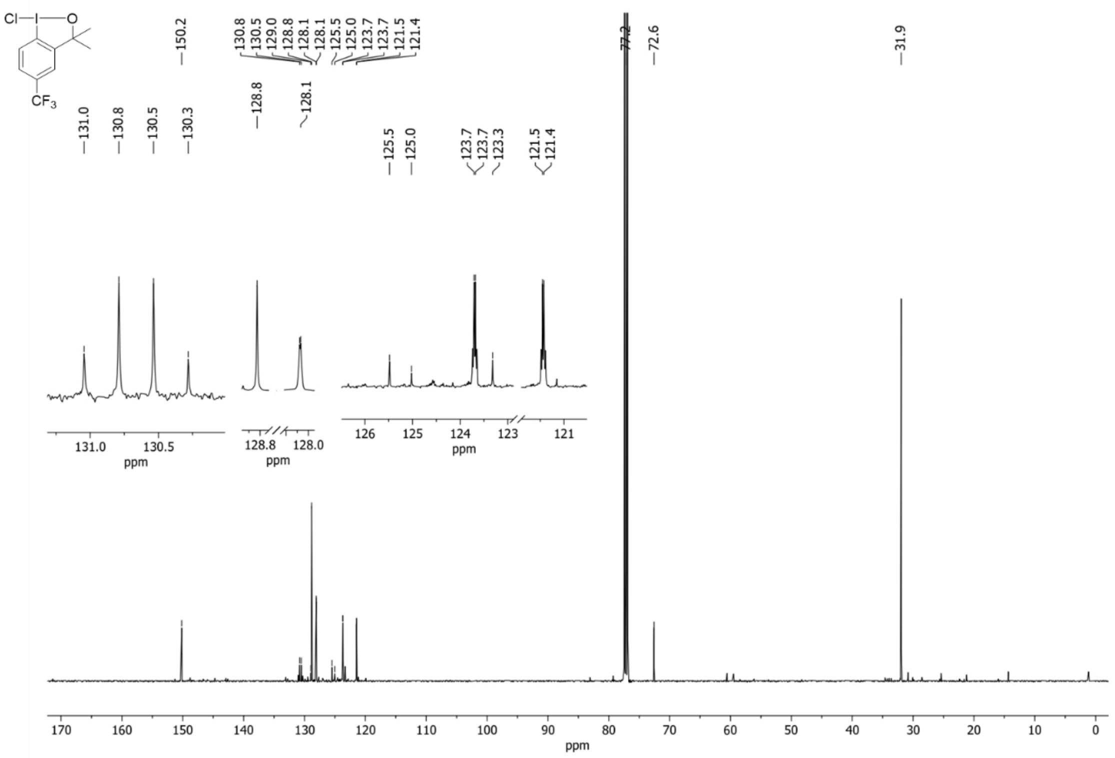

Figure SI-110. ^{13}C-NMR spectrum (126 MHz, $CDCl_3$, 298K) of **Int-5^{CF3}**.

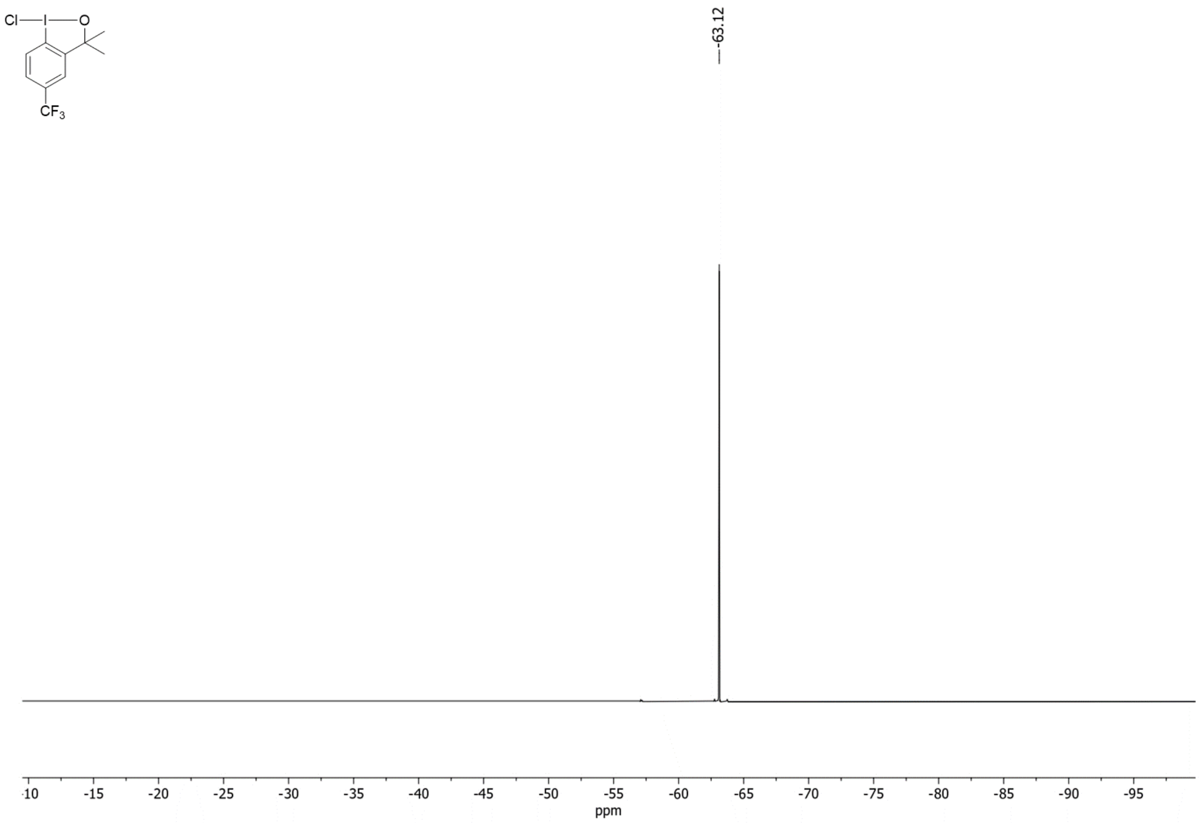

Figure SI-111. ^{19}F-NMR spectrum (282 MHz, $CDCl_3$, 298K) of **Int-5^{CF3}**.

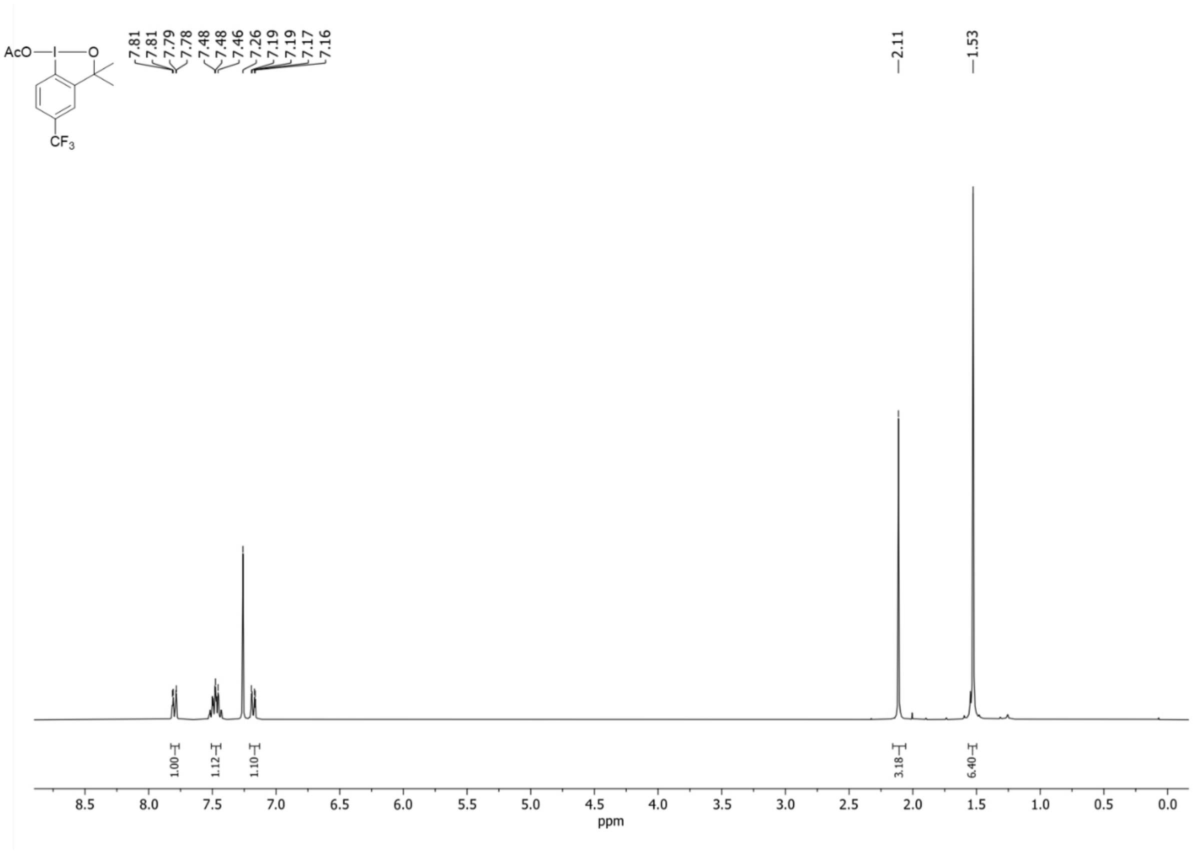

Figure SI-112. ^{1}H-NMR spectrum (300 MHz, $CDCl_3$, 298K) of **Int-6^{CF3}**.

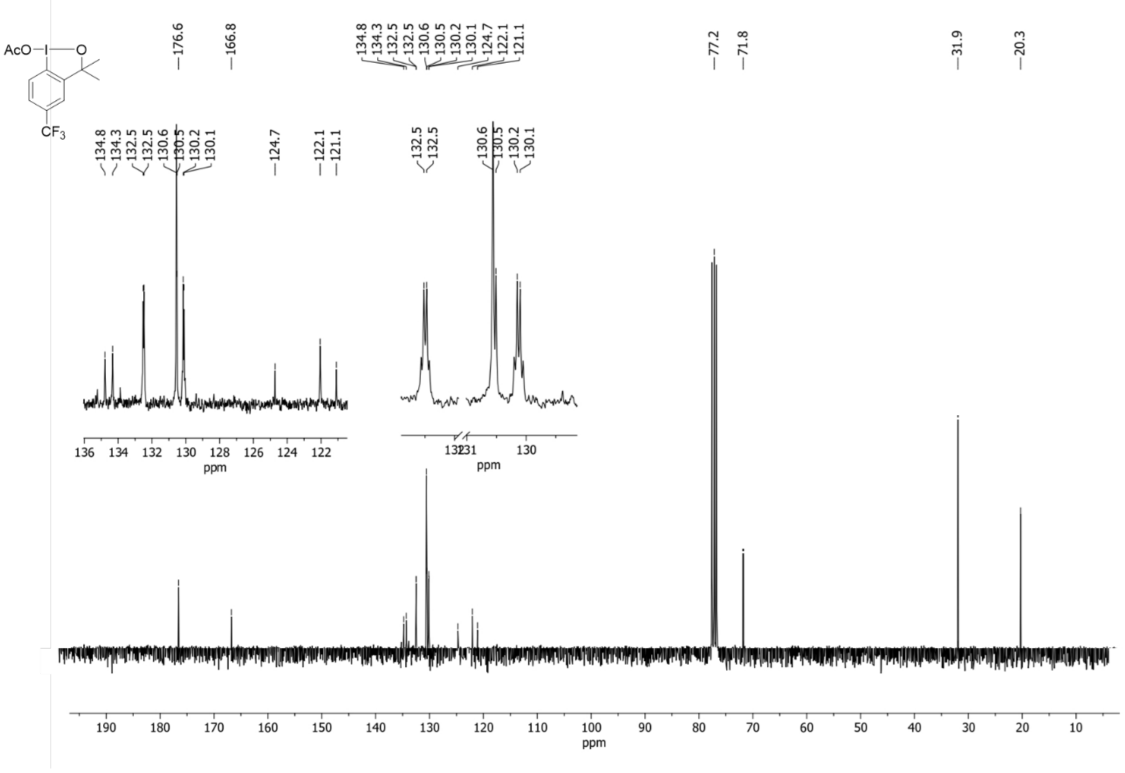

Figure SI-113. ^{13}C-NMR spectrum (126 MHz, $CDCl_3$, 298K) of **Int-6^{CF3}**.

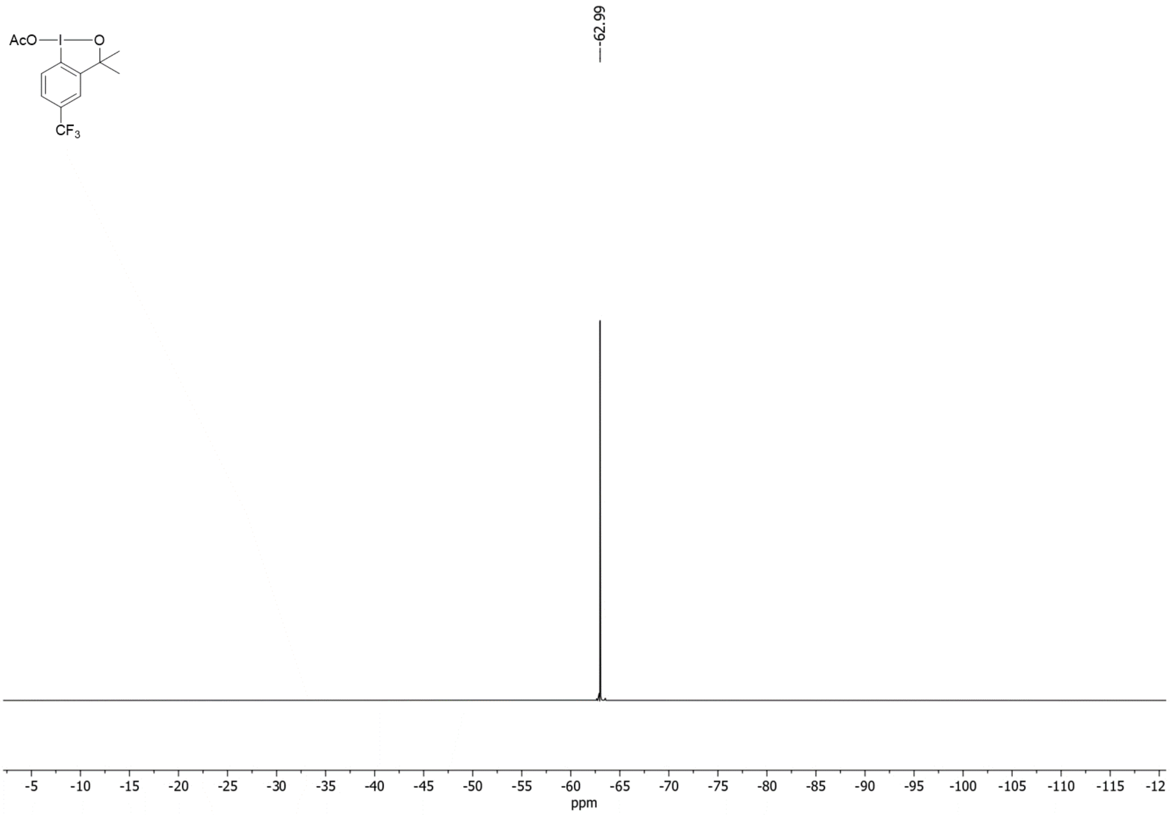

Figure SI-114. ^{19}F-NMR spectrum (282 MHz, $CDCl_3$, 298 K) of **Int-6^{CF3}**.

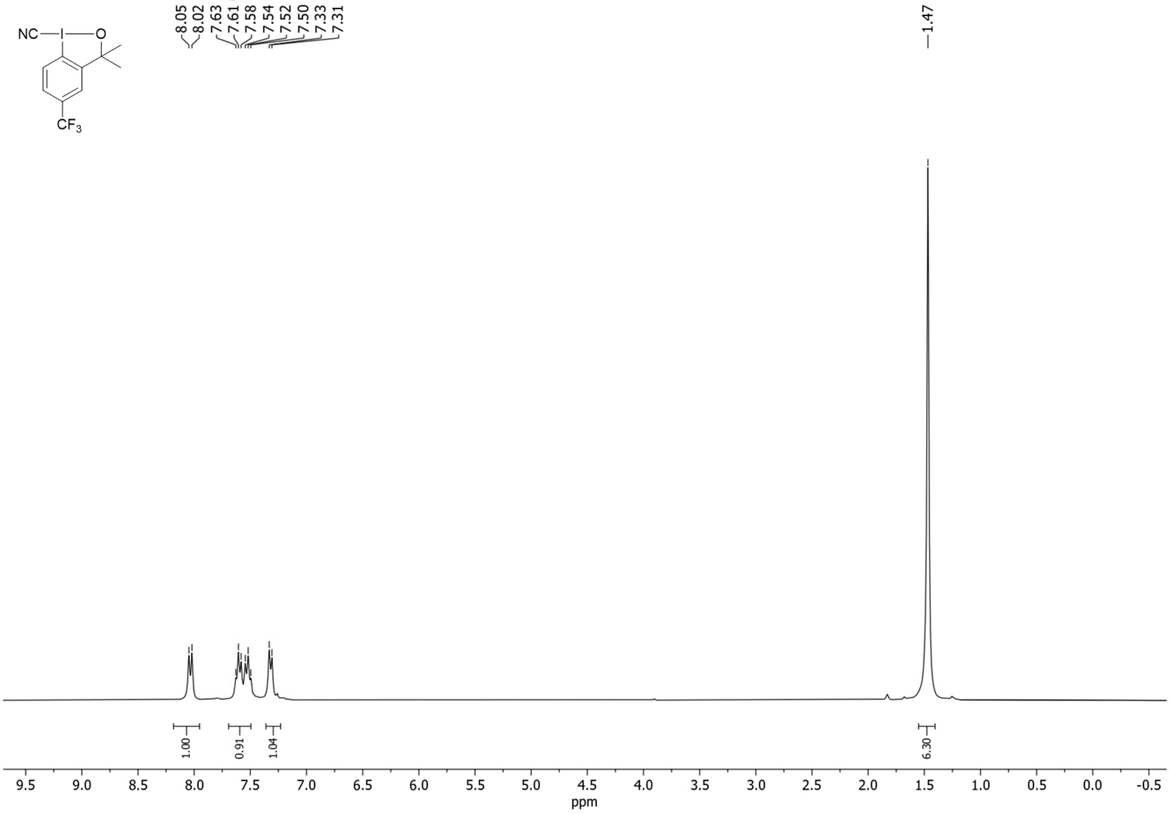

Figure SI-115. ^{1}H-NMR spectrum (300 MHz, $CDCl_3$, 298 K) of **CDBX^{CF3}**.

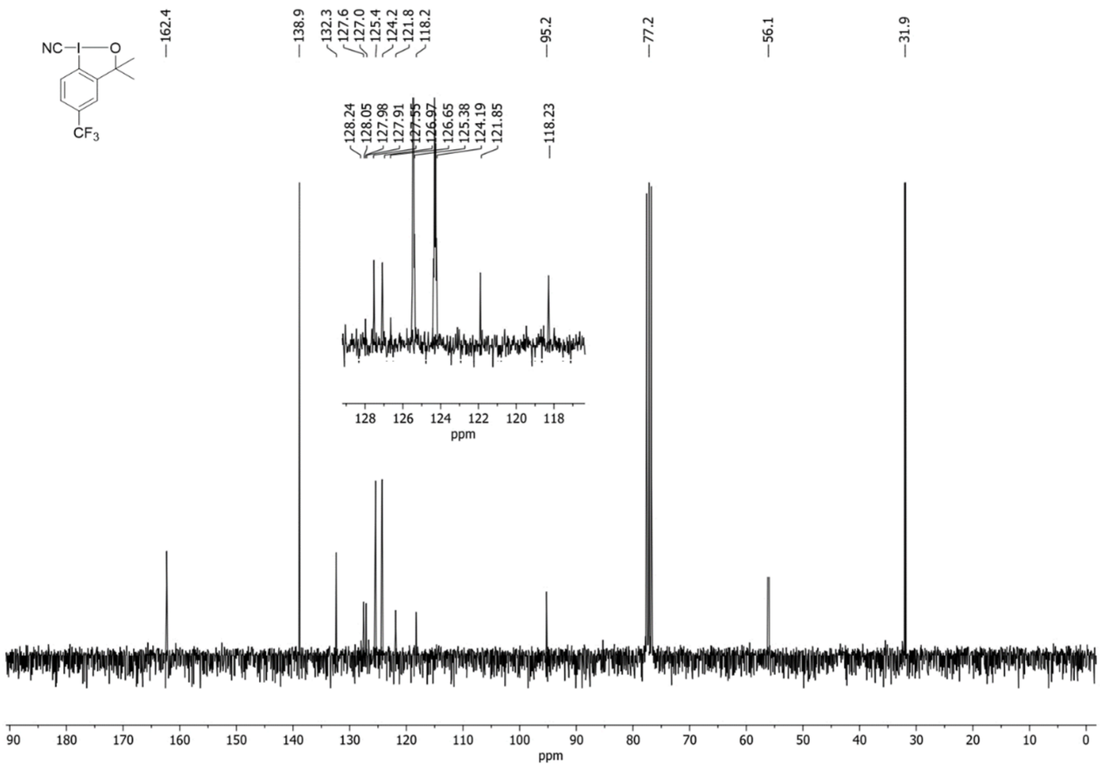

Figure SI-116. ^{13}C-NMR spectrum (126 MHz, $CDCl_3$, 298 K) of **CDBX^{CF3}**.

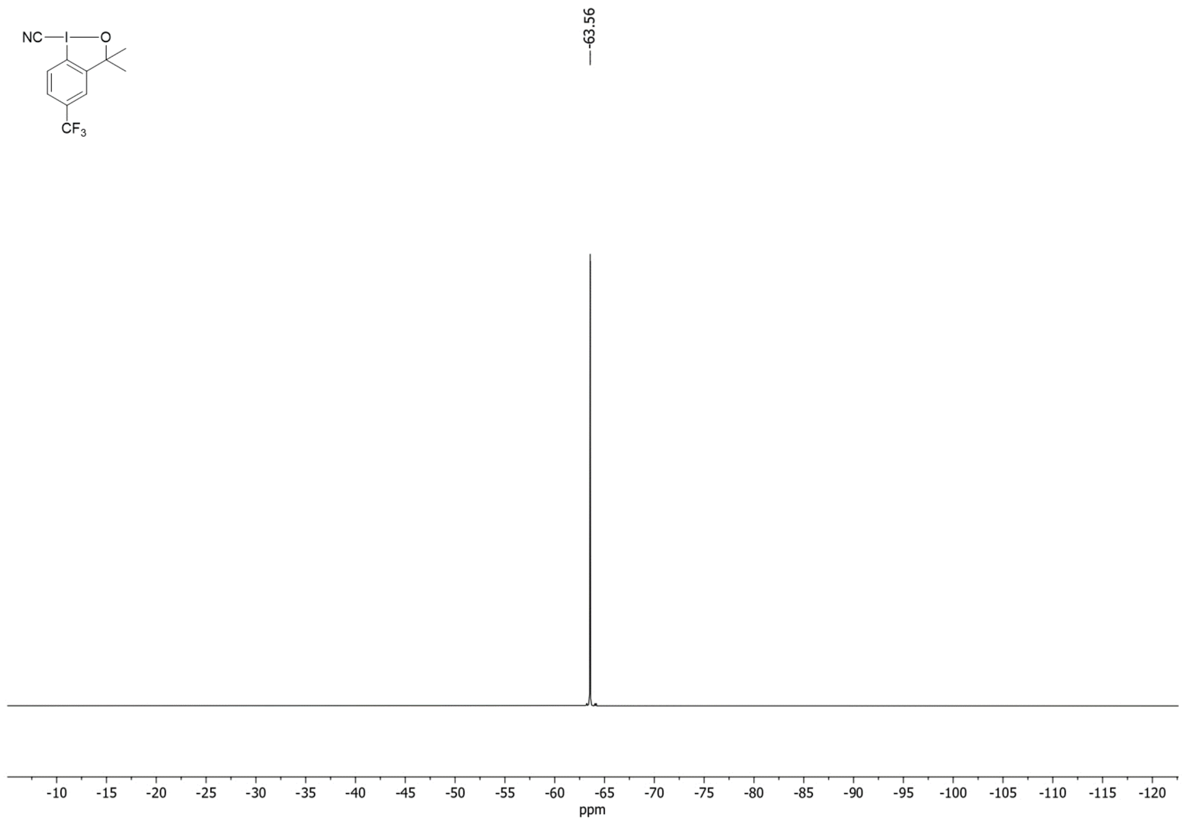

Figure SI-117. ^{19}F-NMR spectrum (282 MHz, $CDCl_3$, 298 K) of **CDBX^{CF3}**.

5. References

[1] S. Stoll, A. Schweiger, *J. Magn. Reson.* **2006**, *178*, 42–45.

[2] R. Aasa, T. Vänngård, *J. Magn. Reson. (1969)* **1975**, *19*, 308–315.

[3] P. Gütlich, E. Bill, A. X. Trautwein, *Mössbauer Spectroscopy and Transition Metal Chemistry: Fundamentals and Applications*, **2011**.

[4] *APEX3, Bruker AXS Inc., Madison, Wisconsin, USA* **2018**.

[5] a) *SADABS, Bruker AXS Inc., Madison, Wisconsin, USA* **2016**; b) L. Krause, R. Herbst-Irmer, G. M. Sheldrick, D. Stalke, *J. Appl. Crystallogr.* **2015**, *48*, 3–10.

[6] a) G. M. Sheldrick, *Acta Crystallogr., Sect. A: Found. Adv.* **2015**, *71*, 3–8; b) G. M. Sheldrick, *Acta Crystallogr., Sect. C: Struct. Chem.* **2015**, *71*, 3–8; c) C. B. Hübschle, G. M. Sheldrick, B. Dittrich, *J. Appl. Crystallogr.* **2011**, *44*, 1281–1284.

[7] D. Kratzert, I. Krossing, *J. Appl. Crystallogr.* **2018**, *51*, 928–934.

[8] *X-Area, STOE & Cie GmbH, Darmstadt, Germany* **2018**.

[9] a) *X-RED32, STOE & Cie GmbH, Darmstadt, Germany* **2018**; b) *LANA - Laue Analyzer, STOE & Cie GmbH, Darmstadt, Germany* **2019**.

[10] a) E. J. Baerends, J. Autschbach, D. Bashford, *et al. ADF2012.01, (2012) SCM, Amsterdam*; b) G. te Velde, F. M. Bickelhaupt, E. J. Baerends, C. Fonseca Guerra, S. J. A. van Gisbergen, J. G. Snijders and T. Ziegler, *J. Comput. Chem.* **2001**, *22*, 931–967.

[11] M. Swart, F. M. Bickelhaupt, *J. Comput. Chem.* **2008**, *29*, 724–734.

[12] a) E. van Lenthe, E. J. Baerends, *J. Comput. Chem.* **2003**, *24*, 1142–1156; b) D. P. Chong, E. van Lenthe, S. J. A. van Gisbergen, E. J. Baerends, *J. Comput. Chem.* **2004**, *25*, 1030–1036.

[13] S. K. Wolff, *Int. J. Quantum Chem.* **2005**, *104*, 645–659.

[14] M. Swart, *Chem. Phys. Lett.* **2013**, *580*, 166–171.

[15] A. Klamt, G. Schüürmann, *J. Chem. Soc., Perkin Trans. 2* **1993**, *2*, 799–805.

[16] M. Swart, E. Rosler, F. M. Bickelhaupt, *Eur. J. Inorg. Chem.* **2007**, 3646–3654.

[17] a) B. B. Averkiev, D. G. Truhlar, *Catal. Sci. Technol.* **2011**, *1*, 1526–1529; b) J. E. M. N. Klein, B. Dereli, L. Que, Jr., C. J. Cramer, *Chem. Commun.* **2016**, *52*, 10509–10512.

[18] E. van Lenthe, E. J. Baerends, J. G. Snijders, *J. Chem. Phys.* **1993**, *99*, 4597–4610.

[19] a) M. Franchini, P. H. T. Philipsen, L. Visscher, *J. Comput. Chem.* **2013**, *34*, 1819–1827; b) A. Becke, *J. Chem. Phys.* **1988**, *88*, 2547–2553.

[20] E. A. Hill, A. C. Weitz, E. Onderko, A. Romero-Rivera, Y. Guo, M. Swart, E. L. Bominaar, M. T. Green, M. P. Hendrich, D. C. Lacy, A. S. Borovik, *J. Am. Chem. Soc.* **2016**, *138*, 13143-13146.

[21] M. D. Wilkinson, M. Dumontier, I. J. Aalbersberg, *et al. Sci. Data* **2016**, *3*, 160018.

[22] P. Roussel, N. W. Alcock, P. Scott, *Inorg. Chem.* **1998**, *37*, 3435–3436.

[23] C. C. Cummins, J. Lee, R. R. Schrock, W. D. Davis, *Angew. Chem. Int. Ed. Engl.* **1992**, *31*, 1501–1503.

[24] F. Le Vaillant, M. D. Wodrich, J. Waser, *Chem. Sci.* **2017**, *8*, 1790–1800.

[25] M. Namazian, C. Y. Lin, M. L. Coote, *J. Chem. Theory Comput.* **2010**, *6*, 2721–2725.

[26] J. E. Bartmess, *J. Phys. Chem.* **1994**, *98*, 6420–6424.
